# Condition-Controlled Divergent Reactivity of Nitroaldol Isatin Adducts with Phenols: Friedel–Crafts Alkylation, Skeletal Rearrangement, and Spirocyclic Oxime Formation

**DOI:** 10.3390/ijms27209320

**Published:** 2026-10-20

**Authors:** Nikolai A. Arutiunov, Daria A. Shtal, Kirill V. Tolstov, Anastasia A. Korneeva, Olga V. Petrova, Anna M. Zatsepilina, Nicolai A. Aksenov, Sergei N. Ovcharov, Alexander V. Aksenov

**Affiliations:** Department of Chemistry, North Caucasus Federal University, 1a Pushkin St., 355017 Stavropol, Russia; shtall010404@gmail.com (D.A.S.); kirilltolstov17@mail.ru (K.V.T.); korneeva9801@gmail.com (A.A.K.); petrova-olgavik@yandex.ru (O.V.P.); ann.zazaza.2002@gmail.com (A.M.Z.); naksenov@ncfu.ru (N.A.A.); sovcharov@ncfu.ru (S.N.O.); aaksenov@ncfu.ru (A.V.A.)

**Keywords:** isatin, nitroaldol adducts, Friedel–Crafts alkylation, divergent reactivity, skeletal rearrangement, spirocyclic oximes, chromenoindolones

## Abstract

We describe the condition-controlled divergent reactivity of nitroaldol isatin adducts with phenols. Under methanesulfonic acid, nitroalcohols undergo Friedel–Crafts alkylation to afford 3-aryl-3-(nitromethyl)indolin-2-ones in yields of up to 99%. Prolonged acidic treatment of selected Friedel–Crafts adducts leads to an unusual C3-to-C2 skeletal rearrangement, providing tetracyclic chromenoindolones in moderate yields. In contrast, treatment of the alkylation products with KOH in aqueous ethanol promotes intramolecular cyclization to spirocyclic hydroxyimino derivatives. This transformation afforded 16 compounds in 80–96% yields, whereas five new chromenoindolones were obtained in 40–56% yields. In total, 51 new compounds were synthesized, comprising 30 Friedel–Crafts alkylation products, 16 spirocyclic oxime derivatives, and five new chromenoindolones. The structures of representative products **8aa** and **13ab** were established by X-ray crystallography. The Friedel–Crafts reaction tolerates a range of alkyl, halogen, hydroxy, alkoxy, and N-substituted substrates, although several strongly electron-withdrawing substituents and α-naphthol were not compatible with the reaction conditions. Scalability was demonstrated on a 10 mmol scale for the synthesis of **7ae** and its subsequent conversion to **13ae**. The results demonstrate that nitroaldol isatin adducts can undergo markedly different reaction pathways depending on the reaction conditions, providing access to structurally distinct indole-containing frameworks.

## 1. Introduction

Isatin and oxindole derivatives are versatile structural motifs in organic and medicinal chemistry and have been extensively employed as building blocks for the synthesis of structurally diverse heterocyclic systems. In particular, spirooxindoles, in which the oxindole core is fused to another ring system through the C3 carbon atom, have attracted considerable attention because of their occurrence in natural products and their broad representation in synthetic molecules with diverse biological activities [1,2,3,4,5,6]. The development of efficient methods for constructing spirocyclic and annulated oxindole frameworks therefore remains an important topic in synthetic chemistry.

Nitroaldol derivatives of isatin, including 3-hydroxy-3-(nitromethyl)indolin-2-ones and the corresponding 3-(nitromethylene)indolin-2-ones, represent a useful but comparatively underexplored class of isatin-derived building blocks. Their reactivity is governed by the coexistence of the oxindole carbonyl and nitroalkyl functionalities, which provides several potential reaction sites. The corresponding nitroalkenes can participate in nucleophilic addition and cycloaddition processes and have been described as electrophilic reaction partners in a variety of transformations [7,8]. Depending on the reaction mode, these substrates can undergo addition followed by elimination or further cyclization to furnish diverse heterocyclic products [9,10,11,12,13,14].

The reactivity of isatin-derived nitroalkenes toward phenols is particularly relevant to the present study. Previous reports have shown that phenols can participate in reactions with these substrates, including processes involving substitution at the carbon bearing the nitro group [12] (Scheme 1). In our previous work, 3-(2-nitrovinyl)indoles reacted with *p*-substituted phenols under acid-catalyzed conditions to give benzofuranone derivatives [15].

**Scheme 1:**
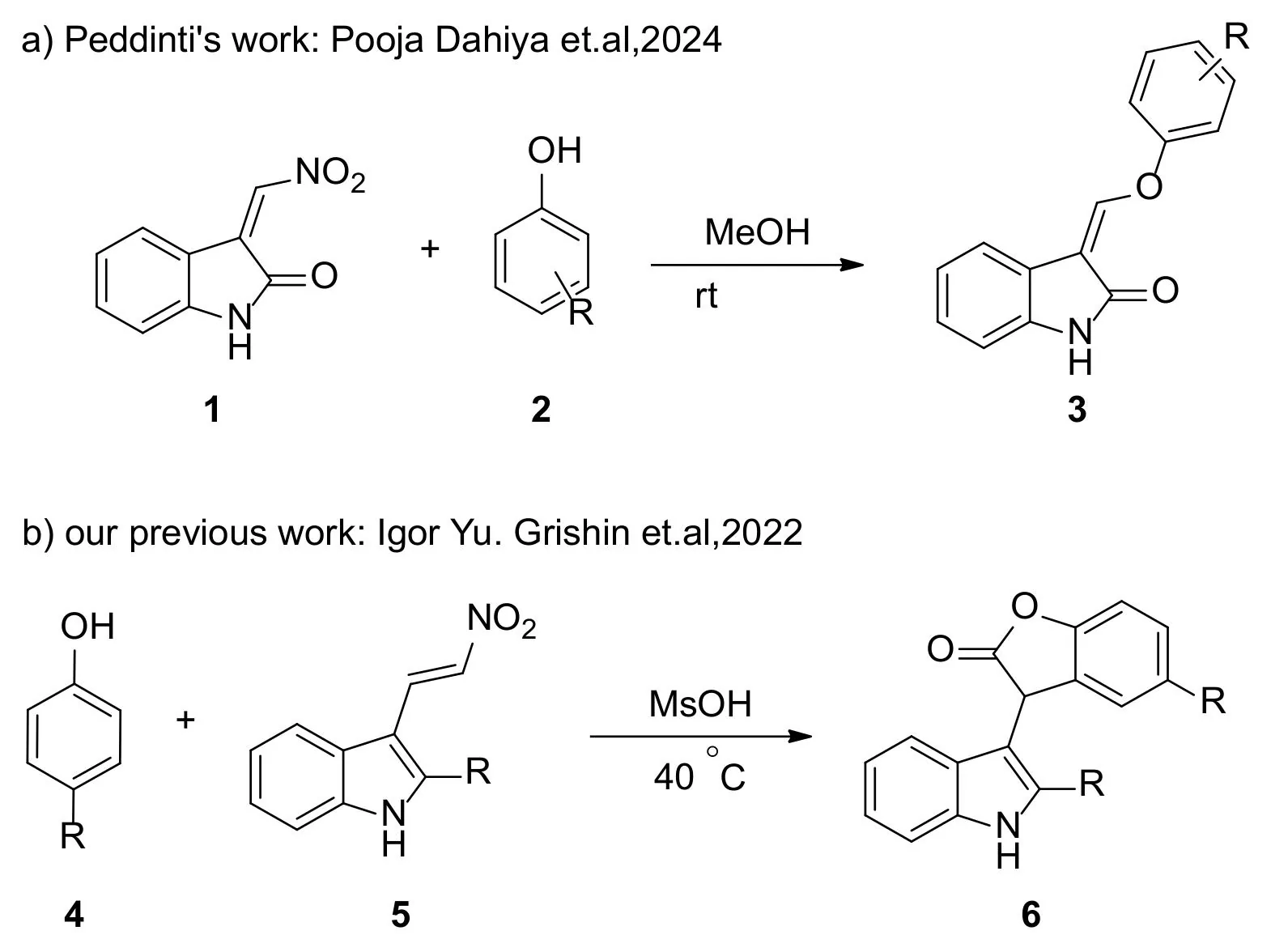
Previous examples of reactions of nitroalkene derivatives with phenols: (**A**) Peddinti’s work on ipso-substitutions of nitro-group [12]; (**B**) our previous work on the reaction of 3-(2-nitrovinyl)indole with phenol leading to benzofuranone derivatives [15].

These observations prompted us to examine whether the corresponding isatin-derived nitroalkene would display analogous reactivity or whether the structural differences of the oxindole system would lead to a different reaction pathway. Despite the substantial interest in isatin-derived nitroalkenes and the broad reactivity of isatin-based substrates, the direct use of nitroaldol isatin adducts as electrophilic partners in Friedel–Crafts alkylation of phenols has received little attention. More importantly, the relationship between the initial alkylation products and their subsequent transformations under acidic or basic conditions has not been systematically explored. We therefore sought to investigate the reaction of nitroaldol isatin adducts with phenols and to determine how the reaction conditions influence the structure of the resulting products.

In this work, we demonstrate that nitroaldol isatin adducts undergo regioselective Friedel–Crafts alkylation with phenols in the presence of methanesulfonic acid to give 3-aryl-3-(nitromethyl)indolin-2-ones. During the study, a divergent reaction pathway was identified under prolonged acidic treatment, leading to chromenoindolones through an unusual C3-to-C2 skeletal rearrangement. In contrast, exposure of the Friedel–Crafts adducts to KOH in aqueous ethanol resulted in intramolecular cyclization and formation of a previously unreported spirocyclic hydroxyimino scaffold. Thus, the same class of nitroaldol-derived intermediates can be directed toward structurally distinct products by changing the reaction conditions. The synthetic scope, limitations, stereochemical features of the spirocyclic oximes, and the experimental evidence relevant to the proposed rearrangement and cyclization pathways are discussed below.

## 2. Results and Discussion

### 2.1. Reaction Design and Discovery of the Friedel–Crafts Alkylation

We initially examined the reaction of isatin-derived nitroalkene **1a** with 4-methylphenol **2a** under acidic conditions, with the aim of determining whether this substrate could undergo the type of phenol addition suggested by the precedents discussed above. The reaction was performed in methanesulfonic acid at 70 °C. Complete consumption of the starting materials was observed after 1.5 h; however, the expected spirocyclic product was not formed. Instead, the reaction afforded a mixture of the Michael addition product **7aa** and chromenoindolone **8aa** (Scheme 2). The structure of **8aa** was unambiguously established by single-crystal X-ray diffraction analysis (Figure 1).

The formation of **7aa** indicated that the nitroalkene readily participated in nucleophilic addition with the phenol under the applied conditions, whereas the simultaneous formation of **8aa** revealed an additional reaction pathway that was not anticipated at the outset. Several variations of the acidic conditions were therefore examined. Weaker acids, including acetic, phosphoric, and phosphorous acids, did not result in detectable conversion of the starting materials, whereas the use of stronger acids, such as sulfuric or trifluoromethanesulfonic acid, led predominantly to resinification. These observations suggested that direct transformation of the nitroalkene was not a suitable preparative route to the desired products.

We therefore considered an alternative approach based on the corresponding nitroalcohols **9**, which are the precursors of nitroalkenes **1**. Under strongly acidic conditions, dehydration of the tertiary nitroalcohol could generate a highly electrophilic benzylic-type species capable of reacting with an electron-rich aromatic partner. This approach also avoids the separate preparation of the nitroalkene and thus provides a more direct entry to the desired C–C bond formation. To test this hypothesis, 3-hydroxy-3-(nitromethyl)indolin-2-one (**9a**) was reacted with 4-methylphenol (**2a**) in methanesulfonic acid at 70 °C (Scheme 3). After 1 h, the Friedel–Crafts alkylation product **7aa** was obtained in 85% yield. Chromenoindolone **8aa** was also detected as a minor product and isolated in 5% yield. The formation of **7aa** in substantially higher yield than that observed from the corresponding nitroalkene established nitroalcohols **9** as suitable substrates for the acid-promoted alkylation of phenols.

The initial experiment also revealed an important feature of the reaction: the chromenoindolone product can form under the same acidic conditions as the Friedel–Crafts adduct. We therefore treated the two products not as unrelated reaction outcomes, but as manifestations of condition-dependent reactivity of the same nitroaldol-derived substrate class. The scope of the Friedel–Crafts alkylation was subsequently investigated using structurally diverse phenols and nitroalcohols **9**. With the formation of the Friedel–Crafts product **7aa** established, we next examined the scope of the reaction with respect to both reaction partners. A series of phenols **2** and substituted nitroalcohols **9** was evaluated under the optimized conditions (Scheme 4).

The reaction accommodated a range of phenolic substrates bearing alkyl substituents. Thus, 4-substituted phenols generally reacted at the ortho-position relative to the hydroxy group, providing products **7aa**–**7ad** in good yields. Phenols containing halogen substituents were also compatible with the reaction conditions, affording products **7ae**, **7ai**, **7aj**, and **7am**. In addition, polyhydroxylated substrates such as pyrocatechol, resorcinol, and hydroquinone participated in the reaction to give products **7al**, **7af**, and **7ag**, respectively. 4-Hydroxyphenylacetic acid was also suitable as a reaction partner and afforded **7ah** in good yield.

The substrate scope also revealed several limitations. Phenols bearing strongly electron-withdrawing substituents directly attached to the aromatic ring, including cyano, nitro, and carboxy groups, did not provide the desired products under the applied conditions. 4-Aminophenol likewise failed to react (zero conversion of starting materials), whereas *o*-acetamidophenol afforded **7ao** in good yield. These observations indicate that the reaction requires a sufficiently nucleophilic aromatic system and that strongly deactivating or competing functional groups can interfere with the transformation.

We next investigated the influence of substitution within the nitroaldol-derived isatin fragment. The reaction tolerated several modifications of the isatin framework (Scheme 5). Nitroalcohols containing alkyl substituents on the nitrogen atom or at the C5 position of the aromatic ring afforded the corresponding Friedel–Crafts products in good yields, as exemplified by **7bb**, **7be**, **7eb**, and **7gb**. *N*-Allyl- and *N*-benzyl-substituted substrates also underwent the transformation to provide **7hb** and **7fb**, respectively. Introduction of a methoxy group or a bromine atom at the C5 position did not substantially alter the reaction outcome.

In contrast, the introduction of strongly electron-withdrawing substituents into the isatin-derived fragment was detrimental to the reaction. For nitroalcohols **9i**–**9l**, no desired Friedel–Crafts products were obtained; instead, the starting nitroalcohols underwent retro-Henry degradation to regenerate the corresponding isatins. This behavior represents an additional limitation of the method and highlights the sensitivity of the nitroalcohol substrates to the strongly acidic reaction conditions. Overall, the reaction provides access to a structurally diverse series of Friedel–Crafts adducts, although its applicability is influenced by both the electronic properties of the phenolic partner and the substitution pattern of the nitroalcohol component.

The substitution pattern of the phenolic component largely determined the regioselectivity of the transformation. In phenols bearing a para substituent, alkylation occurred exclusively at the ortho-position relative to the hydroxy group. When the para position was available, the corresponding para-substituted products were predominantly or exclusively formed under the standard reaction conditions. A distinct regioselective behavior was observed for 4-phenylphenol (**2u**), which contains two potentially reactive aromatic positions. Reaction with nitroalcohol **9a** under the standard conditions afforded a mixture of the ortho-substituted product **7′au** (21%) and the product **7au** (73%), resulting from substitution at the para position of the phenyl ring. Increasing the reaction temperature to 120 °C changed the product distribution, giving **7au** and **7′au** in 30% and 62% yields, respectively (Scheme 6). Thus, the reaction regioselectivity can be influenced by temperature in this substrate class, although the origin of this temperature dependence was not investigated further.

To further define the applicability of the Friedel–Crafts alkylation, we examined several electron-rich aromatic substrates lacking a phenolic hydroxy group. Anisole (**2v**) underwent the reaction to give **7av** in 51% yield, which was lower than the yields generally obtained with phenols. The lower efficiency may reflect the different reactivity of anisole under the strongly acidic conditions; reduced nucleophilicity relative to phenols may contribute, although additional factors cannot be excluded. In contrast, 1,2- and 1,3-dimethoxybenzenes (**2w** and **2x**) and benzodioxane (**2y**) afforded the corresponding products in good yields (Scheme 7). In the case of 4-bromoanisole (**2z**) the reaction afforded the *ortho*-substituted product **7az** in 66% yield. These results demonstrate that the reaction is not restricted to phenols and can also be applied to selected electron-rich alkoxyarenes.

We next investigated the behavior of α- and β-naphthols, whose fused aromatic systems provide additional opportunities for both electrophilic substitution and subsequent intramolecular transformations. Under the standard conditions, α-naphthol did not afford an isolable Friedel–Crafts product. Instead, the reaction was accompanied by extensive resinification, and lowering the reaction temperature did not provide an analytically useful product. In contrast, β-naphthol gave a single isolable product in 56% yield (Scheme 8).

Notably, the product obtained from β-naphthol was not the expected Friedel–Crafts adduct but chromenoindolone **8′az**. The corresponding alkylation product **7′az** was not detected in the reaction mixture. The structure of **8′az** indicates that the carbon originally bearing the nitro group has been incorporated into the C2 position of the oxindole framework, consistent with the C3-to-C2 skeletal rearrangement observed for the chromenoindolone series. This result suggested that the naphthyl substrate may favor the rearrangement pathway and prompted us to investigate the formation and scope of chromenoindolones **8** separately.

### 2.2. Acid-Promoted C3 → C2 Skeletal Rearrangement

The formation of chromenoindolone **8′az** from β-naphthol prompted us to investigate the origin and scope of this unusual product class. Because **8′az** was obtained directly from β-naphthol without detectable accumulation of the corresponding Friedel–Crafts adduct **7′az**, we considered whether the rearrangement might also occur from the initially formed alkylation products under prolonged acidic treatment. To examine this possibility, isolated Friedel–Crafts product **7aa** was treated with methanesulfonic acid at 70 °C. After 2 h, partial conversion of **7aa** into chromenoindolone **8aa** was observed, with the rearranged product obtained in 10% yield. Although the conversion was limited, this experiment demonstrated that an isolated Friedel–Crafts adduct can undergo conversion to the corresponding chromenoindolone under the reaction conditions and therefore provides experimental support for considering Friedel–Crafts products **7** as a precursor to products **8**. We next investigated the factors controlling the efficiency of this rearrangement using **7aa** as a model substrate (Table 1). Increasing the reaction temperature from 70 to 100 or 130 °C for 2 h increased the conversion from 12% to 30% and 43%, respectively, but the isolated yield of **8aa** remained low. The addition of POCl_3_ or PCl_5_ did not improve the reaction outcome and was accompanied by increased resinification. Extending the reaction time at 70 °C from 2 to 10 h increased the yield to 25%, while further prolongation to 24 h gave 32% yield at 57% conversion. Increasing the temperature to 80 °C under the same reaction time resulted in approximately 85% conversion and a 44% yield of **8aa**.

At 90 °C, complete conversion was achieved; however, the isolated yield decreased to 37%, which was accompanied by more pronounced resinification. Thus, prolonged heating at 80 °C provided the best balance between conversion and formation of the rearranged product. Under these conditions, we investigated the scope of the rearrangement (Scheme 9). A small series of chromeno[3,4-*b*]indol-6(7*H*)-ones **8** was obtained, comprising seven compounds in total, including five previously unreported derivatives. The products were isolated in moderate yields ranging from 40% to 56%. Variation of alkyl or halogen substituents in either structural fragment did not result in a pronounced change in the isolated yields. In contrast, the rearrangement was not observed for *N*-substituted isatin derivatives, for which the corresponding Friedel–Crafts products **7eb**, **7fb**, **7gb**, and **7hb** were obtained without detectable formation of chromenoindolones **8**. Although the rearrangement provides a route to a structurally distinct tetracyclic framework, its preparative efficiency is limited by the relatively low yields and the prolonged reaction times required. Its synthetic significance therefore lies primarily in the unusual skeletal reorganization of the nitroaldol-derived oxindole framework rather than in the development of a high-yielding general synthesis of chromenoindolones.

On the basis of the available experimental observations, a plausible pathway for the formation of chromenoindolones **8** is proposed in Scheme 10. The conversion of isolated **7aa** into **8aa** supports the involvement of the Friedel–Crafts adduct, but the individual intermediates proposed below were not directly observed. We therefore regard the following pathway as a mechanistic hypothesis rather than an established sequence. We propose that the rearrangement may begin with migration of the nitro-bearing carbon from C3 to C2 of the oxindole core to give intermediate **10**. Subsequent acid-promoted dehydration could generate conjugated quinone methide **11**.

This intermediate could then undergo oxa-6π-electrocyclization [16,17] to give intermediate **12**, followed by acid-promoted conversion of the nitro-derived functionality to furnish chromenoindolone **8**, plausibly through a Nef-type pathway. This proposal is consistent with the observed conversion of **7aa** into **8aa** under acidic conditions and with the structure of the rearranged products. However, HRMS analysis of the reaction mixture did not provide evidence for intermediates **10**–**12**. Consequently, the intermediacy of these species and the order of the individual elementary steps cannot be established from the present experimental data. Other mechanistic pathways cannot therefore be excluded. Further mechanistic studies, such as isotope-labeling or computational analysis, would be required to establish the origin of the migrating carbon atom and to distinguish between alternative rearrangement pathways.

### 2.3. Base-Promoted Cyclization to Spirocyclic Hydroxyimino Derivatives

The successful formation of the Friedel–Crafts adducts prompted us to investigate their reactivity under basic conditions. Because the alkylation products **7** contain both a phenolic hydroxy group and a nitroalkyl substituent attached to the same carbon atom of the oxindole framework, we considered that these functionalities might undergo an intramolecular transformation under strongly basic conditions. Accordingly, compound **7ab** was treated with 3 equivalents of KOH in an ethanol–water mixture. The reaction was complete within 2 h and afforded a single product in high yield. The product was clearly distinct from the chromenoindolones **8**. Its formation was accompanied by several characteristic spectroscopic changes. In the ^1^H NMR spectrum, the signal of one heteroatom-bound proton shifted from 9.91 to 10.45 ppm, while the signals corresponding to the methylene group adjacent to the nitro group disappeared. In the ^13^C NMR spectrum, the signal of the aliphatic methylene carbon was replaced by a downfield resonance at approximately 155 ppm. These observations were consistent with conversion of the nitroalkyl functionality into a new unsaturated nitrogen-containing group.

To establish the structure unambiguously, single crystals of the product were obtained and analyzed by X-ray diffraction. The analysis identified the compound as (*Z*)-5-ethyl-2-(hydroxyimino)-2*H*-spiro[benzofuran-3,3′-indolin]-2′-one (**13ab**, CCDC 2578801) (Figure 2).

Thus, under basic aqueous-alcoholic conditions, the Friedel–Crafts adduct **7ab** undergoes an intramolecular transformation that generates a spiro-fused benzofuran–oxindole framework while retaining the nitrogen-containing functionality in the form of a hydroxyimino group. On the basis of the observed structural changes, a plausible pathway for the formation of **13** is proposed in Scheme 11.

Deprotonation of the phenolic group under basic conditions may generate a phenolate nucleophile, while the nitroalkyl moiety can access a deprotonated or *aci*-nitro form. Intramolecular C–O bond formation between these functionalities [18,19,20] could then give spirocyclic intermediate **15**. Subsequent proton-transfer steps and conversion of the nitro-derived functionality could lead to a nitroso intermediate **17ab**, which may tautomerize to the corresponding hydroxyimino product **13**. The individual intermediates shown in Scheme 11 were not directly detected, and the proposed pathway should therefore be regarded as a mechanistic hypothesis. In particular, the intermediacy of a discrete nitroso species cannot be established from the present data, and alternative pathways for the nitro-to-oxime conversion cannot be excluded. The experimentally established features are the formation of a single spirocyclic product from **7ab** under KOH treatment and its structural assignment by X-ray crystallography.

The generality of this transformation was subsequently examined using a series of Friedel–Crafts adducts **7** derived from *p*-substituted phenols (Scheme 12). Sixteen spirocyclic hydroxyimino derivatives **13** were obtained in 80–96% yields. The reaction tolerated a range of substituents in both structural fragments. A moderate decrease in the isolated yield was observed for substrates containing halogen substituents on the phenolic moiety, whereas alkyl- and other substituent combinations generally afforded the corresponding spirocyclic products efficiently. The transformation therefore provides a concise conversion of the Friedel–Crafts adducts **7** into a previously unreported class of spirocyclic hydroxyimino compounds. Its primary synthetic significance lies in the efficient generation of the fused benzofuran–oxindole framework and in the preservation of the oxime functionality under the applied alkaline conditions.

The spirocyclic hydroxyimino products **13** were obtained predominantly as a single stereoisomer. The relative abundance of the isomers was assessed by ^1^H NMR spectroscopy from the integrations of the corresponding signals. For most compounds, the minor isomer did not exceed approximately 5%. The configuration of the oxime double bond was established for representative compound **13ab** by single-crystal X-ray diffraction, which unambiguously identified the major isomer as the *Z*-isomer (Figure 2). The configurations of the remaining members of the series were assigned by comparison of their spectroscopic data with those of structurally related compounds and with the NMR characteristics of crystallographically characterized **13ab** (Figure 3). The predominant formation of the Z-isomer may reflect the conformational and steric preferences of the spirocyclic framework, which can favor the arrangement of the oxime and amide carbonyl functionalities observed in the crystallographically characterized structure. Because no independent structural determination was performed for each member of the series, the stereochemical assignments of the remaining compounds should be regarded as assignments based on spectroscopic analogy rather than direct structural proof.

Two compounds, **13ah** and **13db**, showed substantially higher proportions of the minor isomer. Compound **13ah** was obtained as a Z/E mixture in a ratio of 5:3, whereas **13db** was formed with a Z/E ratio of 3.6:1. In both cases, the presence of the minor stereoisomer was evident from duplicated ^1^H NMR resonances, allowing the relative isomer ratios to be estimated by integration. The origin of this increased Z/E ratio is not established experimentally. The behavior of **13ah** may be associated with the presence of the carboxylic acid functionality, whereas the different distribution observed for **13db** may reflect the electronic effects of the methoxy substituent; however, these interpretations remain tentative. Taken together, the available X-ray and NMR data indicate a pronounced preference for the Z configuration throughout the spirocyclic oxime series, with substrate-dependent deviations observed for **13ah** and **13db**.

To assess the preparative applicability of the developed methodology, the Friedel–Crafts alkylation was performed on a 10 mmol scale using nitroalcohol **9a** and the corresponding phenolic partner (Scheme 13). Under the scale-up conditions shown in Scheme 13, Friedel–Crafts product **7ae** was obtained in 83% yield, corresponding to 2.76 g of isolated material. The isolated product **7ae** was subsequently subjected to the base-promoted cyclization under the modified conditions shown in Scheme 13. Spirocyclic hydroxyimino derivative 13ae was obtained in 74% yield after recrystallization from ethanol. This experiment demonstrates that the two-step sequence can be performed on a multigram scale without a substantial change in the reaction protocol. At the same time, because the scale-up was demonstrated for a single substrate, the present data support the practical scalability of the sequence rather than broad scalability across the entire substrate scope.

### 2.4. Limitations and Mechanistic Perspective

The present study demonstrates that nitroaldol isatin adducts display strongly condition-dependent reactivity toward electron-rich aromatic compounds. Under methanesulfonic acid conditions, the primary reaction pathway is Friedel–Crafts alkylation, whereas prolonged acidic treatment can lead to C3-to-C2 skeletal rearrangement. Under basic aqueous-alcoholic conditions, the same Friedel–Crafts adducts undergo intramolecular cyclization to spirocyclic hydroxyimino derivatives. The observed divergence therefore appears to arise from the different reaction environments imposed on the common nitroaldol-derived intermediates.

The synthetic scope is nevertheless subject to clear limitations. Strongly electron-withdrawing substituents directly attached to the phenolic ring were not compatible with the Friedel–Crafts reaction, 4-aminophenol did not react, and α-naphthol underwent extensive resinification. Several nitroalcohols containing electron-withdrawing substituents were also susceptible to retro-Henry degradation under the acidic conditions. The chromenoindolone rearrangement is additionally limited by moderate isolated yields and relatively long reaction times. These limitations indicate that the three reaction pathways are sensitive to both the electronic properties of the substrates and the reaction conditions.

The mechanistic pathways proposed for the C3-to-C2 rearrangement and the nitro-to-oxime conversion are consistent with the available experimental observations but remain provisional. Conversion of isolated **7aa** into **8aa** provides experimental support for the involvement of the Friedel–Crafts adduct in the rearrangement pathway; however, the proposed intermediates **10**–**12** were not detected by HRMS. Likewise, the proposed nitroso intermediate in the formation of **13** was not directly observed. Alternative pathways therefore cannot be excluded on the basis of the current data. Isotope-labeling experiments, mechanistic trapping, and computational studies could provide further insight into these transformations, particularly into the origin of the migrating carbon atom and the sequence of events leading from the nitroalkyl group to the oxime functionality.

## 3. Materials and Methods

### 3.1. General Information

^1^H, ^13^C, ^19^F NMR spectra were measured in solutions of CDCl_3_ on a Bruker AVANCE-III HD (Bruker BioSpin, Karlsruhe, Germany) instrument (at 400, 101, 376 MHz, respectively). Residual solvent signals were used as internal standards: in CDCl_3_ (7.26 ppm for ^1^H, and 77.16 ppm for ^13^C), in DMSO-*d*_6_ (2.5 ppm for ^1^H, and 39.52 ppm for ^13^C). HRMS spectra were measured on Bruker maXis impact (Bruker Daltonik, Bremen, Germany, electrospray ionization, in MeCN solutions, employing HCO_2_Na–HCO_2_H for calibration). X-Ray crystallography was performed on a Rigaku Oxford Diffraction SuperNova dual-source diffractometer (Cu microfocus X-ray source) equipped with an Atlas S2 CCD detector (Wrocław, Poland). The complete X-ray diffraction datasets were deposited at the Cambridge Crystallographic Data Centre (CCDC numbers 2578781 (**8aa**), 2578801 (**13ab**)). These data can be obtained free of charge *via* https://www.ccdc.cam.ac.uk/structures/ (accessed on 17 October 2026), or from the Cambridge Crystallographic Data Centre, 12 Union Road, Cambridge CB2 1EZ, UK fax: (þ44) 1223-336-033; or e-mail: deposit@ccdc.cam.ac.uk. Reaction progress, purity of isolated compounds, and R*_f_* values were monitored by TLC on on ALUGRAM RP-18 W/UV254 plates (Macherey-Nagel GmbH & Co. KG, Düren, Germany). Column chromatography was performed on silica gel (32–63 μm, 60 Å pore size). Melting points were measured with Stuart SMP30 apparatus (Antylia Scientific, Stone, UK). Starting phenols **4** and all other reagents and solvents were purchased from commercial vendors and were used as received. 3-Hydroxy-3-(nitromethyl)indolin-2-one **9a–9l** [21,22] and 3-(nitromethylene)indolin-2-one **3a** were synthesized according to the previously reported procedures and were identical to those described.

### 3.2. General Procedure for the Synthesis of 3-Aryl-3-(nitromethyl)indolin-2-One (Method A)

3-Hydroxy-3-(nitromethyl)indolin-2-one **9a** (104 mg, 0.50 mmol) and *p*-cresol **2a** (54 mg, 0.50 mmol) were placed into a 5 mL Erlenmeyer flask equipped with a magnetic stirrer, and 1 mL of MsOH was added. The resulting solution was stirred at 70 °C for one hour. After the reaction was complete (monitoring by TLC), the reaction mixture was poured into 50 mL of cold water and extracted with EtOAc (3 × 20 mL). The organic phase was evaporated under reduced pressure and the crude material was purified by column chromatography using hexane/EtOAc mixture as eluent.

3-(2-Hydroxy-5-methylphenyl)-3-(nitromethyl)indolin-2-one (**7aa**). This compound was prepared *via* **Method A** employing 3-hydroxy-3-(nitromethyl)indolin-2-one (**9a**) (104 mg, 0.50 mmol) and *p*-cresol (**2a**) (54 mg, 0.50 mmol) in a yield of 127 mg (0.43 mmol, 85%). Purification was performed by column chromatography (hexane/EtOAc, 3:1, *v*/*v*). The title compound was obtained as white solid, m.p. 181–183 °C, *R_f_* 0.52 (hexane/EtOAc, 2:1, *v*/*v*). ^1^H NMR (400 MHz, DMSO-*d*_6_) δ 10.80 (s, 1H), 9.89 (s, 1H), 7.51 (d, *J* = 7.3 Hz, 1H), 7.17 (dd, *J* = 7.7, 1.0 Hz, 1H), 6.95–6.88 (m, 2H), 6.87–6.82 (m, 2H), 6.71 (d, *J* = 8.1 Hz, 1H), 5.92 (d, *J* = 13.3 Hz, 1H), 5.31 (d, *J* = 13.2 Hz, 1H), 2.14 (s, 3H). ^13^C {^1^H} NMR (101 MHz, DMSO-*d*_6_) δ 177.6, 153.4, 142.5, 130.2, 129.6, 128.7, 128.2, 127.8, 124.4, 122.6, 121.7, 116.5, 109.7, 77.6, 54.9, 20.3. HRMS (ES TOF) *m*/*z*: (M + Na)^+^ Calcd for C_16_H_14_N_2_NaO_4_ 321.0846; Found 321.0850.

3-(5-Ethyl-2-hydroxyphenyl)-3-(nitromethyl)indolin-2-one (**7ab**). This compound was prepared *via* **Method A** employing 3-hydroxy-3-(nitromethyl)indolin-2-one (**9a**) (104 mg, 0.50 mmol) and 4-ethylphenol (**2b**) (61 mg, 0.50 mmol) in a yield of 136 mg (0.44 mmol, 87%). Purification was performed by column chromatography (hexane/EtOAc, 3:1, *v*/*v*). The title compound was obtained as white solid, m.p. 175–177 °C, *R_f_* 0.47 (hexane/EtOAc, 2:1, *v*/*v*). ^1^H NMR (400 MHz, DMSO-*d*_6_) δ 10.80 (s, 1H), 9.91 (s, 1H), 7.51 (d, *J* = 7.4 Hz, 1H), 7.21–7.14 (m, 1H), 6.97 (dd, *J* = 8.2, 1.9 Hz, 1H), 6.93–6.87 (m, 2H), 6.84 (d, *J* = 7.7 Hz, 1H), 6.73 (d, *J* = 8.2 Hz, 1H), 5.92 (d, *J* = 13.2 Hz, 1H), 5.32 (d, *J* = 13.2 Hz, 1H), 2.44 (q, *J* = 7.5 Hz, 2H), 1.08 (t, *J* = 7.6 Hz, 3H). ^13^C {^1^H} NMR (101 MHz, DMSO-*d*_6_) δ 177.6, 153.6, 142.5, 134.3, 130.1, 128.6, 128.3, 127.1, 124.4, 122.5, 121.6, 116.5, 109.7, 77.7, 54.9, 27.5, 15.9. HRMS (ES TOF) *m*/*z*: (M + Na)^+^ Calcd for C_17_H_16_N_2_NaO_4_ 335.1002; Found 335.1006.

3-(2-Hydroxy-4,5-dimethylphenyl)-3-(nitromethyl)indolin-2-one (**7ac**). This compound was prepared *via* **Method A** employing 3-hydroxy-3-(nitromethyl)indolin-2-one (**9a**) (104 mg, 0.50 mmol) and 3,4-dimethylphenol (**2c**) (61 mg, 0.50 mmol) in a yield of 126 mg (0.40 mmol, 81%). Purification was performed by column chromatography (hexane/EtOAc, 3:1, *v*/*v*). The title compound was obtained as white solid, m.p. 198–201 °C, *R_f_* 0.47 (hexane/EtOAc, 2:1, *v*/*v*). ^1^H NMR (400 MHz, DMSO-*d*_6_) δ 10.81 (s, 1H), 9.80 (s, 1H), 7.52 (d, *J* = 7.4 Hz, 1H), 7.17 (t, *J* = 7.6 Hz, 1H), 6.90 (t, *J* = 7.5 Hz, 1H), 6.85 (d, *J* = 7.7 Hz, 1H), 6.79 (s, 1H), 6.62 (s, 1H), 5.91 (d, *J* = 13.3 Hz, 1H), 5.30 (d, *J* = 13.3 Hz, 1H), 2.07 (s, 3H), 2.04 (s, 3H). ^13^C {^1^H} NMR (101 MHz, DMSO-*d*_6_) δ 177.8, 153.5, 142.5, 137.2, 130.4, 128.6 (2C), 126.6, 124.4, 121.7, 119.9, 118.0, 109.7, 77.7, 54.8, 19.1, 18.7. HRMS (ES TOF) *m*/*z*: (M + Na)^+^ Calcd for C_17_H_16_N_2_NaO_4_ 335.1002; Found 335.1012.

3-(2-Hydroxy-5-isopropylphenyl)-3-(nitromethyl)indolin-2-one (**7ad**). This compound was prepared *via* **Method A** employing 3-hydroxy-3-(nitromethyl)indolin-2-one (**9a**) (104 mg, 0.50 mmol) and 4-isopropylphenol (**2d**) (68 mg, 0.50 mmol) in a yield of 130 mg (0.40 mmol, 80%). Purification was performed by column chromatography (hexane/EtOAc, 3:1, *v*/*v*). The title compound was obtained as white solid, m.p. 105–107 °C, *R_f_* 0.50 (hexane/EtOAc, 2:1, *v*/*v*). ^1^H NMR (400 MHz, DMSO-*d*_6_) δ 10.82 (s, 1H), 9.93 (s, 1H), 7.53 (d, *J* = 7.3 Hz, 1H), 7.18 (td, *J* = 7.7, 1.0 Hz, 1H), 7.01 (dd, *J* = 8.3, 2.0 Hz, 1H), 6.96–6.88 (m, 2H), 6.85 (d, *J* = 7.7 Hz, 1H), 6.75 (d, *J* = 8.2 Hz, 1H), 5.94 (d, *J* = 13.3 Hz, 1H), 5.34 (d, *J* = 13.3 Hz, 1H), 2.72 (hept, *J* = 6.8 Hz, 1H), 1.10 (d, *J* = 6.9 Hz, 6H). ^13^C {^1^H} NMR (101 MHz, DMSO-*d*_6_) δ 177.7, 153.7, 142.6, 139.0, 130.2, 128.7, 126.7, 125.9, 124.5, 122.4, 121.7, 116.5, 109.7, 77.7, 55.1, 32.8, 24.1 (2C). HRMS (ES TOF) *m*/*z*: (M-H)^−^ Calcd for C_18_H_17_N_2_O_4_ 325.1194; Found 325.1194.

3-(5-Chloro-2-hydroxy-4-methylphenyl)-3-(nitromethyl)indolin-2-one (**7ae**). This compound was prepared *via* **Method A** employing 3-hydroxy-3-(nitromethyl)indolin-2-one (**9a**) (104 mg, 0.50 mmol) and 4-chloro-3-methylphenol (**2e**) (71 mg, 0.50 mmol) in a yield of 143 mg (0.43 mmol, 86%). Purification was performed by column chromatography (hexane/EtOAc, 3:1, *v*/*v*). The title compound was obtained as white solid, m.p. 162–164 °C, *R_f_* 0.52 (hexane/EtOAc, 2:1, *v*/*v*). ^1^H NMR (400 MHz, DMSO-*d*_6_) δ 10.85 (s, 1H), 10.30 (s, 1H), 7.45 (d, *J* = 7.4 Hz, 1H), 7.19 (t, *J* = 7.4 Hz, 1H), 7.06 (s, 1H), 6.92 (t, *J* = 7.5 Hz, 1H), 6.86 (d, *J* = 7.7 Hz, 1H), 6.75 (s, 1H), 5.83 (d, *J* = 13.2 Hz, 1H), 5.37 (d, *J* = 13.2 Hz, 1H), 2.18 (s, 3H). ^13^C {^1^H} NMR (101 MHz, DMSO-*d*_6_) δ 177.0, 154.5, 142.6, 136.3, 129.5, 128.9, 127.8, 124.4, 123.0, 122.5, 121.8, 118.8, 109.8, 77.6, 54.3, 19.3. HRMS (ES TOF) *m*/*z*: (M + Na)^+^ Calcd for C_16_H_13_ClN_2_NaO_4_ 355.0456; Found 355.0458.

3-(2,4-Dihydroxyphenyl)-3-(nitromethyl)indolin-2-one (**7af**). This compound was prepared *via* **Method A** employing 3-hydroxy-3-(nitromethyl)indolin-2-one (**9a**) (104 mg, 0.50 mmol) and resorcinol (**2f**) (55 mg, 0.50 mmol) in a yield of 149 mg (0.49 mmol, 99%). Purification was performed by column chromatography (hexane/EtOAc, 3:1, *v*/*v*). The title compound was obtained as white solid, m.p. 212–213 °C, *R_f_* 0.21 (hexane/EtOAc, 2:1, *v*/*v*). ^1^H NMR (400 MHz, DMSO-*d*_6_) δ 10.77 (s, 1H), 9.97 (s, 1H), 9.43 (s, 1H), 7.55–7.48 (m, 1H), 7.16 (t, *J* = 8.0 Hz, 1H), 6.91 (t, *J* = 7.2 Hz, 1H), 6.86–6.81 (m, 2H), 6.31 (d, *J* = 2.4 Hz, 1H), 6.19 (dd, *J* = 8.6, 2.4 Hz, 1H), 5.85 (d, *J* = 13.2 Hz, 1H), 5.29 (d, *J* = 13.2 Hz, 1H). ^13^C {^1^H} NMR (101 MHz, DMSO-*d*_6_) δ 178.0, 158.3, 156.8, 142.4, 130.6, 128.6, 128.5, 124.4, 121.6, 113.5, 109.7, 106.6, 103.8, 77.8, 54.6. HRMS (ES TOF) *m*/*z*: (M + Na)^+^ Calcd for C_15_H_12_N_2_NaO_5_ 323.0638; Found 323.0647.

3-(2,5-Dihydroxyphenyl)-3-(nitromethyl)indolin-2-one (**7ag**). This compound was prepared *via* **Method A** employing 3-hydroxy-3-(nitromethyl)indolin-2-one (**9a**) (104 mg, 0.50 mmol) and hydroquinone (**2g**) (55 mg, 0.50 mmol) in a yield of 135 mg (0.45 mmol, 90%). Purification was performed by column chromatography (hexane/EtOAc, 3:1, *v*/*v*). The title compound was obtained as yellow oil, *R_f_* 0.24 (hexane/EtOAc, 2:1, *v*/*v*). ^1^H NMR (400 MHz, DMSO-*d*_6_) δ 10.88 (s, 1H), 9.53 (s, 1H), 8.88 (s, 1H), 7.64 (d, *J* = 7.4 Hz, 1H), 7.19 (t, *J* = 7.7 Hz, 1H), 6.94 (t, *J* = 7.6 Hz, 1H), 6.88 (d, *J* = 7.7 Hz, 1H), 6.69 (d, *J* = 8.6 Hz, 1H), 6.57 (dd, *J* = 8.6, 2.8 Hz, 1H), 6.51 (d, *J* = 2.8 Hz, 1H), 5.96 (d, *J* = 13.5 Hz, 1H), 5.37 (d, *J* = 13.5 Hz, 1H). ^13^C {^1^H} NMR (101 MHz, DMSO-*d*_6_) δ 177.8, 150.1, 148.3, 142.4, 130.2, 128.8, 124.7, 123.0, 121.9, 117.6, 115.7, 114.8, 109.9, 77.2, 55.4. HRMS (ES TOF) *m*/*z*: (M + Na)^+^ Calcd for C_15_H_12_N_2_NaO_5_ 323.0638; Found 323.0636.

2-(4-Hydroxy-3-(3-(nitromethyl)-2-oxoindolin-3-yl)phenyl)acetic acid (**7ah**). This compound was prepared *via* **Method A** employing 3-hydroxy-3-(nitromethyl)indolin-2-one (**9a**) (104 mg, 0.50 mmol) and 2-(4-hydroxyphenyl)acetic acid (**2h**) (76 mg, 0.50 mmol) in a yield of 128 mg (0.37 mmol, 75%). Purification was performed by column chromatography (hexane/EtOAc, 3:1, *v*/*v*). The title compound was obtained as white solid, m.p. 198–200 °C, *R_f_* 0.06 (hexane/EtOAc, 2:1, *v*/*v*). ^1^H NMR (400 MHz, DMSO-*d*_6_) δ 12.26 (s, 1H), 10.82 (s, 1H), 10.10 (s, 1H), 7.52 (d, *J* = 7.5 Hz, 1H), 7.18 (t, *J* = 7.6 Hz, 1H), 7.02 (dd, *J* = 8.2, 1.7 Hz, 1H), 6.97–6.93 (m, 1H), 6.90 (t, *J* = 7.6 Hz, 1H), 6.84 (d, *J* = 7.7 Hz, 1H), 6.76 (d, *J* = 8.2 Hz, 1H), 5.92 (d, *J* = 13.3 Hz, 1H), 5.30 (d, *J* = 13.3 Hz, 1H). ^13^C {^1^H} NMR (101 MHz, DMSO-*d*_6_) δ 177.4, 173.0, 154.4, 142.5, 130.2, 130.0, 128.7 (2C), 125.7, 124.4, 122.6, 121.7, 116.5, 109.7, 77.6, 54.9, 40.0. HRMS (ES TOF) *m*/*z*: (M + 2Na-H)^+^ Calcd for C_17_H_13_N_2_Na_2_O_6_ 387.0564; Found 387.0565.

3-(5-Fluoro-2-hydroxyphenyl)-3-(nitromethyl)indolin-2-one (**7ai**). This compound was prepared *via* **Method A** employing 3-hydroxy-3-(nitromethyl)indolin-2-one (**9a**) (104 mg, 0.50 mmol) and 4-fluorophenol (**2i**) (56 mg, 0.50 mmol) in a yield of 124 mg (0.41 mmol, 82%). Purification was performed by column chromatography (hexane/EtOAc, 3:1, *v*/*v*). The title compound was obtained as white solid, m.p. 198–201 °C, *R_f_* 0.45 (hexane/EtOAc, 2:1, *v*/*v*). ^1^H NMR (400 MHz, DMSO-*d*_6_) δ 10.85 (s, 1H), 10.08 (s, 1H), 7.43 (d, *J* = 7.4 Hz, 1H), 7.24–7.16 (m, 1H), 7.00 (td, *J* = 8.4, 3.1 Hz, 1H), 6.98–6.94 (m, 1H), 6.94–6.89 (m, 1H), 6.87 (d, *J* = 7.7 Hz, 1H), 6.79 (dd, *J* = 8.8, 5.1 Hz, 1H), 5.83 (d, *J* = 13.0 Hz, 1H), 5.40 (d, *J* = 13.0 Hz, 1H). ^13^C {^1^H} NMR (101 MHz, DMSO-*d*_6_) δ 177.0, 155.3 (d, *J* = 233.9 Hz), 152.0, 142.8, 129.4, 129.0, 124.4, 124.3 (d, *J* = 7.1 Hz), 121.8, 117.2 (d, *J* = 8.1 Hz), 115.5 (d, *J* = 22.4 Hz), 114.7 (d, *J* = 25.2 Hz), 109.8, 77.8, 54.5.^19^F NMR (376 MHz, DMSO-*d*_6_) δ −124.91. HRMS (ES TOF) *m*/*z*: (M + Na)^+^ Calcd for C_15_H_11_FN_2_NaO_4_ 325.0595; Found 325.0594.

3-(3-Bromo-2-hydroxy-5-methylphenyl)-3-(nitromethyl)indolin-2-one (**7aj**). This compound was prepared *via* **Method A** employing 3-hydroxy-3-(nitromethyl)indolin-2-one (**9a**) (104 mg, 0.50 mmol) and 2-bromo-4-methylphenol (**2j**) (93 mg, 0.50 mmol) in a yield of 147 mg (0.39 mmol, 78%). Purification was performed by column chromatography (hexane/EtOAc, 3:1, *v*/*v*). The title compound was obtained as white solid, m.p. 192–194 °C, *R_f_* 0.55 (hexane/EtOAc, 2:1, *v*/*v*). ^1^H NMR (400 MHz, DMSO-*d*_6_) δ 11.03 (s, 1H), 9.92 (s, 1H), 7.43 (d, *J* = 7.4 Hz, 1H), 7.34 (s, 1H), 7.24 (t, *J* = 7.5 Hz, 1H), 6.99 (t, *J* = 7.5 Hz, 1H), 6.93–6.88 (m, 2H), 5.72 (d, *J* = 13.2 Hz, 1H), 5.52 (d, *J* = 13.2 Hz, 1H), 2.16 (s, 3H). ^13^C {^1^H} NMR (101 MHz, DMSO-*d*_6_) δ 178.1, 149.9, 142.6, 133.1, 130.6, 129.2, 128.8, 128.2, 125.5, 124.8, 122.1, 112.8, 110.3, 77.8, 55.3, 20.0. HRMS (ES TOF) *m*/*z*: (M + Na)^+^ Calcd for C_16_H_13_BrN_2_NaO_4_ 398.9951; Found 398.9961.

3-(4-Hydroxyphenyl)-3-(nitromethyl)indolin-2-one (**7ak**). This compound was prepared *via* **Method A** employing 3-hydroxy-3-(nitromethyl)indolin-2-one (**9a**) (104 mg, 0.50 mmol) and phenol (**2k**) (47 mg, 0.50 mmol) in a yield of 134 mg (0.47 mmol, 94%). Purification was performed by column chromatography (hexane/EtOAc, 3:1, *v*/*v*). The title compound was obtained as white solid, m.p. 154–156 °C, *R_f_* 0.36 (hexane/EtOAc, 2:1, *v*/*v*). ^1^H NMR (400 MHz, DMSO-*d*_6_) δ 10.68 (s, 1H), 9.62 (s, 1H), 7.48 (d, *J* = 7.3 Hz, 1H), 7.27 (t, *J* = 7.7 Hz, 1H), 7.13 (d, *J* = 8.8 Hz, 2H), 7.04 (t, *J* = 7.4 Hz, 1H), 6.94 (d, *J* = 7.7 Hz, 1H), 6.75 (d, *J* = 8.8 Hz, 2H), 5.60 (d, *J* = 14.2 Hz, 1H), 5.47 (d, *J* = 14.2 Hz, 1H). ^13^C {^1^H} NMR (101 MHz, DMSO-*d*_6_) δ 177.6, 157.4, 142.7, 129.2, 129.1, 128.0 (2C), 126.4, 124.9, 121.9, 115.6 (2C), 110.2, 78.6, 54.4. HRMS (ES TOF) *m*/*z*: (M + Na)^+^ Calcd for C_15_H_12_N_2_NaO_4_ 307.0689; Found 307.0689.

3-(3,4-Dihydroxyphenyl)-3-(nitromethyl)indolin-2-one (**7al**). This compound was prepared *via* **Method A** employing 3-hydroxy-3-(nitromethyl)indolin-2-one (**9a**) (104 mg, 0.50 mmol) and pyrocatechol (**2l**) (55 mg, 0.50 mmol) in a yield of 140 mg (0.47 mmol, 93%). Purification was performed by column chromatography (hexane/EtOAc, 3:1, *v*/*v*). The title compound was obtained as yellow oil, *R_f_* 0.12 (hexane/EtOAc, 2:1, *v*/*v*). ^1^H NMR (400 MHz, DMSO-*d*_6_) δ 10.65 (s, 1H), 9.10 (s, 1H), 9.07 (s, 1H), 7.46 (d, *J* = 7.3 Hz, 1H), 7.27 (t, *J* = 8.0 Hz, 1H), 7.04 (t, *J* = 7.4 Hz, 1H), 6.94 (d, *J* = 7.7 Hz, 1H), 6.78–6.67 (m, 2H), 6.63 (dd, *J* = 8.4, 2.3 Hz, 1H), 5.54 (d, *J* = 14.1 Hz, 1H), 5.45 (d, *J* = 14.1 Hz, 1H). ^13^C {^1^H} NMR (101 MHz, DMSO-*d*_6_) δ 177.6, 145.47, 145.45, 142.7, 129.2, 129.1, 126.9, 125.0, 121.9, 117.9, 115.8, 114.4, 110.2, 78.7, 54.4. HRMS (ES TOF) *m*/*z*: (M + Na)^+^ Calcd for C_15_H_12_N_2_NaO_5_ 323.0638; Found 323.0630.

3-(2,5-Dichloro-4-hydroxyphenyl)-3-(nitromethyl)indolin-2-one (**7am**). This compound was prepared *via* **Method A** employing 3-hydroxy-3-(nitromethyl)indolin-2-one (**9a**) (104 mg, 0.50 mmol) and 2,5-dichlorophenol (**2m**) (81 mg, 0.50 mmol) in a yield of 155 mg (0.44 mmol, 88%). Purification was performed by column chromatography (hexane/EtOAc, 3:1, *v*/*v*). The title compound was obtained as white solid, m.p. 127–129 °C, *R_f_* 0.27 (hexane/EtOAc, 2:1, *v*/*v*). ^1^H NMR (400 MHz, DMSO-*d*_6_) δ 10.90 (br. s, 2H), 7.57 (s, 1H), 7.26–7.20 (m, 1H), 7.07 (d, *J* = 7.3 Hz, 1H), 6.96–6.92 (m, 2H), 6.86 (d, *J* = 7.8 Hz, 1H), 5.60 (d, *J* = 12.1 Hz, 1H), 5.51 (d, *J* = 12.1 Hz, 1H). ^13^C {^1^H} NMR (101 MHz, DMSO-*d*_6_) δ 175.6, 153.5, 143.2, 131.3, 130.9, 129.5, 128.4, 125.9, 124.4, 122.2, 118.7, 118.6, 110.0, 80.0, 54.7. HRMS (ES TOF) *m*/*z*: (M + Na)^+^ Calcd for C_15_H_10_ClN_2_NaO_4_ 374.9910; Found 374.9912.

3-(3-Benzyl-4-hydroxyphenyl)-3-(nitromethyl)indolin-2-one (**7an**). This compound was prepared *via* **Method A** employing 3-hydroxy-3-(nitromethyl)indolin-2-one (**9a**) (104 mg, 0.50 mmol) and 2-benzylphenol (**2n**) (92 mg, 0.50 mmol) in a yield of 170 mg (0.46 mmol, 91%). Purification was performed by column chromatography (hexane/EtOAc, 3:1, *v*/*v*). The title compound was obtained as white solid, m.p. 172–174 °C, *R_f_* 0.24 (hexane/EtOAc, 2:1, *v*/*v*). ^1^H NMR (400 MHz, DMSO-*d*_6_) δ 10.62 (s, 1H), 9.62 (s, 1H), 7.43 (d, *J* = 7.4 Hz, 1H), 7.28–7.19 (m, 3H), 7.18–7.09 (m, 4H), 7.00 (t, *J* = 7.5 Hz, 1H), 6.94–6.87 (m, 2H), 6.74 (dd, *J* = 8.4, 3.0 Hz, 1H), 5.56 (d, *J* = 14.2 Hz, 1H), 5.46 (d, *J* = 14.3 Hz, 1H), 3.80 (s, 2H). ^13^C {^1^H} NMR (101 MHz, DMSO-*d*_6_) δ 177.4, 155.0, 142.6, 140.9, 129.1, 129.0 (2C), 128.6 (2C), 128.2 (2C), 127.7, 126.4, 125.8 (2C), 124.8, 121.8, 115.2, 110.1, 78.6, 54.3, 35.7. HRMS (ES TOF) *m*/*z*: (M + Na)^+^ Calcd for C_22_H_18_N_2_NaO_4_ 397.1159; Found 397.1162.

*N*-(2-Hydroxy-5-(3-(nitromethyl)-2-oxoindolin-3-yl)phenyl)acetamide (**7ao**). This compound was prepared *via* **Method A** employing 3-hydroxy-3-(nitromethyl)indolin-2-one (**9a**) (104 mg, 0.50 mmol) and *N*-(2-hydroxyphenyl)acetamide (**2o**) (75 mg, 0.50 mmol) in a yield of 162 mg (0.48 mmol, 95%). Purification was performed by column chromatography (hexane/EtOAc, 3:1, *v*/*v*). The title compound was obtained as white solid, m.p. 195–197 °C, *R_f_* 0.09 (hexane/EtOAc, 2:1, *v*/*v*). ^1^H NMR (400 MHz, DMSO-*d*_6_) δ 10.67 (s, 1H), 9.94 (s, 1H), 9.40 (s, 1H), 7.56 (d, *J* = 2.2 Hz, 1H), 7.45 (d, *J* = 7.4 Hz, 1H), 7.29–7.24 (m, 1H), 7.05–6.98 (m, 2H), 6.92 (d, *J* = 7.7 Hz, 1H), 6.84 (d, *J* = 8.6 Hz, 1H), 5.51 (d, *J* = 14.2 Hz, 1H), 5.46 (d, *J* = 14.1 Hz, 1H), 2.05 (s, 3H). ^13^C {^1^H} NMR (101 MHz, DMSO-*d*_6_) δ 177.3, 169.3, 148.1, 142.6, 129.1, 129.0, 126.7, 126.5, 124.9, 123.3, 121.9, 121.2, 116.3, 110.1, 78.6, 54.3, 23.5. HRMS (ES TOF) *m*/*z*: (M + Na)^+^ Calcd for C_17_H_15_N_3_NaO_5_ 364.0904; Found 364.0912.

3-(4′-Hydroxy-[1,1′-biphenyl]-4-yl)-3-(nitromethyl)indolin-2-one (**7au**). This compound was prepared *via* **Method A** employing 3-hydroxy-3-(nitromethyl)indolin-2-one (**9a**) (104 mg, 0.50 mmol) and [1,1′-biphenyl]-4-ol (**2u**) (85 mg, 0.50 mmol) in a yield of 131 mg (0.36 mmol, 73%) and 54 mg (0.15 mmol, 30%) when the reaction was performed at 120 °C. Purification was performed by column chromatography (hexane/EtOAc, 3:1, *v*/*v*). The title compound was obtained as white solid, m.p. 118–119 °C, *R_f_* 0.24 (hexane/EtOAc, 2:1, *v*/*v*). ^1^H NMR (400 MHz, DMSO-*d*_6_) δ 10.78 (s, 1H), 9.64 (s, 1H), 7.55 (d, *J* = 8.5 Hz, 2H), 7.52 (d, *J* = 7.7 Hz, 1H), 7.46 (d, *J* = 8.6 Hz, 2H), 7.34–7.28 (m, 3H), 7.06 (t, *J* = 7.5 Hz, 1H), 6.97 (d, *J* = 7.7 Hz, 1H), 6.85 (d, *J* = 8.6 Hz, 2H), 5.71 (d, *J* = 14.2 Hz, 1H), 5.60 (d, *J* = 14.2 Hz, 1H). ^13^C {^1^H} NMR (101 MHz, DMSO-*d*_6_) δ 177.1, 157.5, 142.8, 140.1, 134.4, 130.1, 129.3, 128.8, 127.9 (2C), 127.2 (2C), 126.4 (2C), 125.0, 122.0, 115.9 (2C), 110.3, 78.3, 54.8. HRMS (ES TOF) *m*/*z*: (M + Na)^+^ Calcd for C_21_H_16_N_2_NaO_4_ 383.1002; Found 383.1004.

3-(4-Hydroxy-[1,1′-biphenyl]-3-yl)-3-(nitromethyl)indolin-2-one (**7′au**). This compound was prepared *via* **Method A** employing 3-hydroxy-3-(nitromethyl)indolin-2-one (**9a**) (104 mg, 0.50 mmol) and [1,1′-biphenyl]-4-ol (**2u**) (85 mg, 0.50 mmol) in a yield of 38 mg (0.11 mmol, 21%) and 112 mg (0.31 mmol, 62%) when the reaction was performed at 120 °C. Purification was performed by column chromatography (hexane/EtOAc, 3:1, *v*/*v*). The title compound was obtained as white solid, m.p. 187–189 °C, *R_f_* 0.46 (hexane/EtOAc, 2:1, *v*/*v*). ^1^H NMR (400 MHz, DMSO-*d*_6_) δ 10.83 (s, 1H), 10.24 (s, 1H), 7.52 (d, *J* = 7.4 Hz, 2H), 7.48 (d, *J* = 7.4 Hz, 1H), 7.46–7.39 (m, 3H), 7.37–7.35 (m, 1H), 7.29 (t, *J* = 7.3 Hz, 1H), 7.19 (t, *J* = 7.3 Hz, 1H), 6.95–6.87 (m, 2H), 6.85 (d, *J* = 7.7 Hz, 1H), 5.96 (d, *J* = 13.0 Hz, 1H), 5.46 (d, *J* = 13.0 Hz, 1H). ^13^C {^1^H} NMR (101 MHz, DMSO-*d*_6_) δ 177.4, 155.3, 142.7, 140.1, 131.3, 129.9, 128.9 (2C), 128.7, 127.5, 126.7, 126.4, 126.2 (2C), 124.4, 123.6, 121.6, 117.0, 109.6, 78.0, 54.7. HRMS (ES TOF) *m*/*z*: (M + Na)^+^ Calcd for C_21_H_16_N_2_NaO_4_ 383.1002; Found 383.1009.

3-(5-Ethyl-2-hydroxyphenyl)-5-methyl-3-(nitromethyl)indolin-2-one (**7bb**). This compound was prepared *via* **Method A** employing 3-hydroxy-5-methyl-3-(nitromethyl)indolin-2-one (**9b**) (111 mg, 0.50 mmol) and 4-ethylphenol (**2b**) (61 mg, 0.50 mmol) in a yield of 142 mg (0.44 mmol, 87%). Purification was performed by column chromatography (hexane/EtOAc, 3:1, *v*/*v*). The title compound was obtained as white solid, m.p. 134–137 °C, *R_f_* 0.47 (hexane/EtOAc, 2:1, *v*/*v*). ^1^H NMR (400 MHz, DMSO-*d*_6_) δ 10.70 (s, 1H), 9.89 (s, 1H), 7.34 (s, 1H), 7.00–6.95 (m, 2H), 6.89 (d, *J* = 1.9 Hz, 1H), 6.73 (d, *J* = 8.0 Hz, 2H), 5.92 (d, *J* = 13.2 Hz, 1H), 5.30 (d, *J* = 13.2 Hz, 1H), 2.44 (q, *J* = 7.5 Hz, 2H), 2.19 (s, 3H), 1.09 (t, *J* = 7.6 Hz, 3H). ^13^C {^1^H} NMR (101 MHz, DMSO-*d*_6_) δ 177.6, 153.5, 140.1, 134.3, 130.4, 130.3, 128.9, 128.2, 127.1, 124.9, 122.7, 116.5, 109.4, 77.7, 55.0 27.5, 20.9, 15.9. HRMS (ES TOF) *m*/*z*: (M + Na)^+^ Calcd for C_18_H_18_N_2_NaO_4_ 349.1159; Found 349.1167.

3-(5-Chloro-2-hydroxy-4-methylphenyl)-5-methyl-3-(nitromethyl)indolin-2-one (**7be**). This compound was prepared *via* **Method A** employing 3-hydroxy-5-methyl-3-(nitromethyl)indolin-2-one (**9b**) (111 mg, 0.50 mmol) and 4-chloro-3-methylphenol (**2e**) (71 mg, 0.50 mmol) in a yield of 154 mg (0.45 mmol, 89%). Purification was performed by column chromatography (hexane/EtOAc, 3:1, *v*/*v*). The title compound was obtained as white solid, m.p. 110–112 °C, *R_f_* 0.16 (hexane/EtOAc, 2:1, *v*/*v*). ^1^H NMR (400 MHz, DMSO-*d*_6_) δ 10.73 (s, 1H), 10.25 (s, 1H), 7.26 (s, 1H), 7.05 (s, 1H), 6.99 (d, *J* = 7.9 Hz, 1H), 6.80–6.66 (m, 2H), 5.79 (d, *J* = 13.1 Hz, 1H), 5.34 (d, *J* = 13.2 Hz, 1H), 2.19 (s, 6H). ^13^C {^1^H} NMR (101 MHz, DMSO-*d*_6_) δ 176.9, 154.4, 140.2, 136.2, 130.5, 129.7, 129.1, 127.8, 124.9, 122.9, 122.6, 118.8, 109.5, 77.6, 54.2, 20.8, 19.3. HRMS (ES TOF) *m*/*z*: (M + Na)^+^ Calcd for C_17_H_15_ClN_2_NaO_4_ 369.0613; Found 369.0645.

5-Bromo-3-(5-ethyl-2-hydroxyphenyl)-3-(nitromethyl)indolin-2-one (**7cb**). This compound was prepared *via* **Method A** employing 5-bromo-3-hydroxy-3-(nitromethyl)indolin-2-one (**9c**) (143 mg, 0.50 mmol) and 4-ethylphenol (**2b**) (61 mg, 0.50 mmol) in a yield of 154 mg (0.39 mmol, 79%). Purification was performed by column chromatography (hexane/EtOAc, 3:1, *v*/*v*). The title compound was obtained as white solid, m.p. 132–134 °C, *R_f_* 0.49 (hexane/EtOAc, 2:1, *v*/*v*). ^1^H NMR (400 MHz, DMSO-*d*_6_) δ 10.97 (s, 1H), 9.99 (s, 1H), 7.71 (d, *J* = 2.0 Hz, 1H), 7.36 (dd, *J* = 8.3, 2.0 Hz, 1H), 6.99 (dd, *J* = 8.2, 1.8 Hz, 1H), 6.93 (d, *J* = 1.8 Hz, 1H), 6.83 (d, *J* = 8.3 Hz, 1H), 6.75 (d, *J* = 8.2 Hz, 1H), 6.01 (d, *J* = 13.5 Hz, 1H), 5.32 (d, *J* = 13.4 Hz, 1H), 2.46 (q, *J* = 7.6 Hz, 2H), 1.10 (t, *J* = 7.6 Hz, 3H). ^13^C {^1^H} NMR (101 MHz, DMSO-*d*_6_) δ 177.1, 153.4, 142.1, 134.4, 132.8, 131.3, 128.6, 127.1 (2C), 122.1, 116.5, 113.2, 111.6, 77.6, 54.8, 27.6, 15.9. HRMS (ES TOF) *m*/*z*: (M-H)^−^ Calcd for C_17_H_14_BrN_2_O_4_ 389.0142; Found 389.0152.

5-Bromo-3-(5-chloro-2-hydroxy-4-methylphenyl)-3-(nitromethyl)indolin-2-one (**7ce**). This compound was prepared *via* **Method A** employing 5-bromo-3-hydroxy-3-(nitromethyl)indolin-2-one (**9c**) (143 mg, 0.50 mmol) and 4-chloro-3-methylphenol (**2e**) (71 mg, 0.50 mmol) in a yield of 164 mg (0.40 mmol, 80%). Purification was performed by column chromatography (hexane/EtOAc, 3:1, *v*/*v*). The title compound was obtained as white solid, m.p. 127–129 °C, *R_f_* 0.38 (hexane/EtOAc, 2:1, *v*/*v*). ^1^H NMR (400 MHz, DMSO-*d*_6_) δ 10.99 (s, 1H), 10.34 (s, 1H), 7.64 (d, *J* = 1.8 Hz, 1H), 7.37 (dd, *J* = 8.3, 2.0 Hz, 1H), 7.09 (s, 1H), 6.82 (d, *J* = 8.3 Hz, 1H), 6.74 (s, 1H), 5.90 (d, *J* = 13.3 Hz, 1H), 5.36 (d, *J* = 13.3 Hz, 1H), 2.19 (s, 3H). ^13^C {^1^H} NMR (101 MHz, DMSO-*d*_6_) δ 176.6, 154.2, 142.2, 136.6, 132.1, 131.5, 127.8, 127.0, 123.1, 122.0, 118.7, 113.3, 111.6, 77.5, 54.0, 19.3. HRMS (ES TOF) *m*/*z*: (M + Na)^+^ Calcd for C_16_H_12_BrClN_2_NaO_4_ 432.9561; Found 432.9558.

3-(5-Ethyl-2-hydroxyphenyl)-5-methoxy-3-(nitromethyl)indolin-2-one (**7db**). This compound was prepared *via* **Method A** employing 3-hydroxy-5-methoxy-3-(nitromethyl)indolin-2-one (**9d**) (119 mg, 0.50 mmol) and 4-ethylphenol (**2b**) (61 mg, 0.50 mmol) in a yield of 142 mg (0.42 mmol, 83%). Purification was performed by column chromatography (hexane/EtOAc, 3:1, *v*/*v*). The title compound was obtained as white solid, m.p. 77–79 °C, *R_f_* 0.27 (hexane/EtOAc, 2:1, *v*/*v*). ^1^H NMR (400 MHz, DMSO-*d*_6_) δ 10.66 (s, 1H), 9.97 (s, 1H), 7.23 (s, 1H), 6.98 (d, *J* = 7.1 Hz, 1H), 6.87 (s, 1H), 6.83–6.70 (m, 3H), 5.94 (d, *J* = 13.4 Hz, 1H), 5.30 (d, *J* = 13.4 Hz, 1H), 3.65 (s, 3H), 2.44 (q, *J* = 7.5 Hz, 2H), 1.08 (t, *J* = 7.5 Hz, 3H). ^13^C {^1^H} NMR (101 MHz, DMSO-*d*_6_) δ 177.5, 154.8, 153.7, 135.8, 134.5, 131.6, 128.4, 127.3, 122.4, 116.8, 112.8, 112.0, 110.0, 77.5, 55.6, 55.4, 27.6, 15.9. HRMS (ES TOF) *m*/*z*: (M-H)- Calcd for C_18_H_17_N_2_O_5_ 341.1143; Found 341.1143.

3-(5-Ethyl-2-hydroxyphenyl)-1-methyl-3-(nitromethyl)indolin-2-one (**7eb**). This compound was prepared *via* **Method A** employing 3-hydroxy-1-methyl-3-(nitromethyl)indolin-2-one (**9e**) (111 mg, 0.50 mmol) and 4-ethylphenol (**2b**) (61 mg, 0.50 mmol) in a yield of 143 mg (0.44 mmol, 88%). Purification was performed by column chromatography (hexane/EtOAc, 3:1, *v*/*v*). The title compound was obtained as white solid, m.p. 146–148 °C, *R_f_* 0.59 (hexane/EtOAc, 2:1, *v*/*v*). ^1^H NMR (400 MHz, DMSO-*d*_6_) δ 9.82 (s, 1H), 7.50 (d, *J* = 7.2 Hz, 1H), 7.31–7.24 (m, 1H), 7.03 (d, *J* = 7.8 Hz, 1H), 7.01–6.96 (m, 2H), 6.92 (d, *J* = 1.8 Hz, 1H), 6.73 (d, *J* = 8.2 Hz, 1H), 5.97 (d, *J* = 13.1 Hz, 1H), 5.41 (d, *J* = 13.1 Hz, 1H), 3.23 (s, 3H), 2.46 (q, *J* = 7.6 Hz, 2H), 1.11 (t, *J* = 7.6 Hz, 3H). ^13^C {^1^H} NMR (101 MHz, DMSO-*d*_6_) δ 175.9, 153.3, 144.2, 134.3, 129.3, 128.8, 128.3, 127.0, 123.9, 122.6, 122.3, 116.4, 108.5, 78.1, 54.1, 27.5, 26.6, 15.9. HRMS (ES TOF) *m*/*z*: (M + Na)^+^ Calcd for C_18_H_18_N_2_NaO_4_ 349.1159; Found 349.1163.

1-Benzyl-3-(5-ethyl-2-hydroxyphenyl)-3-(nitromethyl)indolin-2-one (**7fb**). This compound was prepared *via* **Method A** employing 1-benzyl-3-hydroxy-3-(nitromethyl)indolin-2-one (**9f**) (149 mg, 0.50 mmol) and 4-ethylphenol (**2b**) (61 mg, 0.50 mmol) in a yield of 151 mg (0.38 mmol, 75%). Purification was performed by column chromatography (hexane/EtOAc, 3:1, *v*/*v*). The title compound was obtained as white solid, m.p. 198–200 °C, *R_f_* 0.64 (hexane/EtOAc, 2:1, *v*/*v*). ^1^H NMR (400 MHz, DMSO-*d*_6_) δ 9.97 (s, 1H), 7.60 (d, *J* = 7.3 Hz, 1H), 7.47–7.41 (m, 2H), 7.35 (t, *J* = 7.3 Hz, 2H), 7.31–7.26 (m, 1H), 7.19 (t, *J* = 7.4 Hz, 1H), 7.01–6.93 (m, 2H), 6.92–6.86 (m, 2H), 6.77 (d, *J* = 8.2 Hz, 1H), 6.04 (d, *J* = 13.3 Hz, 1H), 5.46 (d, *J* = 13.3 Hz, 1H), 5.07 (d, *J* = 15.8 Hz, 1H), 4.96 (d, *J* = 15.8 Hz, 1H), 2.42 (q, *J* = 7.5 Hz, 2H), 1.07 (t, *J* = 7.6 Hz, 3H). ^13^C {^1^H} NMR (101 MHz, DMSO-*d*_6_) δ 176.0, 153.4, 143.1, 136.4, 134.3, 129.4, 128.6, 128.6 (2C), 128.5, 127.5 (3C), 127.0, 124.1, 122.4, 122.4, 116.5, 109.2, 77.7, 54.4, 43.5, 27.5, 15.8. HRMS (ES TOF) *m*/*z*: (M + Na)^+^ Calcd for C_24_H_22_N_2_NaO_4_ 425.1472; Found 425.1459.

1-Butyl-3-(5-ethyl-2-hydroxyphenyl)-3-(nitromethyl)indolin-2-one (**7gb**). This compound was prepared *via* **Method A** employing 1-butyl-3-hydroxy-3-(nitromethyl)indolin-2-one (**9g**) (132 mg, 0.50 mmol) and 4-ethylphenol (**2b**) (61 mg, 0.50 mmol) in a yield of 149 mg (0.40 mmol, 81%). Purification was performed by column chromatography (hexane/EtOAc, 3:1, *v*/*v*). The title compound was obtained as white solid, m.p. 170–172 °C, *R_f_* 0.66 (hexane/EtOAc, 2:1, *v*/*v*). ^1^H NMR (400 MHz, CDCl_3_) δ 10.43 (s, 1H), 7.45 (td, *J* = 7.8, 1.1 Hz, 1H), 7.37 (d, *J* = 7.3 Hz, 1H), 7.27 (t, *J* = 7.5 Hz, 1H), 7.09 (dd, *J* = 8.2, 2.0 Hz, 1H), 7.01 (t, *J* = 8.1 Hz, 2H), 6.65 (d, *J* = 1.9 Hz, 1H), 6.11 (d, *J* = 14.5 Hz, 1H), 4.91 (d, *J* = 14.5 Hz, 1H), 3.91–3.82 (m, 1H), 3.70–3.61 (m, 1H), 2.43 (q, *J* = 7.5 Hz, 2H), 1.78–1.66 (m, 2H), 1.49–1.37 (m, 2H), 1.08 (t, *J* = 7.6 Hz, 3H), 0.96 (t, *J* = 7.3 Hz, 3H). ^13^C {^1^H} NMR (101 MHz, CDCl_3_) δ 178.9, 154.9, 143.4, 137.0, 130.23, 130.19, 127.6, 125.9, 125.8, 123.5, 121.1, 119.6, 110.4, 76.3, 56.2, 41.0, 29.0, 28.1, 20.3, 15.7, 13.8. HRMS (ES TOF) *m*/*z*: (M + Na)^+^ Calcd for C_21_H_24_N_2_NaO_4_ 391.1628; Found 391.1634.

1-Allyl-3-(5-ethyl-2-hydroxyphenyl)-3-(nitromethyl)indolin-2-one (**7hb**). This compound was prepared *via* **Method A** employing 1-allyl-3-hydroxy-3-(nitromethyl)indolin-2-one (**9h**) (124 mg, 0.50 mmol) and 4-ethylphenol (**2b**) (61 mg, 0.50 mmol) in a yield of 125 mg (0.36 mmol, 71%). Purification was performed by column chromatography (hexane/EtOAc, 3:1, *v*/*v*). The title compound was obtained as white solid, m.p. 152–154 °C, *R_f_* 0.64 (hexane/EtOAc, 2:1, *v*/*v*). ^1^H NMR (400 MHz, CDCl_3_) δ 10.27 (s, 1H), 7.43 (td, *J* = 7.8, 1.1 Hz, 1H), 7.37 (d, *J* = 7.3 Hz, 1H), 7.30–7.26 (m, 1H), 7.10 (dd, *J* = 8.2, 2.0 Hz, 1H), 7.01 (d, *J* = 8.1 Hz, 2H), 6.65 (d, *J* = 2.0 Hz, 1H), 6.14 (d, *J* = 14.5 Hz, 1H), 5.95–5.78 (m, 1H), 5.31 (d, *J* = 17.9 Hz, 1H), 5.27 (d, *J* = 10.5 Hz, 1H), 4.93 (d, *J* = 14.5 Hz, 1H), 4.55–4.49 (m, 1H), 4.35–4.28 (m, 1H), 2.43 (q, *J* = 7.5 Hz, 2H), 1.08 (t, *J* = 7.6 Hz, 3H). ^13^C {^1^H} NMR (101 MHz, CDCl_3_) δ 178.8, 154.9, 143.1, 137.2, 130.3 (2C), 130.2, 127.6, 125.7, 125.7, 123.9, 121.2, 119.6, 118.6, 111.1, 76.3, 56.3, 43.4, 28.1, 15.74. HRMS (ES TOF) *m*/*z*: (M + Na)^+^ Calcd for C_20_H_20_N_2_NaO_4_ 375.1315; Found 375.1321.

3-(4-Methoxyphenyl)-3-(nitromethyl)indolin-2-one (**7av**). This compound was prepared *via* **Method A** employing 3-hydroxy-3-(nitromethyl)indolin-2-one (**9a**) (104 mg, 0.50 mmol) and anisole (**2v**) (54 mg, 0.50 mmol) in a yield of 76 mg (0.26 mmol, 51%). Purification was performed by column chromatography (hexane/EtOAc, 3:1, *v*/*v*). The title compound was obtained as white solid, m.p. 123–125 °C, *R_f_* 0.36 (hexane/EtOAc, 2:1, *v*/*v*). ^1^H NMR (400 MHz, DMSO-*d*_6_) δ 10.69 (s, 1H), 7.48 (d, *J* = 7.3 Hz, 1H), 7.30–7.25 (m, 1H), 7.24–7.19 (m, 2H), 7.04 (t, *J* = 7.4 Hz, 1H), 6.95–6.88 (m, 3H), 5.63 (d, *J* = 14.3 Hz, 1H), 5.51 (d, *J* = 14.3 Hz, 1H), 3.71 (s, 3H). ^13^C {^1^H} NMR (101 MHz, DMSO-*d*_6_) δ 177.3, 159.0, 142.7, 129.2, 128.9, 128.1, 128.0 (2C), 124.9, 121.9, 114.2 (2C), 110.2, 78.4, 55.2, 54.3. HRMS (ES TOF) *m*/*z*: (M + Na)^+^ Calcd for C_16_H_14_N_2_NaO_4_ 321.0846; Found 321.0845.

3-(3,4-Dimethoxyphenyl)-3-(nitromethyl)indolin-2-one (**7aw**). This compound was prepared *via* **Method A** employing 3-hydroxy-3-(nitromethyl)indolin-2-one (**9a**) (104 mg, 0.50 mmol) and 1,2-dimethoxybenzene (**2w**) (69 mg, 0.50 mmol) in a yield of 128 mg (0.39 mmol, 78%). Purification was performed by column chromatography (hexane/EtOAc, 3:1, *v*/*v*). The title compound was obtained as white solid, m.p. 178–180 °C, *R_f_* 0.25 (hexane/EtOAc, 2:1, *v*/*v*). ^1^H NMR (400 MHz, DMSO-*d*_6_) δ 10.72 (s, 1H), 7.54 (d, *J* = 7.3 Hz, 1H), 7.31–7.25 (m, 1H), 7.08 (d, *J* = 2.2 Hz, 1H), 7.05 (t, *J* = 7.7 Hz, 1H), 6.95 (d, *J* = 7.7 Hz, 1H), 6.88 (d, *J* = 8.6 Hz, 1H), 6.71 (dd, *J* = 8.5, 2.2 Hz, 1H), 5.70 (d, *J* = 14.4 Hz, 1H), 5.55 (d, *J* = 14.4 Hz, 1H), 3.73 (s, 3H), 3.69 (s, 3H). ^13^C {^1^H} NMR (101 MHz, DMSO-*d*_6_) δ 177.3, 148.82, 148.79, 142.7, 129.2, 128.9, 128.3, 124.9, 121.8, 119.2, 111.7, 110.8, 110.2, 78.5, 55.7, 55.6, 54.6. HRMS (ES TOF) *m*/*z*: (M + Na)^+^ Calcd for C_17_H_16_N_2_NaO_5_ 351.0951; Found 351.0957.

3-(2,4-Dimethoxyphenyl)-3-(nitromethyl)indolin-2-one (**7ax**). This compound was prepared *via* **Method A** employing 3-hydroxy-3-(nitromethyl)indolin-2-one (**9a**) (104 mg, 0.50 mmol) and 1,3-dimethoxybenzene (**2x**) (69 mg, 0.50 mmol) in a yield of 120 mg (0.37 mmol, 73%). Purification was performed by column chromatography (hexane/EtOAc, 3:1, *v*/*v*). The title compound was obtained as white solid, m.p. 169–171 °C, *R_f_* 0.27 (hexane/EtOAc, 2:1, *v*/*v*). ^1^H NMR (400 MHz, DMSO-*d*_6_) δ 10.76 (s, 1H), 7.29 (d, *J* = 7.4 Hz, 1H), 7.20–7.15 (m, 1H), 7.10 (d, *J* = 8.7 Hz, 1H), 6.89 (t, *J* = 7.3 Hz, 1H), 6.85 (d, *J* = 7.7 Hz, 1H), 6.57 (d, *J* = 2.5 Hz, 1H), 6.51 (dd, *J* = 8.7, 2.5 Hz, 1H), 5.75 (d, *J* = 13.1 Hz, 1H), 5.31 (d, *J* = 13.1 Hz, 1H), 3.73 (s, 3H), 3.66 (s, 3H). ^13^C {^1^H} NMR (101 MHz, DMSO-*d*_6_) δ 177.5, 160.4, 158.5, 142.7, 130.2, 128.6, 128.3, 124.0, 121.6, 117.7, 109.5, 105.1, 100.0, 78.3, 55.7, 55.3, 54.0. HRMS (ES TOF) *m*/*z*: (M + Na)^+^ Calcd for C_17_H_16_N_2_NaO_5_ 351.0951; Found 351.0955.

3-(2,3-Dihydrobenzo[*b*][1,4]dioxin-6-yl)-3-(nitromethyl)indolin-2-one (**7ay**). This compound was prepared *via* **Method A** employing 3-hydroxy-3-(nitromethyl)indolin-2-one (**9a**) (104 mg, 0.50 mmol) and 2,3-dihydrobenzo[*b*][1,4]dioxine (**2y**) (68 mg, 0.50 mmol) in a yield of 145 mg (0.45 mmol, 89%). Purification was performed by column chromatography (hexane/EtOAc, 3:1, *v*/*v*). The title compound was obtained as white solid, m.p. 145–147 °C, *R_f_* 0.3 (hexane/EtOAc, 2:1, *v*/*v*). ^1^H NMR (400 MHz, DMSO-*d*_6_) δ 10.72 (s, 1H), 7.49 (d, *J* = 7.3 Hz, 1H), 7.31–7.24 (m, 1H), 7.04 (t, *J* = 7.3 Hz, 1H), 6.94 (d, *J* = 7.7 Hz, 1H), 6.86–6.80 (m, 2H), 6.74 (dd, *J* = 8.6, 2.3 Hz, 1H), 5.63 (d, *J* = 14.3 Hz, 1H), 5.51 (d, *J* = 14.3 Hz, 1H), 4.19 (s, 4H). ^13^C {^1^H} NMR (101 MHz, DMSO-*d*_6_) δ 177.2, 143.37, 143.36, 142.7, 129.3, 129.1, 128.8, 124.9, 121.91, 119.6, 117.3, 115.8, 110.2, 78.3, 64.2 (2C), 54.3. HRMS (ES TOF) *m*/*z*: (M + Na)^+^ Calcd for C_17_H_14_N_2_NaO_5_ 349.0795; Found 349.0797.

3-(5-Bromo-2-methoxyphenyl)-3-(nitromethyl)indolin-2-one (**7az**). This compound was prepared *via* **Method A** employing 3-hydroxy-3-(nitromethyl)indolin-2-one (**9a**) (104 mg, 0.50 mmol) and 4-bromoanizole (**2z**) (93 mg, 0.50 mmol) in a yield of 124 mg (0.33 mmol, 66%). Purification was performed by column chromatography (hexane/EtOAc, 3:1, *v*/*v*). The title compound was obtained as white solid, m.p. 93–94 °C, *R_f_* 0.4 (hexane/EtOAc, 2:1, *v*/*v*). ^1^H NMR (400 MHz, DMSO-*d*_6_) δ 10.85 (s, 1H), 7.49 (d, *J* = 8.7 Hz, 1H), 7.39 (s, 1H), 7.28 (d, *J* = 7.2 Hz, 1H), 7.20 (t, *J* = 7.4 Hz, 1H), 6.98 (d, *J* = 8.6 Hz, 1H), 6.94–6.87 (m, 2H), 5.77 (d, *J* = 13.0 Hz, 1H), 5.42 (d, *J* = 12.9 Hz, 1H), 3.62 (s, 3H). ^13^C NMR (101 MHz, DMSO-*d*_6_) δ 177.1, 157.0, 143.2, 132.4, 130.7, 129.6, 129.4, 128.3, 124.4, 122.2, 115.4, 112.7, 110.1, 78.4, 56.4, 54.4. HRMS (ES TOF) *m*/*z*: (M + Na)^+^ Calcd for C_16_H_13_BrN_2_NaO_4_ 398.9951; Found 398.9958.

### 3.3. General Procedure for the Synthesis of Chromeno[3,4-b]indol-6(7H)-One 8 (Method B)

3-Hydroxy-3-(nitromethyl)indolin-2-one **9a** (104 mg, 0.50 mmol) and *p*-cresol **2a** (54 mg, 0.50 mmol) were placed into a 5 mL Erlenmeyer flask equipped with a magnetic stirrer, and 1 mL of MsOH was added. The resulting solution was stirred at 80 °C for 24 h. After the reaction was complete (monitoring by TLC), the reaction mixture was poured into 50 mL of cold water and extracted with EtOAc (3 × 20 mL). The organic phase was evaporated under reduced pressure and the crude material was purified by column chromatography using hexane/EtOAc mixture as eluent.

2-Methylchromeno[3,4-*b*]indol-6(7*H*)-one (**8aa**). This compound was prepared *via* **Method B** employing 3-hydroxy-3-(nitromethyl)indolin-2-one (**9a**) (104 mg, 0.50 mmol) and *p*-cresol (**2a**) (54 mg, 0.50 mmol) in a yield of 55 mg (0.22 mmol, 44%). Purification was performed by column chromatography (hexane/EtOAc, 3:1, *v*/*v*). The title compound was obtained as white solid, m.p. 241–243 °C, *R_f_* 0.29 (hexane/EtOAc, 2:1, *v*/*v*). ^1^H NMR (400 MHz, DMSO-*d*_6_) δ 12.66 (s, 1H), 8.50 (d, *J* = 8.2 Hz, 1H), 8.21 (s, 1H), 7.63 (d, *J* = 8.4 Hz, 1H), 7.58–7.49 (m, 1H), 7.41 (d, *J* = 8.4 Hz, 1H), 7.38–7.33 (m, 1H), 7.27 (dd, *J* = 8.5, 1.7 Hz, 1H), 2.49 (s, 3H). ^13^C {^1^H} NMR (101 MHz, DMSO-*d*_6_) δ 155.4, 148.5, 139.7, 134.3, 128.2, 127.2, 123.4, 122.8, 121.5, 121.4, 121.3, 119.9, 118.1, 116.6, 113.4, 20.6. HRMS (ES TOF) *m*/*z* (M + Na)^+^ Calcd for C_16_H_11_NNaO_2_ 272.0682; Found 272.0682.

2-Ethylchromeno[3,4-*b*]indol-6(7*H*)-one (**8ab**). This compound was prepared *via* **Method B** employing 3-hydroxy-3-(nitromethyl)indolin-2-one (**9a**) (104 mg, 0.50 mmol) and 4-ethylphenol (**2b**) (61 mg, 0.50 mmol) in a yield of 53 mg (0.20 mmol, 40%). Purification was performed by column chromatography (hexane/EtOAc, 3:1, *v*/*v*). The title compound was obtained as white solid, m.p. 225–227 °C, *R_f_* 0.31 (hexane/EtOAc, 2:1, *v*/*v*). ^1^H NMR (400 MHz, DMSO-*d*_6_) δ 12.68 (s, 1H), 8.50 (d, *J* = 8.2 Hz, 1H), 8.20 (d, *J* = 1.6 Hz, 1H), 7.63 (d, *J* = 8.3 Hz, 1H), 7.53 (t, *J* = 7.4 Hz, 1H), 7.43 (d, *J* = 8.4 Hz, 1H), 7.35 (t, *J* = 7.7 Hz, 1H), 7.31 (dd, *J* = 8.5, 1.9 Hz, 1H), 2.79 (q, *J* = 7.5 Hz, 2H), 1.28 (t, *J* = 7.6 Hz, 3H). ^13^C {^1^H} NMR (101 MHz, DMSO-*d*_6_) δ 155.5, 148.7, 140.7, 139.8, 127.2, 127.1, 122.9, 122.3, 121.6, 121.5, 121.3, 120.0, 118.2, 116.8, 113.4, 27.7, 16.1. HRMS (ES TOF) *m*/*z* (M + Na)^+^ Calcd for C_17_H_13_NNaO_2_ 286.0838; Found 286.0838.

2-Fluorochromeno[3,4-*b*]indol-6(7*H*)-one (**8ai**). This compound was prepared *via* **Method B** employing 3-hydroxy-3-(nitromethyl)indolin-2-one (**9a**) (104 mg, 0.50 mmol) and 4-fluorophenol (**2i**) (56 mg, 0.50 mmol) in a yield of 52 mg (0.21 mmol, 41%). Purification was performed by column chromatography (hexane/EtOAc, 3:1, *v*/*v*). The title compound was obtained as white solid, m.p. 283–285 °C, *R_f_* 0.45 (hexane/EtOAc, 2:1, *v*/*v*). ^1^H NMR (400 MHz, DMSO-*d*_6_) δ 12.85 (s, 1H), 8.53 (d, *J* = 8.2 Hz, 1H), 8.22 (dd, *J* = 9.2, 2.9 Hz, 1H), 7.65 (d, *J* = 8.3 Hz, 1H), 7.62–7.58 (m, 1H), 7.55 (t, *J* = 7.5 Hz, 1H), 7.39–7.31 (m, 2H). ^13^C {^1^H} NMR (101 MHz, DMSO-*d*_6_) δ 158.9 (d, *J* = 240.3 Hz), 155.1, 146.7, 139.7, 127.4, 122.7, 121.9, 121.7, 121.4, 119.5 (d, *J* = 10.0 Hz), 119.2 (d, *J* = 2.9 Hz), 118.7 (d, *J* = 9.3 Hz), 114.4 (d, *J* = 24.5 Hz), 113.5, 109.4 (d, *J* = 25.1 Hz). ^19^F NMR (376 MHz, DMSO-*d*_6_) δ -117.11. HRMS (ES TOF) *m*/*z* (M + Na)^+^ Calcd for C_15_H_8_FNNaO_2_ 276.0431; Found 276.0428.

Benzo [5,6]chromeno[3,4-*b*]indol-8(9*H*)-one (**8az**). This compound was prepared *via* **Method B** employing 3-hydroxy-3-(nitromethyl)indolin-2-one (**9a**) (104 mg, 0.50 mmol) and *β*-naphthol (**2′z**) (80 mg, 0.50 mmol) in a yield of 80 mg (0.28 mmol, 56%). Purification was performed by column chromatography (hexane/EtOAc, 3:1, *v*/*v*). The title compound was obtained as white solid, m.p. 298–300 °C, *R_f_* 0.28 (hexane/EtOAc, 2:1, *v*/*v*). ^1^H NMR (400 MHz, DMSO-*d*_6_) δ 12.69 (s, 1H), 8.89 (d, *J* = 8.5 Hz, 1H), 8.43 (d, *J* = 8.4 Hz, 1H), 8.11 (d, *J* = 7.9 Hz, 1H), 8.06 (d, *J* = 8.9 Hz, 1H), 7.80 (t, *J* = 7.6 Hz, 1H), 7.76–7.62 (m, 3H), 7.57 (t, *J* = 7.6 Hz, 1H), 7.37 (t, *J* = 7.6 Hz, 1H). ^13^C {^1^H} NMR (101 MHz, DMSO) δ 155.4, 148.8, 140.6, 130.8, 128.7, 128.6, 128.2, 127.2, 126.4, 126.2, 125.8, 124.4, 122.8, 121.8, 121.1, 119.8, 117.4, 113.8, 113.7. HRMS (ES TOF) *m*/*z* (M + Na)^+^ Calcd for C_19_H_11_NNaO_2_ 308.0682; Found 308.0691.

2-Ethyl-10-methylchromeno[3,4-*b*]indol-6(7*H*)-one (**8bb**). This compound was prepared *via* **Method B** employing 3-hydroxy-5-methyl-3-(nitromethyl)indolin-2-one (**9b**) (111 mg, 0.50 mmol) and 4-ethylphenol (**2b**) (61 mg, 0.50 mmol) in a yield of 65 mg (0.24 mmol, 47%). Purification was performed by column chromatography (hexane/EtOAc, 3:1, *v*/*v*). The title compound was obtained as white solid, m.p. 251–253 °C, *R_f_* 0.53 (hexane/EtOAc, 2:1, *v*/*v*). ^1^H NMR (400 MHz, DMSO-*d*_6_) δ 12.55 (s, 1H), 8.25 (s, 1H), 8.17 (d, *J* = 1.6 Hz, 1H), 7.51 (d, *J* = 8.5 Hz, 1H), 7.41 (d, *J* = 8.4 Hz, 1H), 7.37–7.32 (m, 1H), 7.29 (dd, *J* = 8.5, 1.8 Hz, 1H), 2.80 (q, *J* = 7.5 Hz, 2H), 2.52 (s, 3H), 1.28 (t, *J* = 7.6 Hz, 3H). ^13^C {^1^H} NMR (101 MHz, DMSO-*d*_6_) δ 155.5, 148.6, 140.6, 138.2, 130.6, 129.1, 126.8, 122.4, 121.9, 121.7, 121.3, 119.4, 118.4, 116.7, 113.1, 27.8, 21.2, 16.2. HRMS (ES TOF) *m*/*z* (M-H)^−^ Calcd for C_18_H_14_NO_2_ 276.1030; Found 276.1030.

2-Isopropyl-10-methylchromeno[3,4-*b*]indol-6(7*H*)-one (**8bd**). This compound was prepared *via* **Method B** employing 3-hydroxy-5-methyl-3-(nitromethyl)indolin-2-one (**9b**) (111 mg, 0.50 mmol) and 4-isopropylphenol (**2d**) (68 mg, 0.50 mmol) in a yield of 70 mg (0.24 mmol, 48%). Purification was performed by column chromatography (hexane/EtOAc, 3:1, *v*/*v*). The title compound was obtained as white solid, m.p. 262–264 °C, *R_f_* 0.51 (hexane/EtOAc, 2:1, *v*/*v*). ^1^H NMR (400 MHz, DMSO-*d*_6_) δ 12.58 (s, 1H), 8.28 (s, 1H), 8.20 (s, 1H), 7.53 (d, *J* = 8.3 Hz, 1H), 7.46 (d, *J* = 8.6 Hz, 1H), 7.42–7.33 (m, 2H), 3.22–3.15 (m, 1H), 2.54 (s, 3H), 1.32 (d, *J* = 6.6 Hz, 6H). ^13^C NMR (101 MHz, DMSO-*d*_6_) δ 155.5, 148.6, ′145.3, 138.2, 130.7, 129.1, 125.1, 121.9, 121.7, 121.3, 121.1, 119.5, 118.3, 116.9, 113.1, 33.1, 24.2 (2C), 21.3. HRMS (ES TOF) *m*/*z* (M + Na)^+^ Calcd for C_19_H_17_NNaO_2_ 314.1151; Found 314.1155.

10-Bromo-2-ethylchromeno[3,4-*b*]indol-6(7*H*)-one (**8cb**). This compound was prepared *via* **Method B** employing 5-bromo-3-hydroxy-3-(nitromethyl)indolin-2-one (**9c**) (143 mg, 0.50 mmol) and 4-ethylphenol (**2b**) (61 mg, 0.50 mmol) in a yield of 68 mg (0.20 mmol, 40%). Purification was performed by column chromatography (hexane/EtOAc, 3:1, *v*/*v*). The title compound was obtained as white solid, m.p. 211–214 °C, *R_f_* 0.48 (hexane/EtOAc, 2:1, *v*/*v*). ^1^H NMR (400 MHz, DMSO-*d*_6_) δ 12.87 (s, 1H), 8.72 (s, 1H), 8.21 (s, 1H), 7.63 (d, *J* = 8.7 Hz, 1H), 7.57 (d, *J* = 8.8 Hz, 1H), 7.42 (d, *J* = 8.4 Hz, 1H), 7.32 (d, *J* = 8.2 Hz, 1H), 2.81 (q, *J* = 7.2 Hz, 2H), 1.27 (t, *J* = 7.5 Hz, 3H). ^13^C NMR (101 MHz, DMSO-*d*_6_) δ 155.2, 148.6, 141.0, 138.3, 129.9, 127.3, 124.8, 122.9, 122.6, 122.3, 119.3, 117.6, 116.8, 115.3, 114.1, 27.6, 16.4. HRMS (ES TOF) *m*/*z* (M + Na)^+^ Calcd for C_17_H_12_BrNNaO_2_ 363.9944; Found 363.9946.

### 3.4. General Procedure for the Synthesis of (Z)-2-(Hydroxyimino)-2H-spiro[benzofuran-3,3′-indolin]-2′-One 13 (Method C)

3-(2-Hydroxyphenyl)-3-(nitromethyl)indolin-2-one **7aa** (75 mg, 0.25 mmol), 2 mL of EtOH and 0.5 mL of water were placed into a 5 mL round-bottom flask equipped with a magnetic stirrer and condenser, then 42 mg of KOH (0.75 mmol, 3 equiv) was added in one portion. The resulting solution was refluxed for 2 h. After the reaction was complete (monitoring by TLC), the reaction mixture was poured into 50 mL of cold water and extracted with EtOAc (3 × 20 mL). The organic phase was evaporated under reduced pressure and the crude material was purified by column chromatography using hexane/EtOAc mixture as eluent.

(*Z*)-2-(Hydroxyimino)-5-methyl-2*H*-spiro[benzofuran-3,3′-indolin]-2′-one (**13aa**). This compound was prepared *via* **Method C** employing 3-(2-hydroxy-5-methylphenyl)-3-(nitromethyl)indolin-2-one **7aa** (75 mg, 0.25 mmol) in a yield of 64 mg (0.23 mmol, 92%). Purification was performed by column chromatography (hexane/EtOAc, 3:1, *v*/*v*). The title compound was obtained as white solid, m.p. 253–255 °C, *R_f_* 0.27 (hexane/EtOAc, 1:1, *v*/*v*). ^1^H NMR (400 MHz, DMSO-*d*_6_) δ 10.86 (s, 1H), 10.45 (s, 1H), 7.32–7.27 (m, 1H), 7.19 (s, 2H), 6.99–6.96 (m, 3H), 6.76 (s, 1H), 2.20 (s, 3H). ^13^C {^1^H} NMR (101 MHz, DMSO-*d*_6_) δ 174.7, 155.4, 154.5, 142.5, 133.5, 131.0, 130.6, 129.5, 127.2, 124.3, 123.9, 122.7, 110.3, 110.2, 58.5, 20.3. HRMS (ES TOF) *m*/*z* (M + Na)^+^ Calcd for C_16_H_12_N_2_NaO_3_ 303.0740; Found 303.0733.

(*Z*)-5-Ethyl-2-(hydroxyimino)-2*H*-spiro[benzofuran-3,3′-indolin]-2′-one (**13ab**). This compound was prepared *via* **Method C** employing 3-(5-Ethyl-2-hydroxyphenyl)-3-(nitromethyl)indolin-2-one **7ab** (78 mg, 0.25 mmol) in a yield of 71 mg (0.24 mmol, 96%). Purification was performed by column chromatography (hexane/EtOAc, 3:1, *v*/*v*). The title compound was obtained as white solid, m.p. 242–244 °C, *R_f_* 0.27 (hexane/EtOAc, 1:1, *v*/*v*). ^1^H NMR (400 MHz, DMSO-*d*_6_) δ 10.86 (s, 1H), 10.45 (s, 1H), 7.30 (td, *J* = 7.3, 2.3 Hz, 1H), 7.26–7.20 (m, 2H), 7.01–6.95 (m, 3H), 6.78 (s, 1H), 2.55–2.51 (m, 2H), 1.08 (t, *J* = 7.6 Hz, 3H). ^13^C NMR (101 MHz, DMSO-*d*_6_) δ 174.7, 155.4, 154.6, 142.5, 139.9, 131.0, 129.50, 129.48, 127.1, 124.3, 122.8, 122.7, 110.4, 110.2, 58.5, 27.5, 15.8. HRMS (ES TOF) *m*/*z* (M + Na)^+^ Calcd for C_17_H_14_N_2_NaO_3_ 317.0897; Found 317.0904.

(*Z*)-2-(Hydroxyimino)-5,6-dimethyl-2*H*-spiro[benzofuran-3,3′-indolin]-2′-one (**13ac**). This compound was prepared *via* **Method C** employing 3-(2-hydroxy-4,5-dimethylphenyl)-3-(nitromethyl)indolin-2-one **7ac** (78 mg, 0.25 mmol) in a yield of 66 mg (0.225 mmol, 90%). Purification was performed by column chromatography (hexane/EtOAc, 3:1, *v*/*v*). The title compound was obtained as white solid, m.p. 255–257 °C, *R_f_* 0.28(hexane/EtOAc, 1:1, *v*/*v*). ^1^H NMR (400 MHz, DMSO-*d*_6_) δ 10.82 (s, 1H), 10.41 (s, 1H), 7.30–7.25 (m, 1H), 7.11 (s, 1H), 6.99–6.94 (m, 3H), 6.72 (s, 1H), 2.24 (s, 3H), 2.10 (s, 3H). ^13^C {^1^H} NMR (101 MHz, DMSO-*d*_6_) δ 174.8, 155.6, 154.8, 142.5, 138.9, 132.2, 131.2, 129.4, 124.3, 124.3, 124.0, 122.7, 111.3, 110.2, 58.3, 19.9, 18.8. HRMS (ES TOF) *m*/*z* (M + Na)^+^ Calcd for C_17_H_14_N_2_NaO_3_ 317.0897; Found 317.0905

(*Z*)-2-(Hydroxyimino)-5-isopropyl-2*H*-spiro[benzofuran-3,3′-indolin]-2′-one (**13ad**). This compound was prepared *via* **Method C** employing 3-(2-hydroxy-5-isopropylphenyl)-3-(nitromethyl)indolin-2-one **7ad** (82 mg, 0.25 mmol) in a yield of 73 mg (0.237 mmol, 95%). Purification was performed by column chromatography (hexane/EtOAc, 3:1, *v*/*v*). The title compound was obtained as white solid, m.p. 248–250 °C, *R_f_* 0.31 (hexane/EtOAc, 1:1, *v*/*v*). ^1^H NMR (400 MHz, DMSO-*d*_6_) δ 10.86 (s, 1H), 10.46 (s, 1H), 7.32–7.26 (m, 2H), 7.22 (d, *J* = 8.4 Hz, 1H), 7.01–6.96 (m, 3H), 6.81–6.78 (m, 1H), 2.86–2.79 (m, 1H), 1.10 (d, *J* = 3.4 Hz, 3H), 1.09 (d, *J* = 3.4 Hz, 3H). ^13^C {^1^H} NMR (101 MHz, DMSO-*d*_6_) δ 174.7, 155.5, 154.8, 144.7, 142.6, 131.1, 129.5, 128.1, 127.1, 124.3, 122.8, 121.4, 110.4, 110.3, 58.6, 33.0, 24.10, 24.05. HRMS (ES TOF) *m*/*z* (M + Na)^+^ Calcd for C_18_H_16_N_2_NaO_3_ 331.1053; Found 331.1058.

(*Z*)-5-chloro-2-(hydroxyimino)-6-methyl-2*H*-spiro[benzofuran-3,3′-indolin]-2′-one (**13ae**). This compound was prepared *via* **Method C** employing 3-(5-chloro-2-hydroxy-4-methylphenyl)-3-(nitromethyl)indolin-2-one **7ae** (83 mg, 0.25 mmol) in a yield of 68 mg (0.217 mmol, 87%). Purification was performed by column chromatography (hexane/EtOAc, 3:1, *v*/*v*). The title compound was obtained as white solid, m.p. 248–250 °C, *R_f_* 0.33 (hexane/EtOAc, 1:1, *v*/*v*). ^1^H NMR (400 MHz, DMSO-*d*_6_) δ 10.89 (s, 1H), 10.58 (s, 1H), 7.38 (s, 1H), 7.30 (t, *J* = 7.1 Hz, 1H), 7.09 (s, 1H), 7.04–6.94 (m, 3H), 2.36 (s, 3H). ^13^C {^1^H} NMR (101 MHz, DMSO-*d*_6_) δ 174.2, 155.3, 154.9, 142.6, 138.2, 130.5, 129.7, 128.1, 126.4, 124.3, 124.0, 122.7, 113.1, 110.4, 58.2, 20.2. HRMS (ES TOF) *m*/*z* (M + Na)^+^ Calcd for C_16_H_11_ClN_2_NaO_3_ 337.0350; Found 337.0350.

2-(2-(Hydroxyimino)-2′-oxo-2*H*-spiro[benzofuran-3,3′-indolin]-5-yl)acetic acid (**13ah**) **(Mixture of Z/E isomers in 5:3 ratio).** This compound was prepared *via* **Method C** employing 2-(4-hydroxy-3-(3-(nitromethyl)-2-oxoindolin-3-yl)phenyl)acetic acid **7ah** (86 mg, 0.25 mmol) in a yield of 66 mg (0.204 mmol, 81%). Purification was performed by column chromatography (hexane/EtOAc/HCOOH, 75:25:1, *v*/*v*). The title compound was obtained as yellow oil, *R_f_* 0.3 (hexane/EtOAc/HCOOH, 50:50:1, *v*/*v*/*v*). ^1^H NMR Z (major)/E (minor) mixture (400 MHz, DMSO-*d*_6_) δ 12.23 (br. s, 1H + 0.6H), 10.94 (s, 0.6H, minor isomer), 10.88 (s, 1H, major isomer), 10.50 (s, 1H, major isomer), 10.15 (s, 0.6H, minor isomer), 7.32–7.20 (m, 4.8H, mixture major + minor), 7.02–6.91 (m, 4.8H, mixture major + minor), 6.85 (s, 1H, major isomer), 6.79 (s, 0.6H, minor isomer), 3.51 (s, 2H, major isomer), 3.49 (s, 1.2H, minor isomer). ^13^C NMR Z (major)/E (minor) mixture (101 MHz, DMSO-*d*_6_) δ 174.6 (major), 173.0 (minor), 172.70 (major), 172.68 (2C, major + minor), 172.1 (minor), 163.2 (minor), 155.3 (major), 155.2 (major), 154.9 (minor), 142.7 (minor), 142.5 (major), 131.5 (major), 131.2 (minor), 131.2 (major), 130.9 (major), 130.7 (minor), 129.6 (major), 129.1 (minor), 129.0 (minor), 127.7 (minor), 127.1 (major), 124.5 (major), 124.4 (major), 124.2 (minor), 123.4 (minor), 122.8 (major), 122.3 (minor), 110.4 (major), 110.3 (major), 110.2 (minor), 110.0 (minor), 58.4 (major), 58.2 (minor). HRMS (ES TOF) *m*/*z* (M + Na)^+^ Calcd for C_17_H_12_N_2_NaO_5_ 347.0638; Found 347.0646.

(*Z*)-5-Fluoro-2-(hydroxyimino)-2*H*-spiro[benzofuran-3,3′-indolin]-2′-one (**13ai**). This compound was prepared *via* **Method C** employing 3-(5-fluoro-2-hydroxyphenyl)-3-(nitromethyl)indolin-2-one **7ai** (76 mg, 0.25 mmol) in a yield of 59 mg (0.208 mmol, 83%). Purification was performed by column chromatography (hexane/EtOAc, 3:1, *v*/*v*). The title compound was obtained as white solid, m.p. 292–294 °C, *R_f_* 0.5 (hexane/EtOAc, 1:1, *v*/*v*). ^1^H NMR (400 MHz, DMSO-*d*_6_) δ 10.45 (s, 1H), 9.02 (s, 1H), 7.66–7.60 (m, 1H), 7.59–7.52 (m, 2H), 7.23 (td, *J* = 7.6, 1.4 Hz, 1H), 7.20–7.15 (m, 1H), 7.11–7.04 (m, 2H). ^13^C NMR (101 MHz, DMSO-*d*_6_) δ 160.7, 159.3 (d, *J* = 236.5 Hz), 149.8, 146.9, 136.9, 127.9, 127.4 (d, *J* = 10.9 Hz), 125.9, 124.6, 122.2, 121.6, 112.0 (d, *J* = 9.8 Hz), 110.0 (d, *J* = 25.9 Hz), 104.9 (d, *J* = 26.5 Hz), 98.6 (d, *J* = 3.5 Hz). ^19^F NMR (376 MHz, DMSO-*d*_6_) δ −119.04. HRMS (ES TOF) *m*/*z* (M + Na)^+^ Calcd for C_15_H_9_FN_2_NaO_3_ 307.0489; Found 307.0491.

(*Z*)-7-Bromo-2-(hydroxyimino)-5-methyl-2*H*-spiro[benzofuran-3,3′-indolin]-2′-one (**13aj**). This compound was prepared *via* **Method C** employing 3-(3-bromo-2-hydroxy-5-methylphenyl)-3-(nitromethyl)indolin-2-one **7aj** (94 mg, 0.25 mmol) in a yield of 72 mg (0.20 mmol, 80%). Purification was performed by column chromatography (hexane/EtOAc, 3:1, *v*/*v*). The title compound was obtained as white solid, m.p. 252–254 °C, *R_f_* 0.13 (hexane/EtOAc, 1:2, *v*/*v*). ^1^H NMR (400 MHz, DMSO-*d*_6_) δ 10.92 (s, 1H), 10.69 (s, 1H), 7.45 (s, 1H), 7.31 (t, *J* = 7.7 Hz, 1H), 7.10 (d, *J* = 7.0 Hz, 1H), 7.00–6.96 (m, 2H), 6.79 (s, 1H), 2.21 (s, 3H). ^13^C NMR (101 MHz, DMSO-*d*_6_) δ 174.2, 153.8, 151.9, 142.5, 135.7, 133.2, 130.4, 129.8, 128.5, 124.6, 123.4, 122.9, 110.4, 101.9, 59.3, 19.9. HRMS (ES TOF) *m*/*z* (M + Na)^+^ Calcd for C_16_H_11_BrN_2_NaO_3_ 380.9845; Found 380.9852.

(*Z*)-2-(Hydroxyimino)-5-phenyl-2*H*-spiro[benzofuran-3,3′-indolin]-2′-one (**13au**). This compound was prepared *via* **Method C** employing 3-(4-hydroxy-[1,1′-biphenyl]-3-yl)-3-(nitromethyl)indolin-2-one **7′au** (90 mg, 0.25 mmol) in a yield of 80 mg (0.233 mmol, 93%). Purification was performed by column chromatography (hexane/EtOAc, 3:1, *v*/*v*). The title compound was obtained as white solid, m.p. 239–241 °C, *R_f_* 0.27 (hexane/EtOAc, 1:1, *v*/*v*). ^1^H NMR (400 MHz, DMSO-*d*_6_) δ 10.90 (s, 1H), 10.56 (s, 1H), 7.71 (d, *J* = 8.0 Hz, 1H), 7.54 (d, *J* = 6.9 Hz, 2H), 7.43–7.35 (m, 3H), 7.33–7.28 (m, 2H), 7.23 (s, 1H), 7.08–7.03 (m, 1H), 7.01–6.94 (m, 2H). ^13^C NMR (101 MHz, DMSO-*d*_6_) δ 174.5, 156.1, 155.2, 142.7, 139.0, 136.6, 130.8, 129.6, 128.93 (2C), 128.87, 128.1, 127.4, 126.6 (2C), 124.4, 122.7, 121.9, 111.1, 110.3, 58.5. HRMS (ES TOF) *m*/*z* (M + Na)^+^ Calcd for C_21_H_14_N_2_NaO_3_ 365.0897; Found 365.0907.

(*Z*)-5-Ethyl-2-(hydroxyimino)-5′-methyl-2*H*-spiro[benzofuran-3,3′-indolin]-2′-one (**13bb**). This compound was prepared *via* **Method C** employing 3-(5-ethyl-2-hydroxyphenyl)-5-methyl-3-(nitromethyl)indolin-2-one **7bb** (82 mg, 0.25 mmol) in a yield of 70 mg (0.227 mmol, 91%). Purification was performed by column chromatography (hexane/EtOAc, 3:1, *v*/*v*). The title compound was obtained as white solid, m.p. 272–274 °C, *R_f_* 0.59 (hexane/EtOAc, 1:1, *v*/*v*). ^1^H NMR (400 MHz, DMSO-*d*_6_) δ 10.21 (s, 1H), 8.84 (s, 1H), 7.60 (s, 1H), 7.43 (s, 1H), 7.41 (d, *J* = 8.3 Hz, 1H), 7.08 (dd, *J* = 8.4, 1.3 Hz, 1H), 7.05–7.00 (m, 1H), 6.99–6.95 (m, 1H), 2.72 (q, *J* = 7.5 Hz, 2H), 2.33 (s, 3H), 1.23 (t, *J* = 7.6 Hz, 3H). ^13^C {^1^H} NMR (101 MHz, DMSO-*d*_6_) δ 161.3, 149.1, 148.3, 139.5, 134.6, 133.5, 128.2, 126.4, 126.1, 122.8, 122.8, 121.6, 117.6, 110.6, 98.2, 28.4, 20.5, 16.6. HRMS (ES TOF) *m*/*z* (M + Na)^+^ Calcd for C_18_H_16_N_2_NaO_3_ 331.1053; Found 331.1050.

(*Z*)-5′-Bromo-5-chloro-2-(hydroxyimino)-6-methyl-2*H*-spiro[benzofuran-3,3′-indolin]-2′-one (**13ce**). This compound was prepared *via* **Method C** employing 5-bromo-3-(5-chloro-2-hydroxy-4-methylphenyl)-3-(nitromethyl)indolin-2-one **7ce** (103 mg, 0.25 mmol) in a yield of 83 mg (0.212 mmol, 85%). Purification was performed by column chromatography (hexane/EtOAc, 3:1, *v*/*v*). The title compound was obtained as white solid, m.p. 248–250 °C, *R_f_* 0.16 (hexane/EtOAc, 2:1, *v*/*v*). ^1^H NMR (400 MHz, DMSO-*d*_6_) δ 11.03 (s, 1H), 10.65 (s, 1H), 7.49 (dd, *J* = 8.3, 1.9 Hz, 1H), 7.37 (s, 1H), 7.35 (d, *J* = 1.9 Hz, 1H), 7.19 (s, 1H), 6.93 (d, *J* = 8.3 Hz, 1H), 2.36 (s, 3H). ^13^C NMR (101 MHz, DMSO-*d*_6_) δ 173.8, 155.5, 154.6, 142.1, 138.3, 132.5, 132.4, 128.1, 127.4, 125.7, 124.1, 114.2, 113.2, 112.4, 58.1, 20.2. HRMS (ES TOF) *m*/*z* (M + Na)^+^ Calcd for C_16_H_10_BrClN_2_NaO_3_ 414.9456; Found 414.9453.

5-Ethyl-2-(hydroxyimino)-5′-methoxy-2*H*-spiro[benzofuran-3,3′-indolin]-2′-one (**13db**) (Mixture of *Z*/*E* isomers in 3.6:1 ratio). This compound was prepared *via* **Method C** employing 3-(5-ethyl-2-hydroxyphenyl)-5-methoxy-3-(nitromethyl)indolin-2-one **7db** (86 mg, 0.25 mmol) in a yield of 76 mg (0.235 mmol, 94%). Purification was performed by column chromatography (hexane/EtOAc, 3:1, *v*/*v*). The title compound was obtained as white solid, m.p. 122–124 °C, *R_f_* 0.21 (hexane/EtOAc, 1:1, *v*/*v*). ^1^H NMR *Z* (major)/*E* (minor) mixture (400 MHz, DMSO-*d*_6_) δ 10.73 (s, 0.28H, minor isomer), 10.67 (s, 1H, major isomer), 10.45 (s, 1H, major isomer), 10.10 (s, 0.28H, minor isomer), 7.25–7.15 (m, 2.56H, major + minor isomers), 6.93–6.82 (m, 2.56H, major + minor isomers), 6.80–6.74 (m, 1H, major isomer), 6.71–6.68 (m, 0.28H, minor isomer), 6.62 (d, *J* = 2.2 Hz, 1H, major isomer), 6.54 (d, *J* = 2.3 Hz, 0.28H, minor isomer), 3.63 (s, 3H, major isomer), 3.62 (s, 0.84H, minor isomer), 2.55–2.50 (m, 2.56H, major + minor isomers), 1.09 (t, *J* = 7.6 Hz, 3.84H, major + minor isomers). (*Z*)- major isomer: ^13^C NMR (101 MHz, DMSO-*d*_6_) δ 174.6, 155.5, 155.4, 154.7, 139.9, 135.8, 132.0, 129.5, 127.2, 122.7, 114.6, 110.8 (2C), 110.4, 59.0, 55.6, 27.53, 15.8. (*E*)- minor isomer: ^13^C NMR (101 MHz, DMSO-*d*_6_) δ 173.0, 163.3, 155.2, 154.2, 139.4, 136.0, 130.3, 129.3, 127.8, 122.4, 113.7, 110.3 (2C), 110.0, 58.7, 55.5, 27.48, 15.9. HRMS (ES TOF) *m*/*z* (M + Na)^+^ Calcd for C_18_H_16_N_2_NaO_4_ 347.1002; Found 347.1016.

(*Z*)-5-Ethyl-2-(hydroxyimino)-1′-methyl-2*H*-spiro[benzofuran-3,3′-indolin]-2′-one (**13eb**). This compound was prepared *via* **Method C** employing 3-(5-ethyl-2-hydroxyphenyl)-1-methyl-3-(nitromethyl)indolin-2-one **7eb** (82 mg, 0.25 mmol) in a yield of 68 mg (0.22 mmol, 88%). Purification was performed by column chromatography (hexane/EtOAc, 3:1, *v*/*v*). The title compound was obtained as white solid, m.p. 252–254 °C, *R_f_* 0.38 (hexane/EtOAc, 1:1, *v*/*v*). ^1^H NMR (400 MHz, DMSO-*d*_6_) δ 10.47 (s, 1H), 7.44–7.38 (m, 1H), 7.27–7.21 (m, 2H), 7.18 (d, *J* = 7.9 Hz, 1H), 7.09–7.03 (m, 2H), 6.80 (s, 1H), 3.23 (s, 3H), 2.54–2.50 (m, 2H), 1.08 (t, *J* = 7.6 Hz, 3H). ^13^C NMR (101 MHz, DMSO-*d*_6_) δ 173.0, 155.2, 154.7, 143.9, 140.0, 130.2, 129.4, 129.3, 126.8, 123.9, 123.4, 123.0, 110.4, 109.4, 57.9, 27.5, 26.8, 15.8. HRMS (ES TOF) *m*/*z* (M + Na)^+^ Calcd for C_18_H_16_N_2_NaO_3_ 331.1053; Found 331.1057.

(*Z*)-1′-Benzyl-5-ethyl-2-(hydroxyimino)-2*H*-spiro[benzofuran-3,3′-indolin]-2′-one (**13fb**). This compound was prepared *via* **Method C** employing 1-benzyl-3-(5-ethyl-2-hydroxyphenyl)-3-(nitromethyl)indolin-2-one **7fb** (101 mg, 0.25 mmol) in a yield of 83 mg (0.216 mmol, 87%). Purification was performed by column chromatography (hexane/EtOAc, 3:1, *v*/*v*). The title compound was obtained as white solid, m.p. 182–184 °C, *R_f_* 0.6 (hexane/EtOAc, 1:1, *v*/*v*). ^1^H NMR (400 MHz, CDCl_3_) δ 7.43 (s, 1H), 7.36–7.31 (m, 4H), 7.30–7.26 (m, 1H), 7.26–7.22 (m, 1H), 7.20–7.14 (m, 2H), 7.06–7.00 (m, 2H), 6.80 (d, *J* = 7.9 Hz, 1H), 6.69 (s, 1H), 5.00 (d, *J* = 15.7 Hz, 1H), 4.84 (d, *J* = 15.8 Hz, 1H), 2.55 (q, *J* = 7.6 Hz, 2H), 1.15 (t, *J* = 7.6 Hz, 3H). ^13^C NMR (101 MHz, CDCl_3_) δ 173.6, 157.6, 155.2, 143.0, 140.7, 135.3, 129.9 (2C), 129.7, 129.0 (2C), 127.9, 127.3 (2C), 126.7, 124.5, 123.9, 122.7, 110.9, 110.1, 58.7, 44.3, 28.4, 15.7. HRMS (ES TOF) *m*/*z* (M + Na)^+^ Calcd for C_24_H_20_N_2_NaO_3_ 407.1366; Found 407.1354.

(*Z*)-1′-Butyl-5-ethyl-2-(hydroxyimino)-2*H*-spiro[benzofuran-3,3′-indolin]-2′-one (**13gb**). This compound was prepared *via* **Method C** employing 1-butyl-3-(5-ethyl-2-hydroxyphenyl)-3-(nitromethyl)indolin-2-one **7gb** (92 mg, 0.25 mmol) in a yield of 73 mg (0.208 mmol, 83%). Purification was performed by column chromatography (hexane/EtOAc, 3:1, *v*/*v*). The title compound was obtained as white solid, m.p. 195–197 °C, *R_f_* 0.66 (hexane/EtOAc, 1:1, *v*/*v*). ^1^H NMR (400 MHz, DMSO-*d*_6_) δ 10.49 (s, 1H), 7.41–7.35 (m, 1H), 7.26–7.20 (m, 3H), 7.06–7.01 (m, 2H), 6.70 (s, 1H), 3.78–3.71 (m, 2H), 2.53–2.49 (m, 2H), 1.66–1.59 (m, 2H), 1.37–1.29 (m, 2H), 1.06 (t, *J* = 7.6 Hz, 3H), 0.90 (t, *J* = 7.3 Hz, 3H). ^13^C {^1^H} NMR (101 MHz, DMSO-*d*_6_) δ 173.0, 155.3, 154.7, 143.2, 140.0, 130.3, 129.7, 129.6, 127.0, 124.2, 123.3, 122.6, 110.5, 109.6, 58.0, 29.0, 27.5 (2C), 19.3, 15.7, 13.7. HRMS (ES TOF) *m*/*z* (M + Na)^+^ Calcd for C_21_H_22_N_2_NaO_3_ 373.1523; Found 373.1526.

(*Z*)-1′-Allyl-5-ethyl-2-(hydroxyimino)-2*H*-spiro[benzofuran-3,3′-indolin]-2′-one (**13hb**). This compound was prepared *via* **Method C** employing 1-allyl-3-(5-ethyl-2-hydroxyphenyl)-3-(nitromethyl)indolin-2-one **7hb** (88 mg, 0.25 mmol) in a yield of 69 mg (0.206 mmol, 82%). Purification was performed by column chromatography (hexane/EtOAc, 3:1, *v*/*v*). The title compound was obtained as yellow oil, *R_f_* 0.52 (hexane/EtOAc, 1:1, *v*/*v*). ^1^H NMR (400 MHz, CDCl_3_) δ 7.44 (s, 1H), 7.35–7.30 (m, 1H), 7.18–7.11 (m, 2H), 7.08–7.02 (m, 2H), 6.91 (d, *J* = 7.9 Hz, 1H), 6.71–6.64 (m, 1H), 5.91–5.81 (m, 1H), 5.29 (d, *J* = 17.3 Hz, 1H), 5.25 (d, *J* = 10.4 Hz, 1H), 4.42–4.30 (m, 2H), 2.53 (q, *J* = 7.6 Hz, 2H), 1.13 (t, *J* = 7.6 Hz, 3H). ^13^C NMR (101 MHz, CDCl_3_) δ 173.2, 157.6, 155.2, 143.2, 140.7, 130.8, 130.0, 129.9, 129.7, 126.6, 124.5, 123.8, 122.8, 117.9, 110.9, 109.9, 58.7, 42.9, 28.4, 15.7. HRMS (ES TOF) *m*/*z* (M + Na)^+^ Calcd for C_20_H_18_N_2_NaO_3_ 357.1210; Found 357.1211.

### 3.5. Scale-Up Experiment

3-Hydroxy-3-(nitromethyl)indolin-2-one (**9a**) (2.08 g, 10.0 mmol) and 4-chloro-3-methylphenol (**2e**) (1.42 g, 10.0 mmol) were placed in a suitable round-bottom flask equipped with a magnetic stir bar, and methanesulfonic acid (15 mL) was added. The resulting mixture was stirred at 70 °C for 1 h. After completion of the reaction, as monitored by TLC, the reaction mixture was poured into 100 mL of cold water and extracted with EtOAc (3 × 40 mL). The combined organic layers were evaporated under reduced pressure, and the crude product was purified by column chromatography on silica gel using hexane/EtOAc as the eluent to afford **7ae** as a white solid (2.76 g, 8.30 mmol, 83%). The isolated Friedel–Crafts product **7ae** (2.76 g, 8.30 mmol) was subjected to the base-promoted cyclization under modified conditions based on **Method C** (dissolved in a mixture of 20 mL of ethanol with 5 mL of water, and then 30 mmol of KOH was added). The reaction mixture was heated under reflux until complete conversion of the starting material, as monitored by TLC. After completion, the reaction mixture was worked up according to Method C, and the crude product was purified and subsequently recrystallized from ethanol to afford (Z)-5-chloro-2-(hydroxyimino)-6-methyl-2*H*-spiro[benzofuran-3,3′-indolin]-2′-one (**13ae**) in 74% yield (1.93 g).

## 4. Conclusions

In conclusion, we have established a condition-controlled divergent reactivity of nitroaldol isatin adducts with phenols and related electron-rich aromatic compounds. Under methanesulfonic acid conditions, the nitroalcohols undergo Friedel–Crafts alkylation to furnish 3-aryl-3-(nitromethyl)indolin-2-ones in yields of up to 99%. The reaction accommodates a range of alkyl, halogen, hydroxy, alkoxy, and *N*-substituted substrates, although several electronically deactivated substrates and α-naphthol were not compatible with the reaction conditions. Prolonged acidic treatment of selected Friedel–Crafts adducts provided chromeno[3,4-*b*]indol-6(7*H*)-ones through an unusual C3-to-C2 skeletal rearrangement. Although this transformation has limited preparative efficiency, it is mechanistically notable because it converts the nitroaldol-derived oxindole framework into a distinct tetracyclic scaffold. Seven chromenoindolones were obtained in total, including five previously unreported compounds.

In contrast, treatment of the Friedel–Crafts adducts with KOH in aqueous ethanol promoted intramolecular cyclization to spirocyclic hydroxyimino derivatives. Sixteen compounds of this type were obtained in 80–96% yields. X-ray crystallography established the structure and *Z* configuration of representative product **13ab**, while the configurations of the remaining products were assigned on the basis of NMR data and comparison with the crystallographically characterized compound. In total, 51 new compounds were synthesized, including 16 spirocyclic oxime derivatives and five new chromenoindolones.

The present results show that nitroaldol isatin adducts can serve as versatile precursors whose reactivity can be directed toward structurally distinct products by changing the reaction conditions. The proposed mechanisms for the skeletal rearrangement and nitro-to-oxime conversion provide plausible interpretations of the available experimental observations but remain provisional because the corresponding intermediates were not directly detected. Further mechanistic studies will be required to clarify the detailed pathways of these transformations.

## Figures and Tables

**Figure 1:**
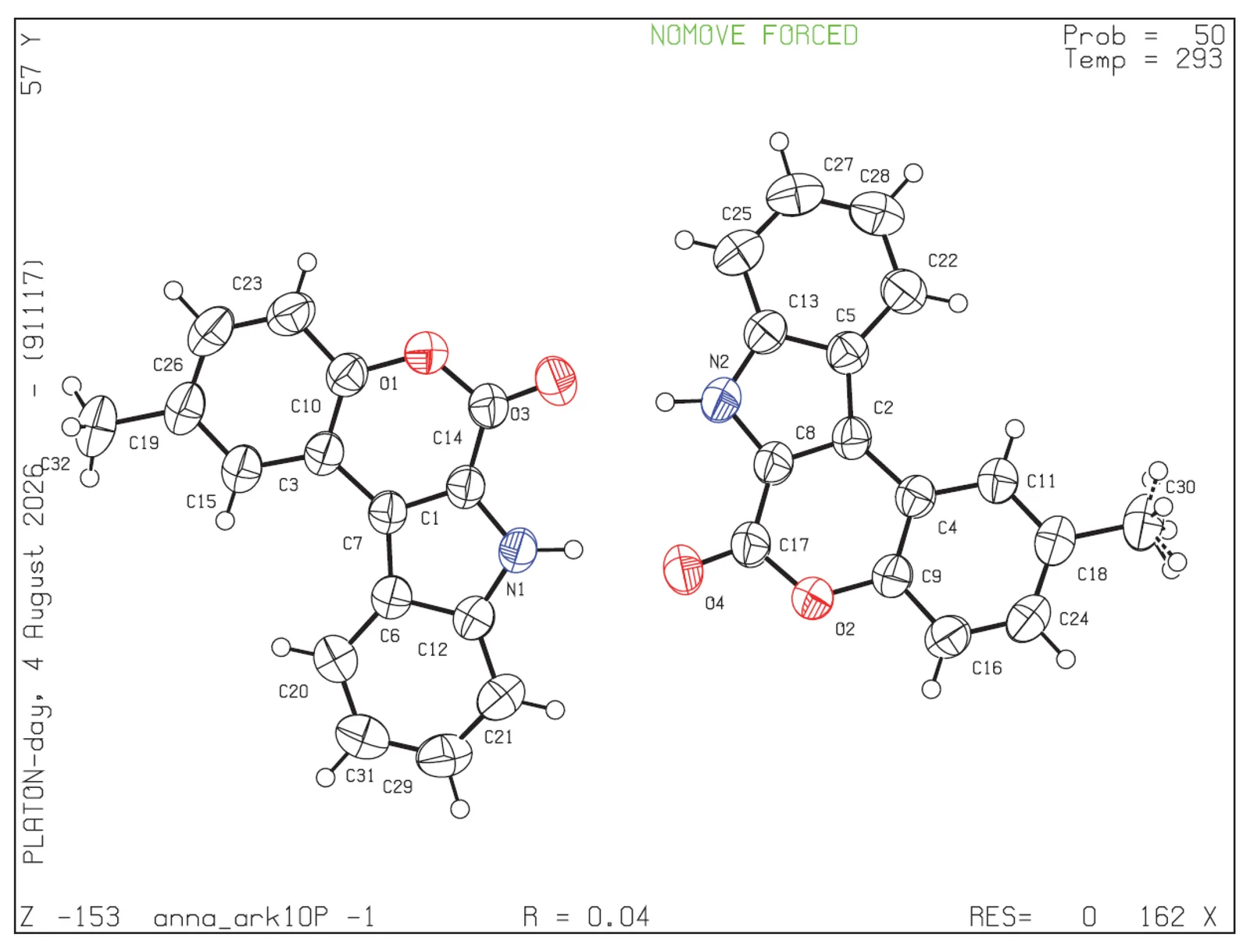
ORTEP drawing of the X-ray structure of 2-Methylchromeno[3,4-b]indol-6(7H)-one (**8aa**, CCDC #2578781). The thermal ellipsoids are shown at 50% probability. The carbon atoms are shown in black, oxygen in red, and nitrogen in blue. Hydrogen atoms are represented by black empty circles.

**Figure 2:**
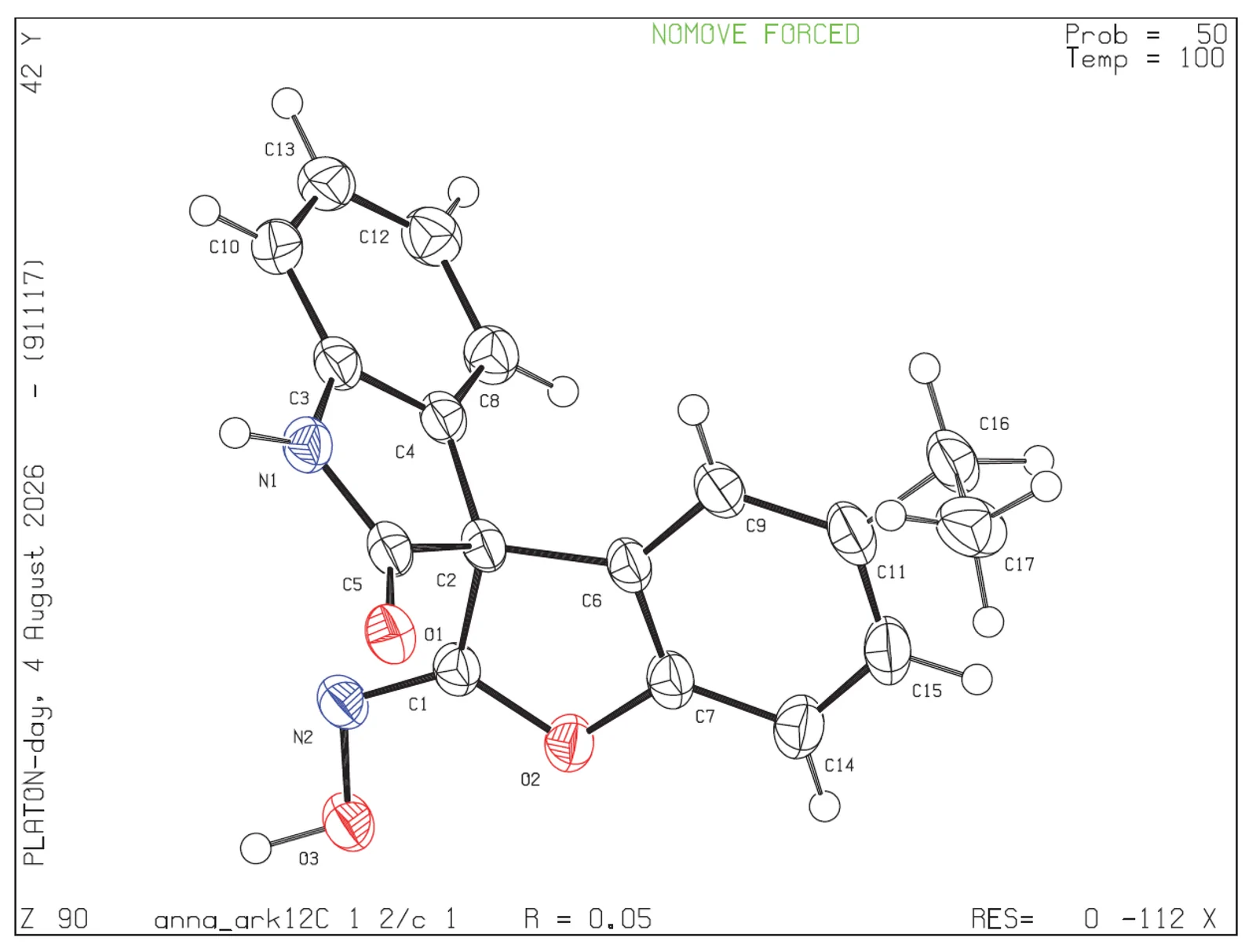
ORTEP drawing of the X-ray structure of (*Z*)-5-Ethyl-2-(hydroxyimino)-2*H*-spiro[benzofuran-3,3′-indolin]-2′-one (**13ab**, CCDC #2578801). The thermal ellipsoids are shown at 50% probability. The carbon atoms are shown in black, oxygen in red, and nitrogen in blue. Hydrogen atoms are represented by black empty circles.

**Figure 3:**
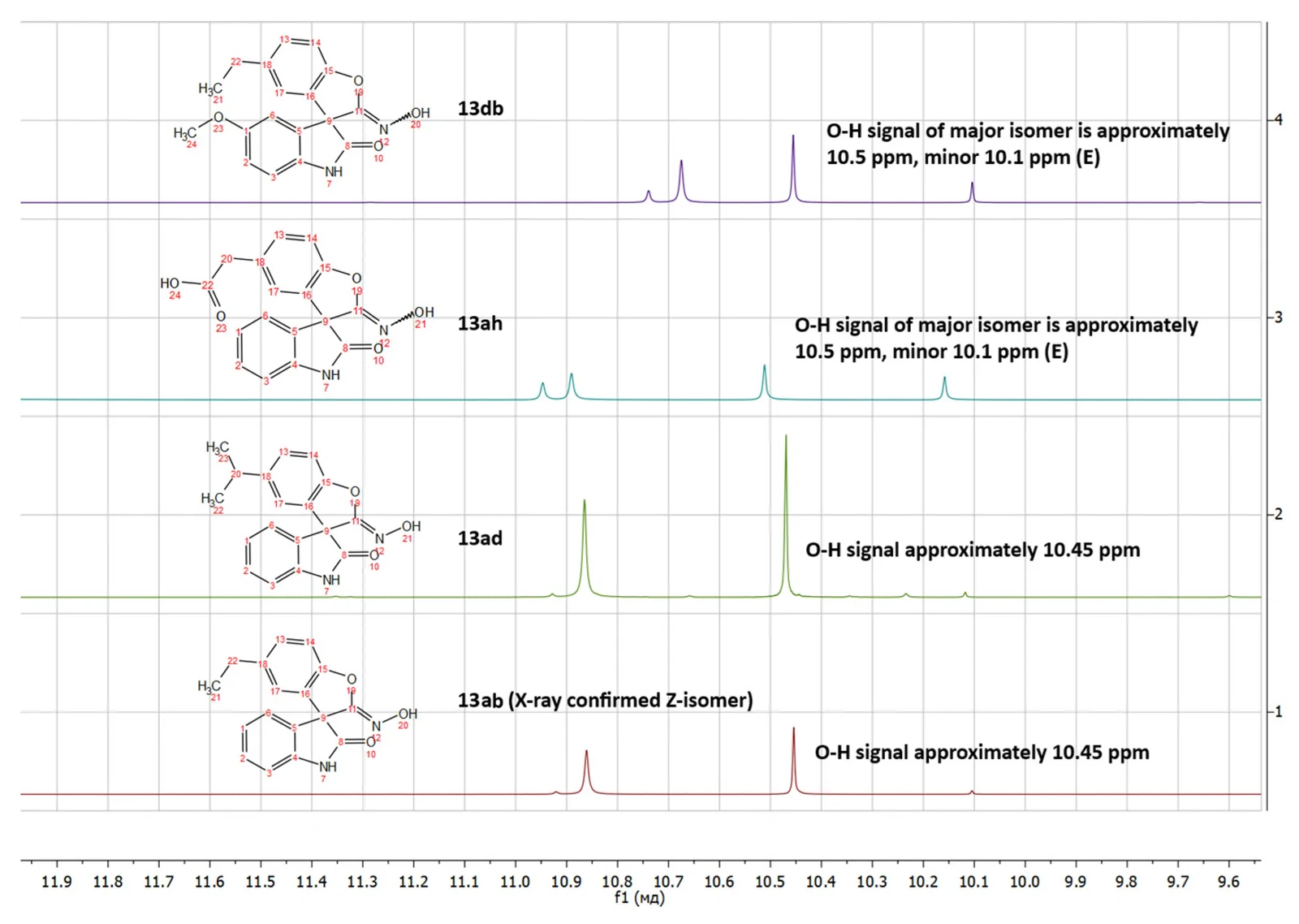
Assignment of ^1^H NMR spectra for compounds **13ab**, **13ad**, **13ah**, **13db**.

**Scheme 2:**
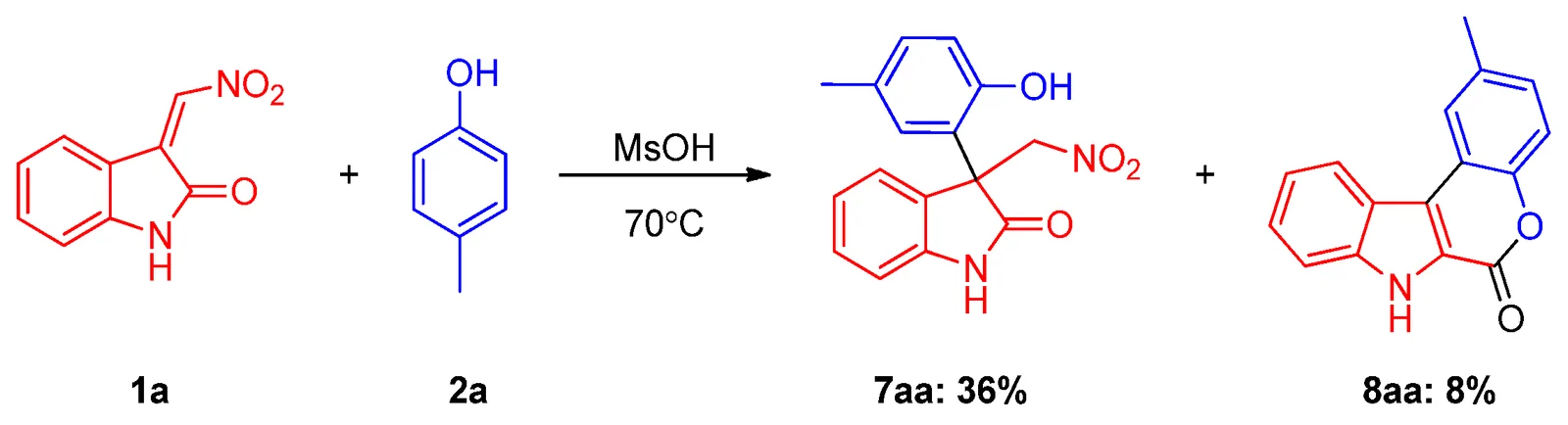
Initial reaction of nitroalkene **1a** with phenol **2a**.

**Scheme 3:**
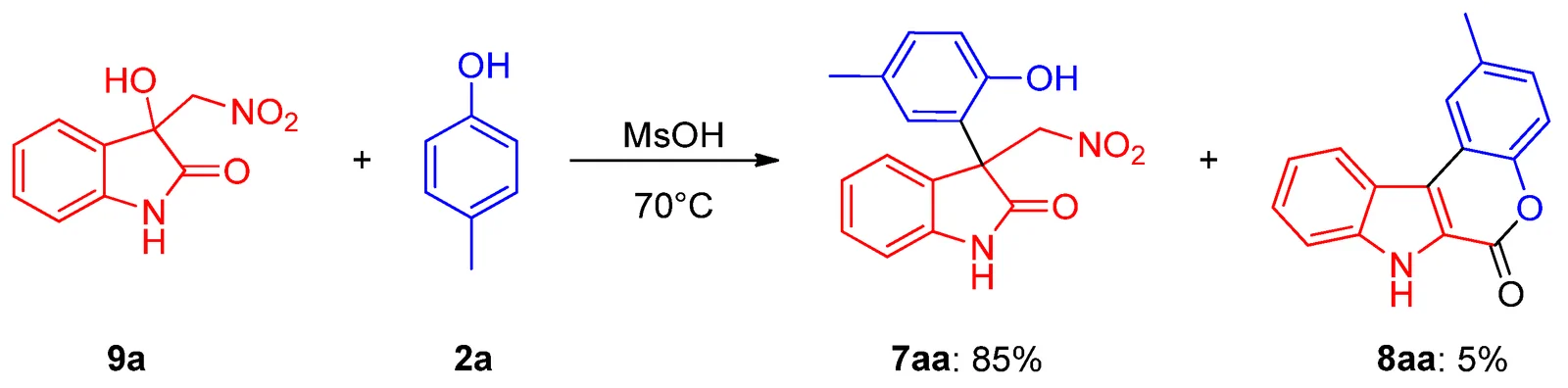
Friedel–Crafts reaction of nitroalcohol **9a** with phenol **2a**.

**Scheme 4:**
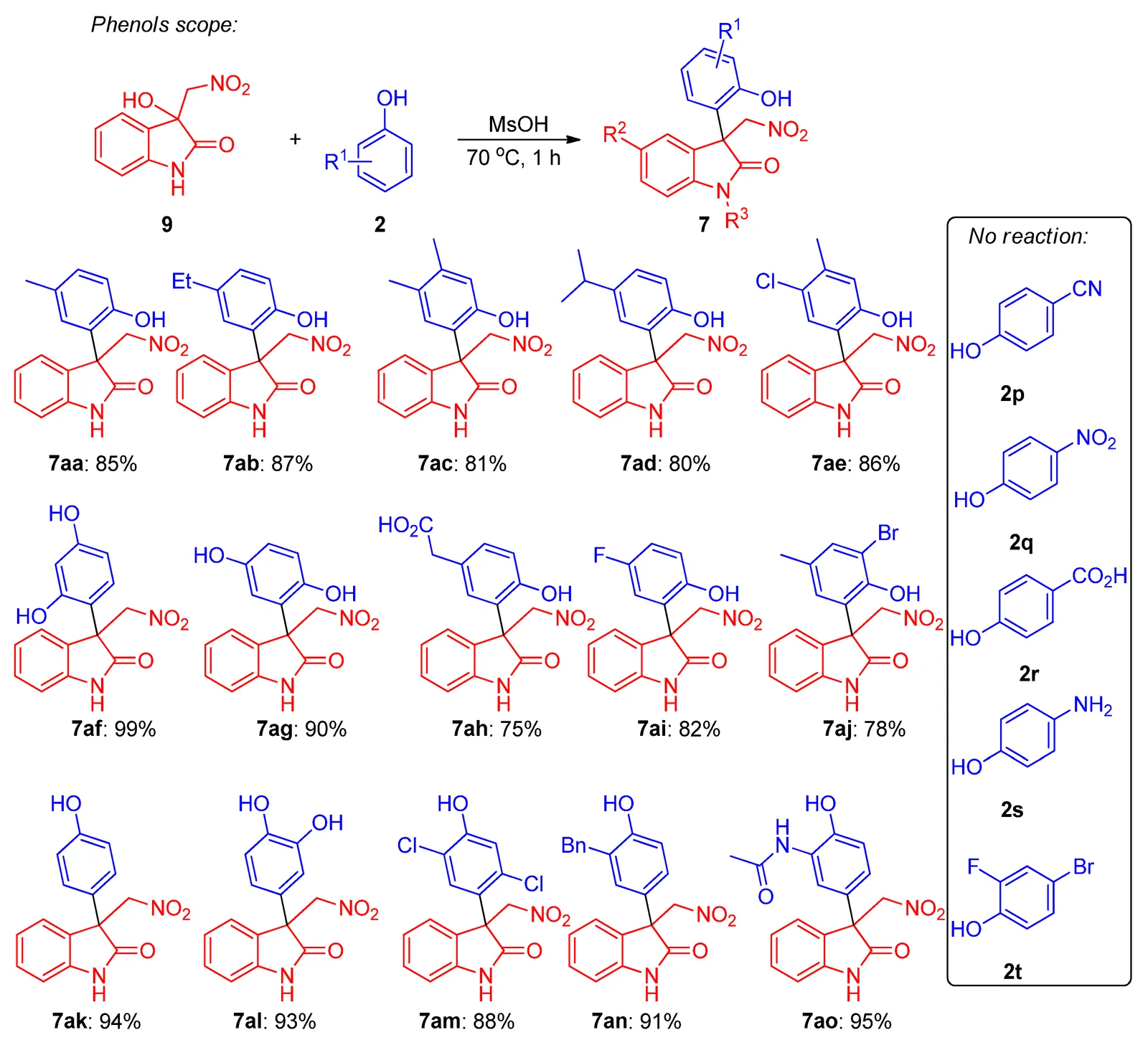
Scope of phenolic reaction partners.

**Scheme 5:**
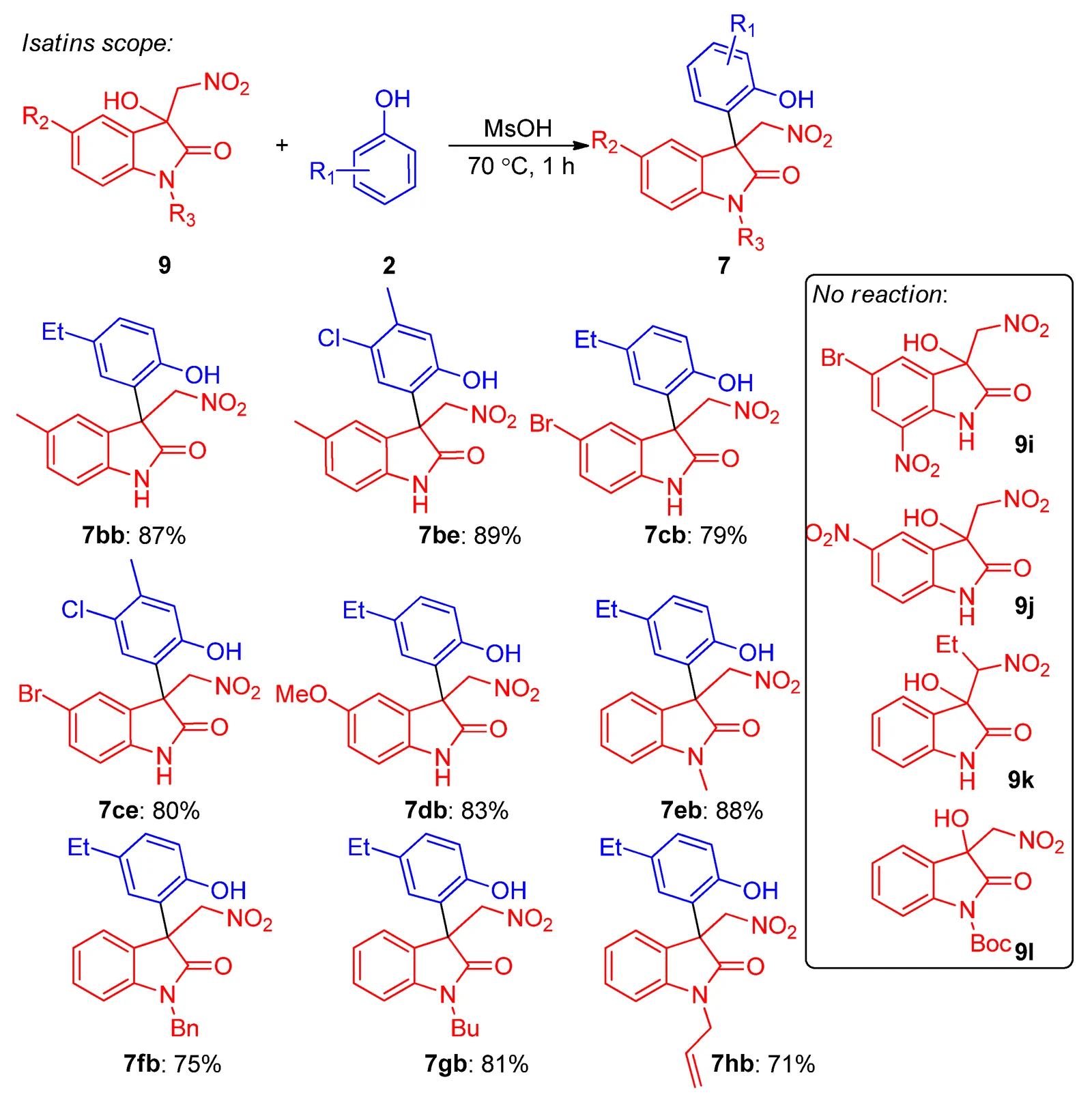
Scope of nitroalcohol reaction partners.

**Scheme 6:**
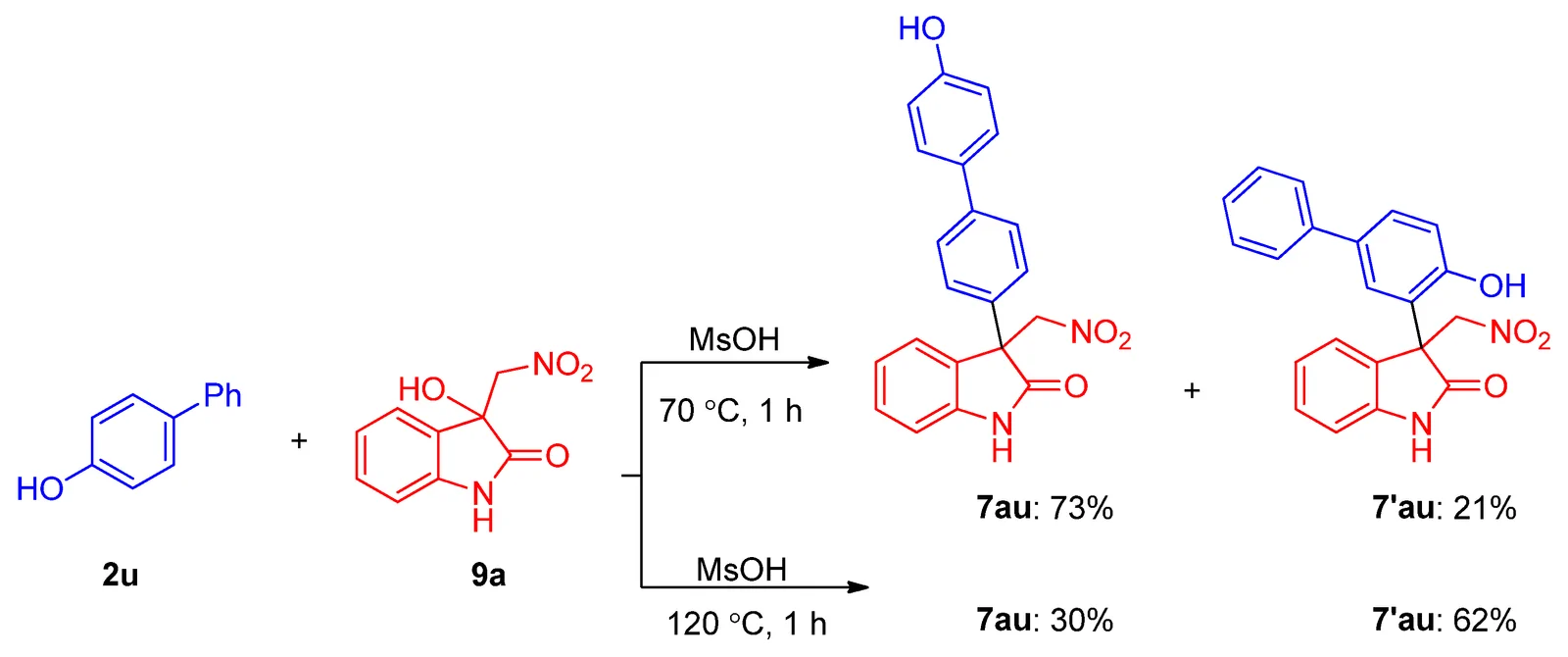
Result of the reaction with 4-phenylphenol **2u** at different temperatures.

**Scheme 7:**
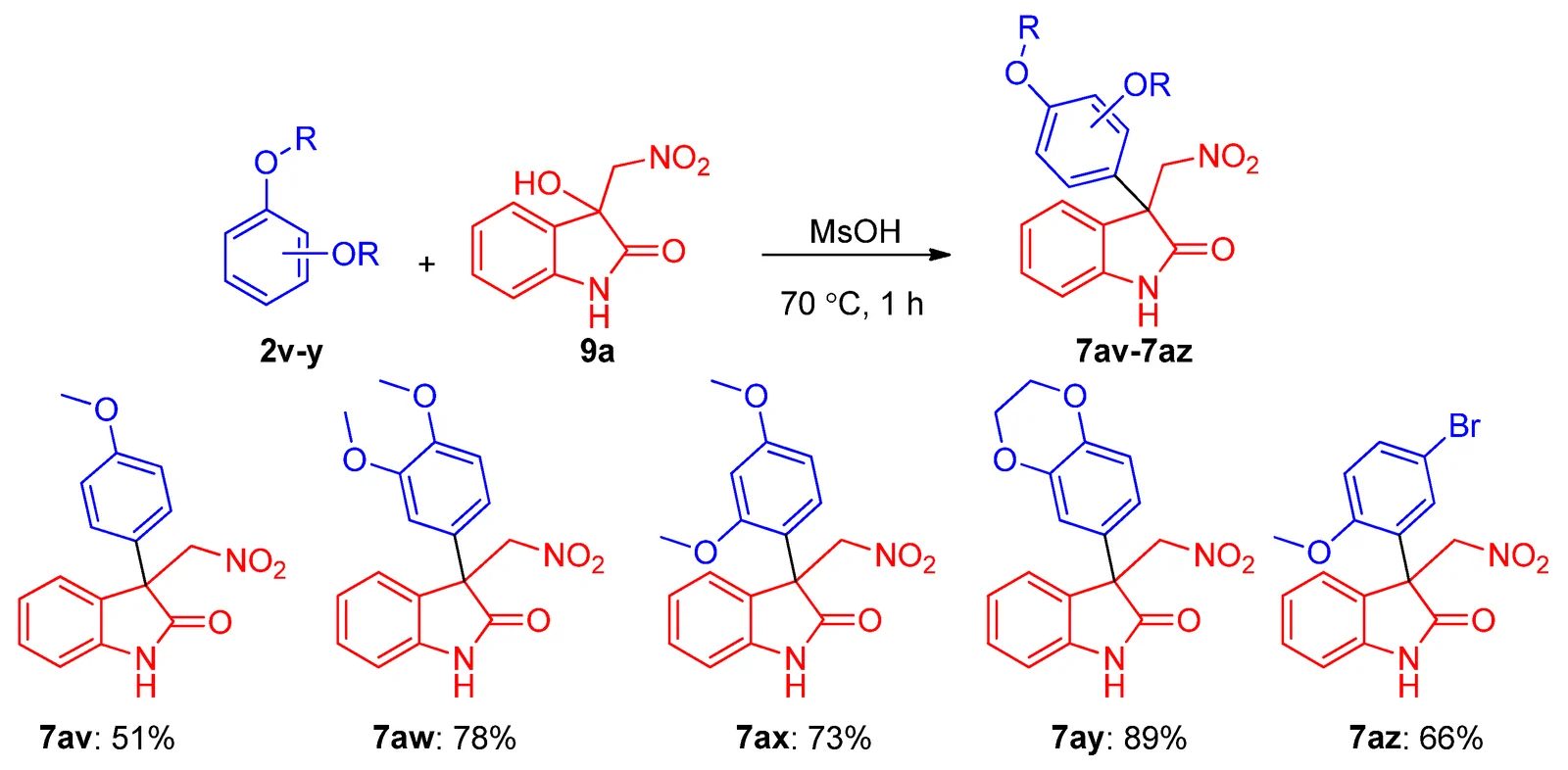
Friedel–Crafts alkylation products **7** synthesized using alkoxybenzenes.

**Scheme 8:**
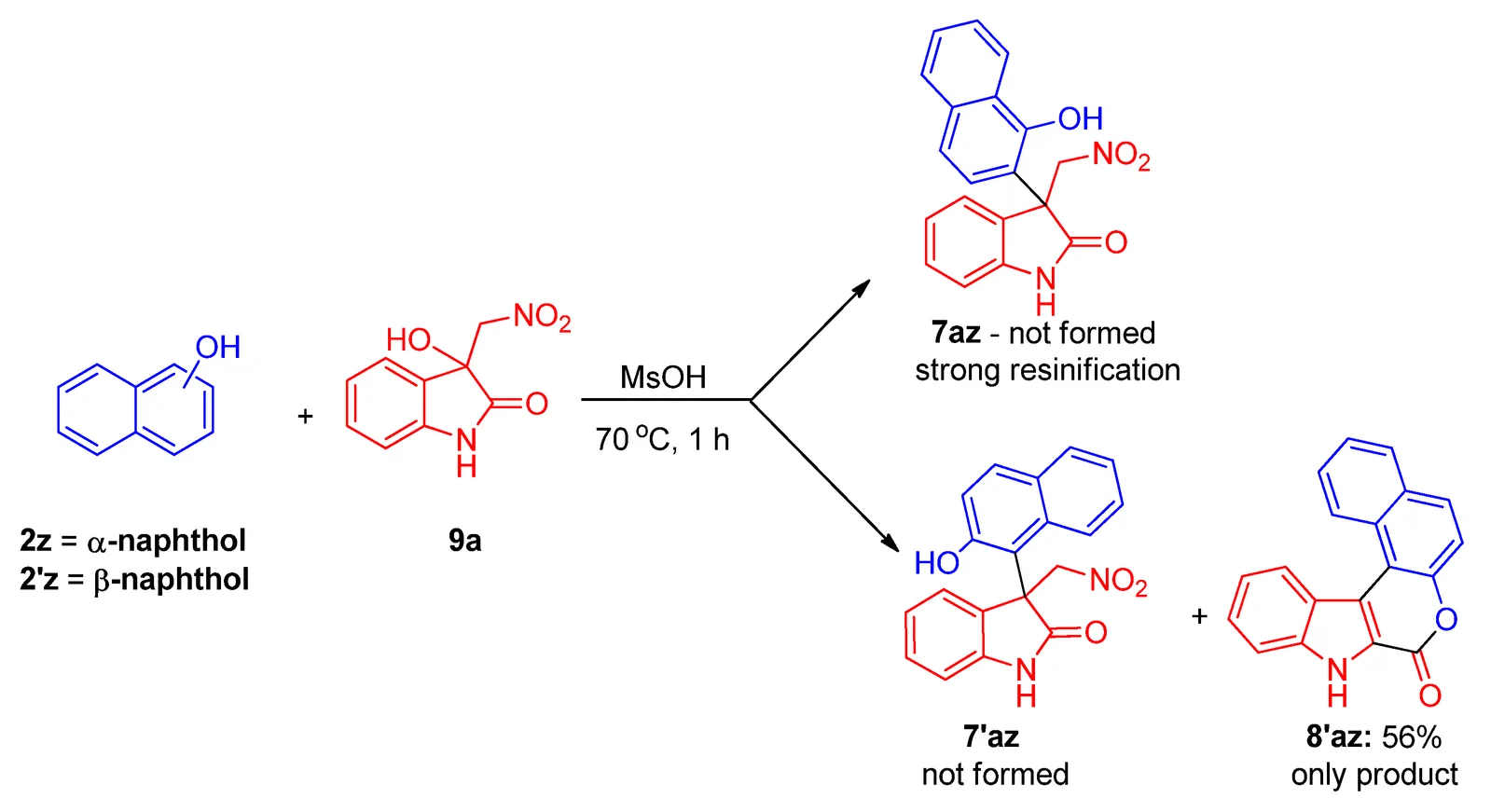
Reactivity of α- and β-naphthols under the Friedel–Crafts alkylation conditions.

**Scheme 9:**
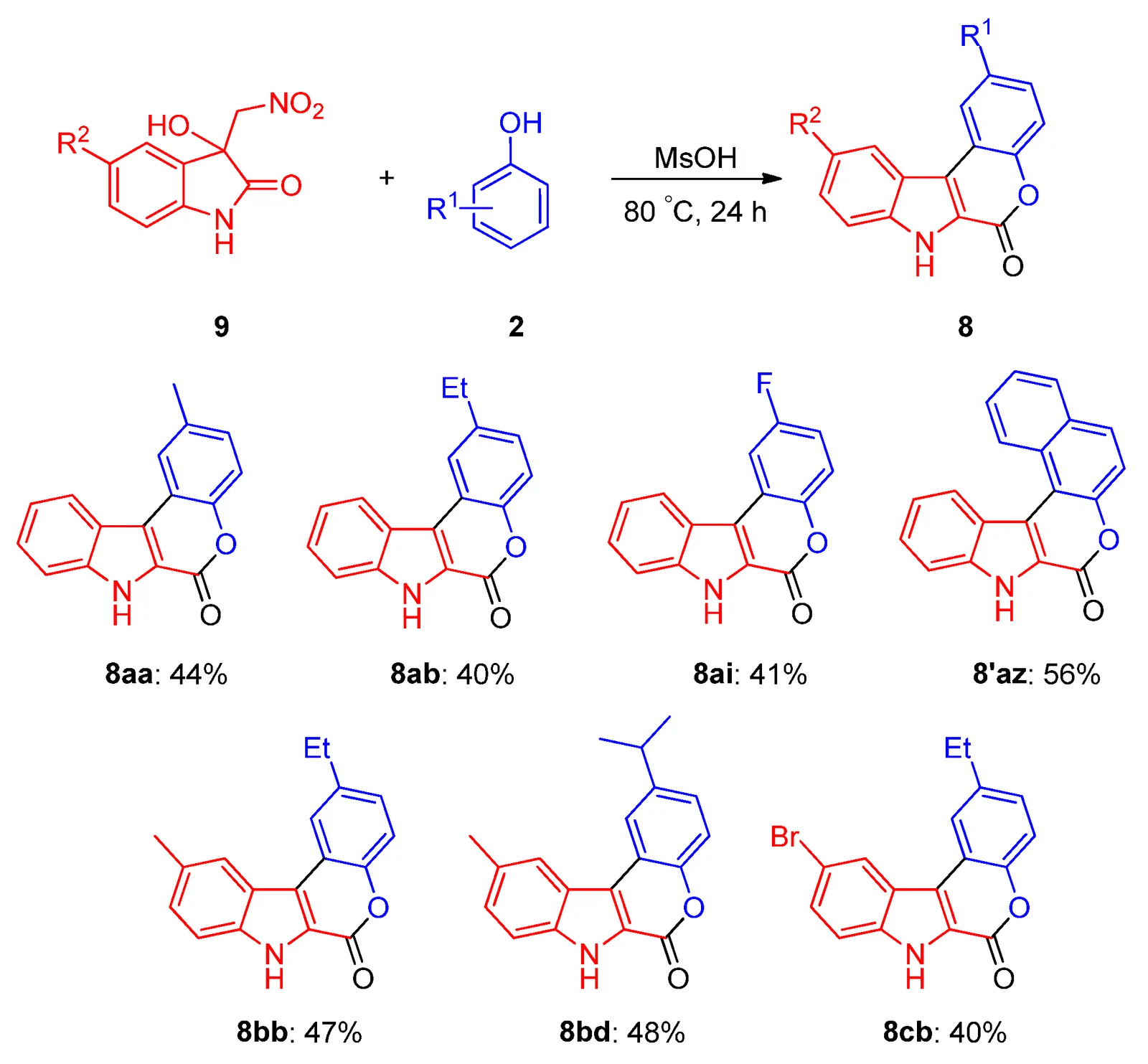
Library of synthesized compounds **8**.

**Scheme 10:**
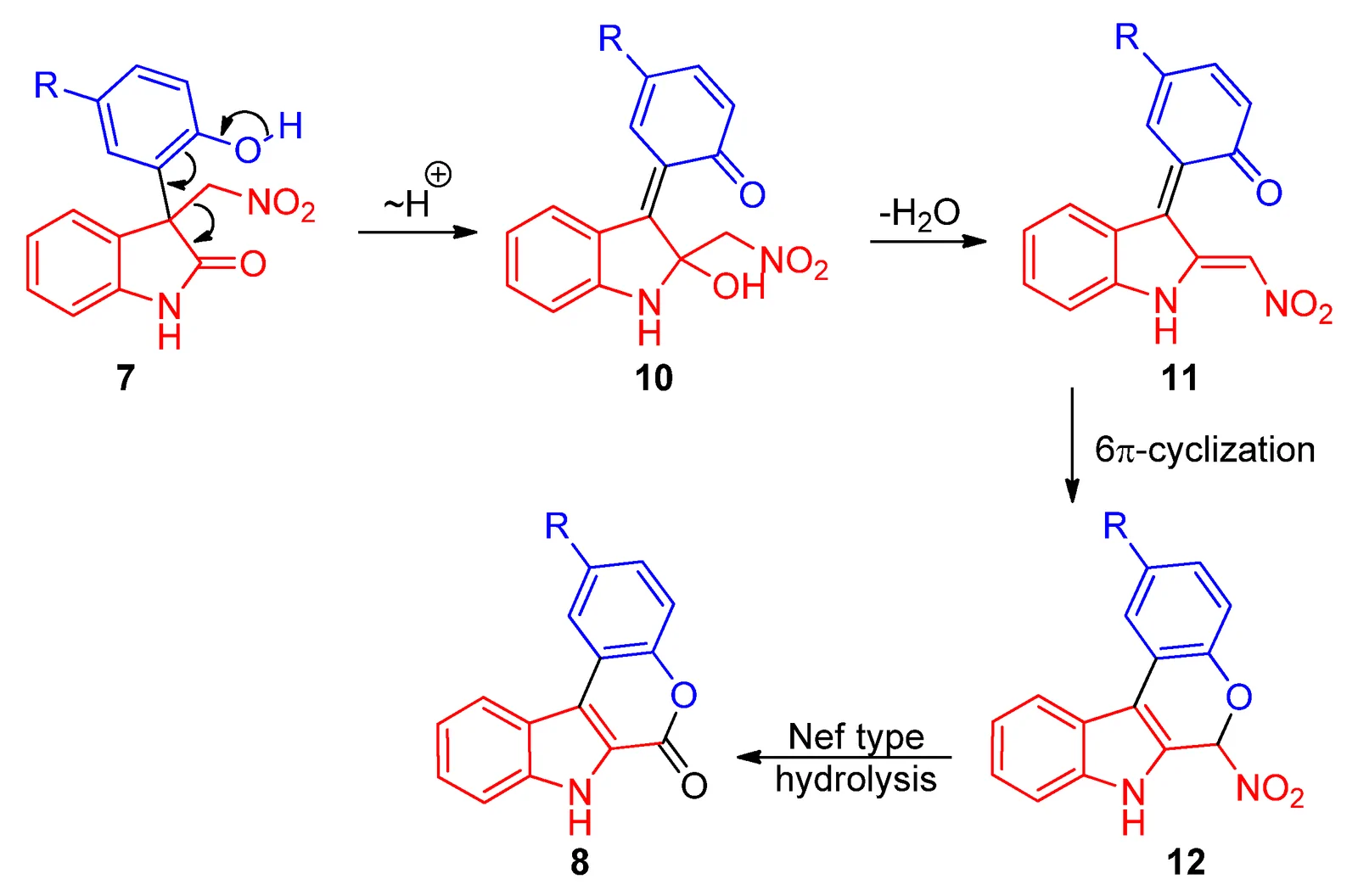
Proposed mechanism for the formation of chromenoindolones **8**.

**Scheme 11:**
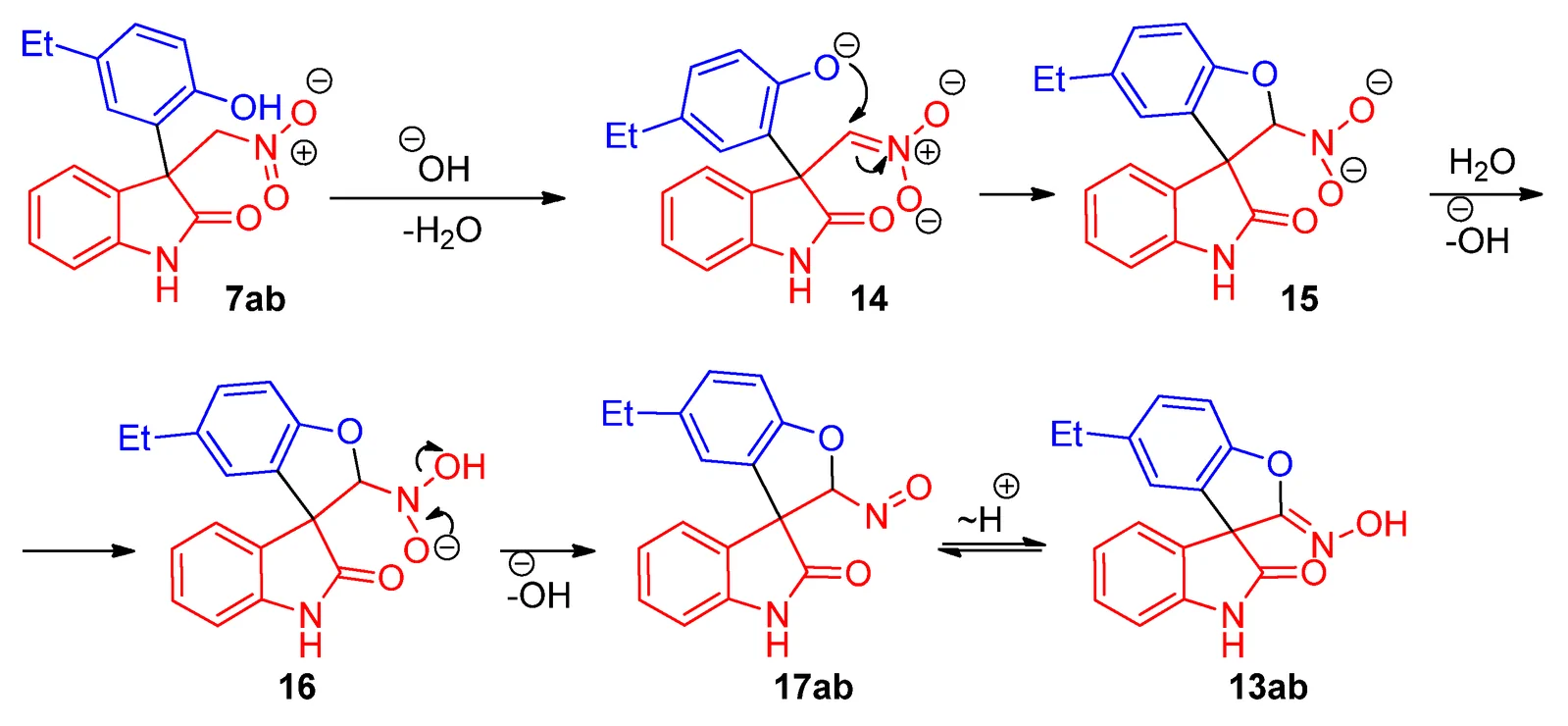
Proposed mechanism for the potassium hydroxide-promoted formation of spirocyclic oximes **13**.

**Scheme 12:**
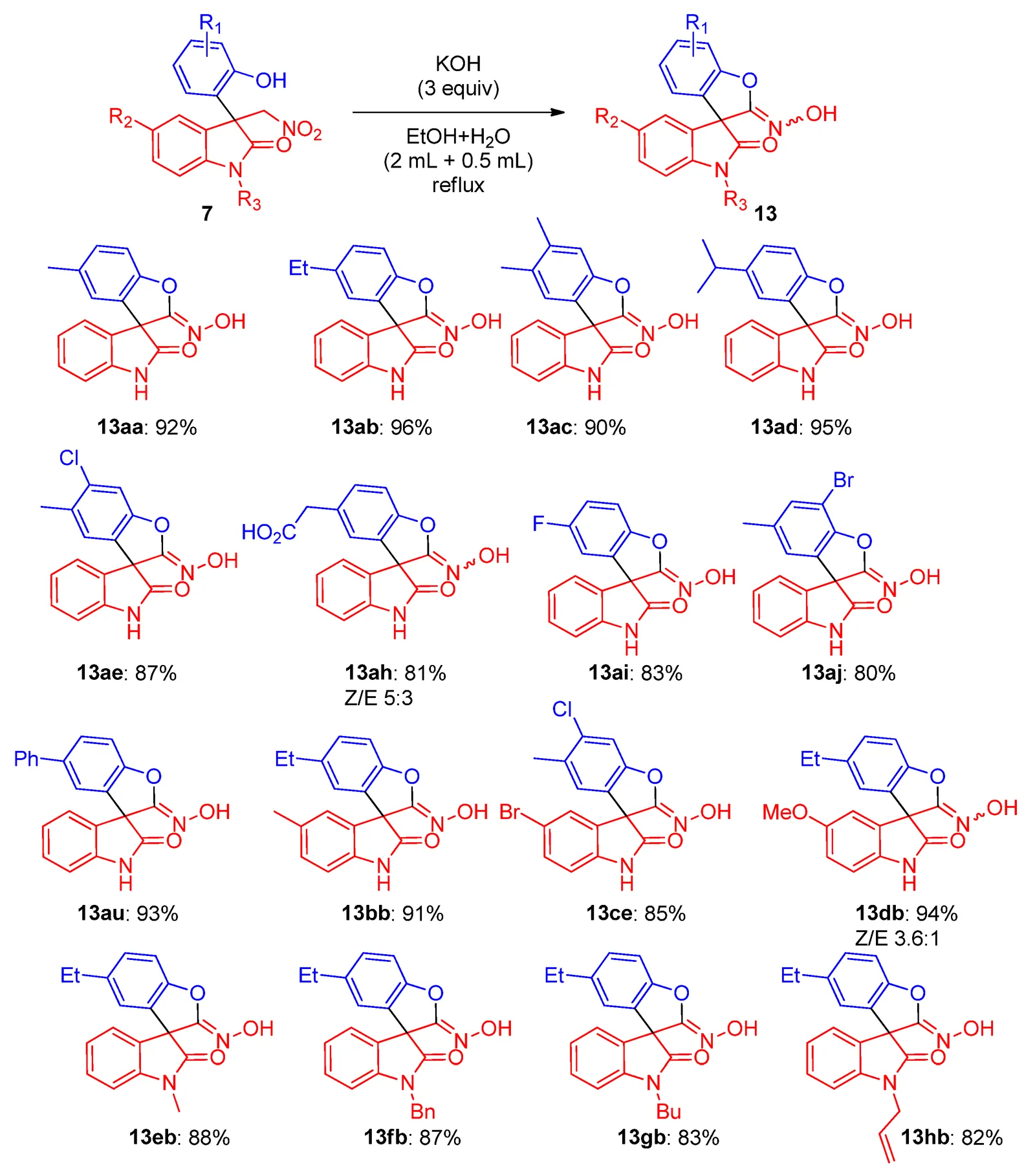
Library of synthesized spirocyclic products **13**.

**Scheme 13:**
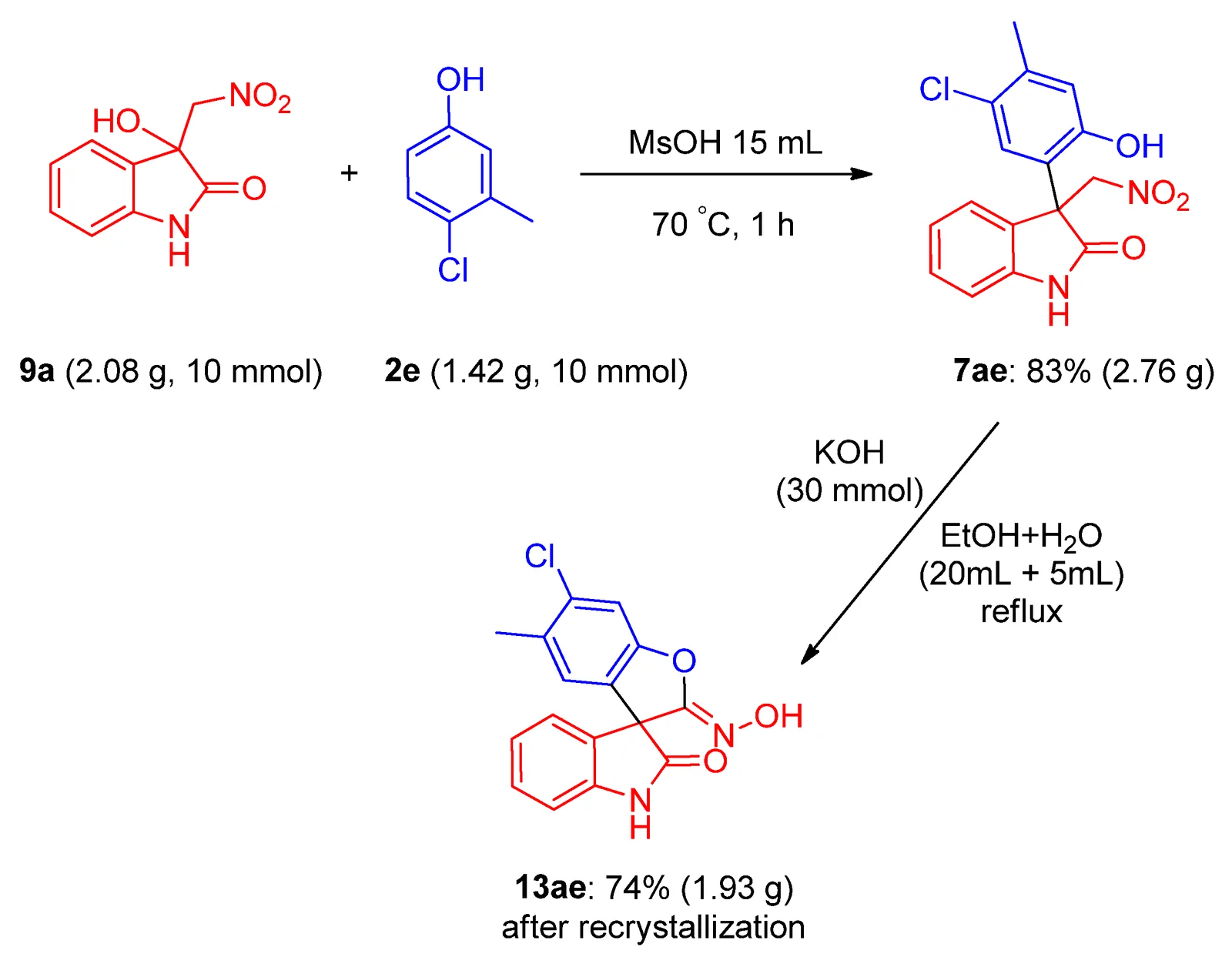
Multigram-scale synthesis of compounds **7ae** and **13ae**.

**Table 1:** Optimization of the reaction conditions for compound 8aa. Optimal conditions are marked in bold. (^a^ 0.5 mmol scale, ^b^ 1 mL of MsOH, ^c^ isolated yields).

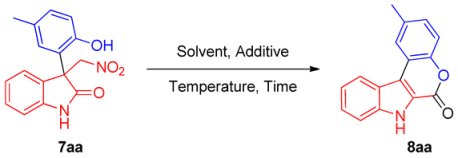						
# ^a^	Solvent ^b^	Additive, mmol	T, °C	Time, h	Conversion, %	Yield, % ^c^
1	MsOH	-	70	2	12	10
2	MsOH	-	100	2	30	15
3	MsOH	-	130	2	43	18
4	MsOH	POCl_3_ (2 equiv)	70	2	54	17
5	MsOH	PCl_5_ (2 equiv)	70	2	35	12
6	MsOH	-	70	10	30	25
7	MsOH	-	70	24	57	32
**8**	**MsOH**	**-**	**80**	**24**	**85**	**44**
9	MsOH	-	90	24	100	37

## Data Availability

The original contributions presented in this study are included in the article/Supplementary Material. Further inquiries can be directed to the corresponding author.

## Supplementary Materials

The following supporting information can be downloaded at https://www.mdpi.com/article/10.3390/ijms27209320/s1 [23,24,25].
