# Supplementary material for: Condition-Controlled Divergent Reactivity of Nitroaldol Isatin Adducts with Phenols: Friedel–Crafts Alkylation, Skeletal Rearrangement, and Spirocyclic Oxime Formation
Source: Int J Mol Sci. 2026 Oct 20;27(20):9320. doi: 10.3390/ijms27209320 (PMC13704967; doi:10.3390/ijms27209320)
Supplement: Supplementary file 1 [file ijms-27-09320-s001.zip › ijms-4627856-supplementary.pdf]

Electronic Supplementary Material (ESI)

Condition-Controlled Divergent Reactivity of Nitroaldol Isatin Adducts with Phenols: Friedel–Crafts Alkylation, Skeletal Rearrangement, and Spirocyclic Oxime Formation

Nikolai A. Arutiunov*, Daria A. Shtal, Kirill V. Tolstov, Anastasia A. Korneeva, Olga V. Petrova, Anna M. Zatsepilina, Nicolai A. Aksenov, Sergei N. Ovcharov, Alexander V. Aksenov*.

Department of Chemistry, North Caucasus Federal University, 1a Pushkin St., Stavropol 355009, Russian Federation. E-mail: naarutiunov@ncfu.ru

Table of Contents

1. ^{1}H, ^{13}C, ^{19}F NMR spectral charts of compounds **7**…………………………………………………… S2
2. ^{1}H, ^{13}C, ^{19}F NMR spectral charts of compounds **8**…………………………………………………… S65
3. ^{1}H, ^{13}C, ^{19}F NMR spectral charts of compounds **13**…………………………………………………… S80
4. X-Ray Crystallography data for 2-Methylchromeno[3,4-*b*]indol-6(7*H*)-one **8aa**………………………………… S113
5. X-Ray Crystallography data for (*Z*)-5-Ethyl-2-(hydroxyimino)-2*H*-spiro[benzofuran-3,3'-indolin]-2'-one **13ab**………….. S124
6. References……………………………………………………………………………………………... S134

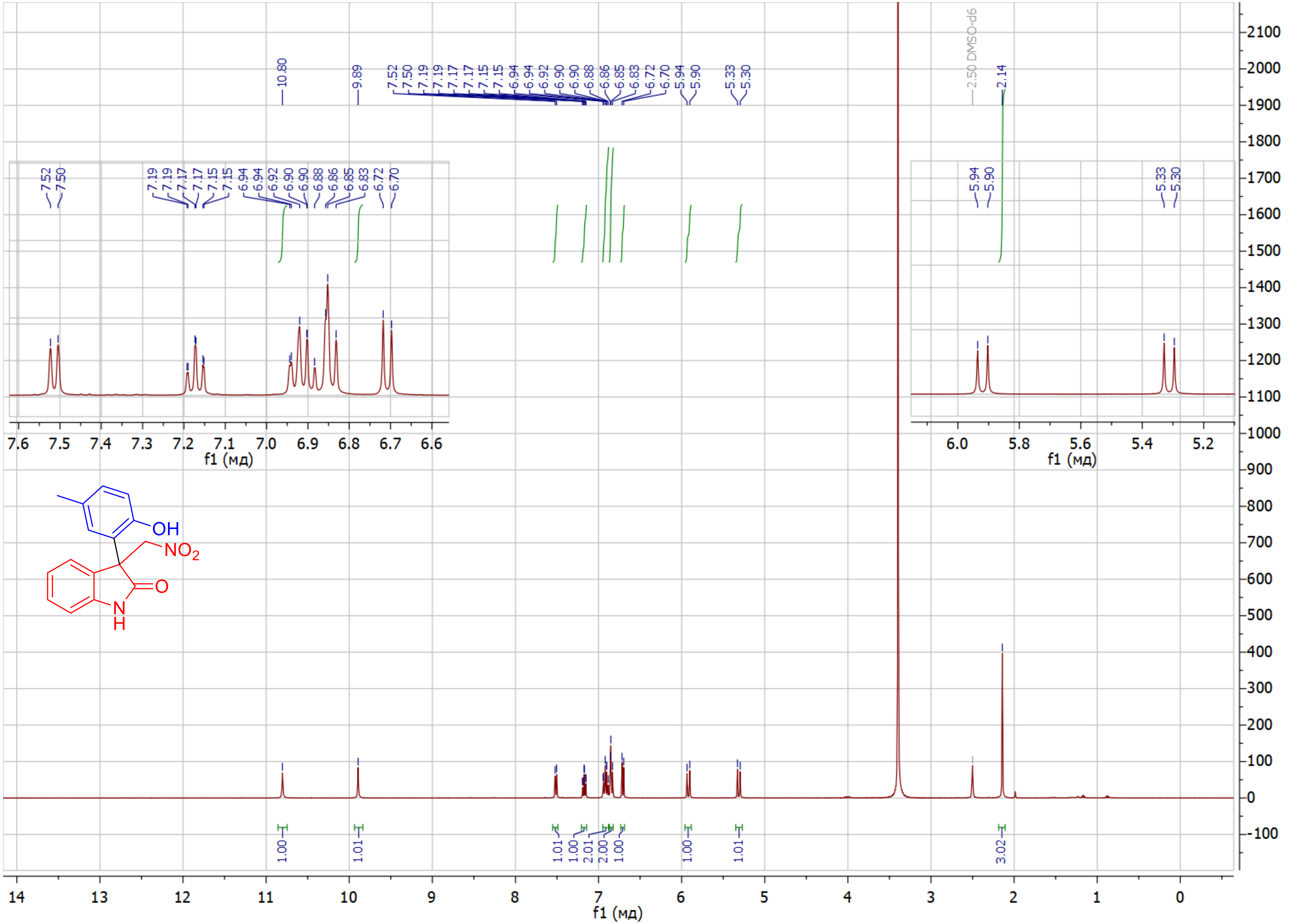

Figure S1. ^{1}H NMR spectrum of compound **7aa** in DMSO-d_6 (400 MHz)

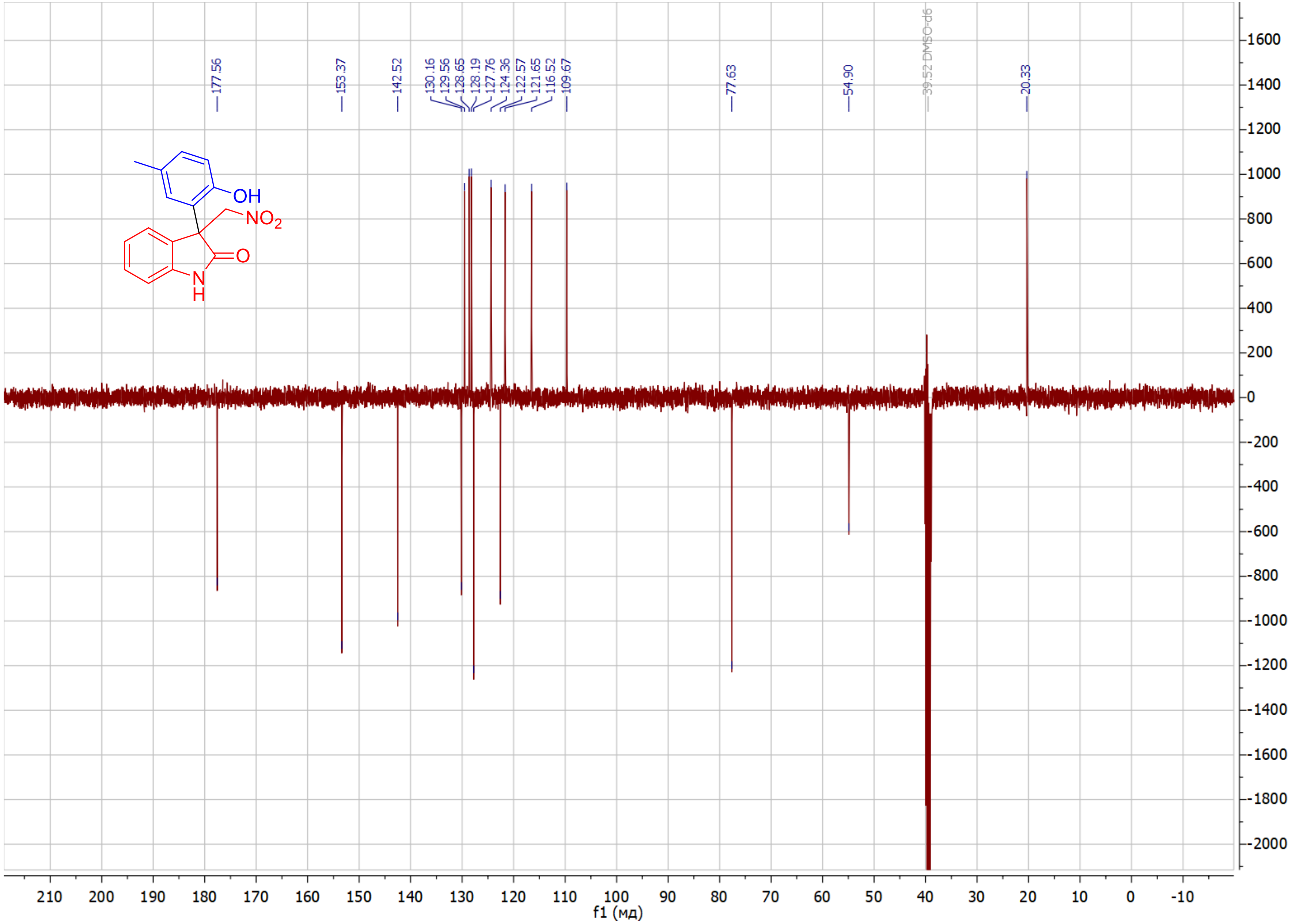

Figure S2. ^{13}C DEPTQ {^{1}H} NMR spectrum of compound **7aa** in DMSO-d_6 (101 MHz)

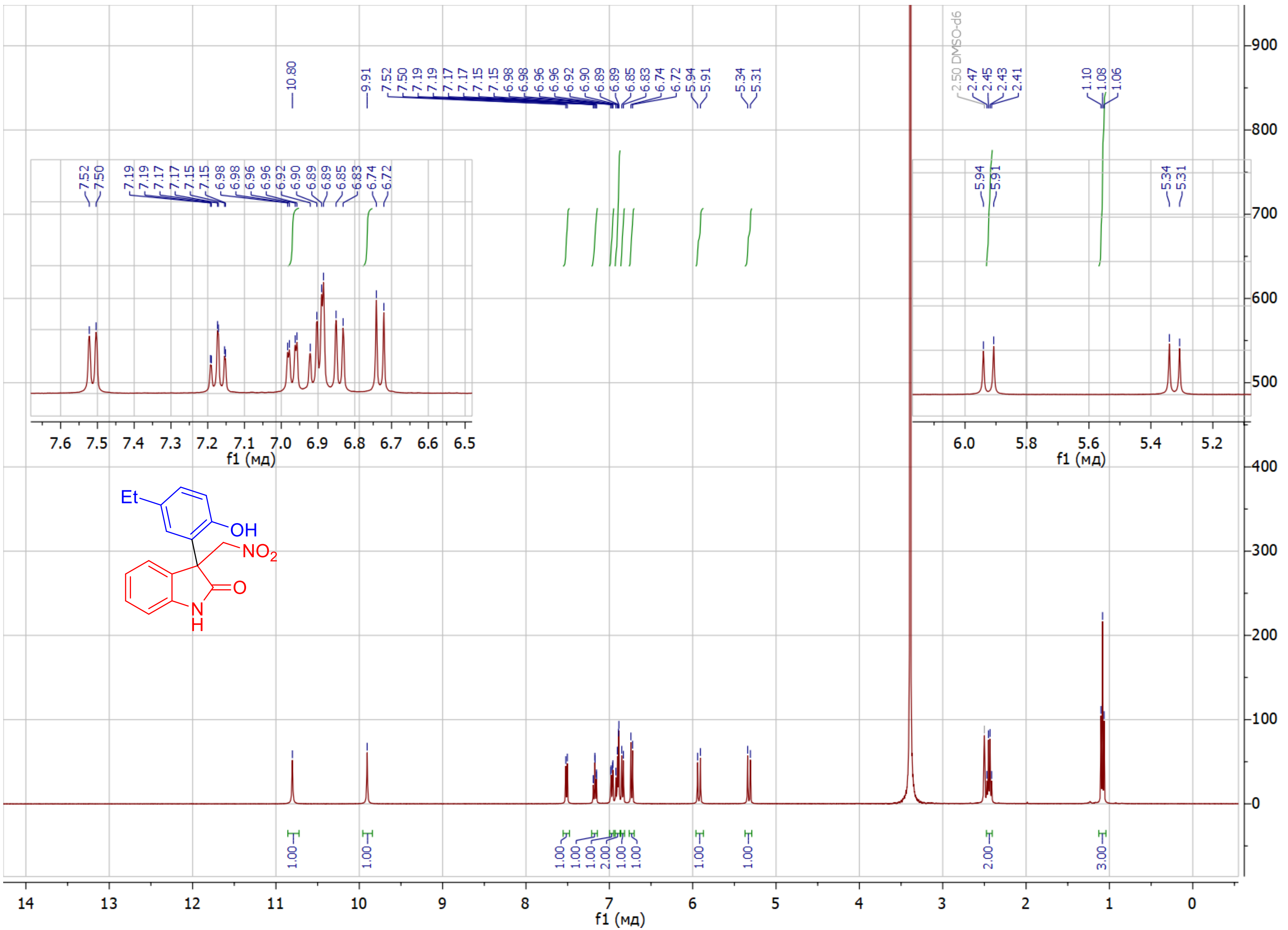

Figure S3. ^{1}H NMR spectrum of compound **7ab** in DMSO-d_6 (400 MHz)

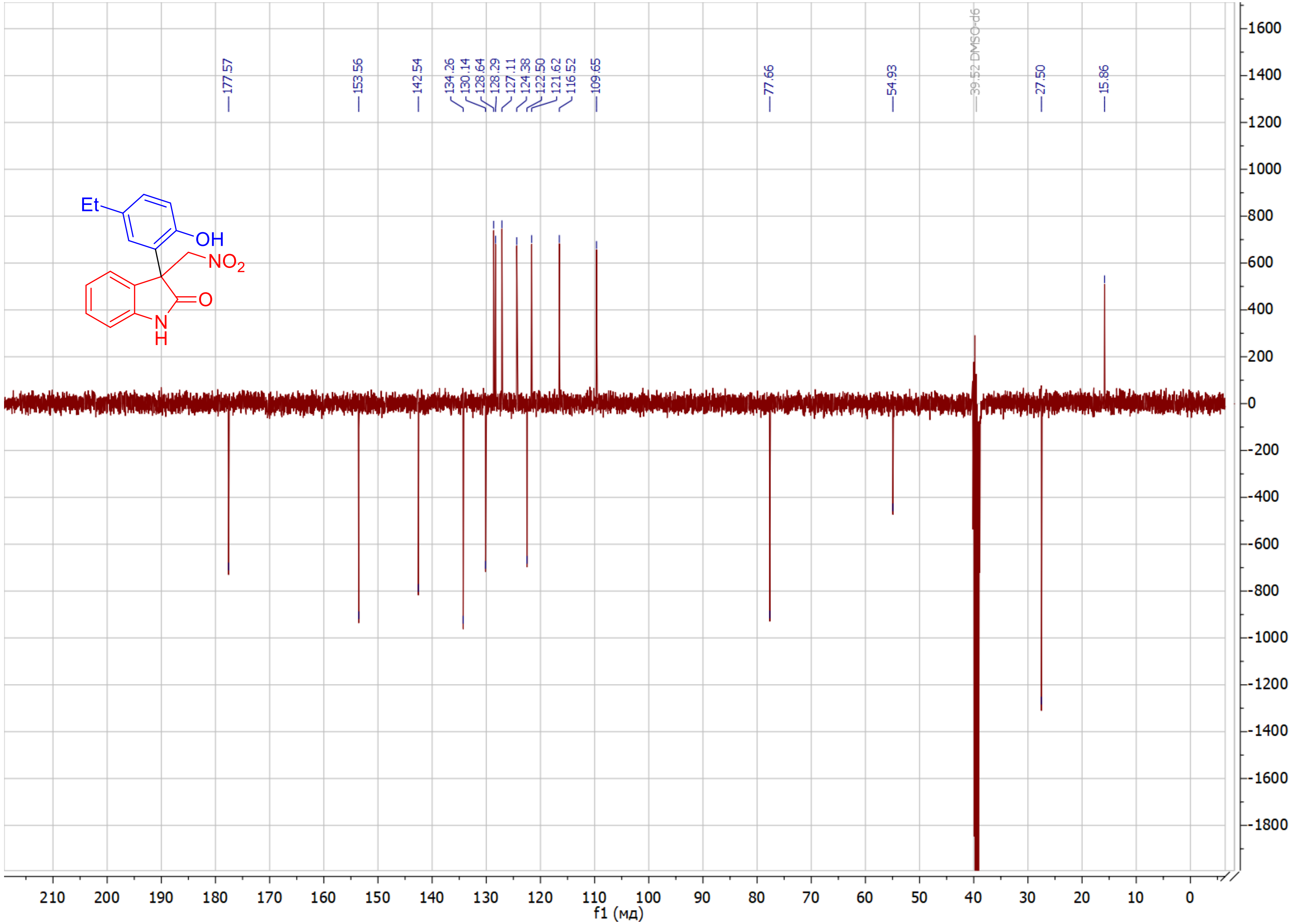

Figure S4. ^{13}C DEPTQ {^{1}H} NMR spectrum of compound **7ab** in DMSO-d_6 (101 MHz)

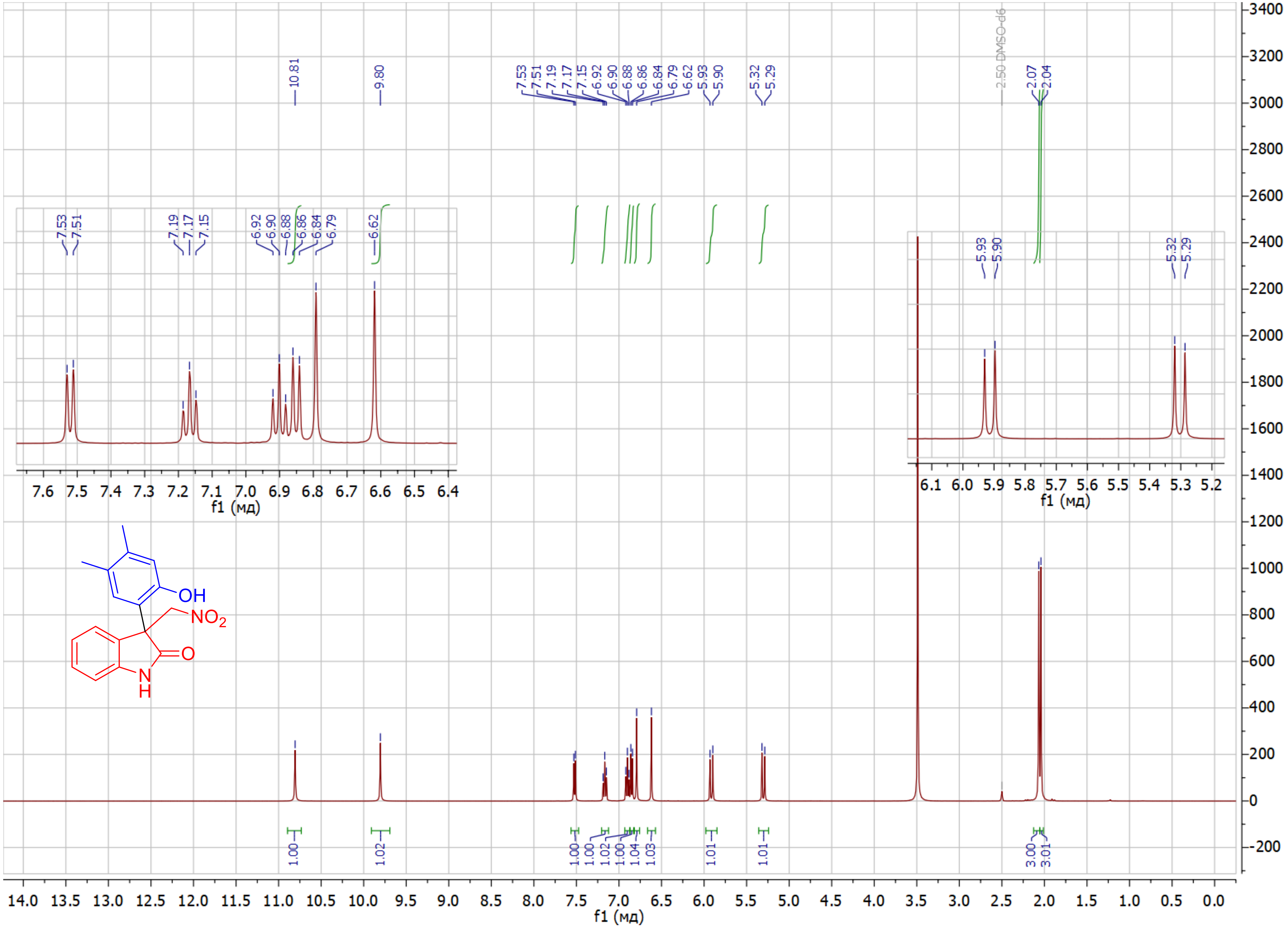

Figure S5. ^{1}H NMR spectrum of compound **7ac** in DMSO-d_6 (400 MHz)

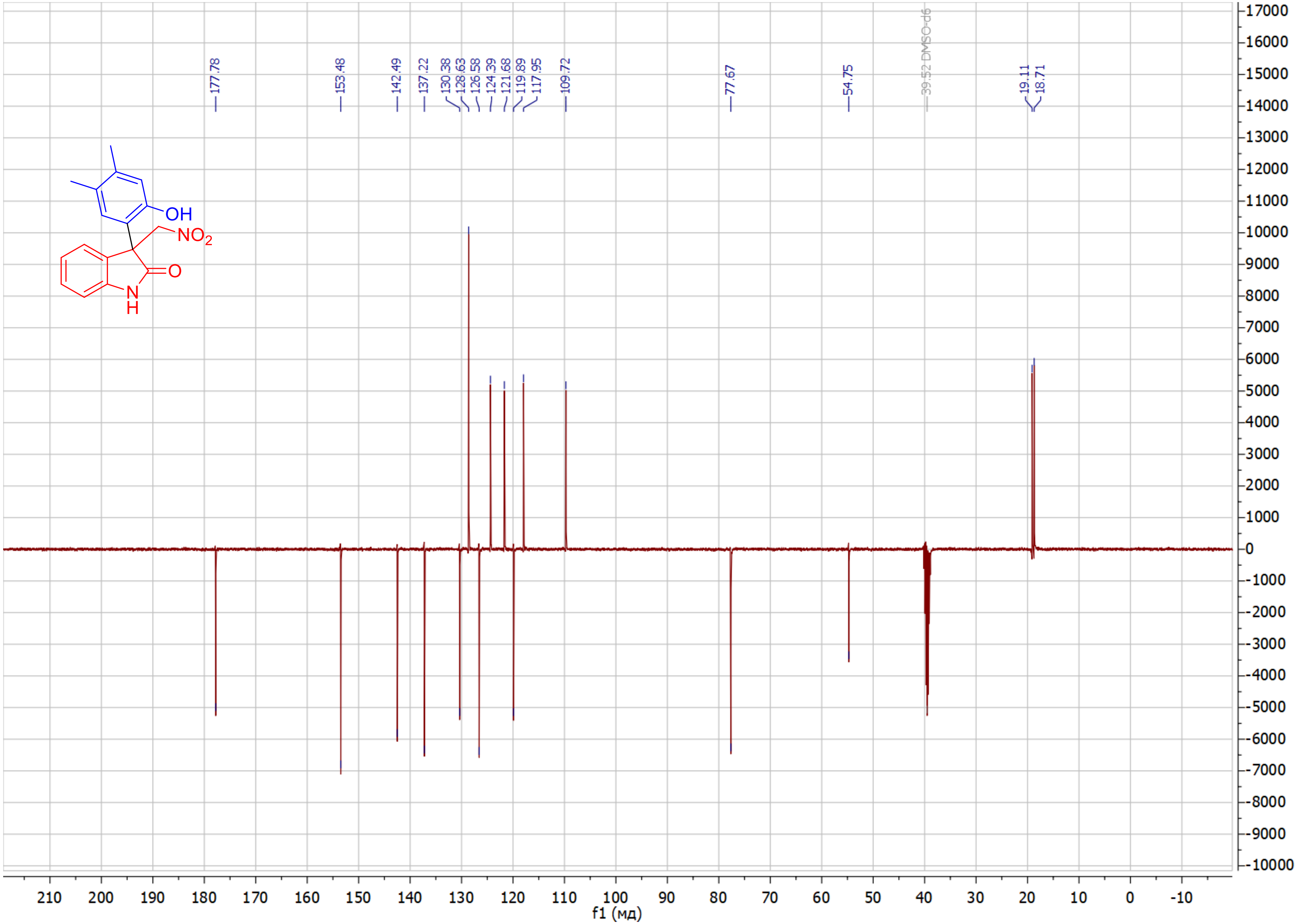

Figure S6. ^{13}C DEPTQ {^{1}H} NMR spectrum of compound **7ac** in DMSO-d_6 (101 MHz)

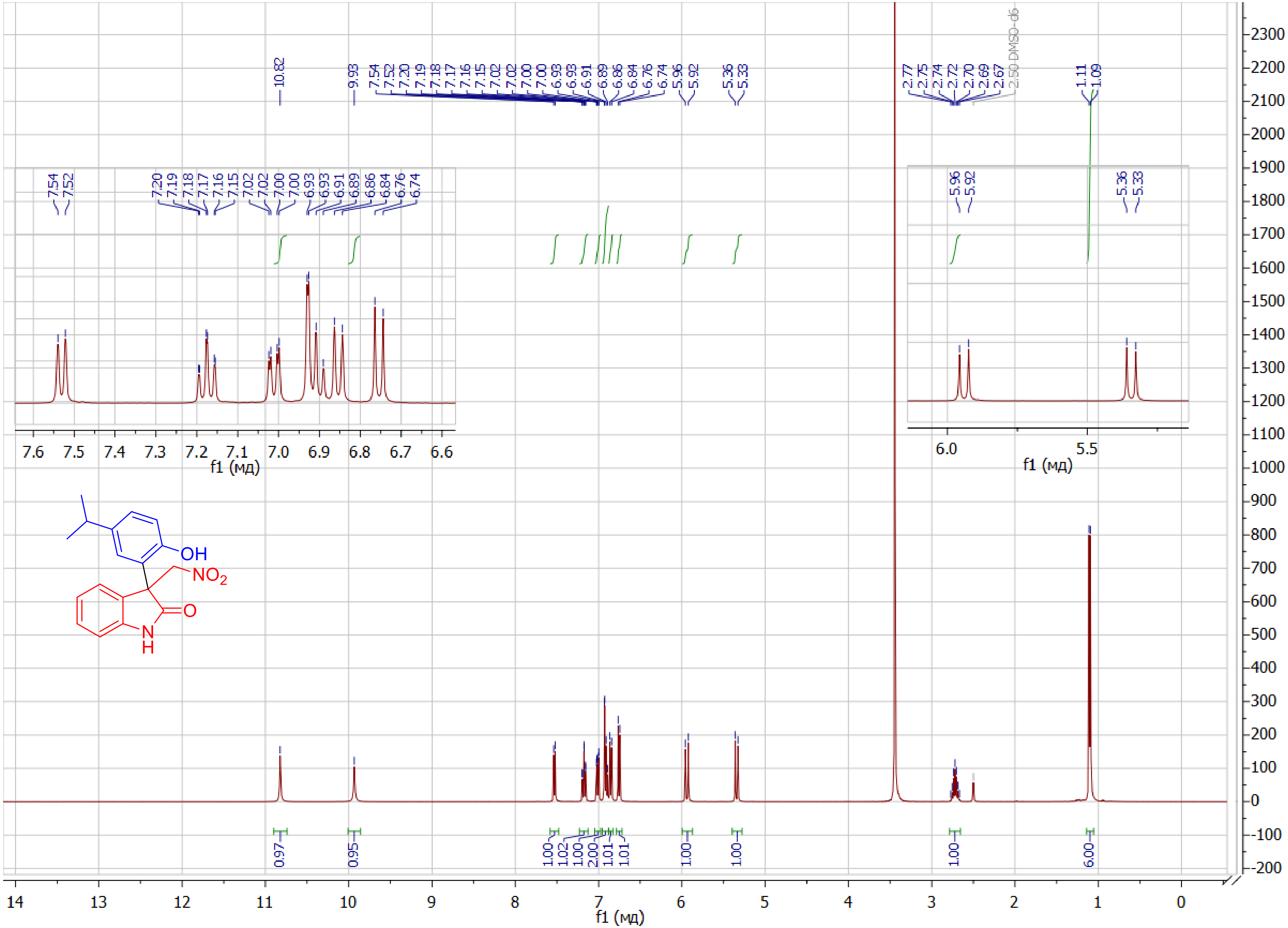

Figure S7. ^{1}H NMR spectrum of compound **7ad** in DMSO-d_6 (400 MHz)

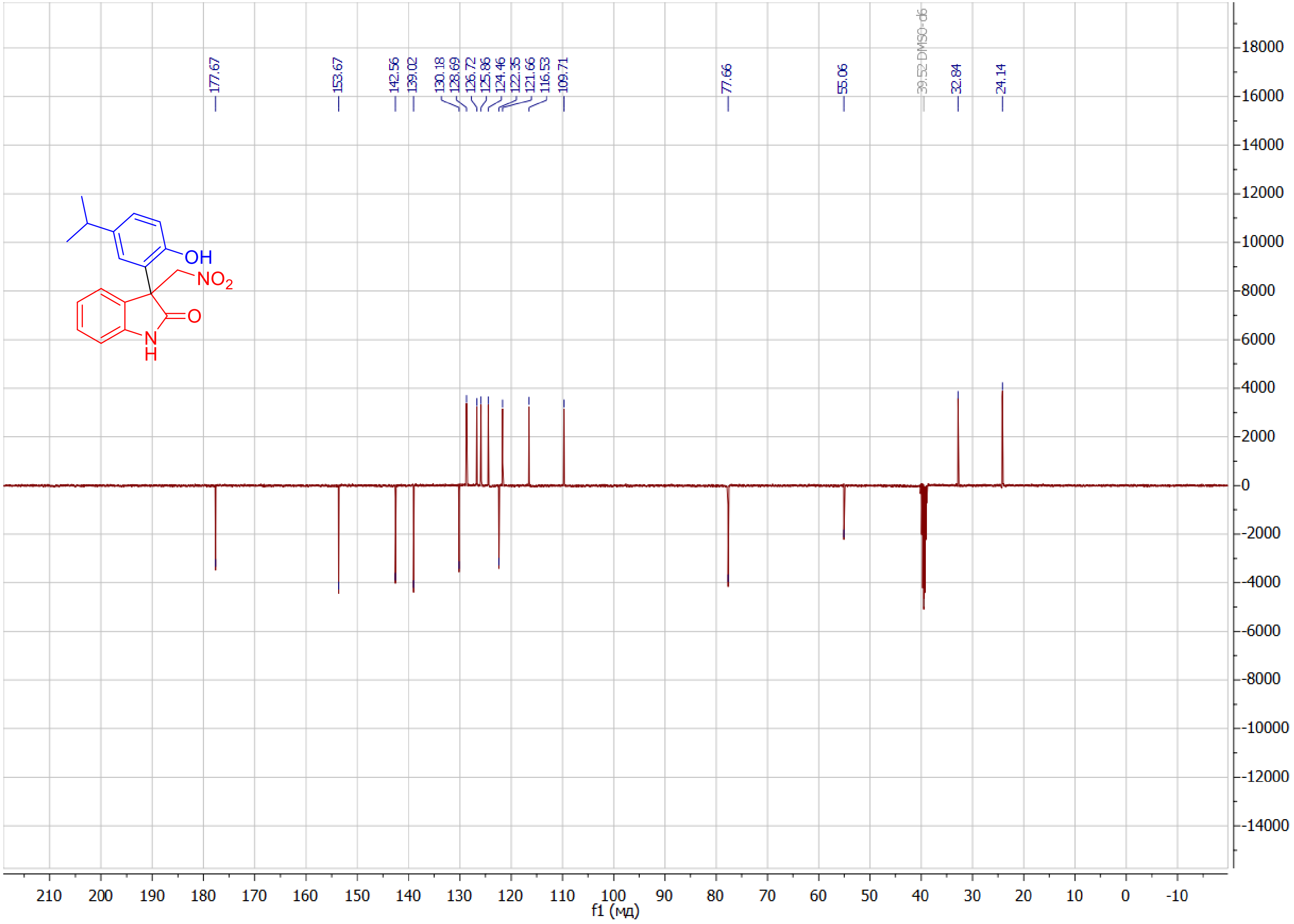

Figure S8. ^{13}C DEPTQ {^{1}H} NMR spectrum of compound **7ad** in DMSO-d_6 (101 MHz)

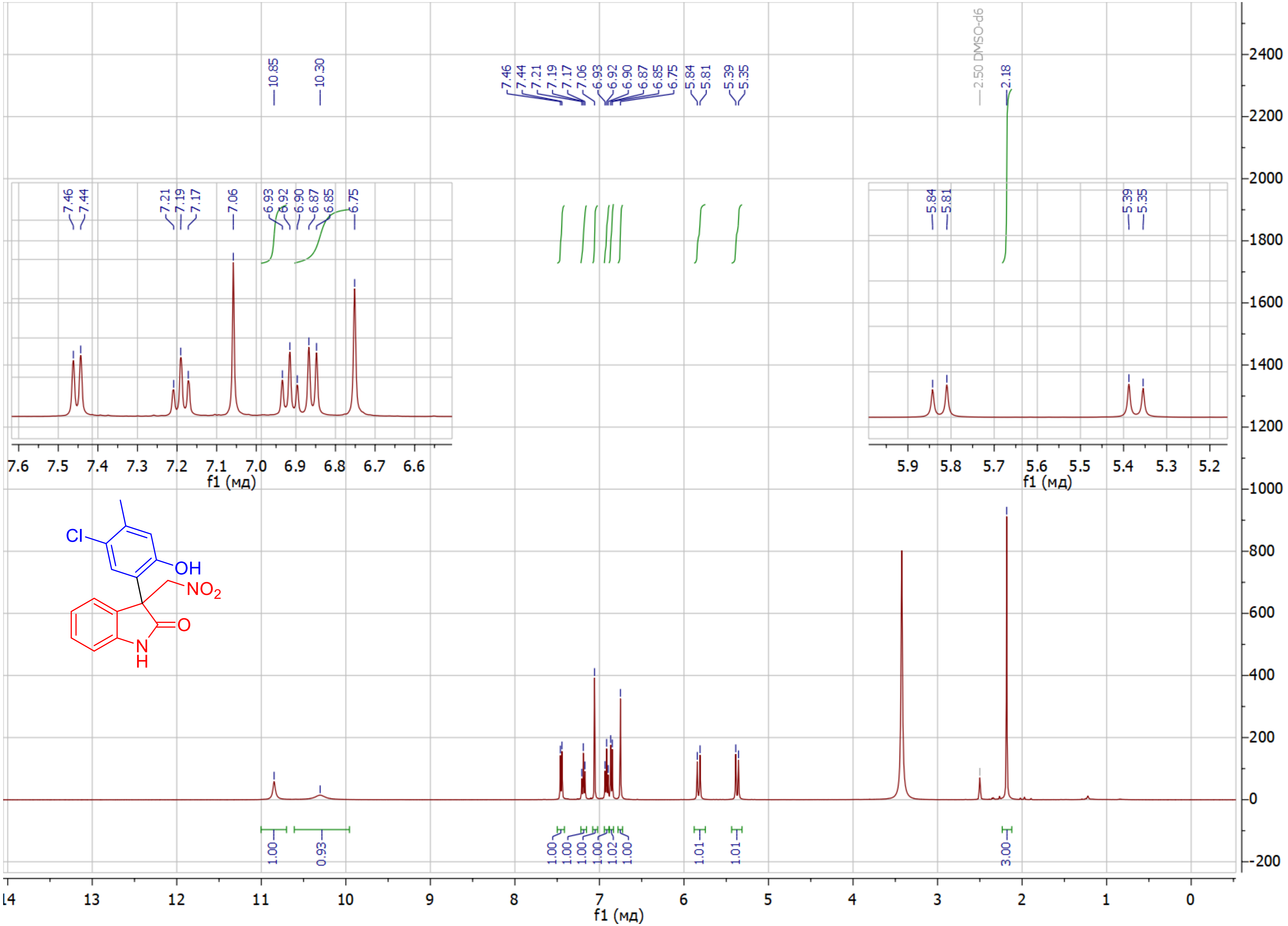

Figure S9. ^{1}H NMR spectrum of compound **7ae** in DMSO-d_6 (400 MHz)

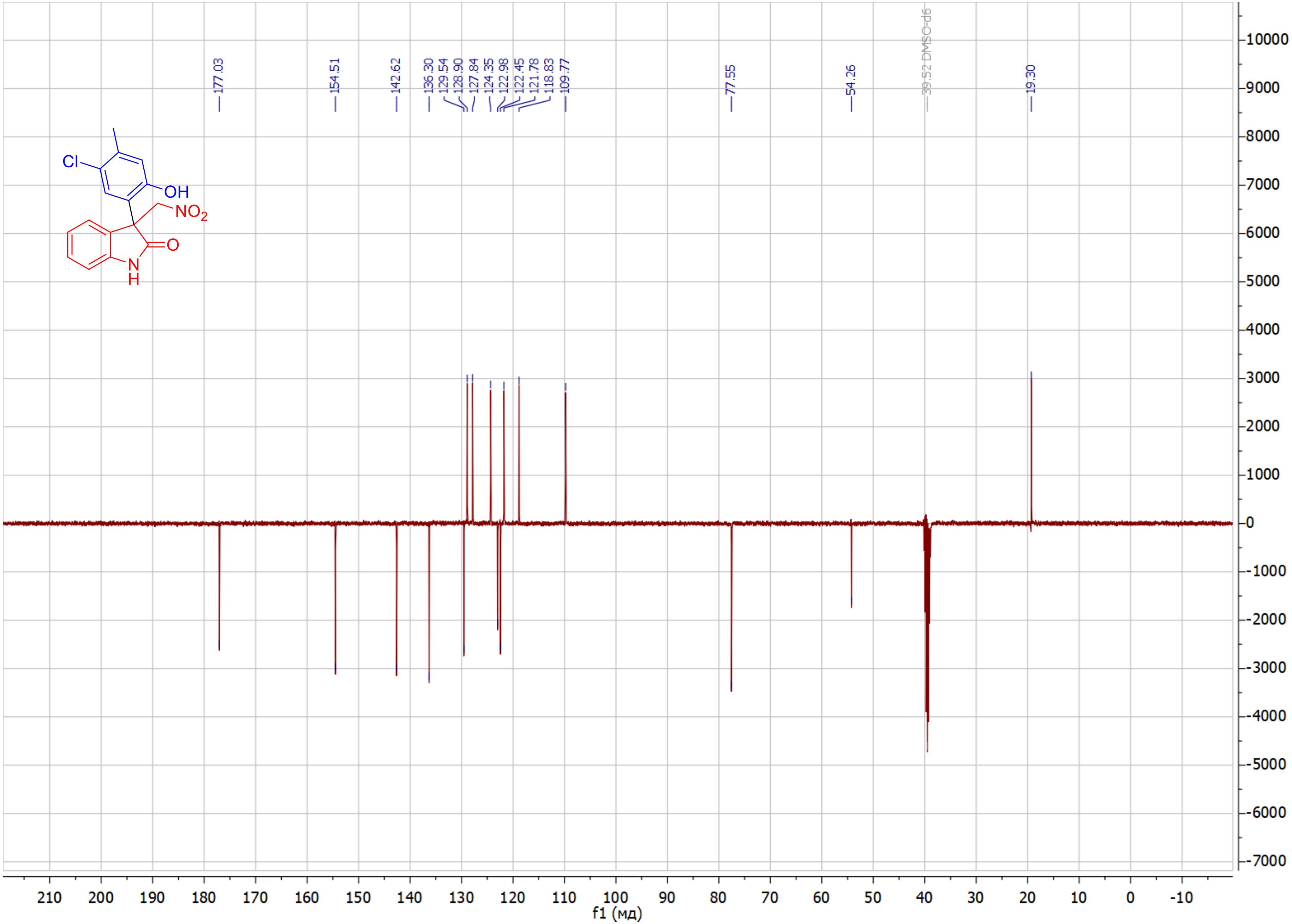

Figure S10. ^{13}C DEPTQ {^{1}H} NMR spectrum of compound **7ae** in DMSO-d_6 (101 MHz)

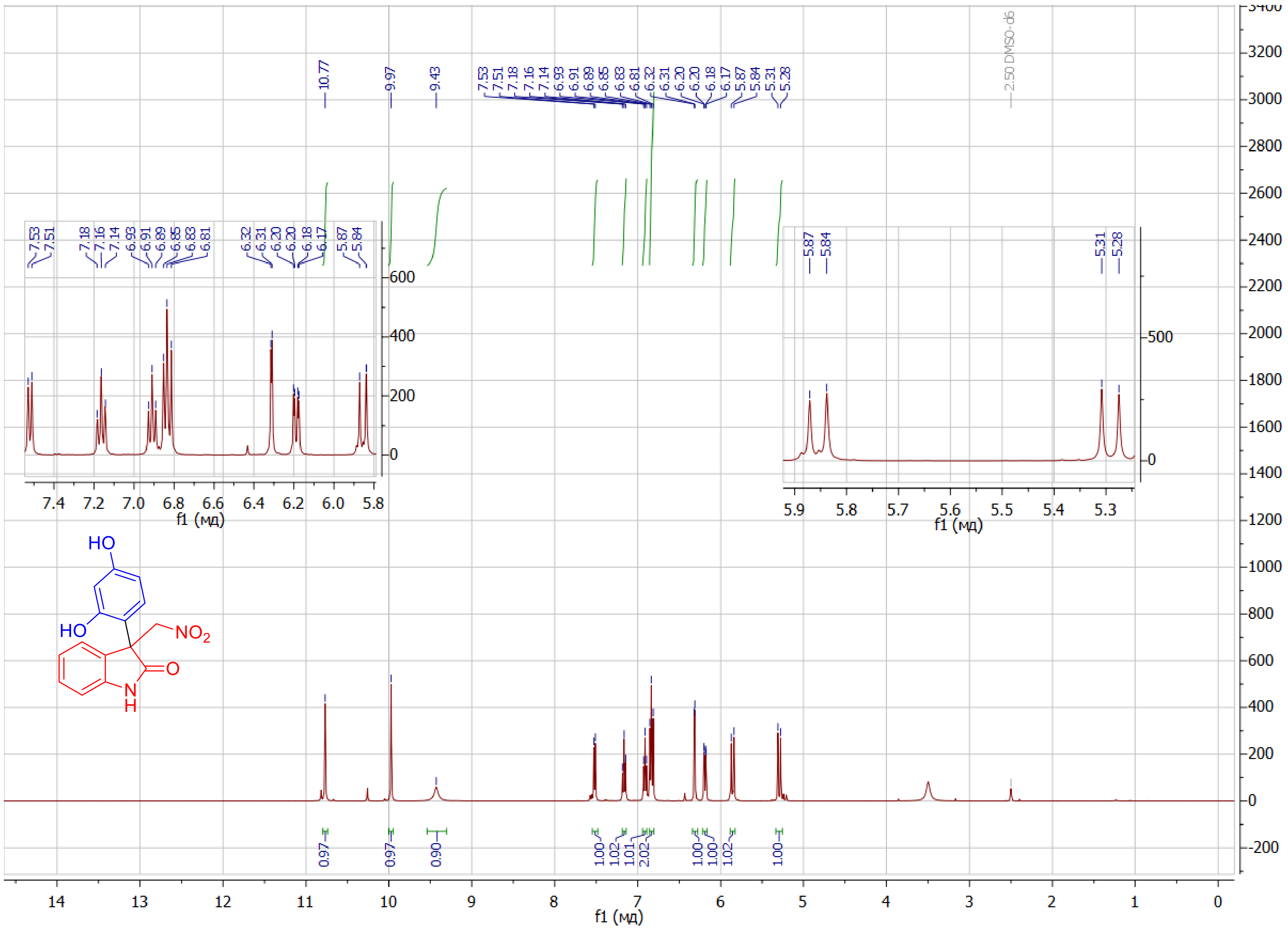

Figure S11. ^{1}H NMR spectrum of compound **7af** in DMSO-d_6 (400 MHz)

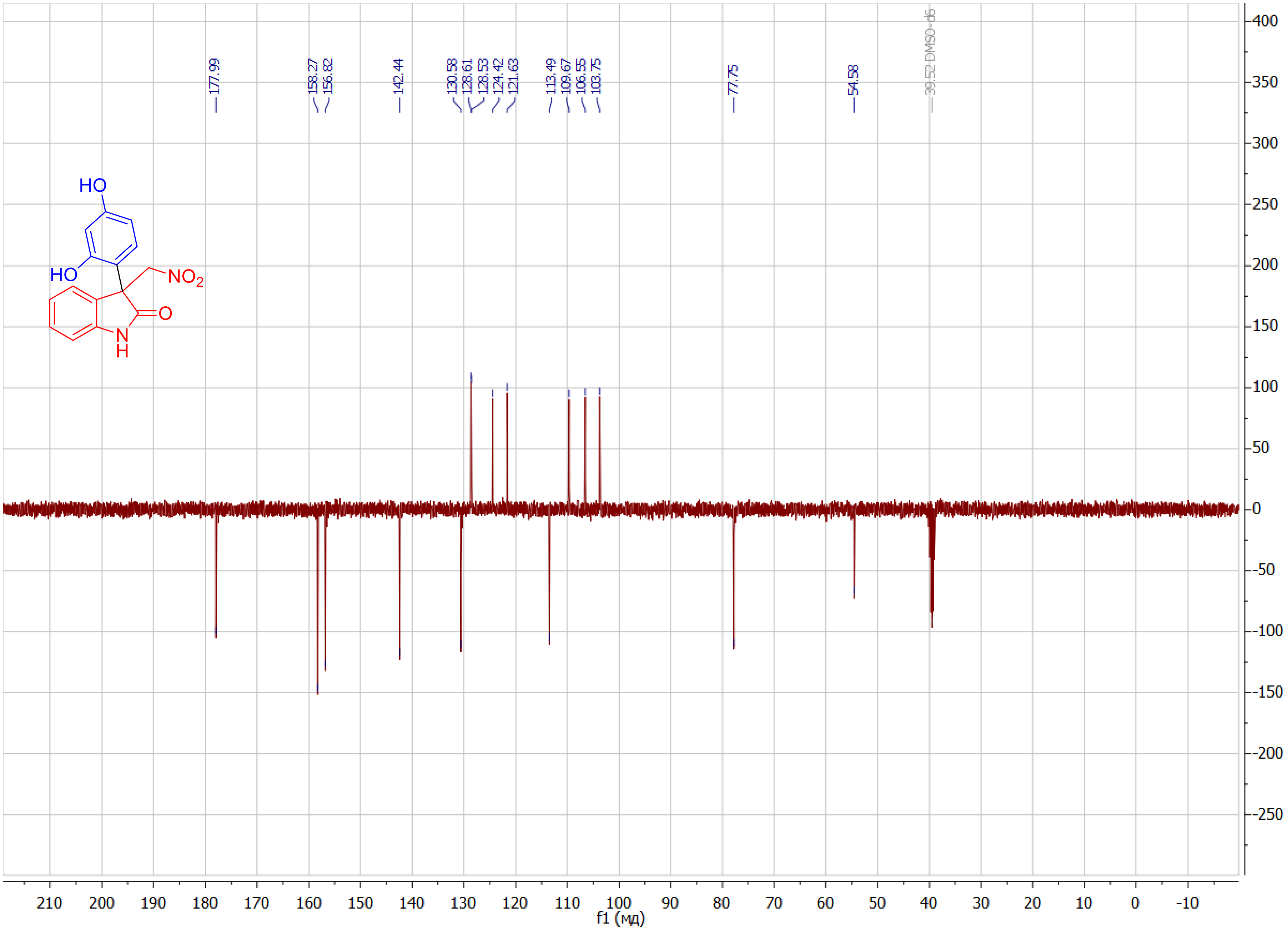

Figure S12. ^{13}C DEPTQ {^{1}H} NMR spectrum of compound **7af** in DMSO-d_6 (101 MHz)

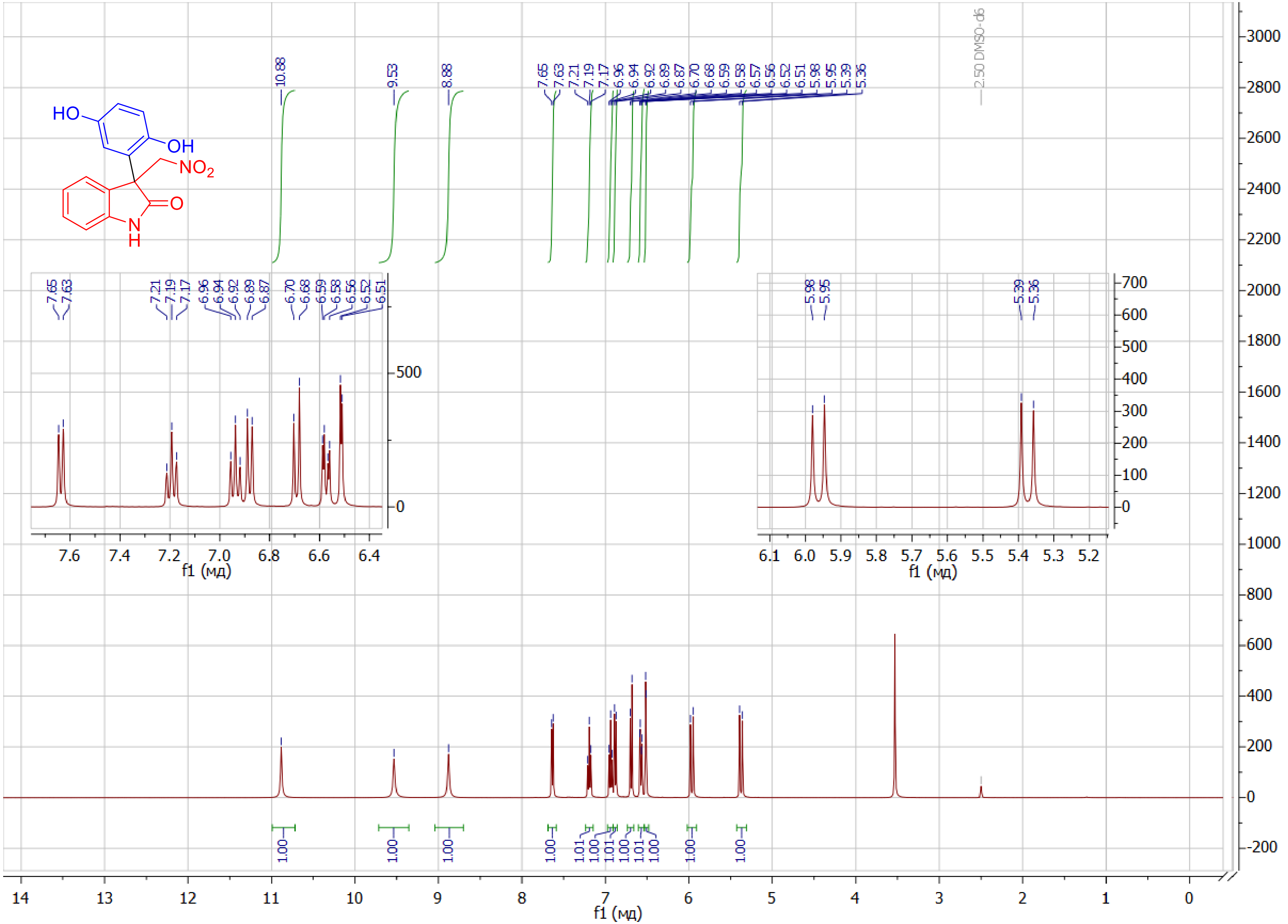

Figure S13. ^{1}H NMR spectrum of compound **7ag** in DMSO-d_6 (400 MHz)

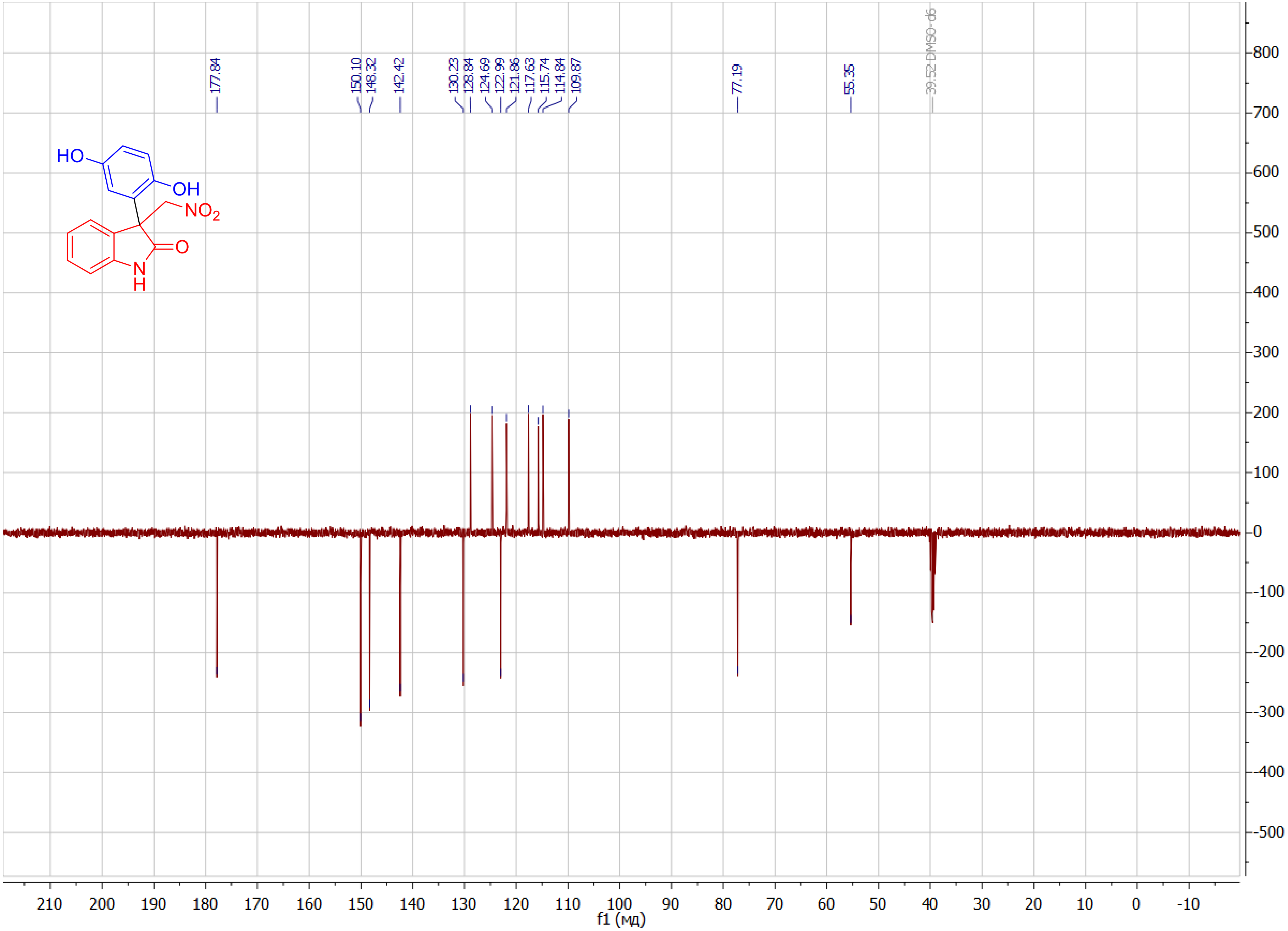

Figure S14. ^{13}C DEPTQ {^{1}H} NMR spectrum of compound **7ag** in DMSO-d_6 (101 MHz)

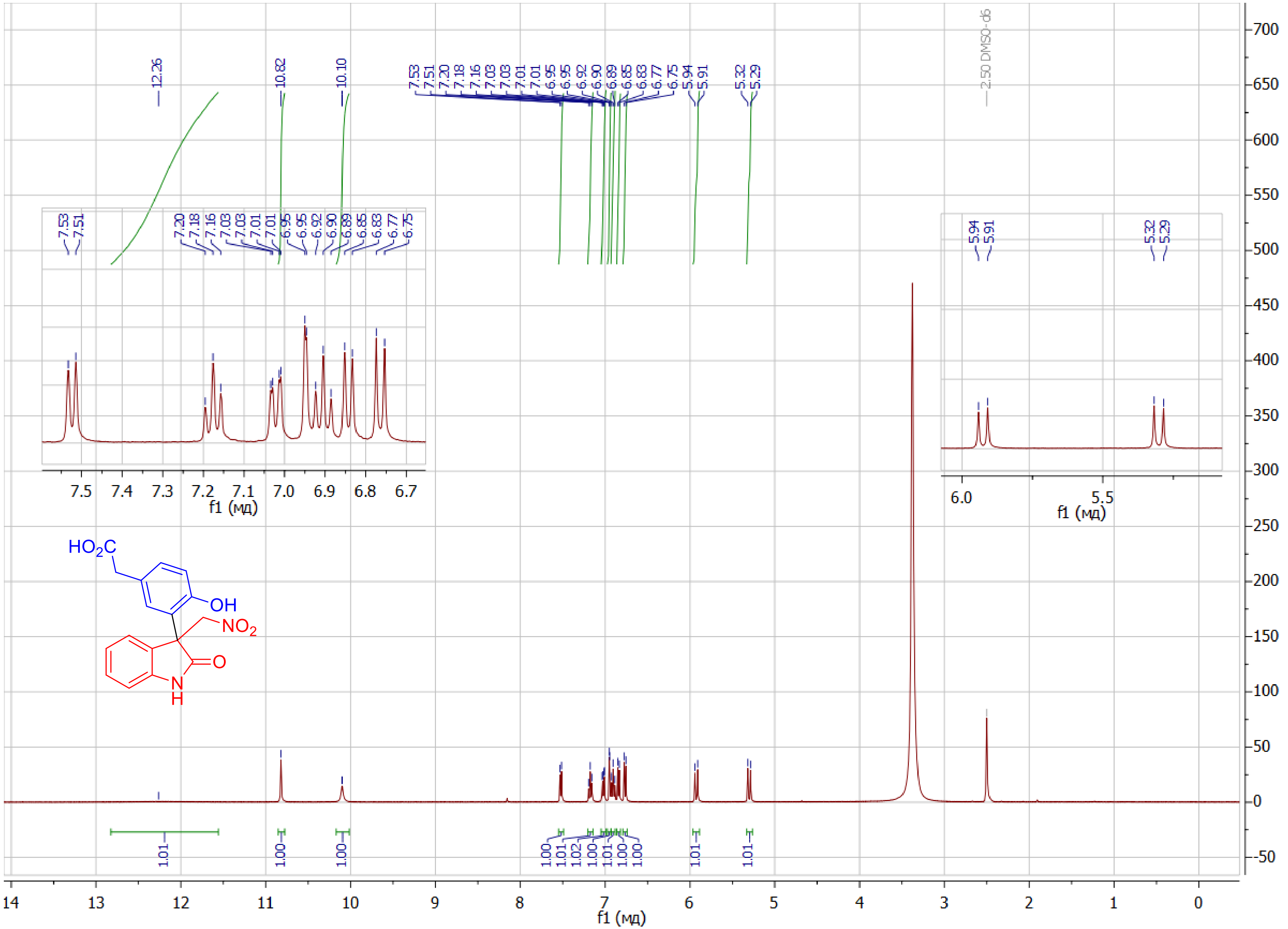

Figure S15. ^{1}H NMR spectrum of compound **7ah** in DMSO-d_6 (400 MHz)

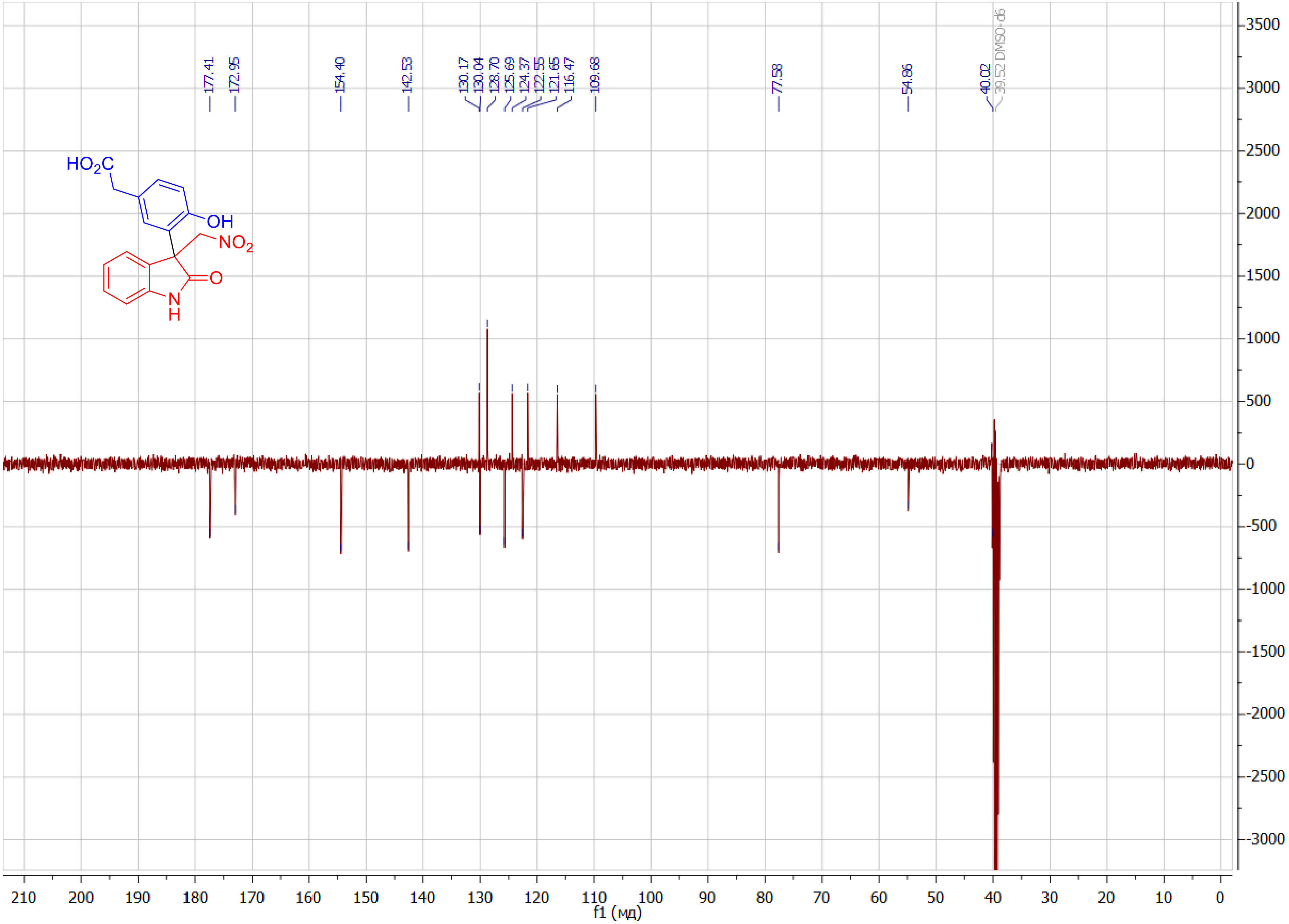

Figure S16. ^{13}C DEPTQ {^{1}H} NMR spectrum of compound **7ah** in DMSO-d_6 (101 MHz)

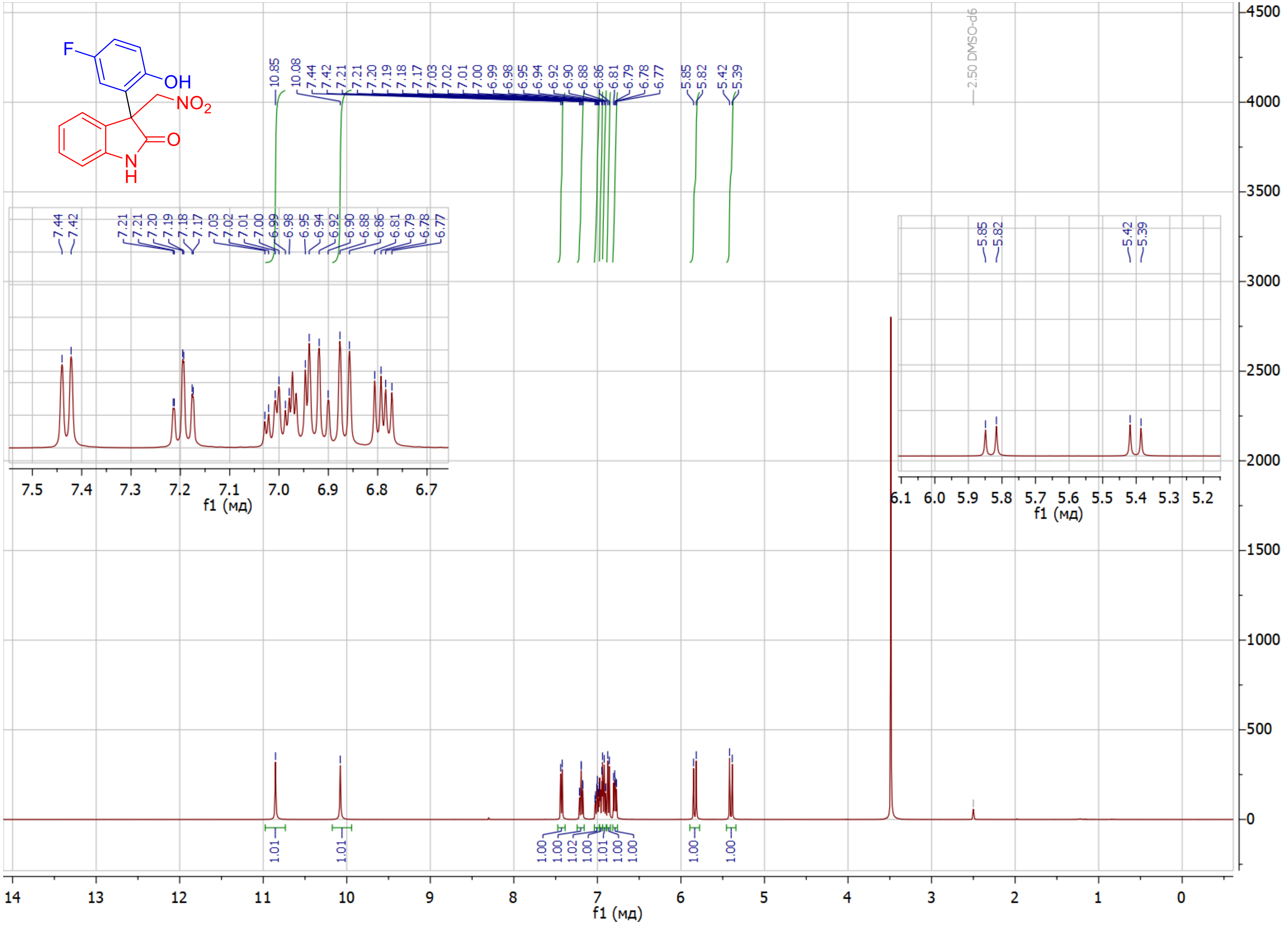

Figure S17. ^{1}H NMR spectrum of compound **7ai** in DMSO-d_6 (400 MHz)

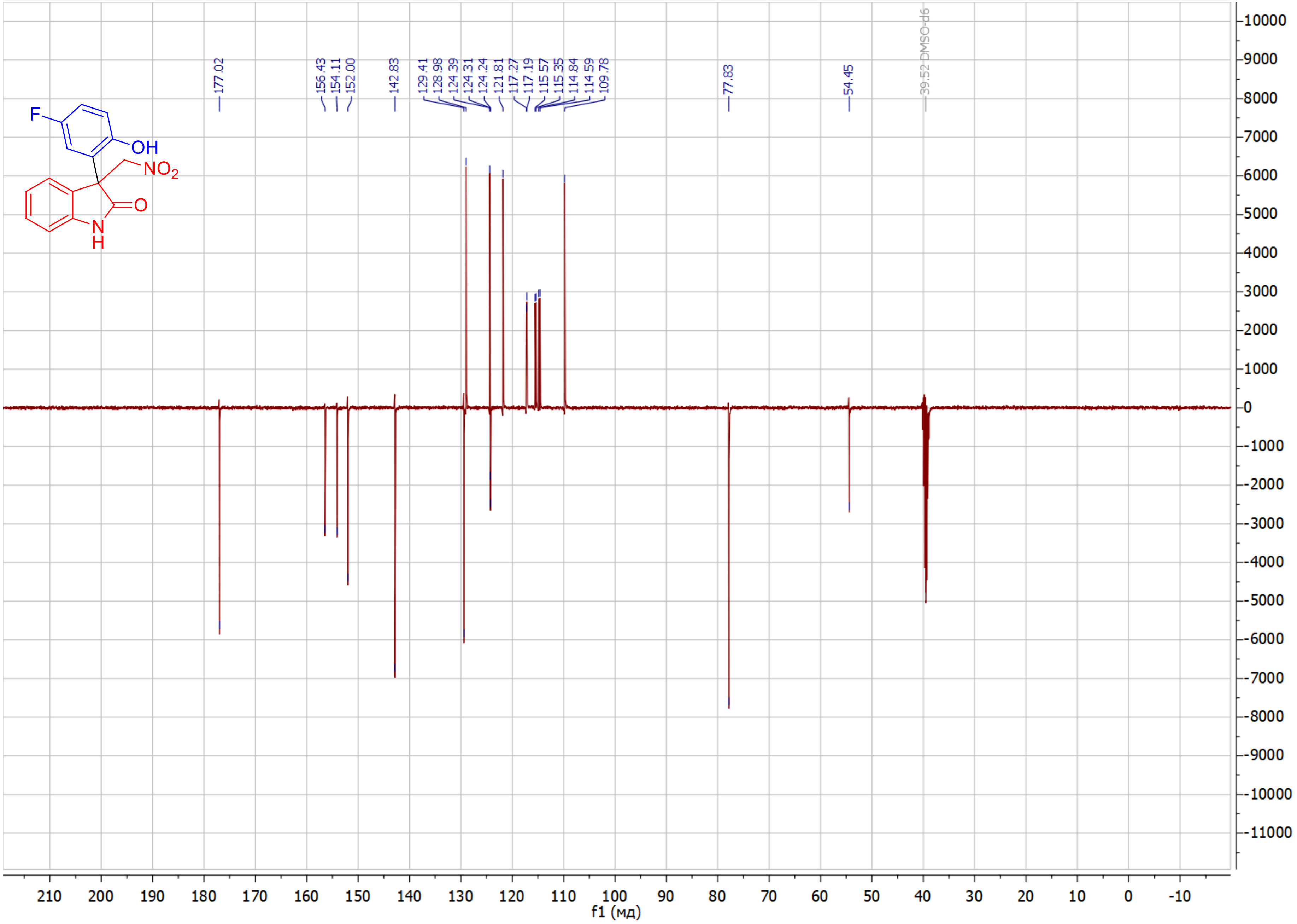

Figure S18. ^{13}C DEPTQ {^{1}H} NMR spectrum of compound **7ai** in DMSO-d_6 (101 MHz)

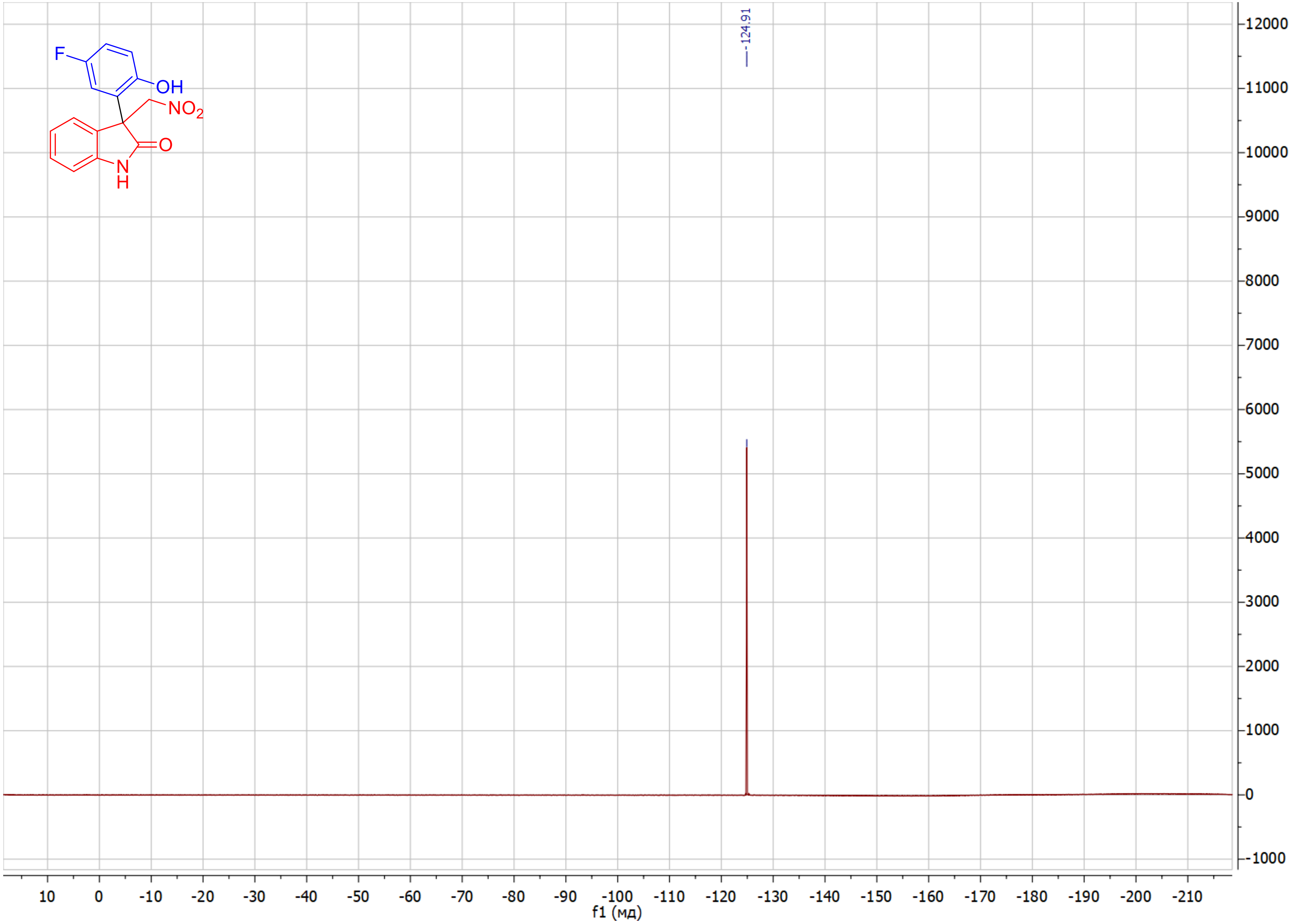

Figure S19. ^{19}F NMR spectrum of compound **7ai** in DMSO-*d*$_6$ (376 MHz)

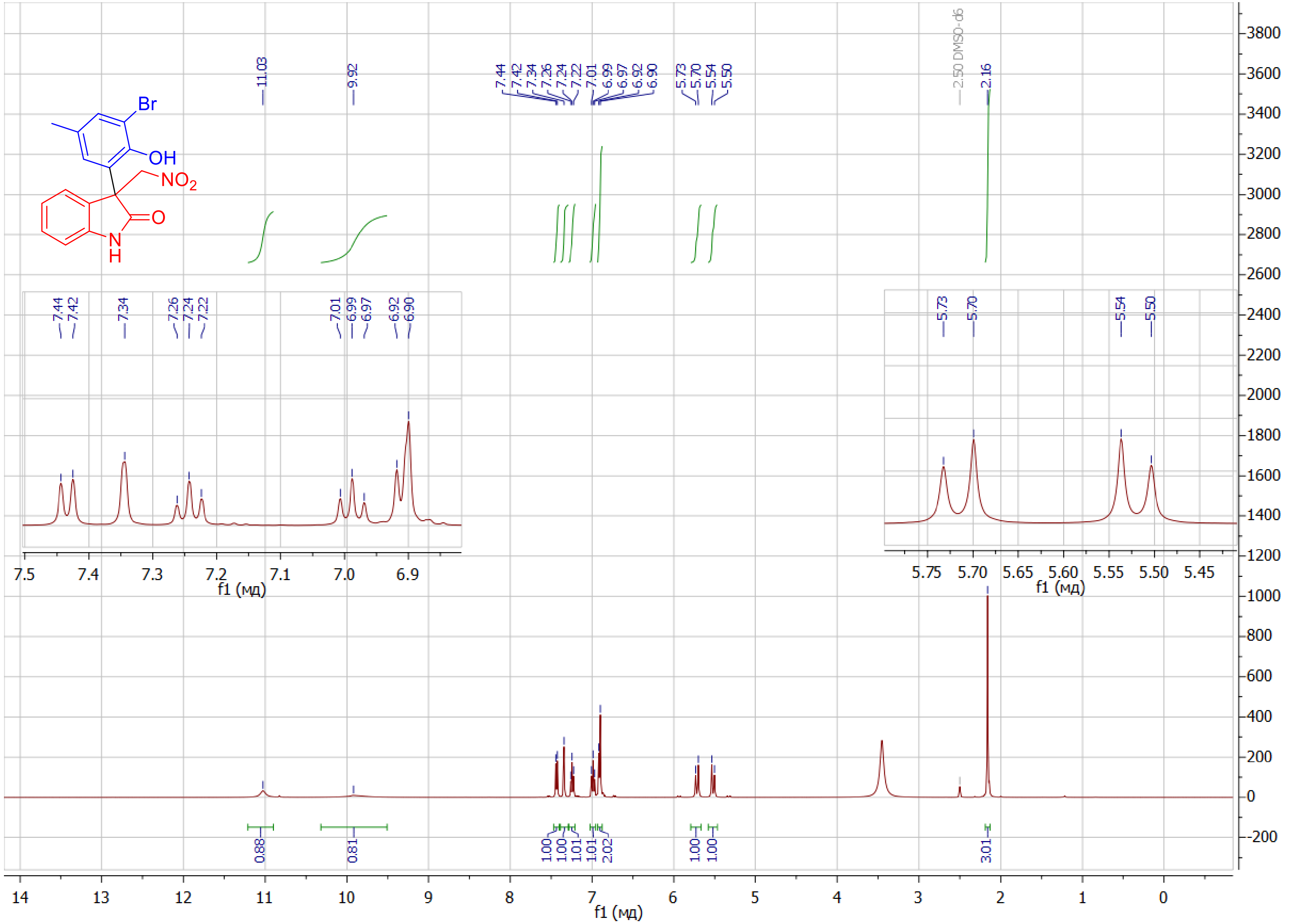

Figure S20. ^{1}H NMR spectrum of compound **7aj** in DMSO-d_6 (400 MHz)

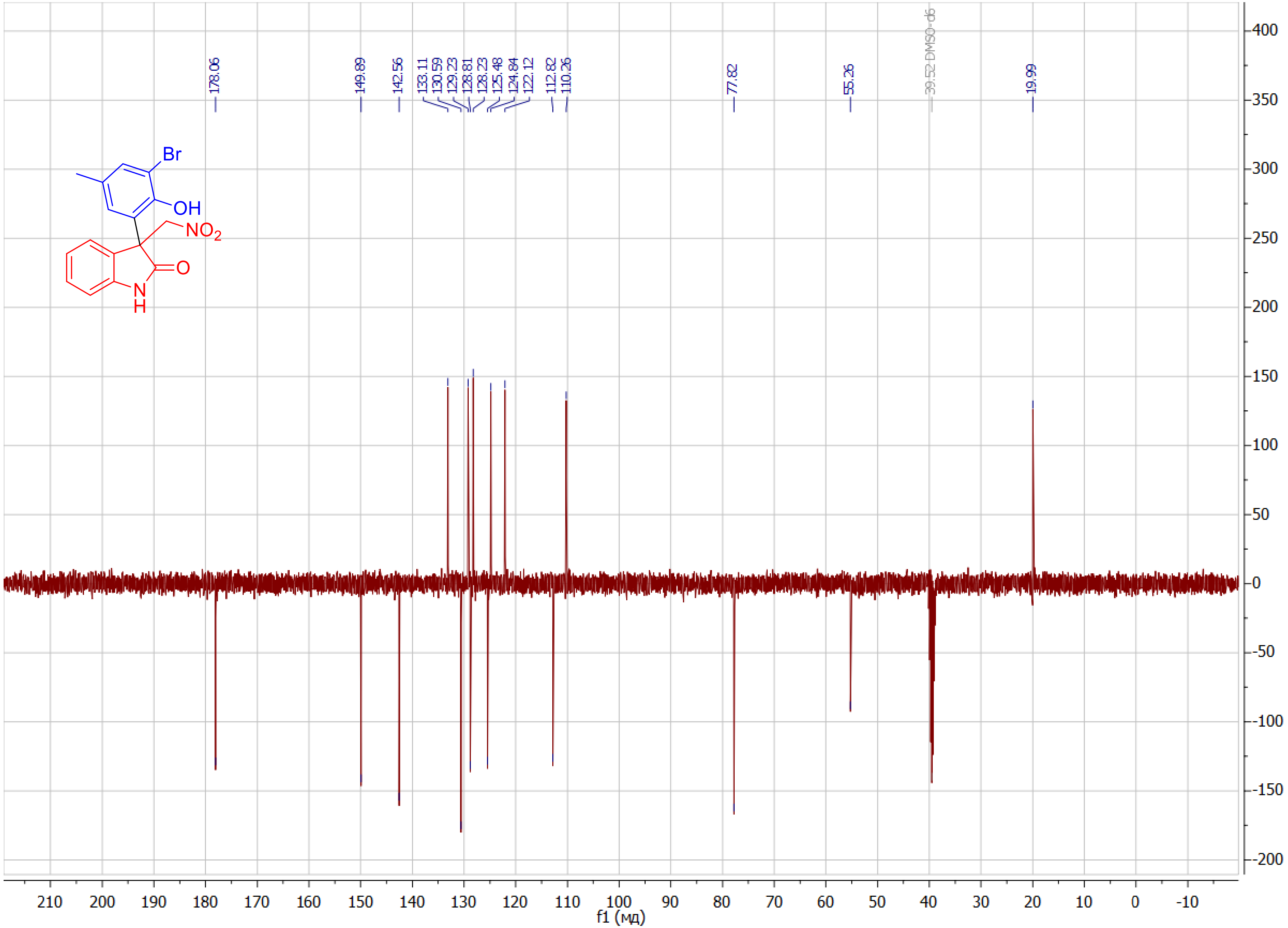

Figure S21. ^{13}C DEPTQ {^{1}H} NMR spectrum of compound **7aj** in DMSO-d_6 (101 MHz)

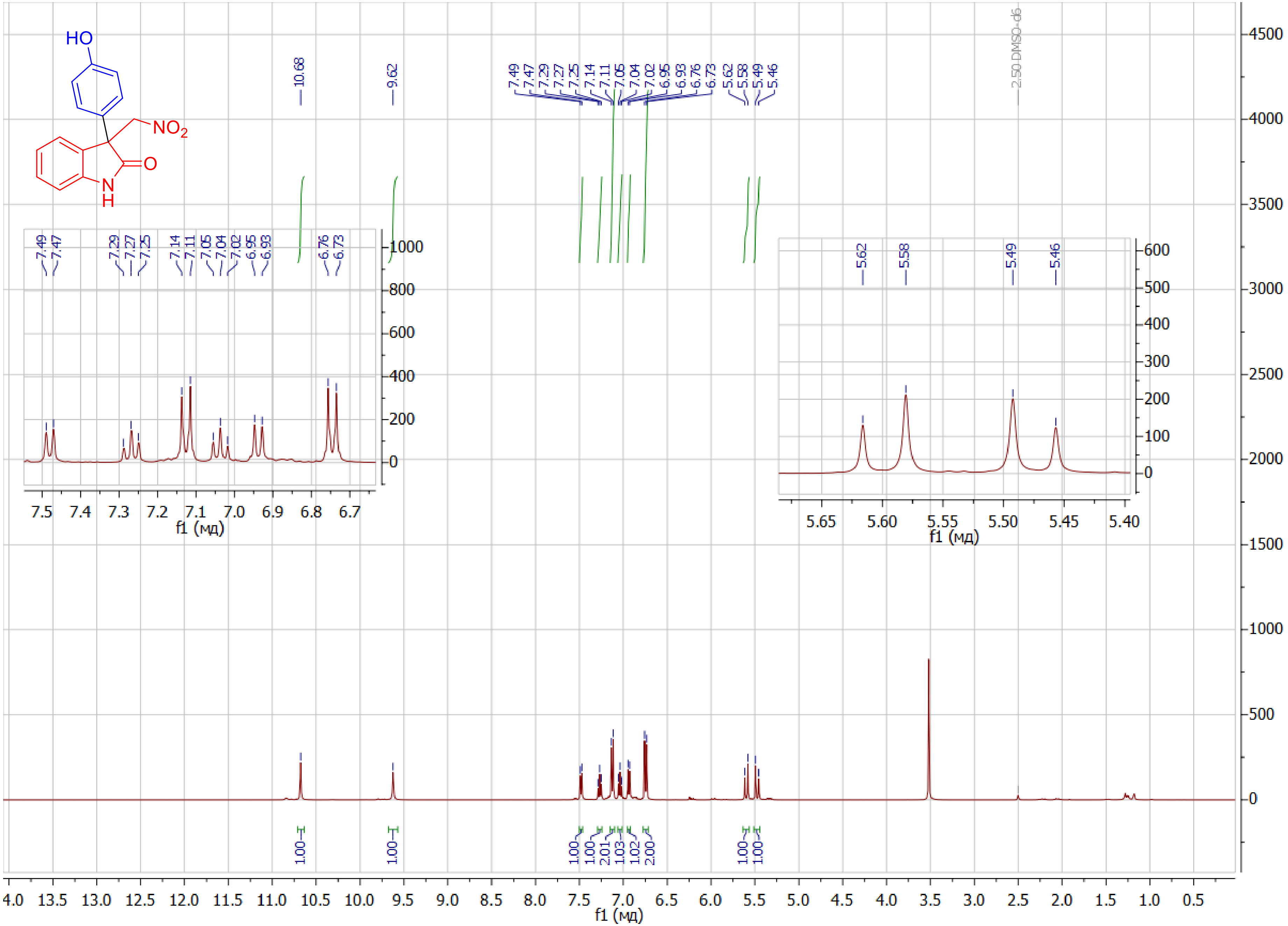

Figure S22. ^{1}H NMR spectrum of compound **7ak** in DMSO-d_6 (400 MHz)

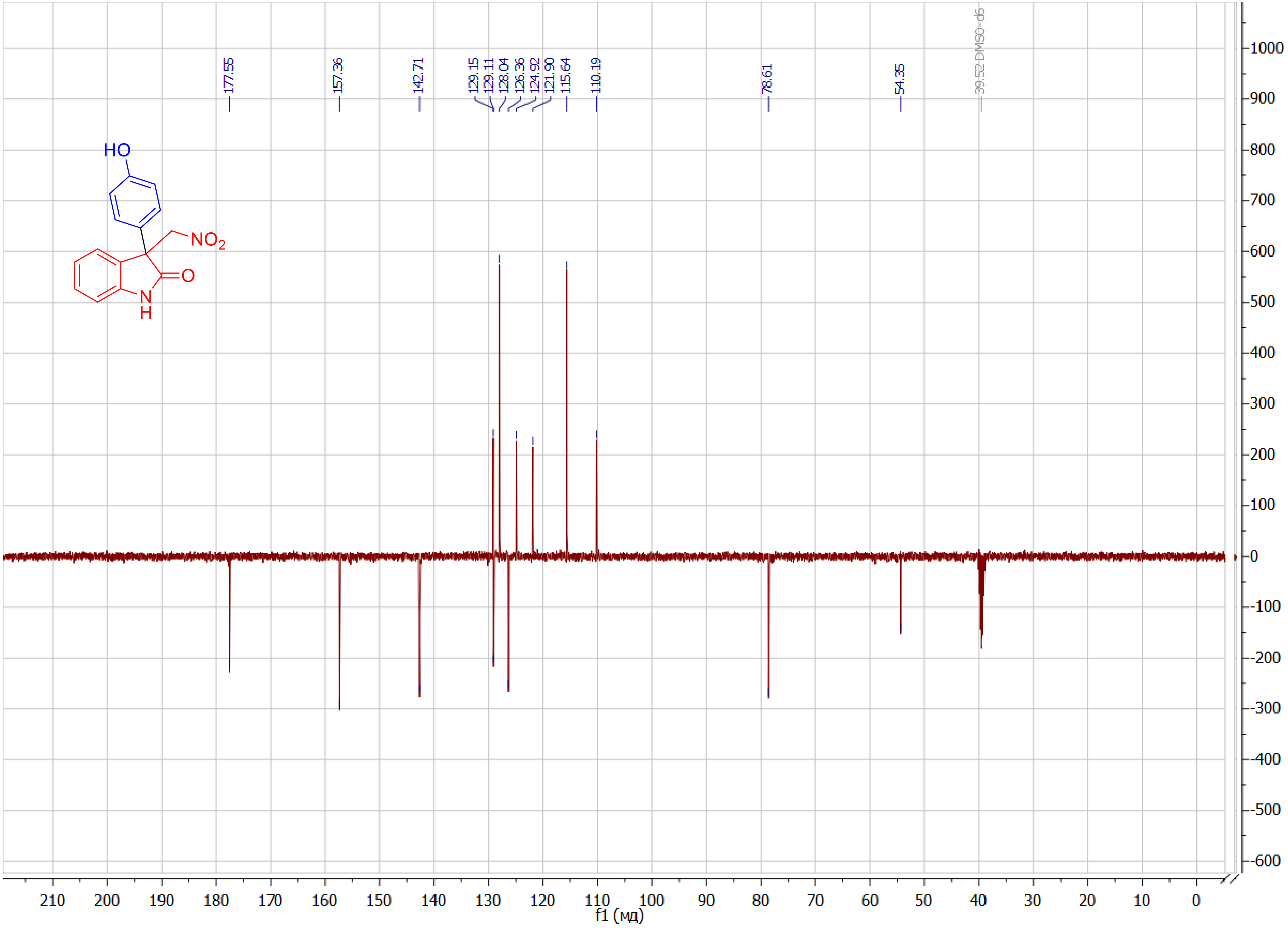

Figure S23. ^{13}C DEPTQ {^{1}H} NMR spectrum of compound **7ak** in DMSO-d_6 (101 MHz)

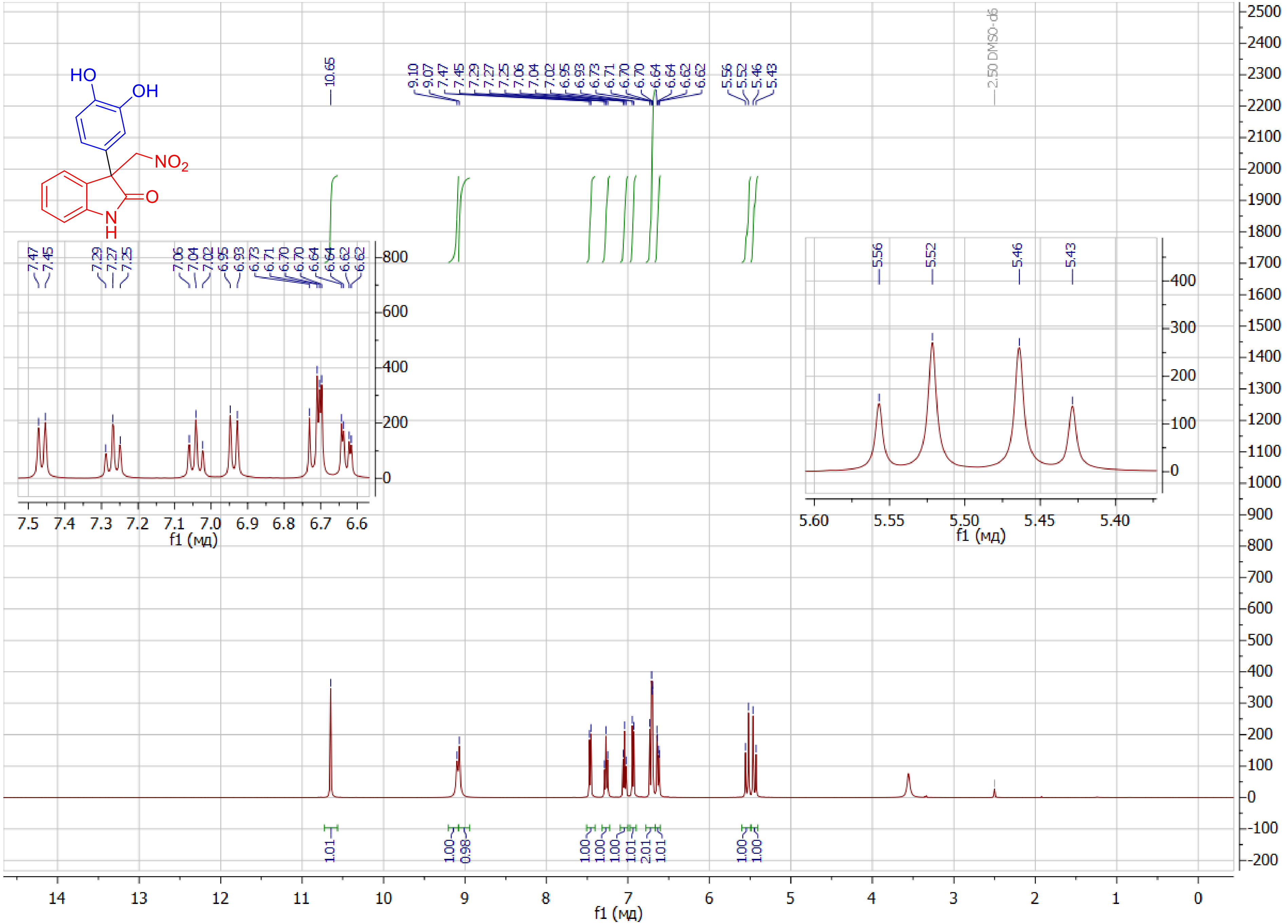

Figure S24. ^{1}H NMR spectrum of compound **7al** in DMSO-d_6 (400 MHz)

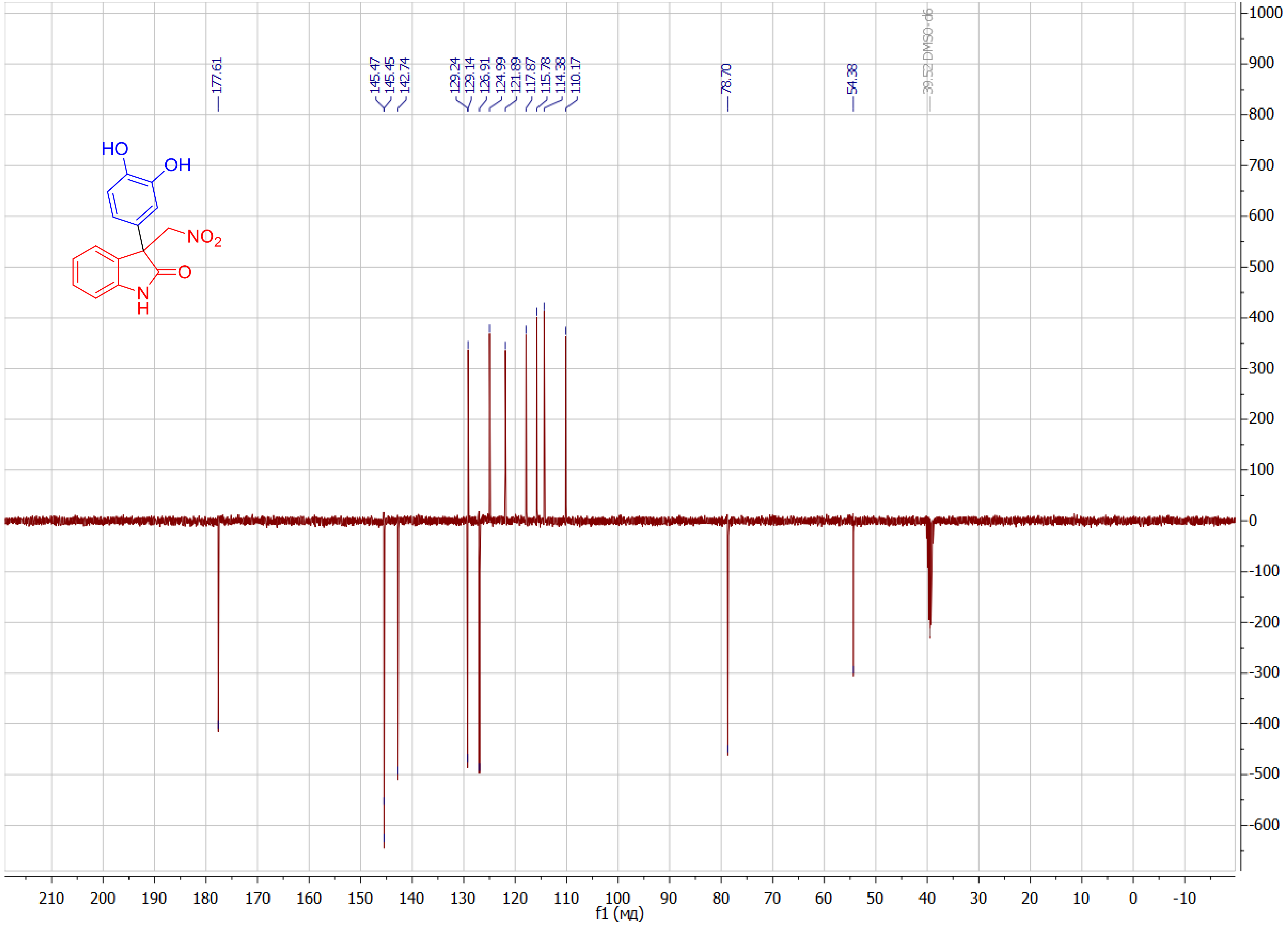

Figure S25. ^{13}C DEPTQ {^{1}H} NMR spectrum of compound **7al** in DMSO-d_6 (101 MHz)

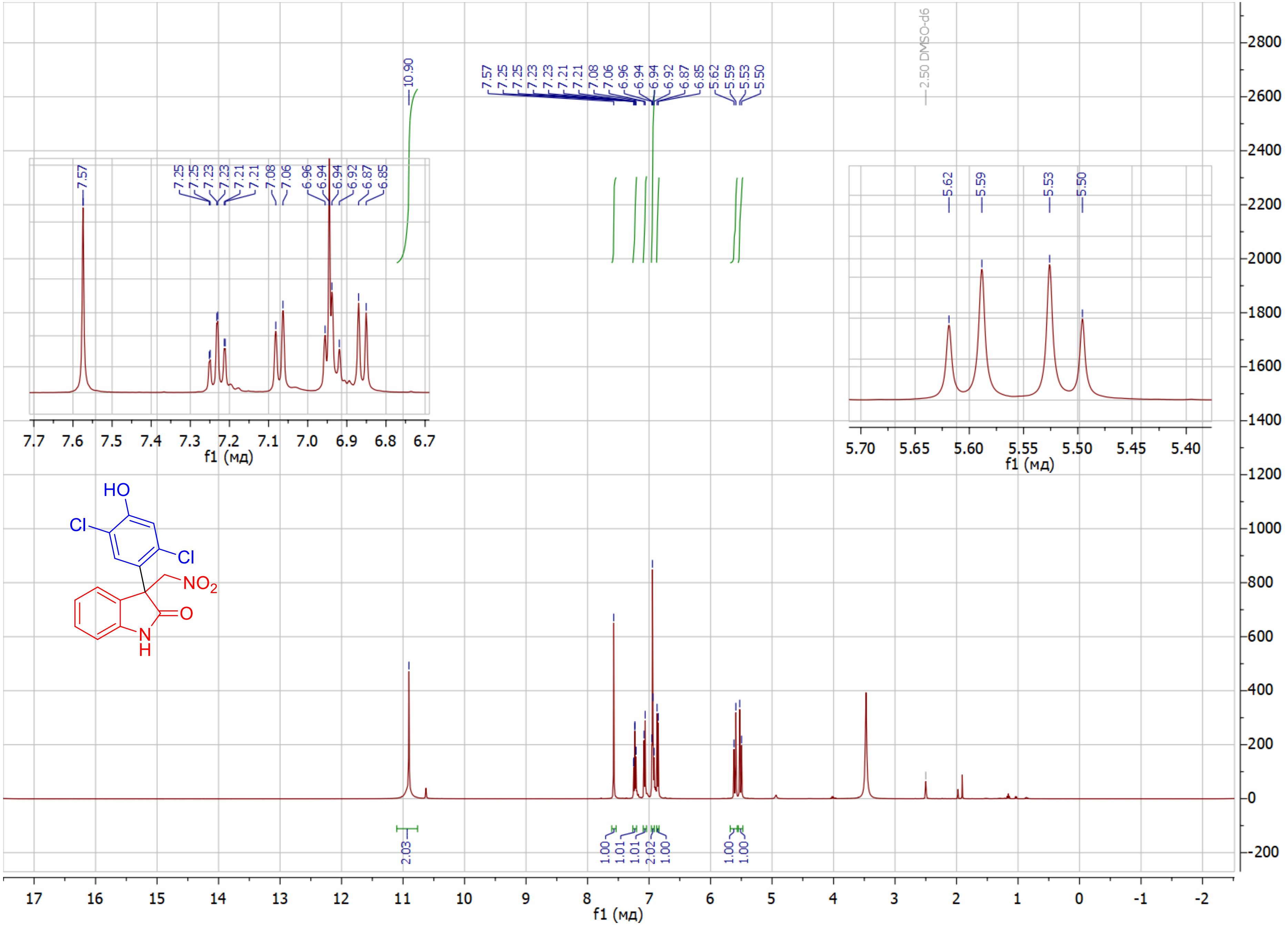

Figure S26. ^{1}H NMR spectrum of compound **7am** in DMSO-d_6 (400 MHz)

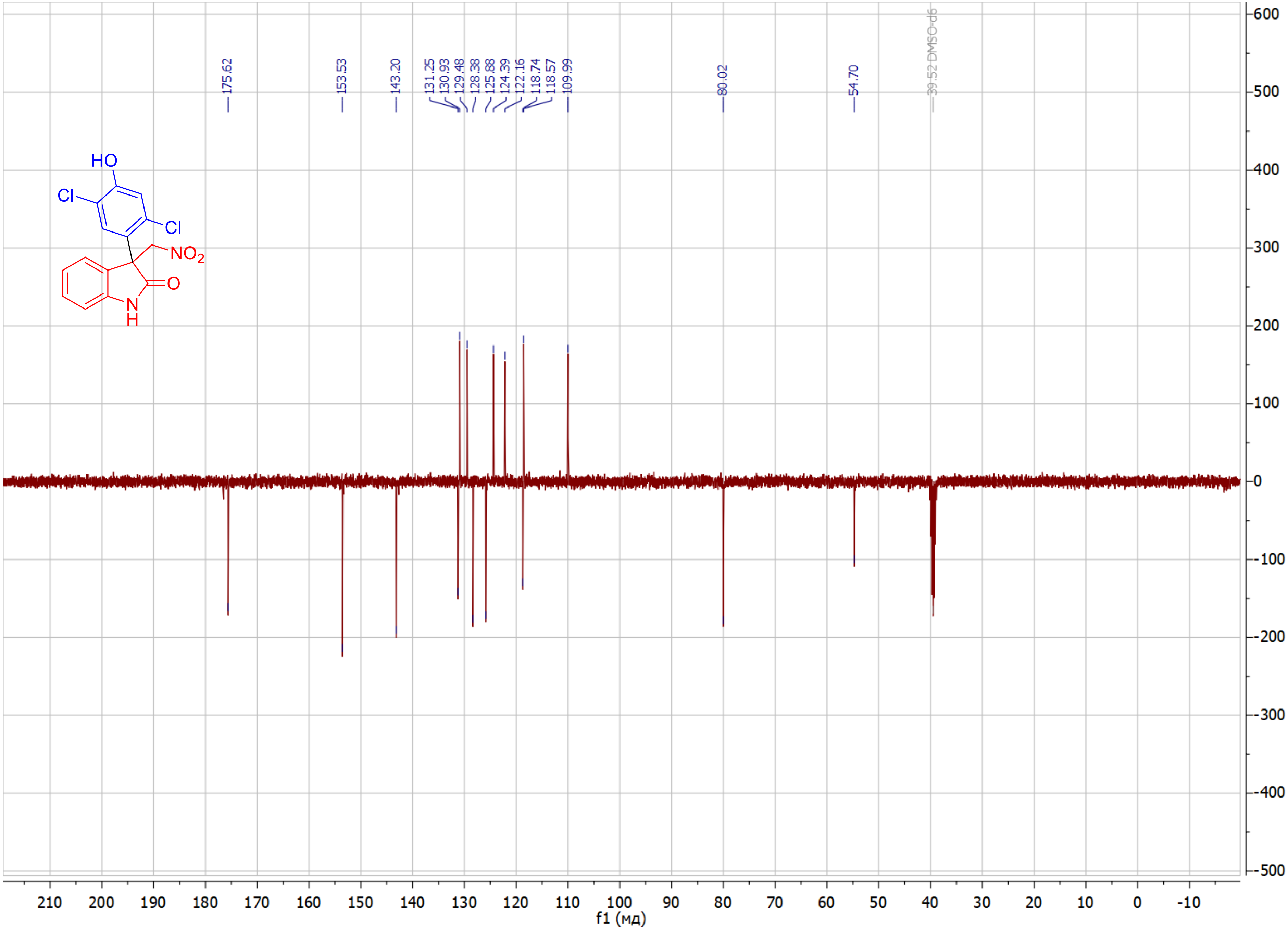

Figure S27. ^{13}C DEPTQ {^{1}H} NMR spectrum of compound **7am** in DMSO-d_6 (101 MHz)

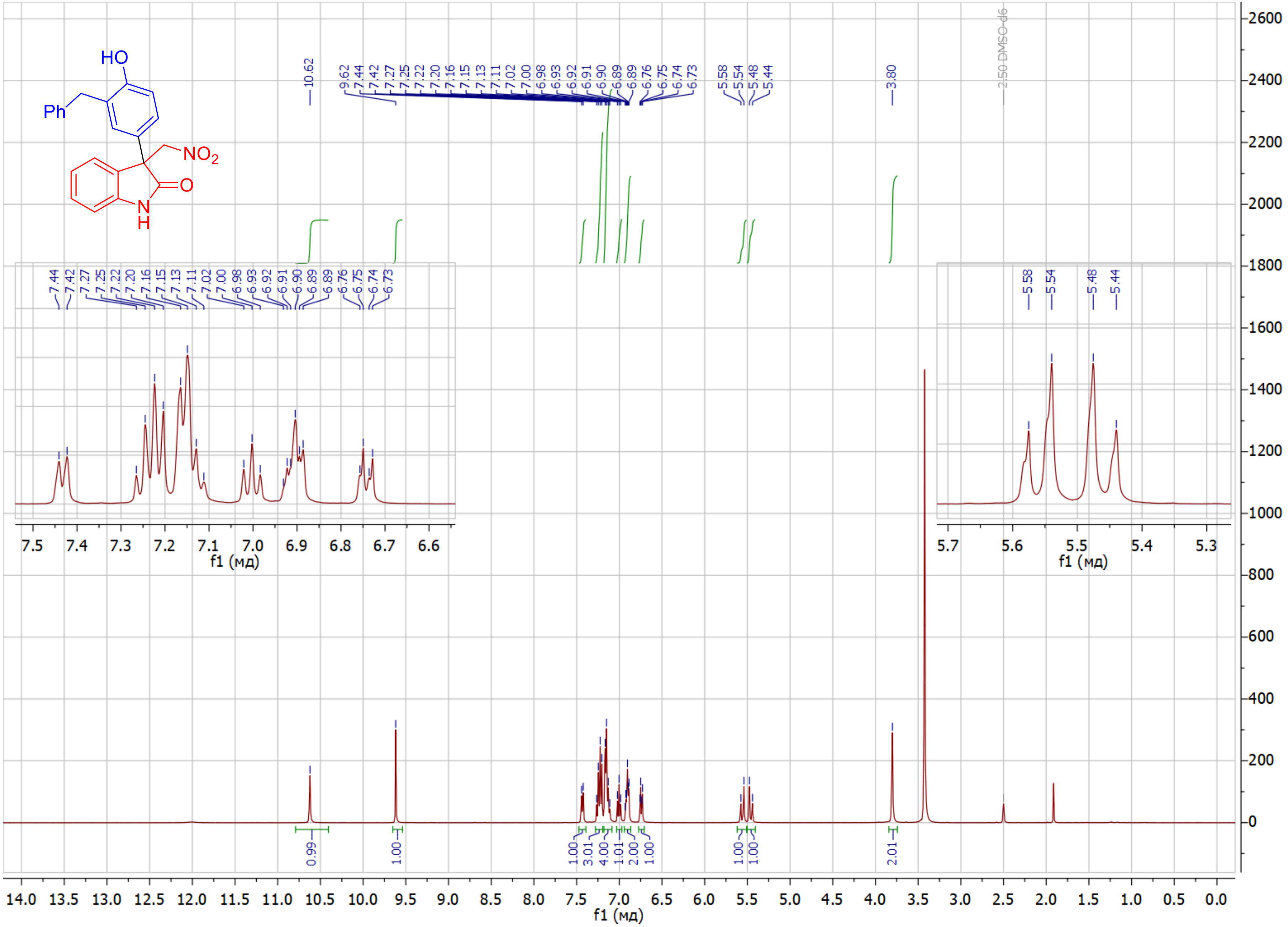

Figure S28. ^{1}H NMR spectrum of compound **7an** in DMSO-d_6 (400 MHz)

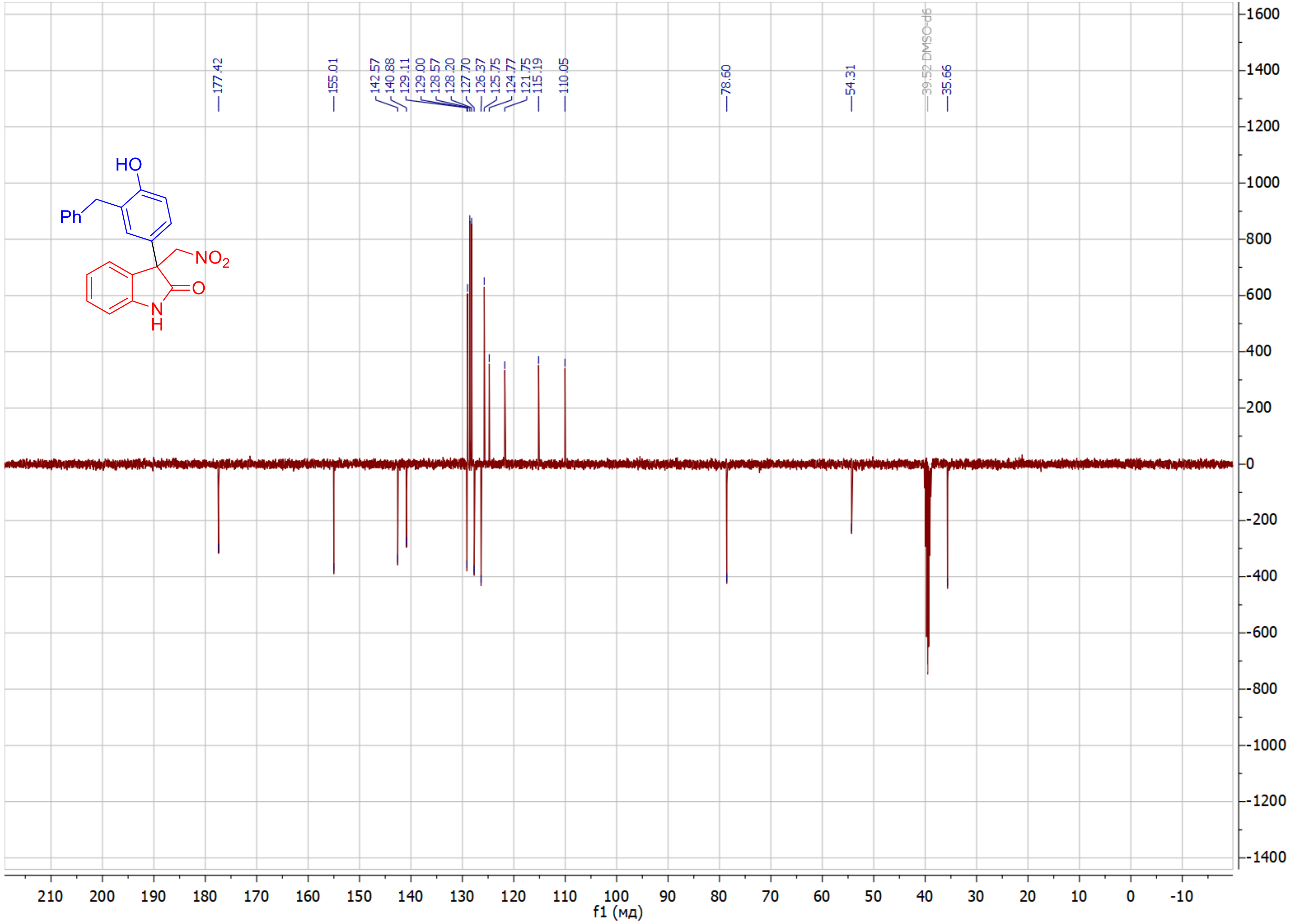

Figure S29. ^{13}C DEPTQ {^{1}H} NMR spectrum of compound **7an** in DMSO-d_6 (101 MHz)

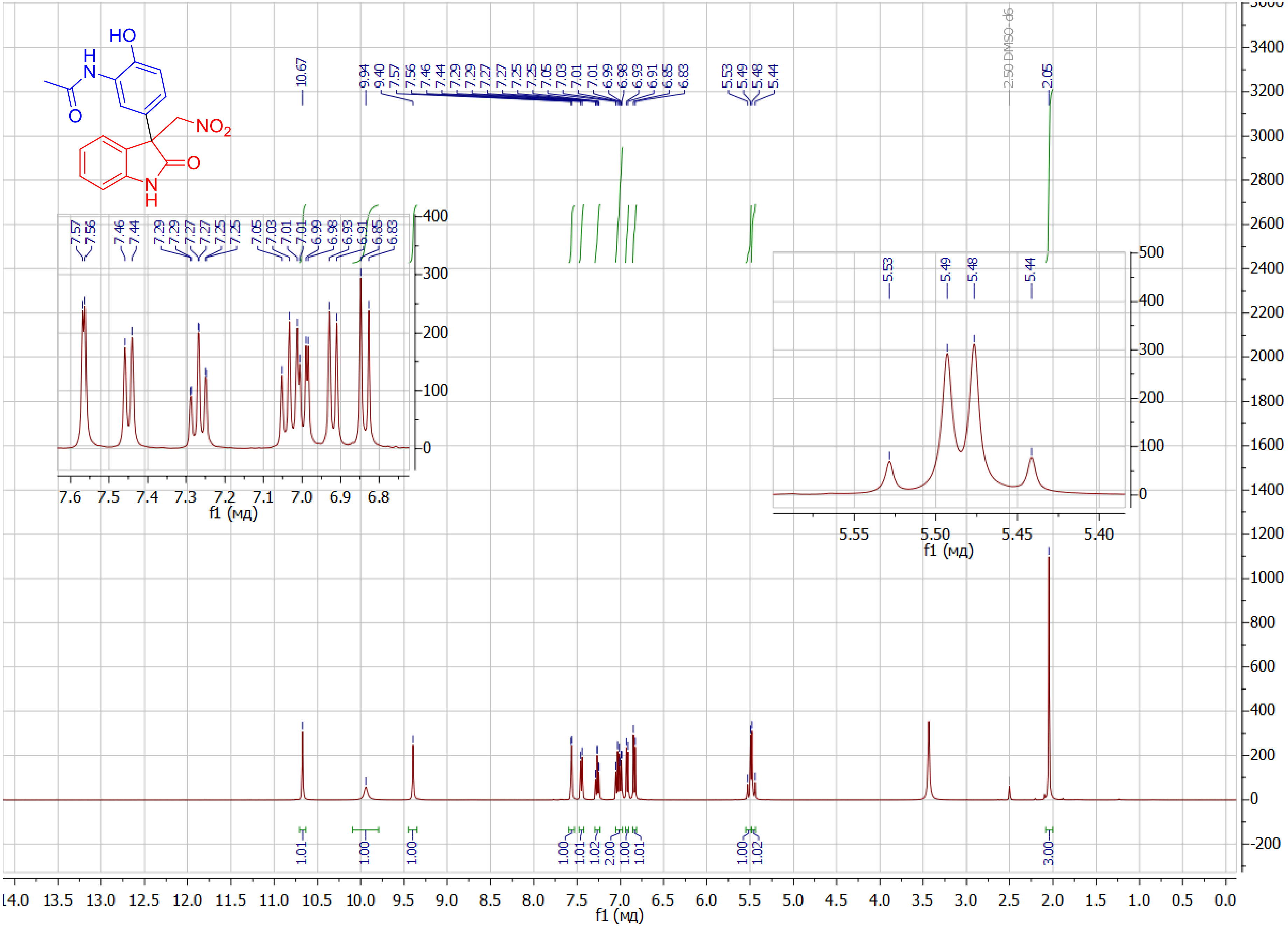

Figure S30. ^{1}H NMR spectrum of compound **7ao** in DMSO-d_6 (400 MHz)

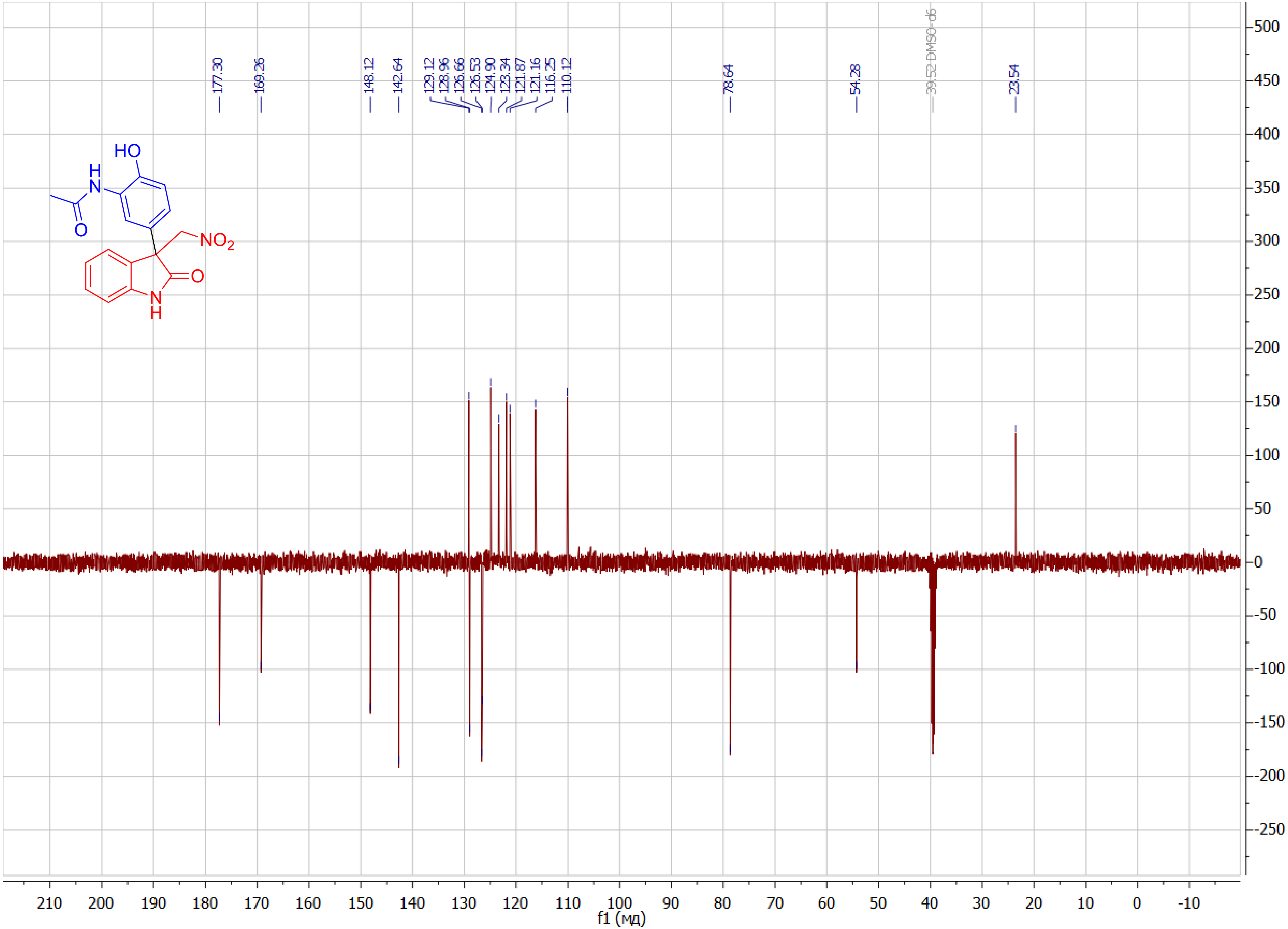

Figure S31. ^{13}C DEPTQ {^{1}H} NMR spectrum of compound **7ao** in DMSO-d_6 (101 MHz)

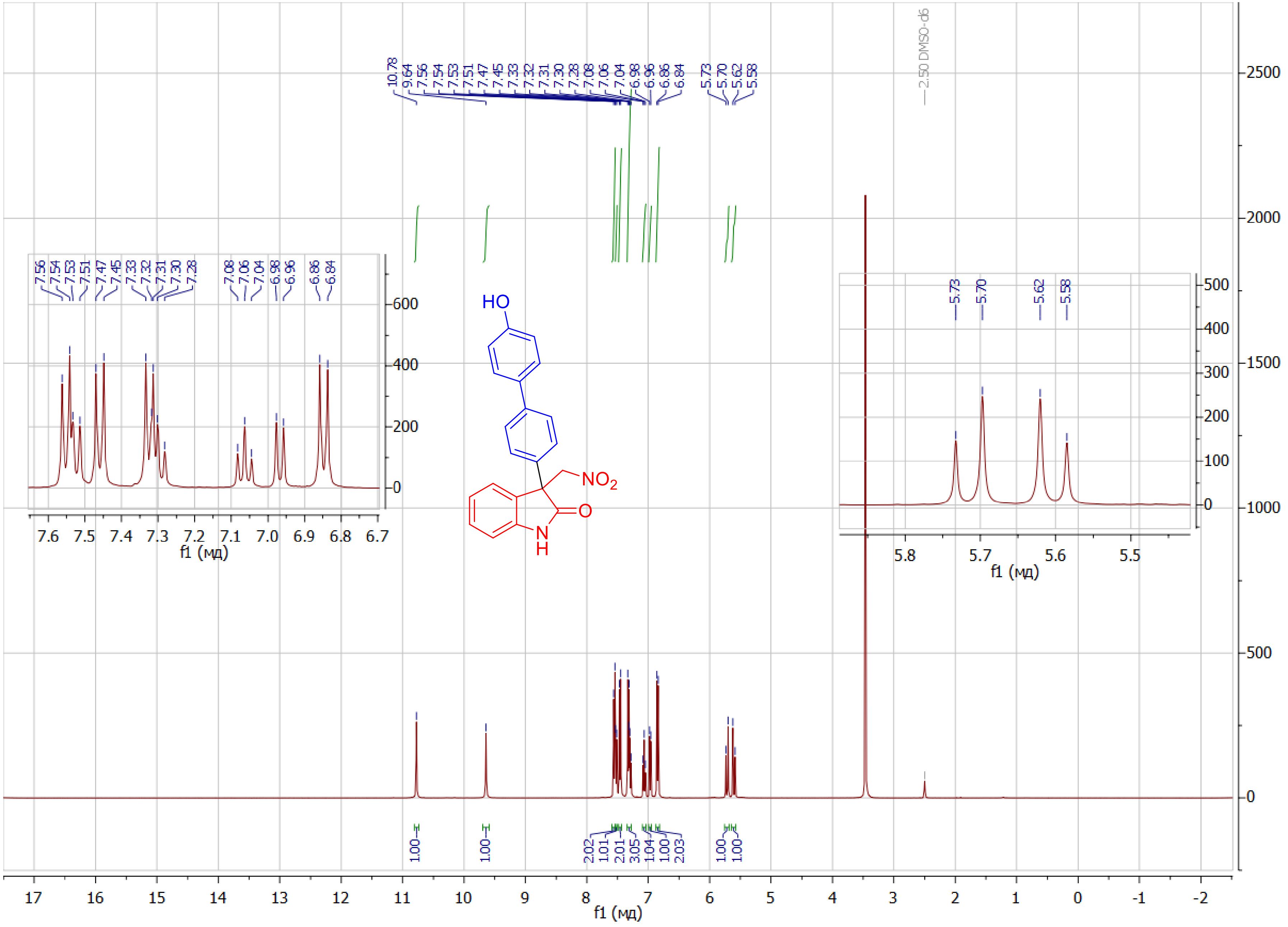

Figure S32. ^{1}H NMR spectrum of compound **7au** in DMSO-d_6 (400 MHz)

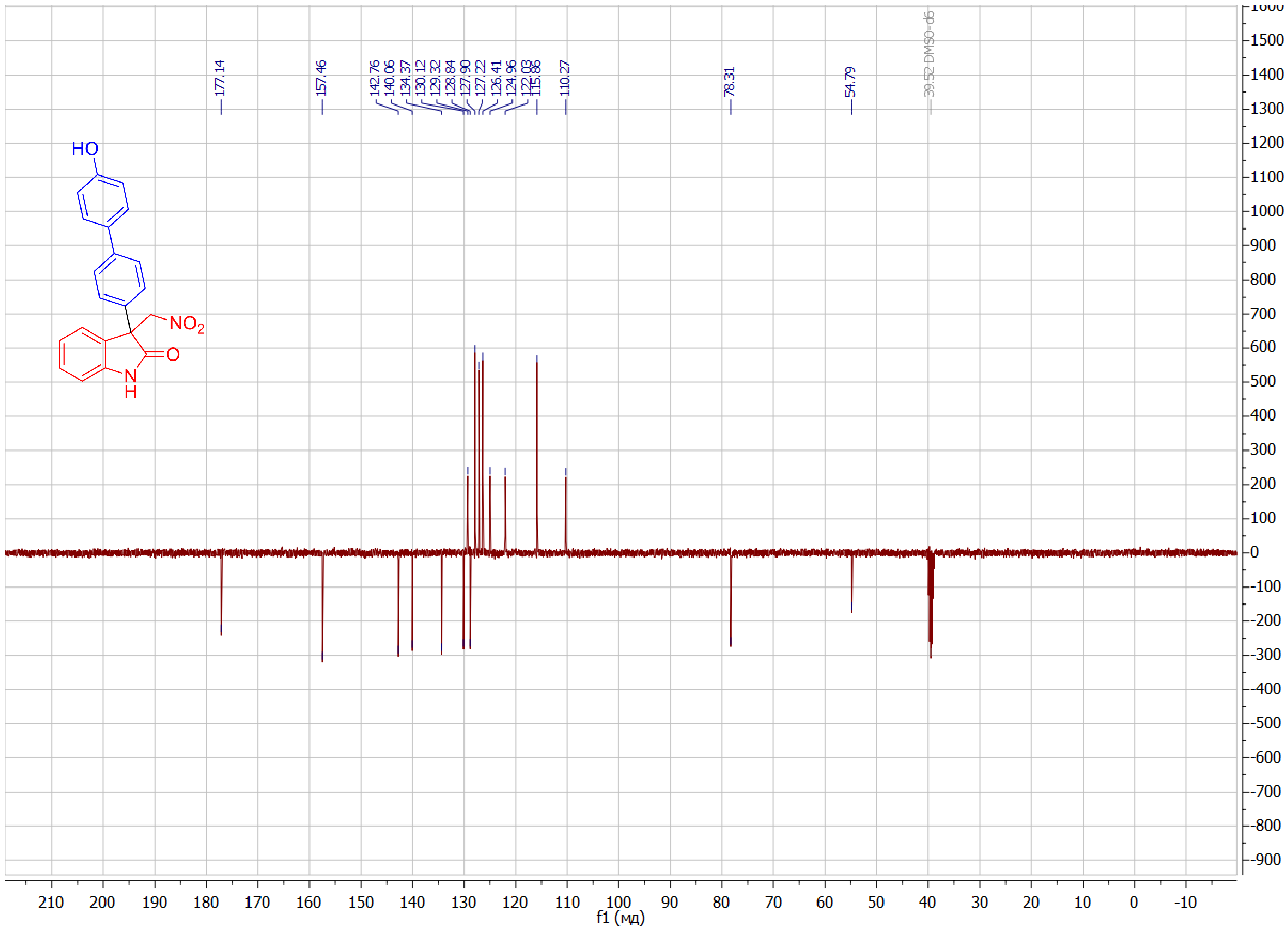

Figure S33. ^{13}C DEPTQ {^{1}H} NMR spectrum of compound **7au** in DMSO-d_6 (101 MHz)

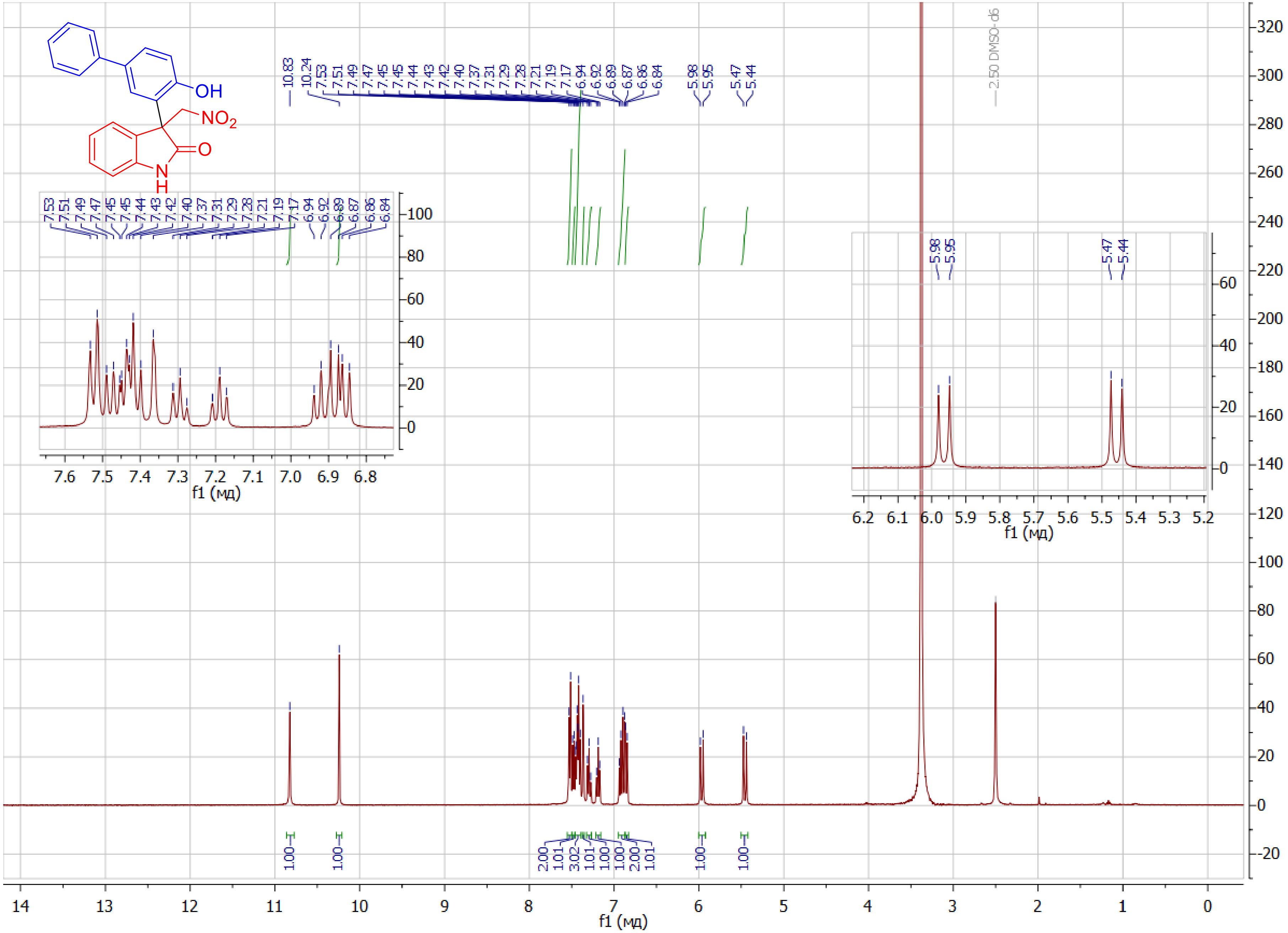

Figure S34. ^{1}H NMR spectrum of compound **7'au** in DMSO-d_6 (400 MHz)

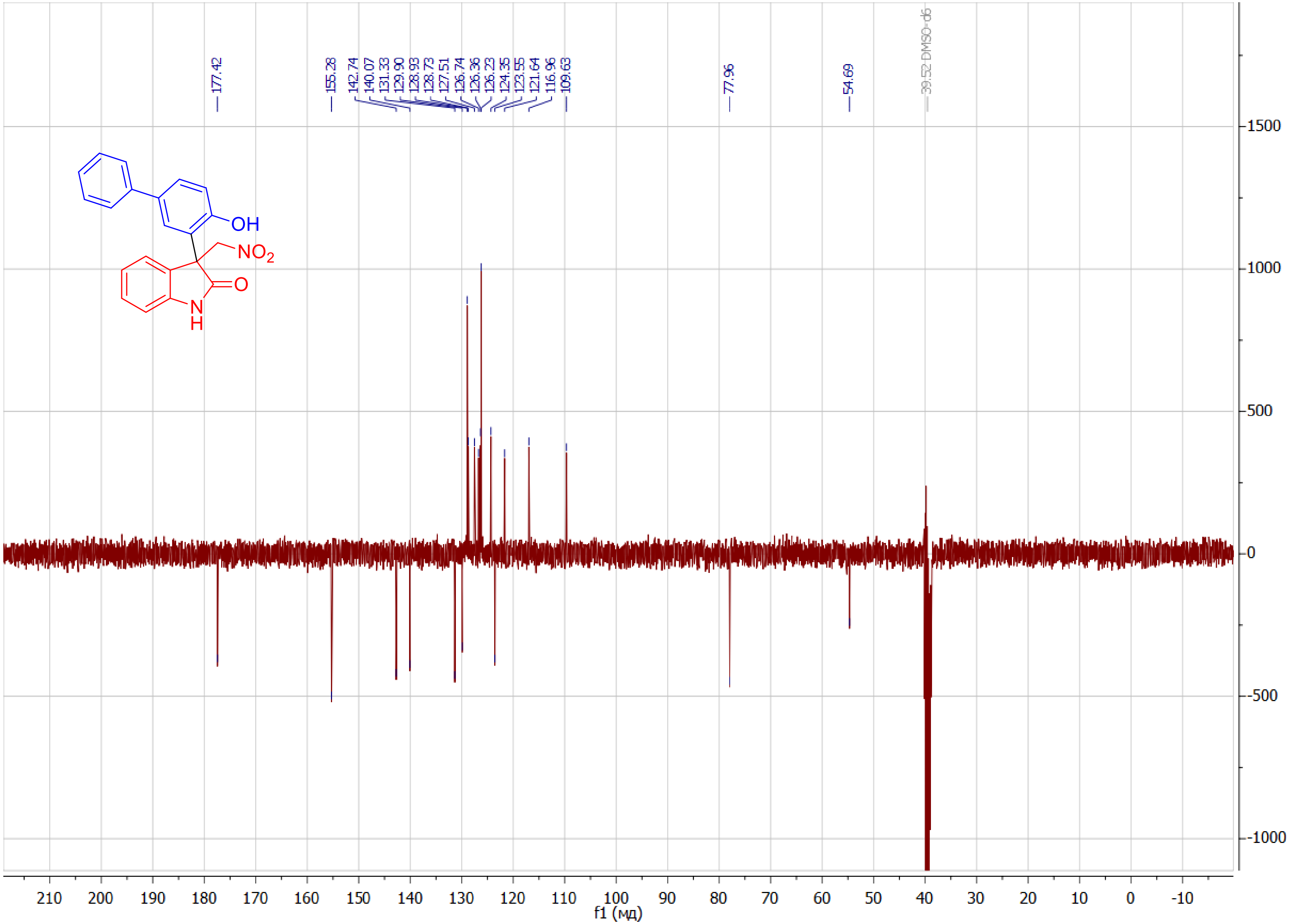

Figure S35. ^{13}C DEPTQ {^{1}H} NMR spectrum of compound **7’au** in DMSO-d_6 (101 MHz)

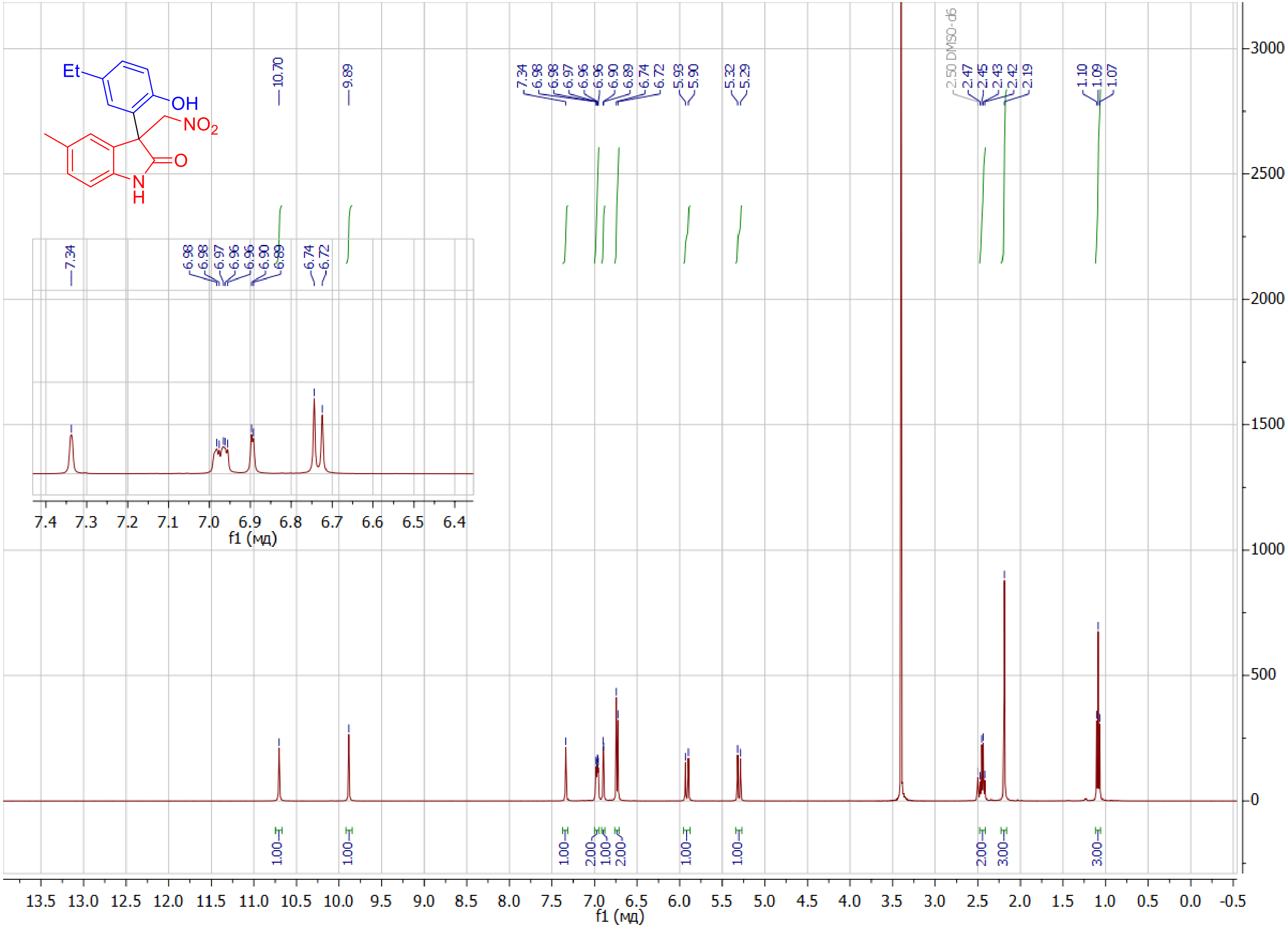

Figure S36. ^{1}H NMR spectrum of compound **7bb** in DMSO-d_6 (400 MHz)

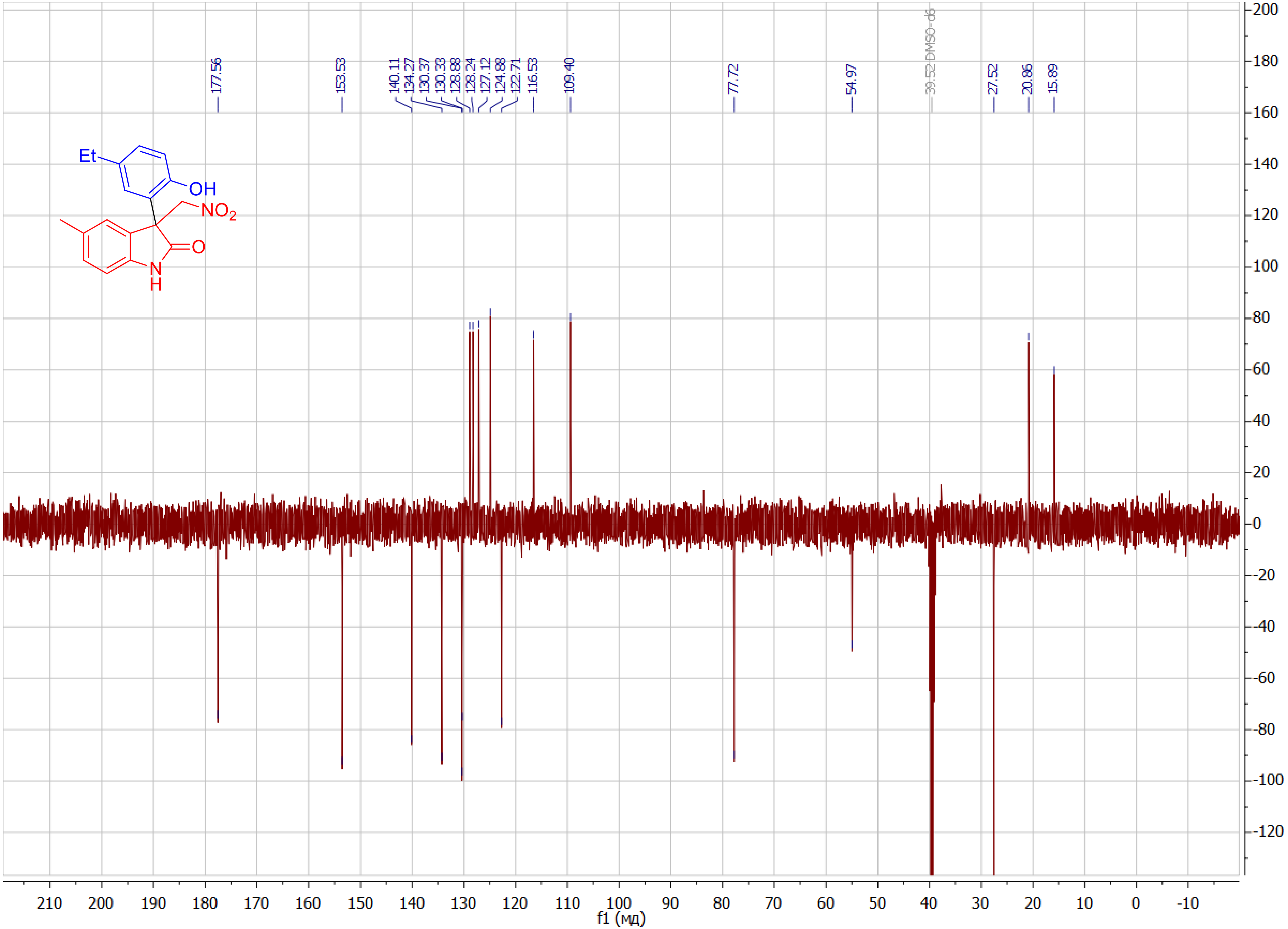

Figure S37. ^{13}C DEPTQ {^{1}H} NMR spectrum of compound **7bb** in DMSO-d_6 (101 MHz)

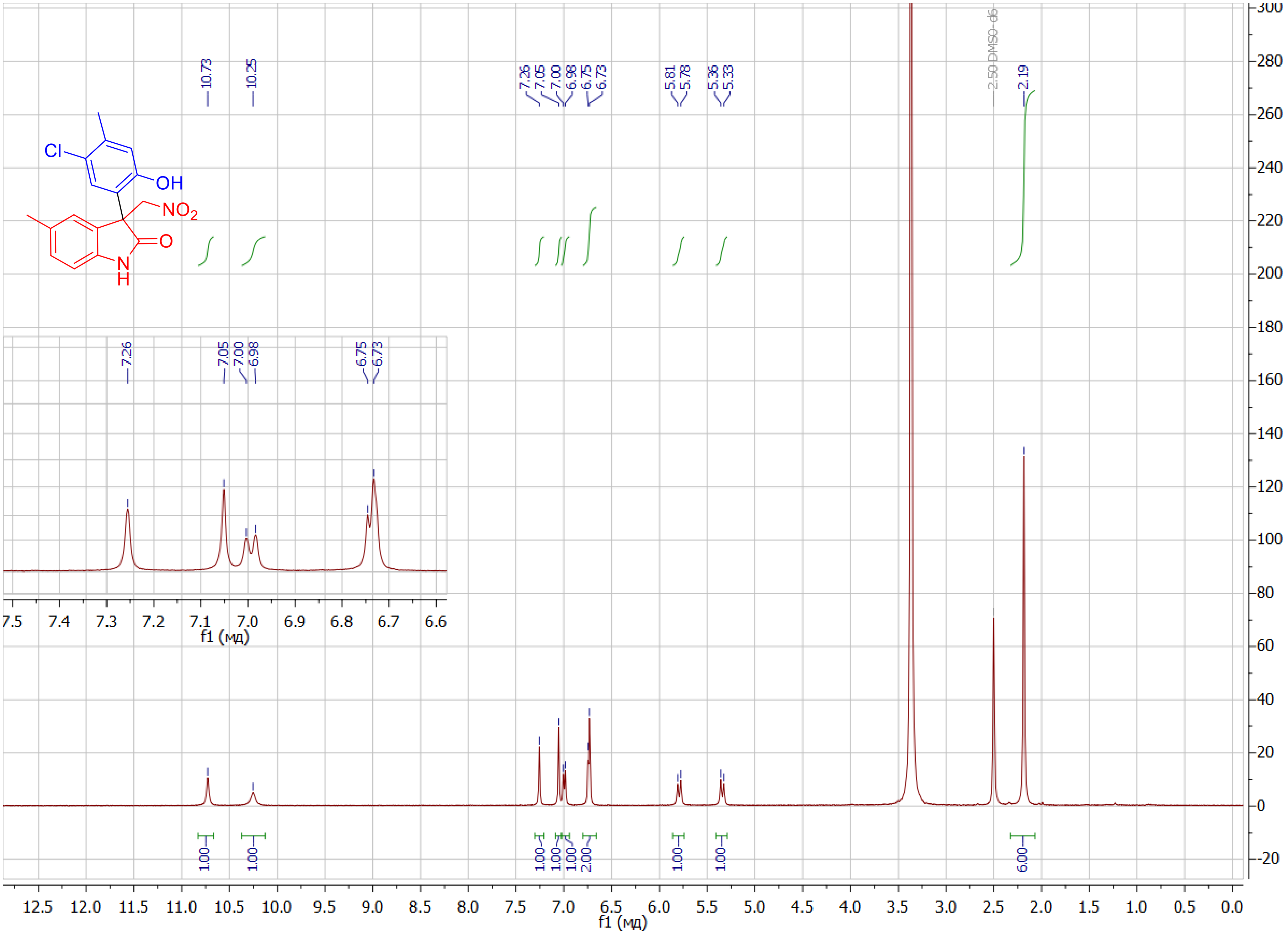

Figure S38. ^{1}H NMR spectrum of compound **7be** in DMSO-d_6 (400 MHz)

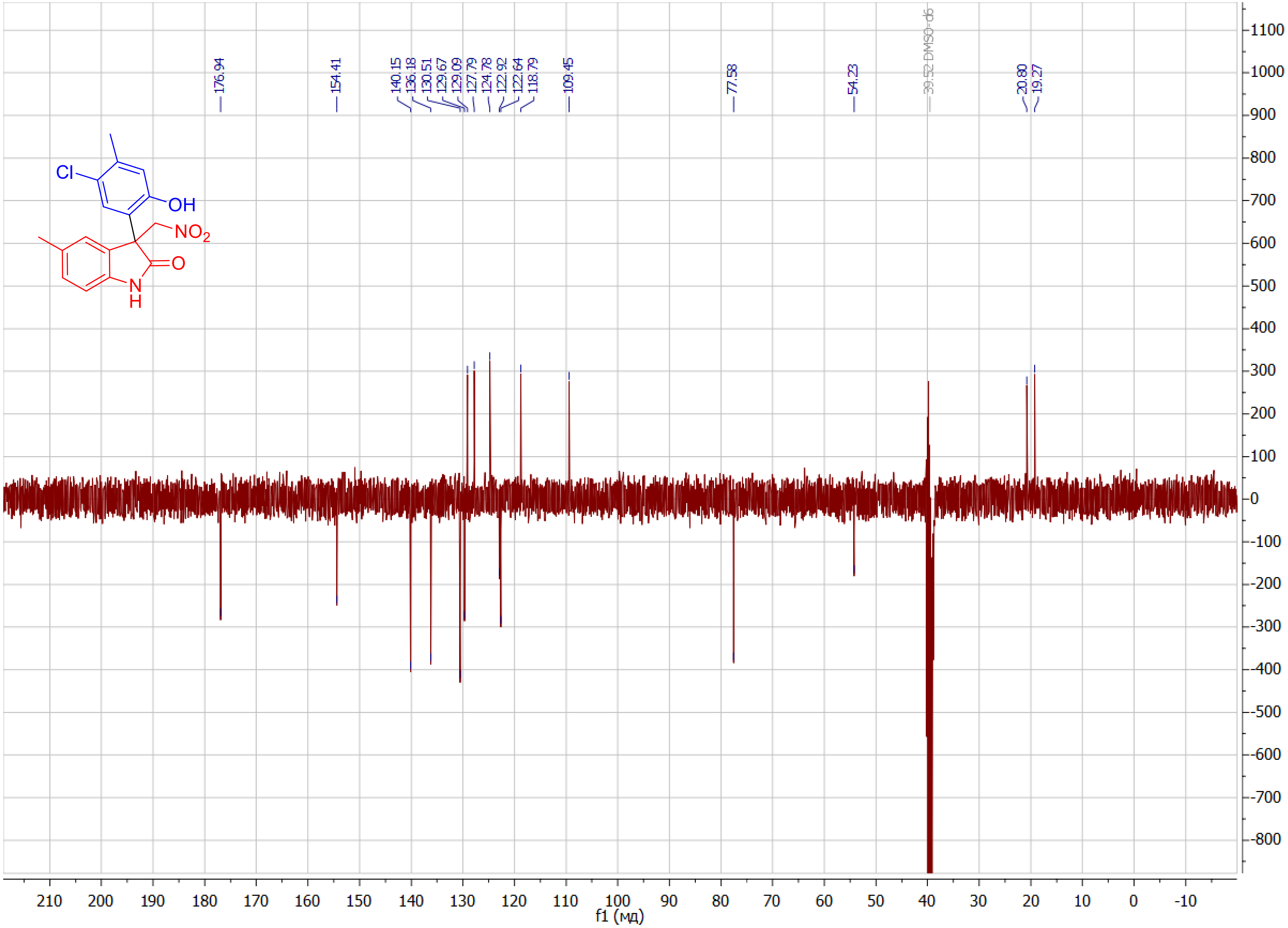

Figure S39. ^{13}C DEPTQ {^{1}H} NMR spectrum of compound **7be** in DMSO-d_6 (101 MHz)

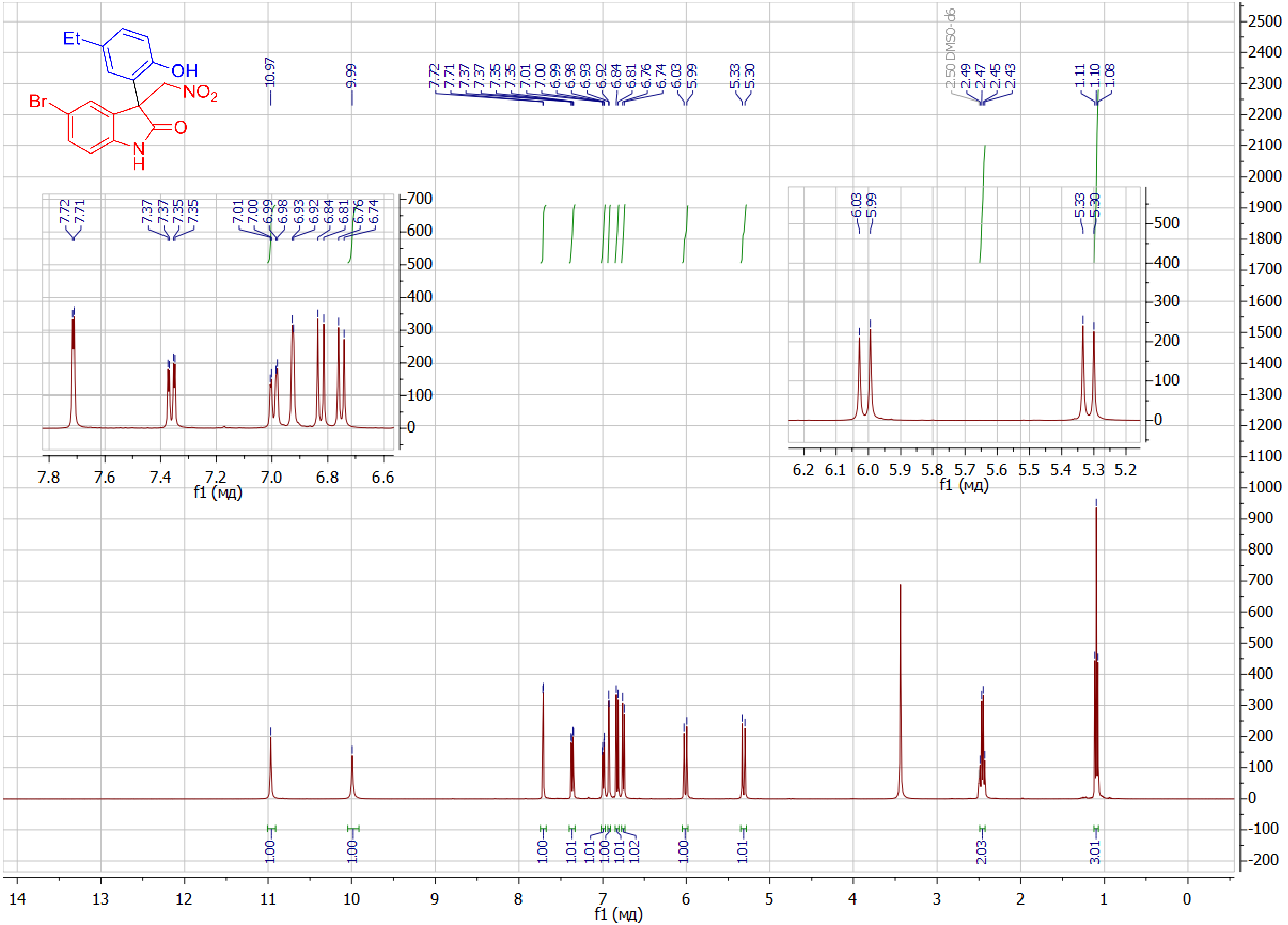

Figure S40. ^{1}H NMR spectrum of compound **7cb** in DMSO-d_6 (400 MHz)

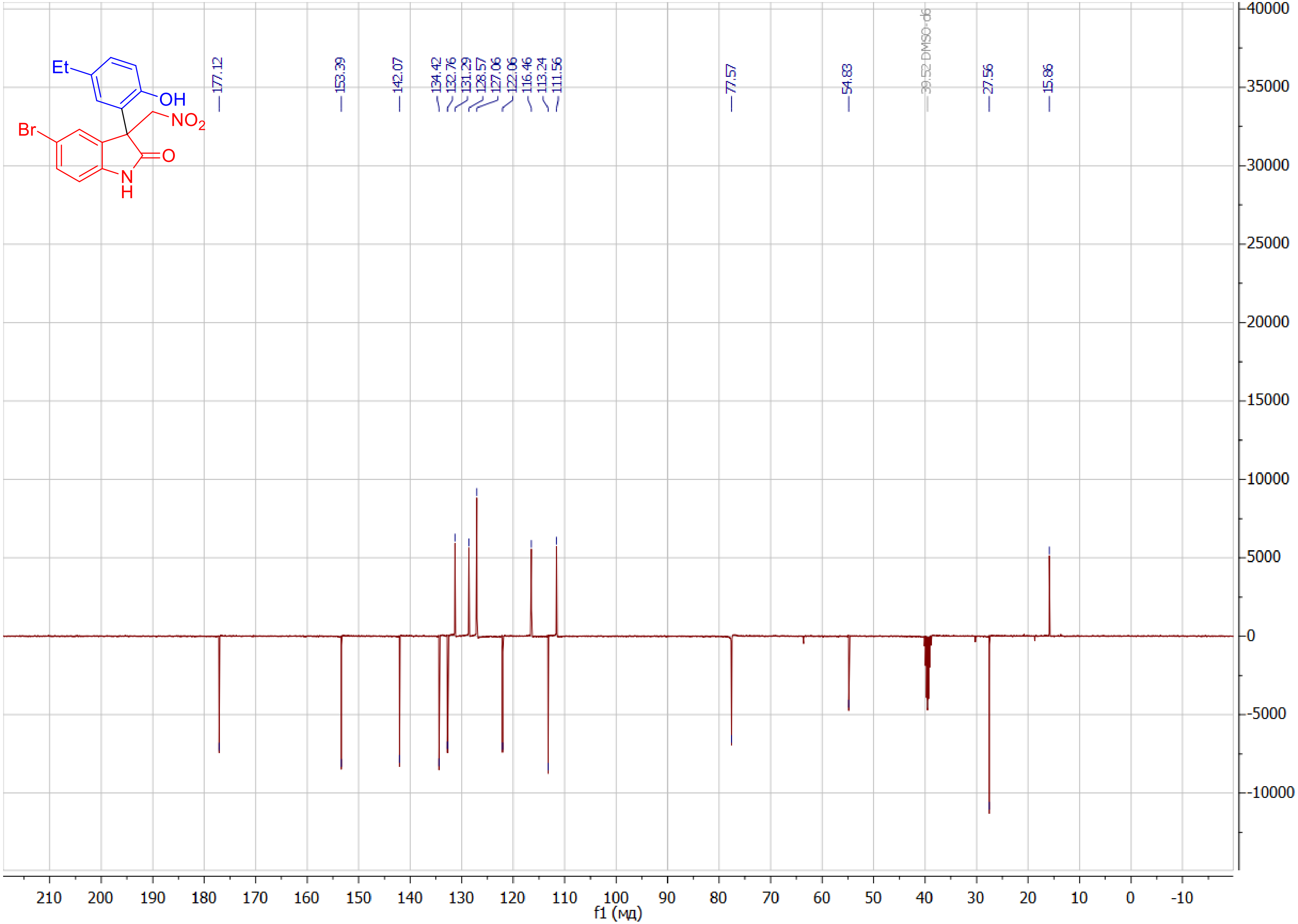

Figure S41. ^{13}C DEPTQ {^{1}H} NMR spectrum of compound **7cb** in DMSO-d_6 (101 MHz)

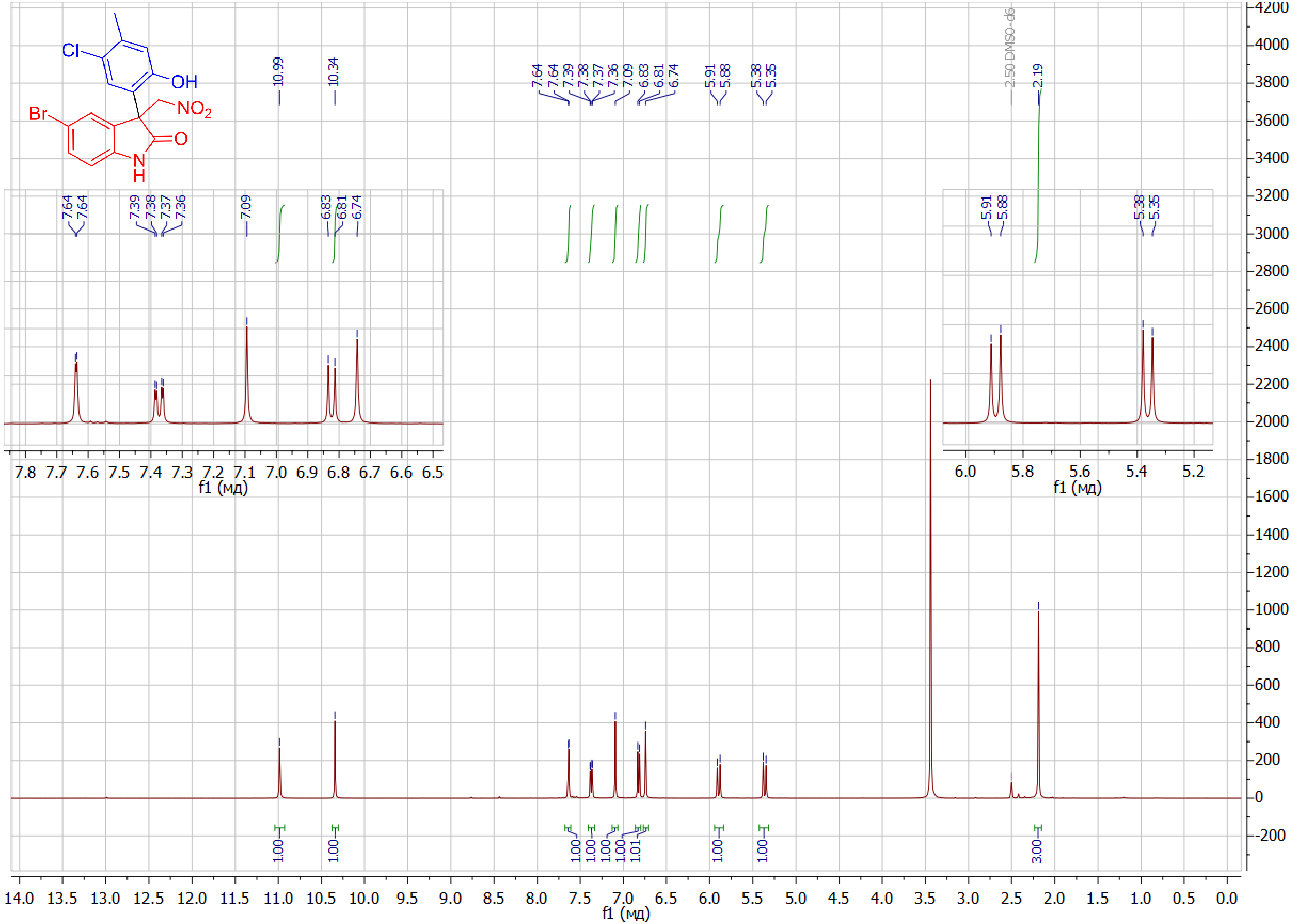

Figure S42. ^{1}H NMR spectrum of compound **7ce** in DMSO-d_6 (400 MHz)

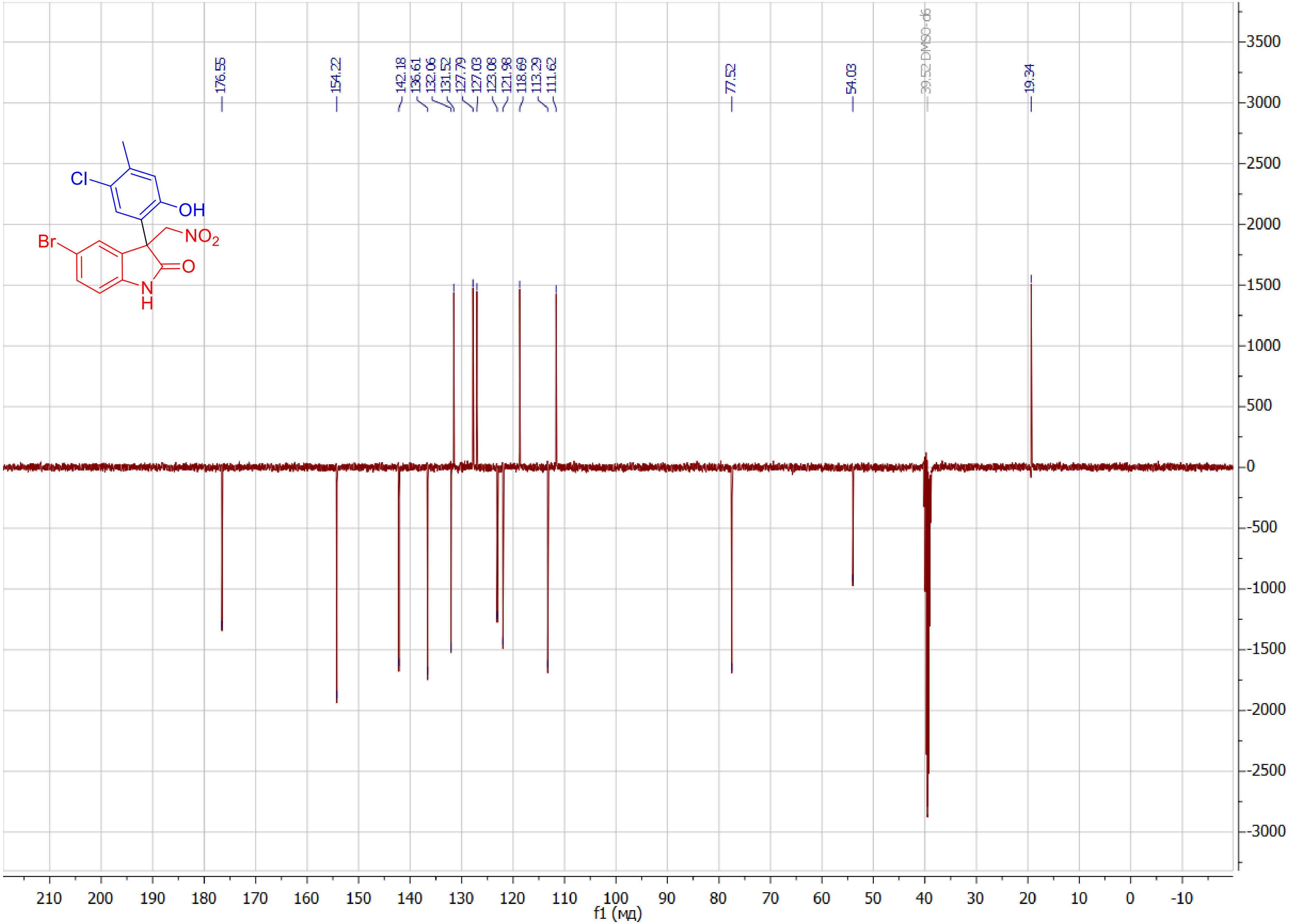

Figure S43. ^{13}C DEPTQ {^{1}H} NMR spectrum of compound **7ce** in DMSO-d_6 (101 MHz)

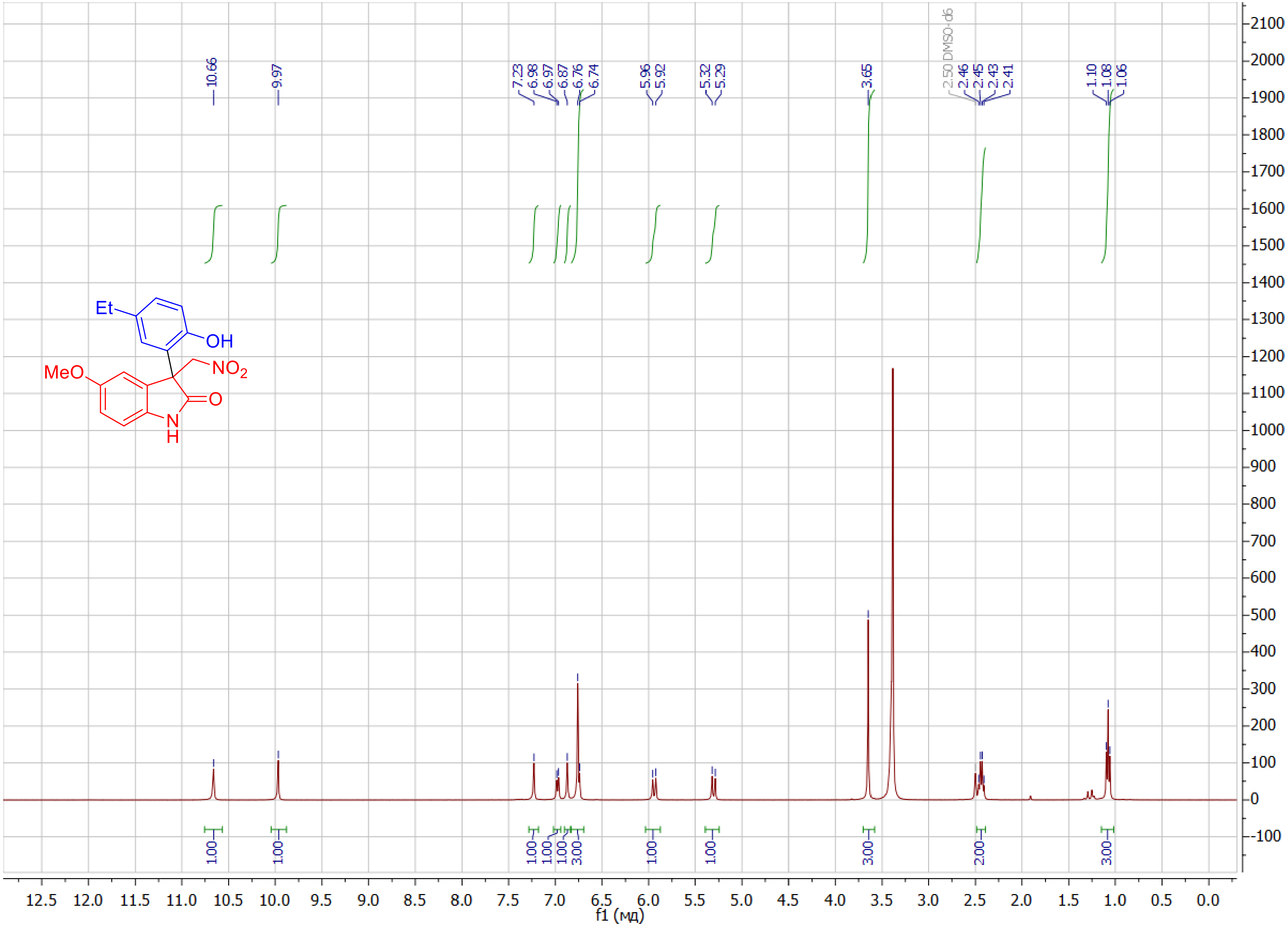

Figure S44. ^{1}H NMR spectrum of compound **7db** in DMSO-d_6 (400 MHz)

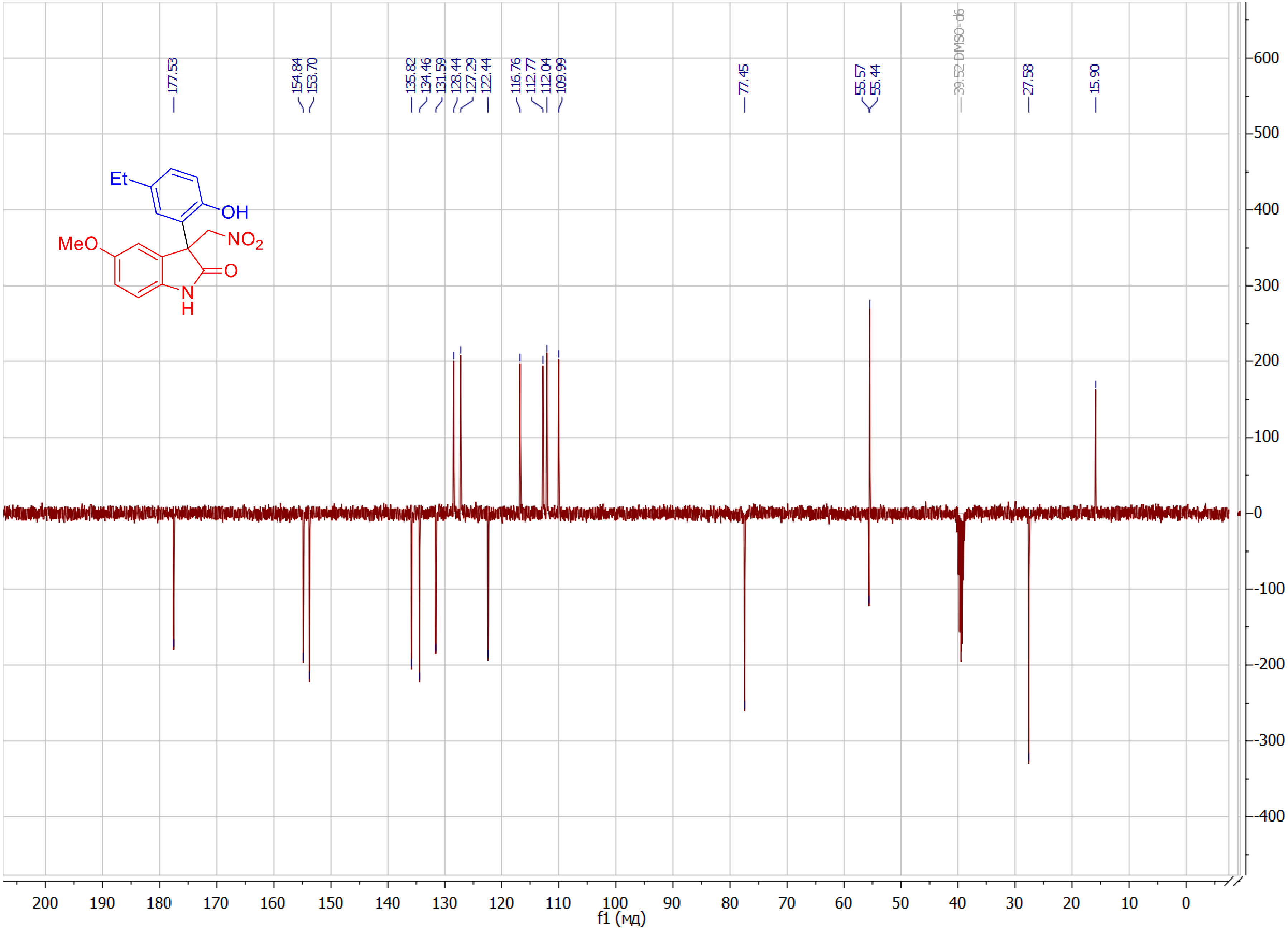

Figure S45. ^{13}C DEPTQ {^{1}H} NMR spectrum of compound **7db** in DMSO-d_6 (101 MHz)

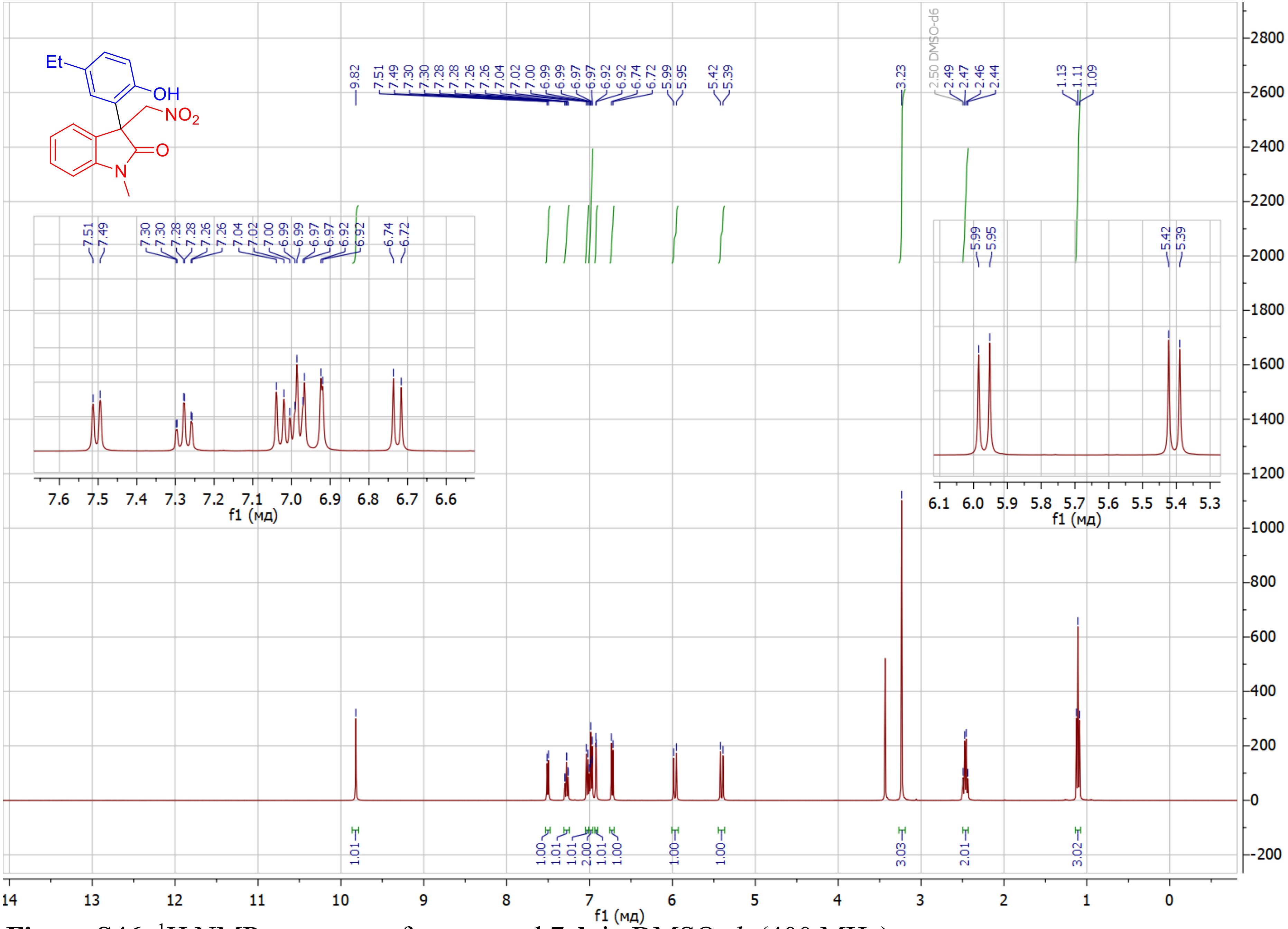

Figure S46. ^{1}H NMR spectrum of compound **7eb** in DMSO-d_6 (400 MHz)

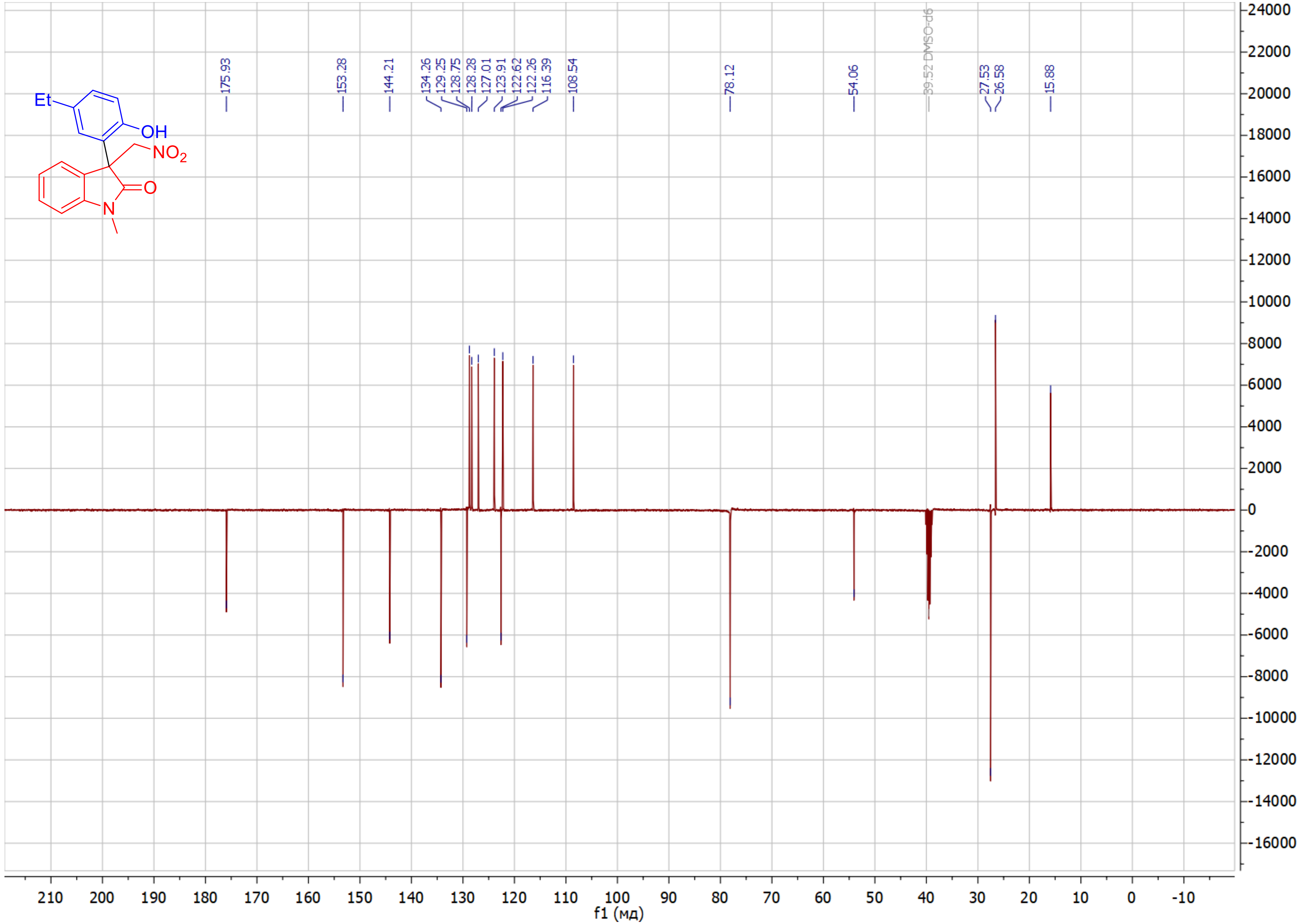

Figure S47. ^{13}C DEPTQ {^{1}H} NMR spectrum of compound **7eb** in DMSO-d_6 (101 MHz)

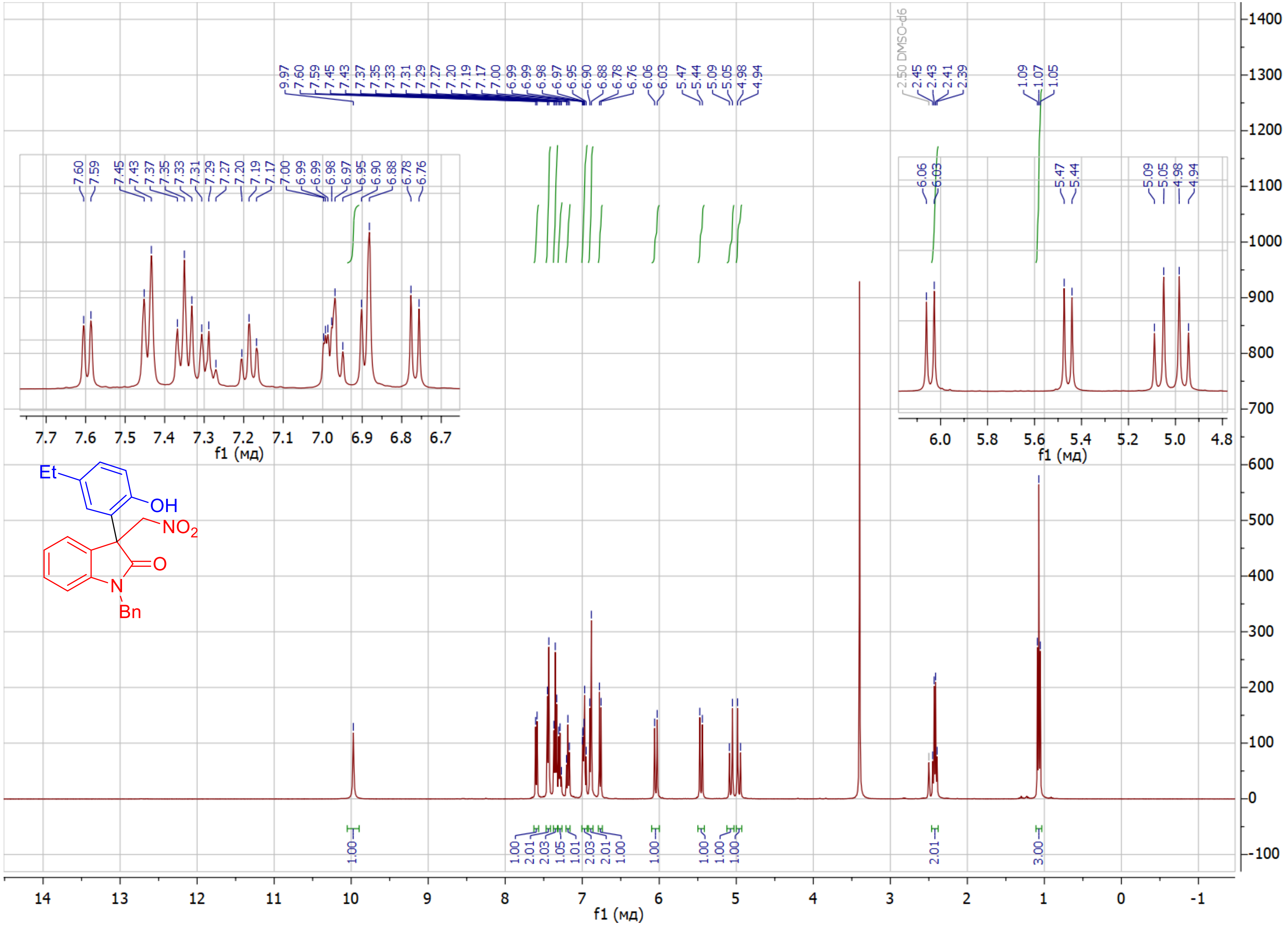

Figure S48. ^{1}H NMR spectrum of compound **7fb** in DMSO-d_6 (400 MHz)

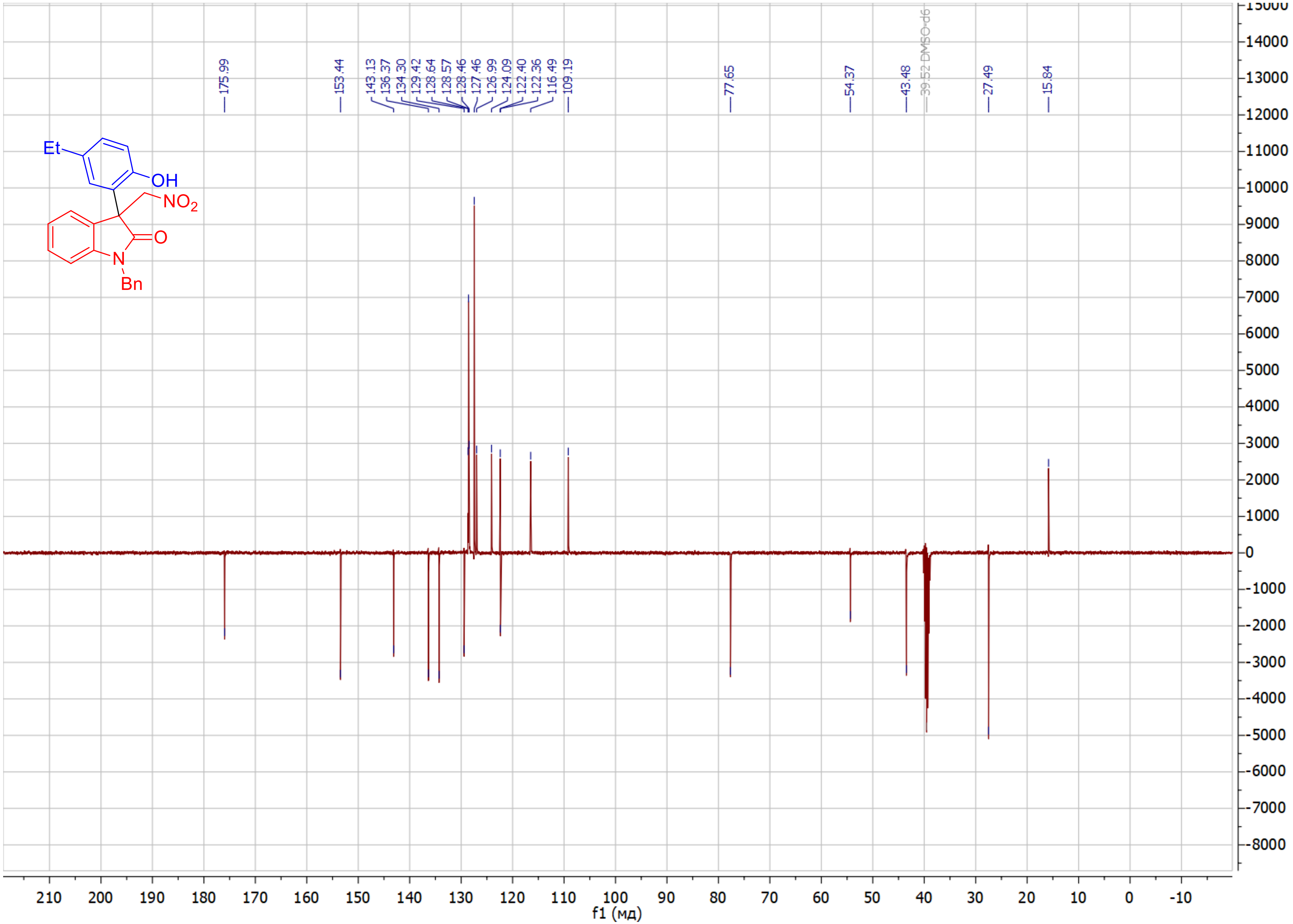

Figure S49. ^{13}C DEPTQ {^{1}H} NMR spectrum of compound **7fb** in DMSO-d_6 (101 MHz)

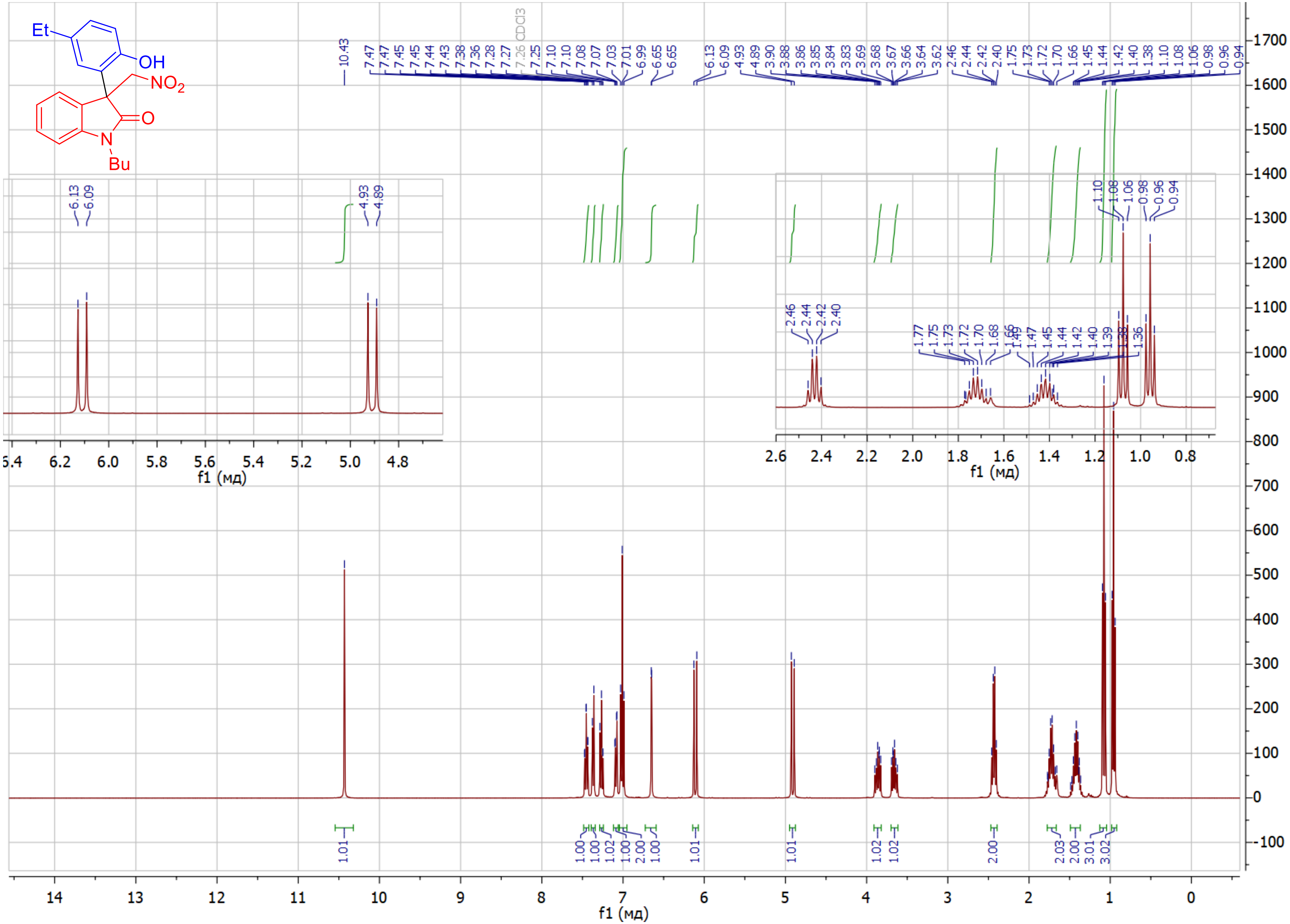

Figure S50. ^{1}H NMR spectrum of compound **7gb** in $CDCl_3$ (400 MHz)

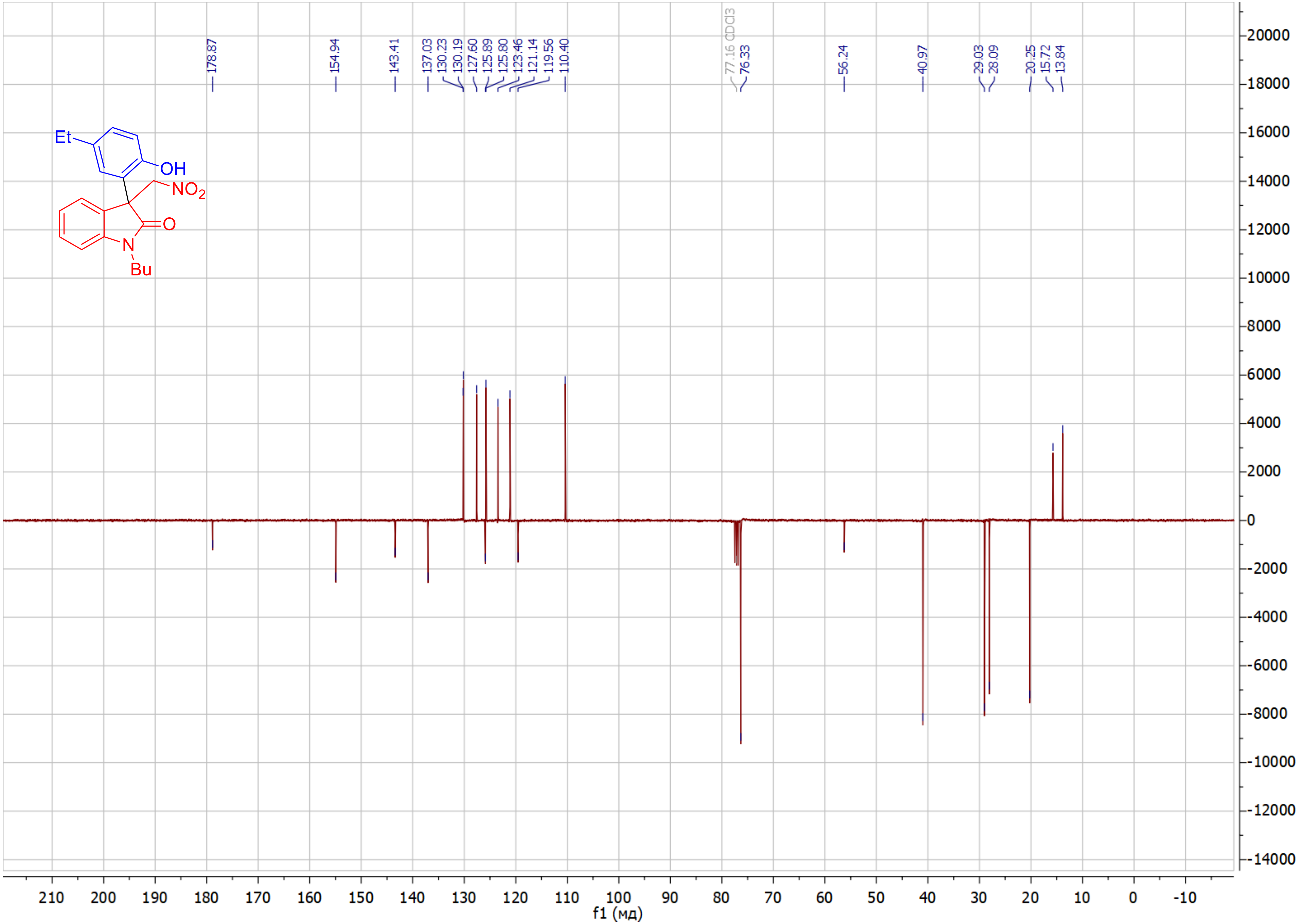

Figure S51. ^{13}C DEPTQ {^{1}H} NMR spectrum of compound **7gb** in $CDCl_3$ (101 MHz)

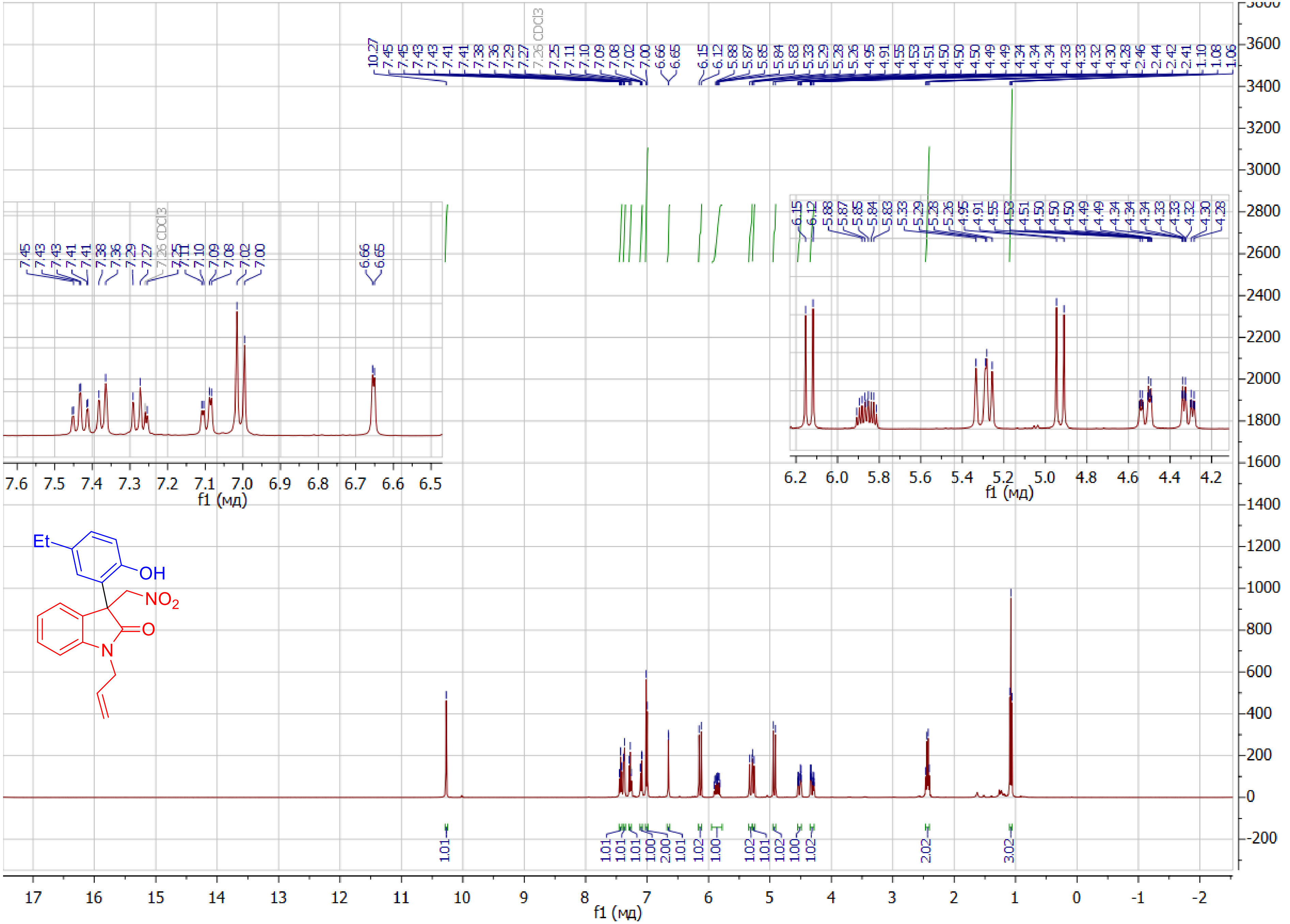

Figure S52. ^{1}H NMR spectrum of compound **7hb** in $CDCl_3$ (400 MHz)

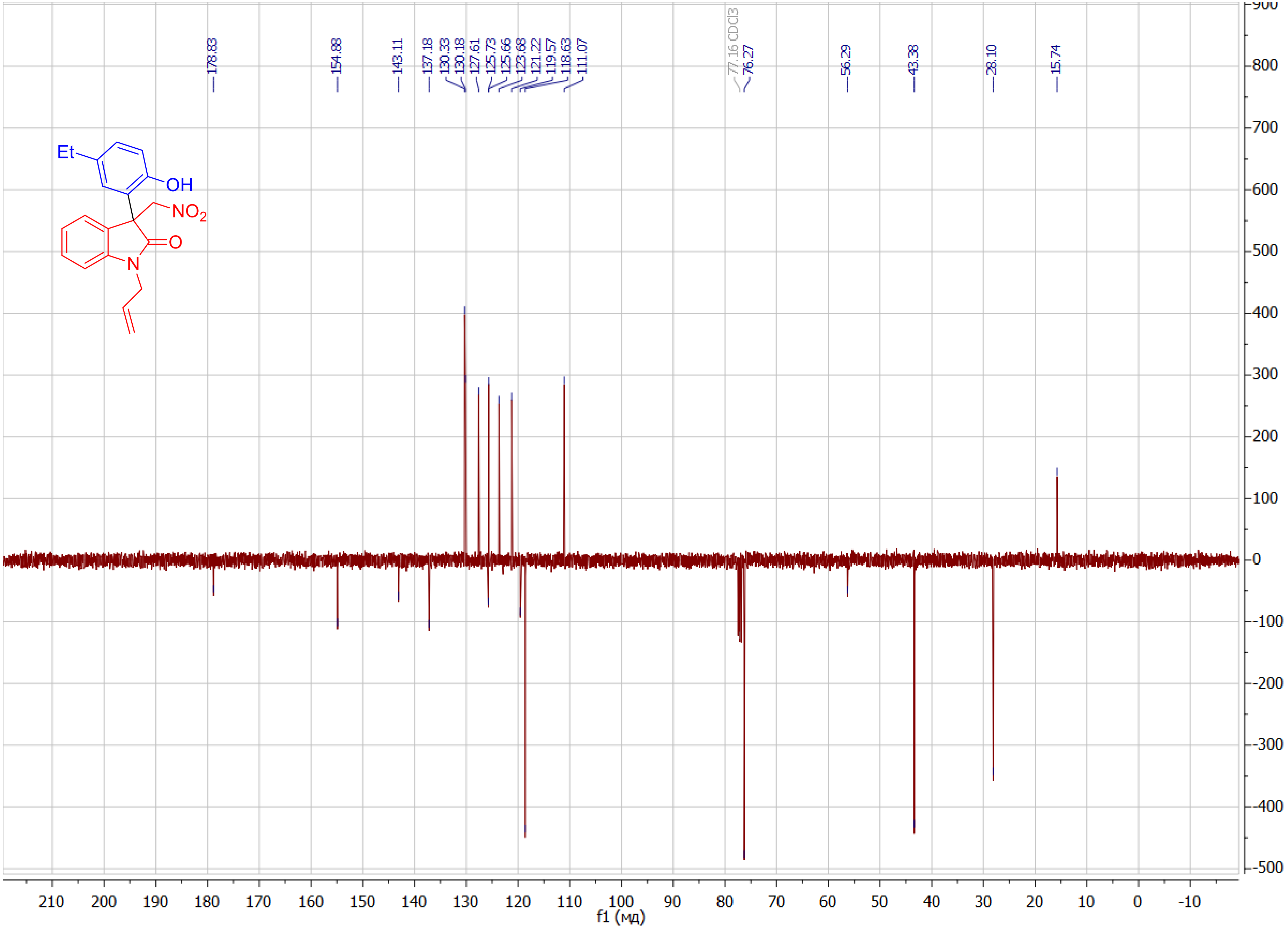

Figure S53. ^{13}C DEPTQ {^{1}H} NMR spectrum of compound **7hb** in $CDCl_3$ (101 MHz)

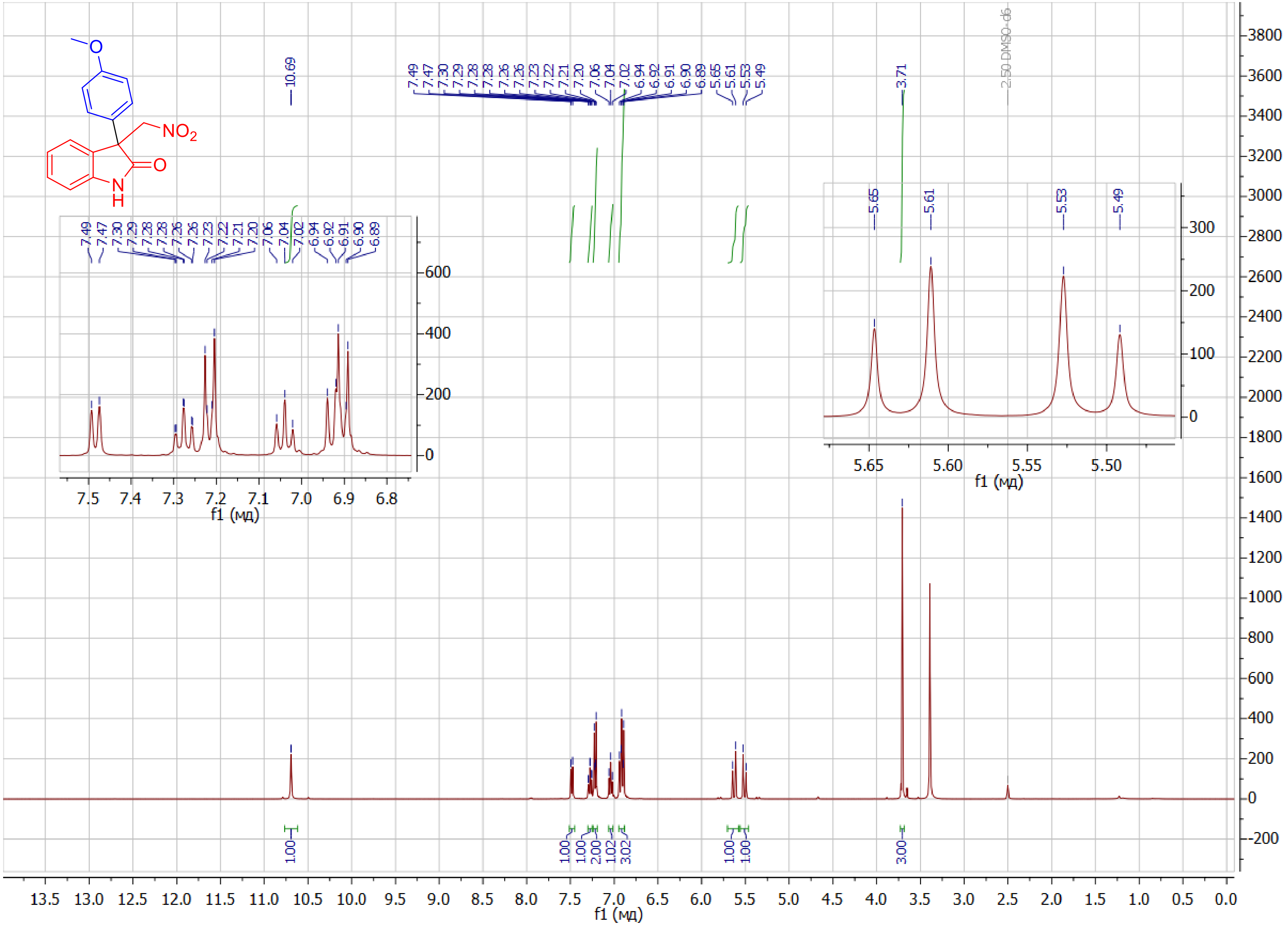

Figure S54. ^{1}H NMR spectrum of compound **7av** in DMSO-d_6 (400 MHz)

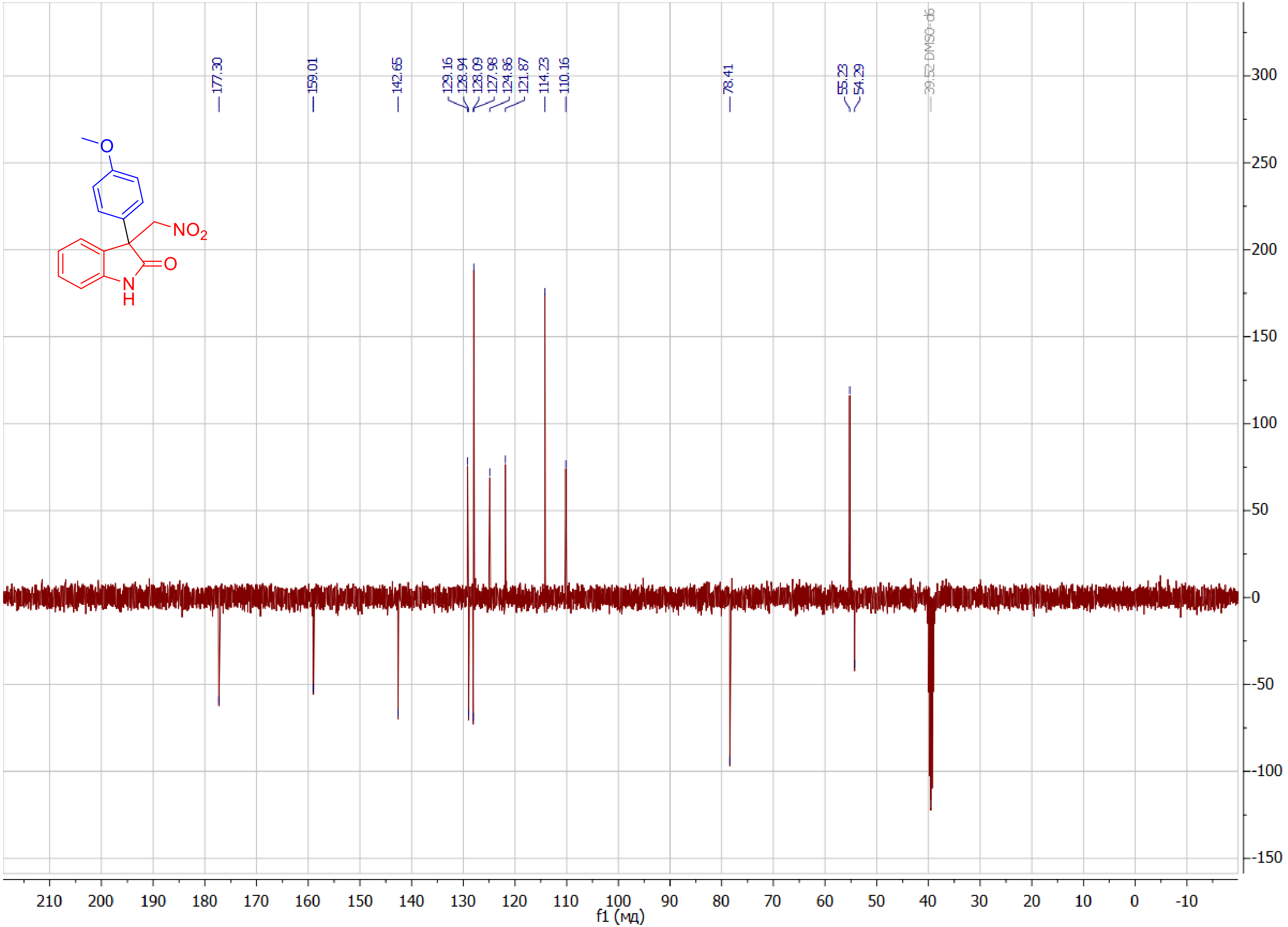

Figure S55. ^{13}C DEPTQ {^{1}H} NMR spectrum of compound **7av** in DMSO-d_6 (101 MHz)

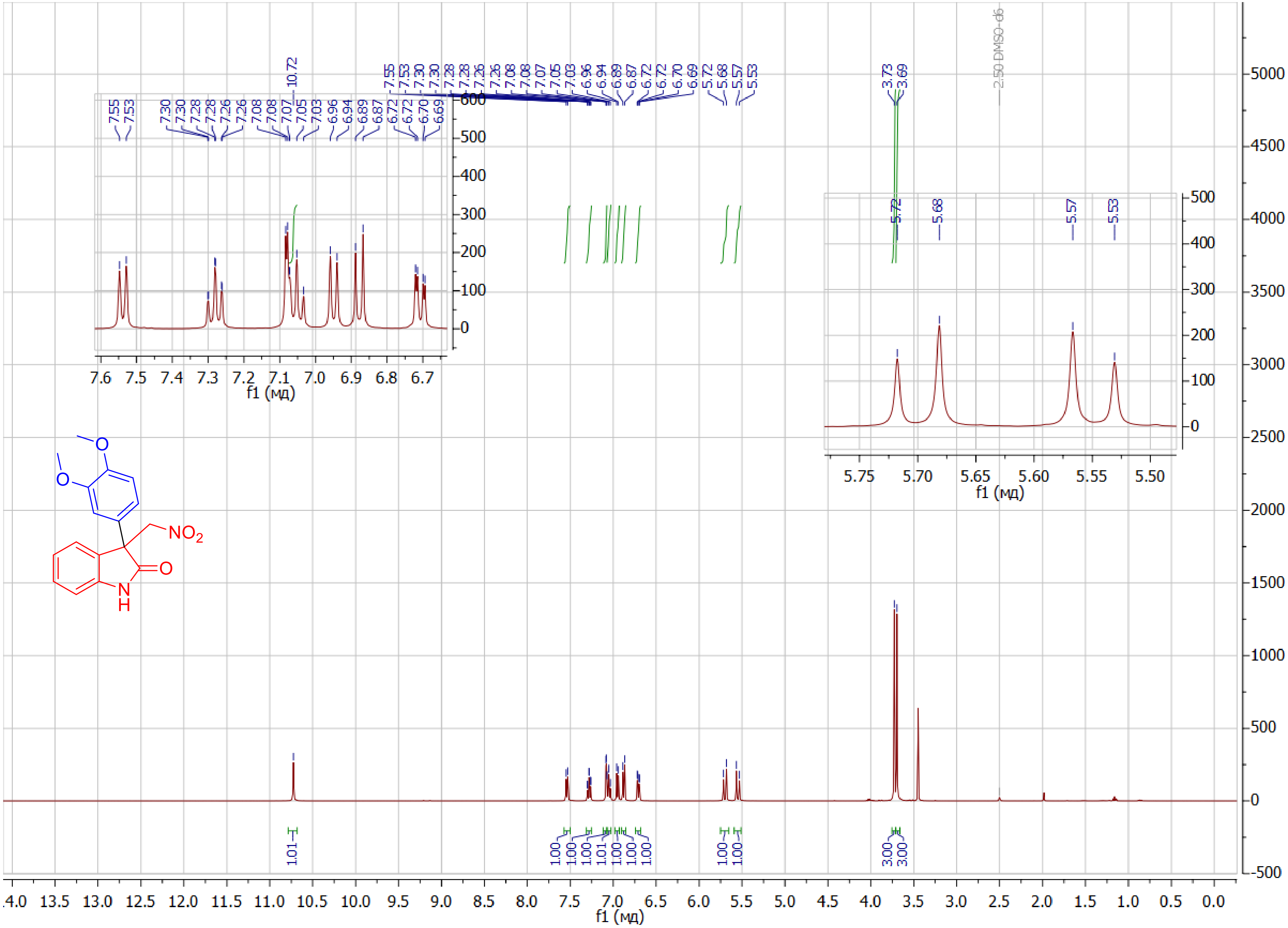

Figure S56. ^{1}H NMR spectrum of compound **7aw** in DMSO-d_6 (400 MHz)

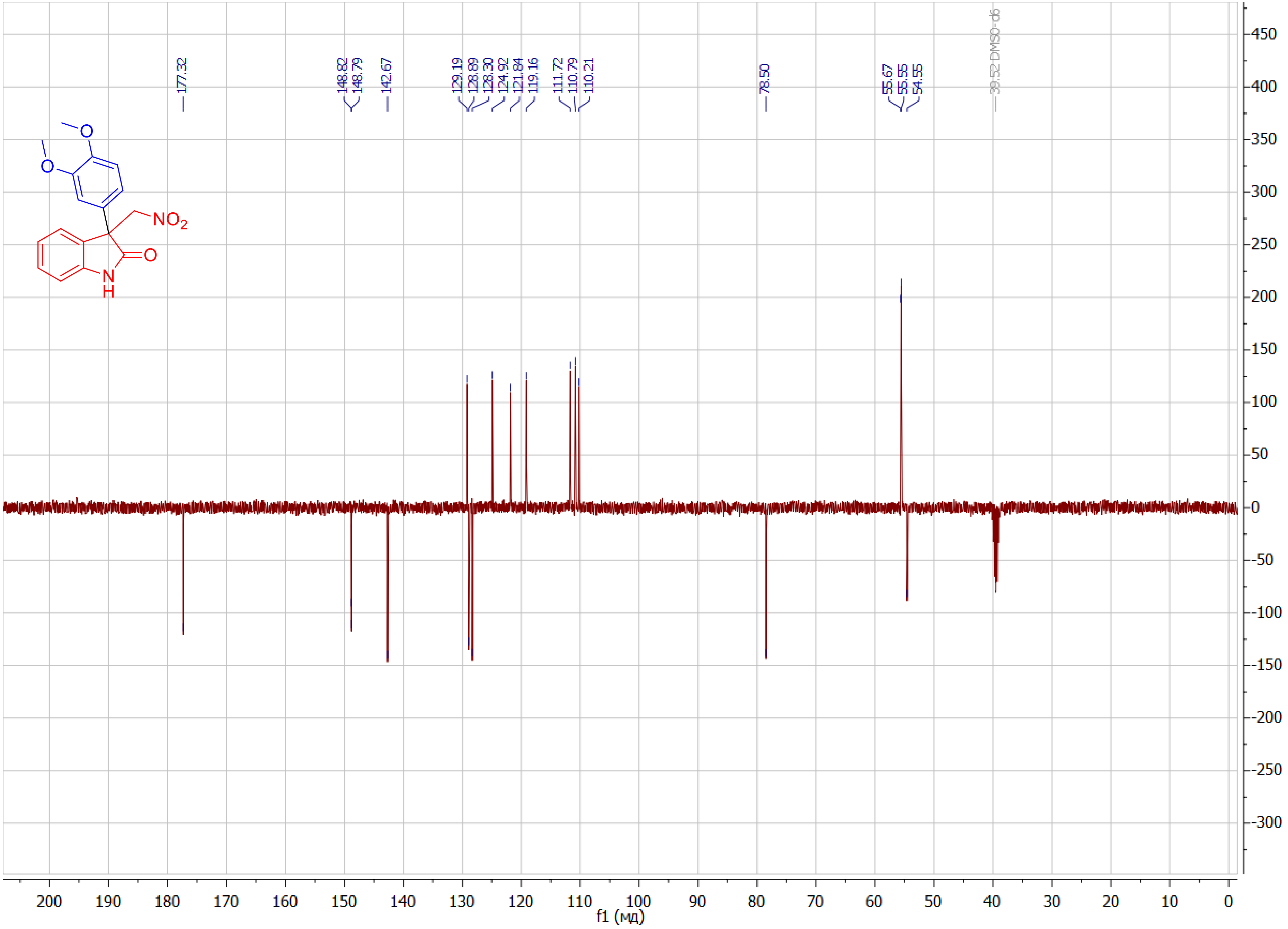

Figure S57. ^{13}C DEPTQ {^{1}H} NMR spectrum of compound **7aw** in DMSO-d_6 (101 MHz)

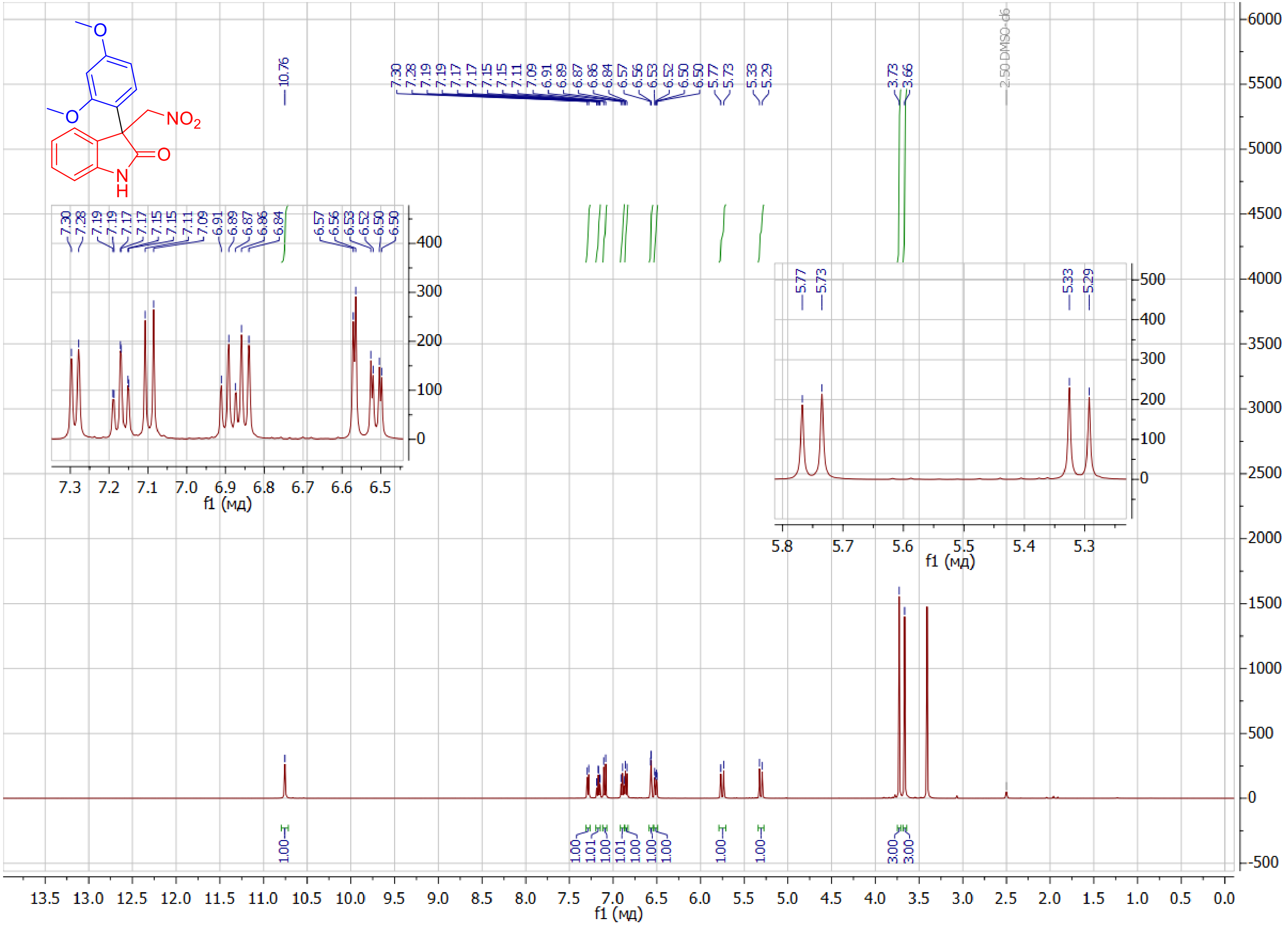

Figure S58. ^{1}H NMR spectrum of compound **7ax** in DMSO-d_6 (400 MHz)

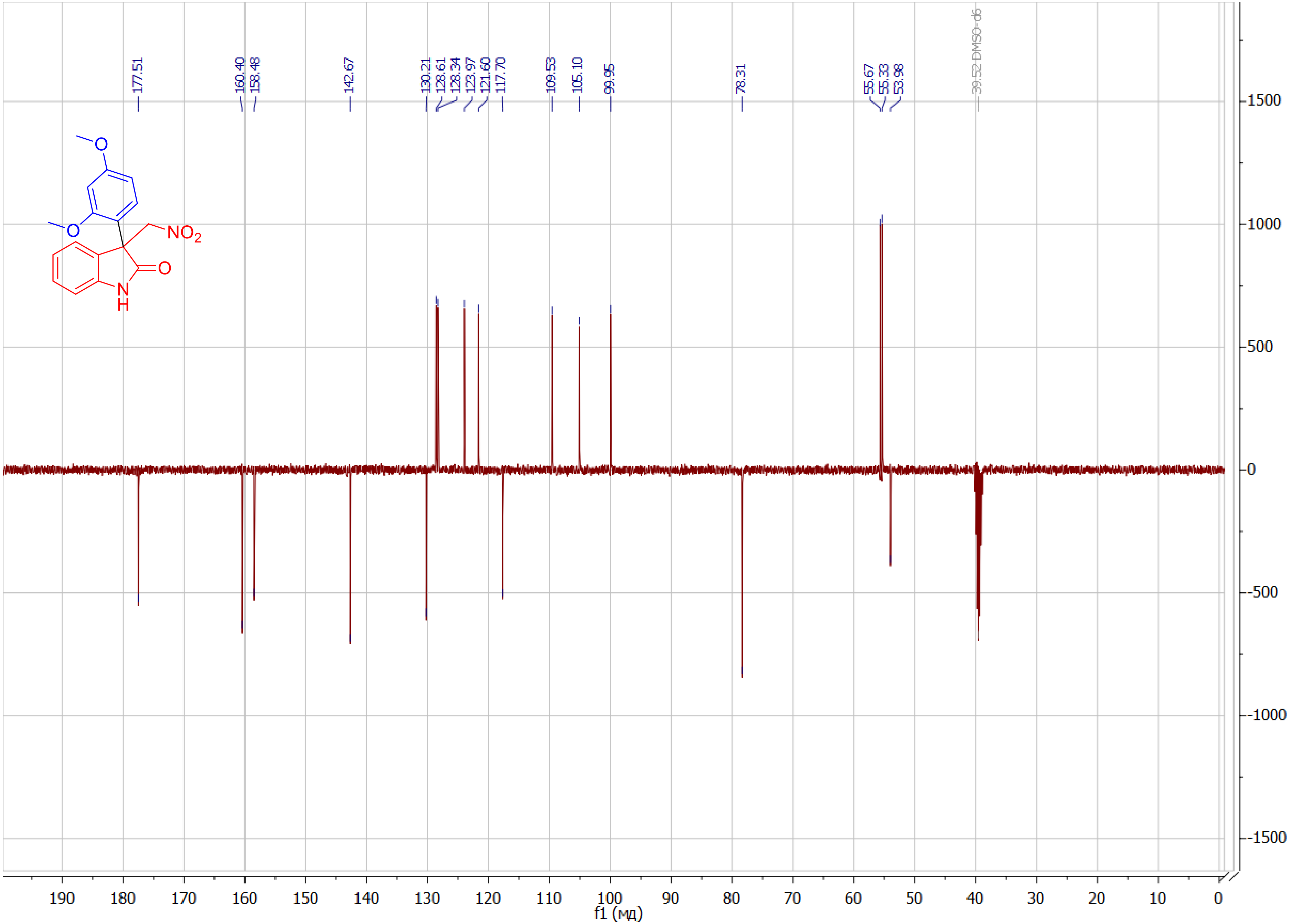

Figure S59. ^{13}C DEPTQ {^{1}H} NMR spectrum of compound **7ax** in DMSO-d_6 (101 MHz)

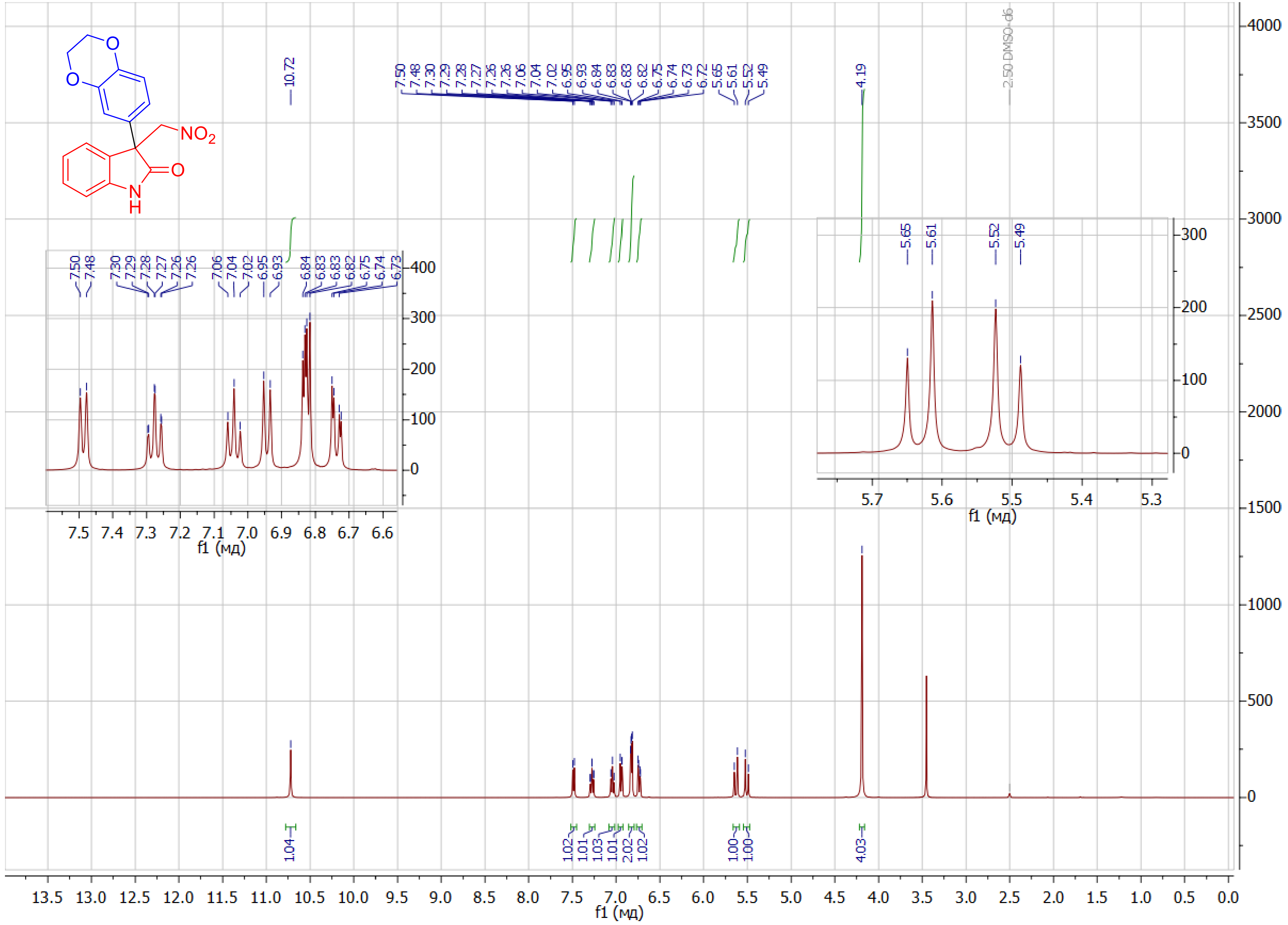

Figure S60. ^{1}H NMR spectrum of compound **7ay** in DMSO-d_6 (400 MHz)

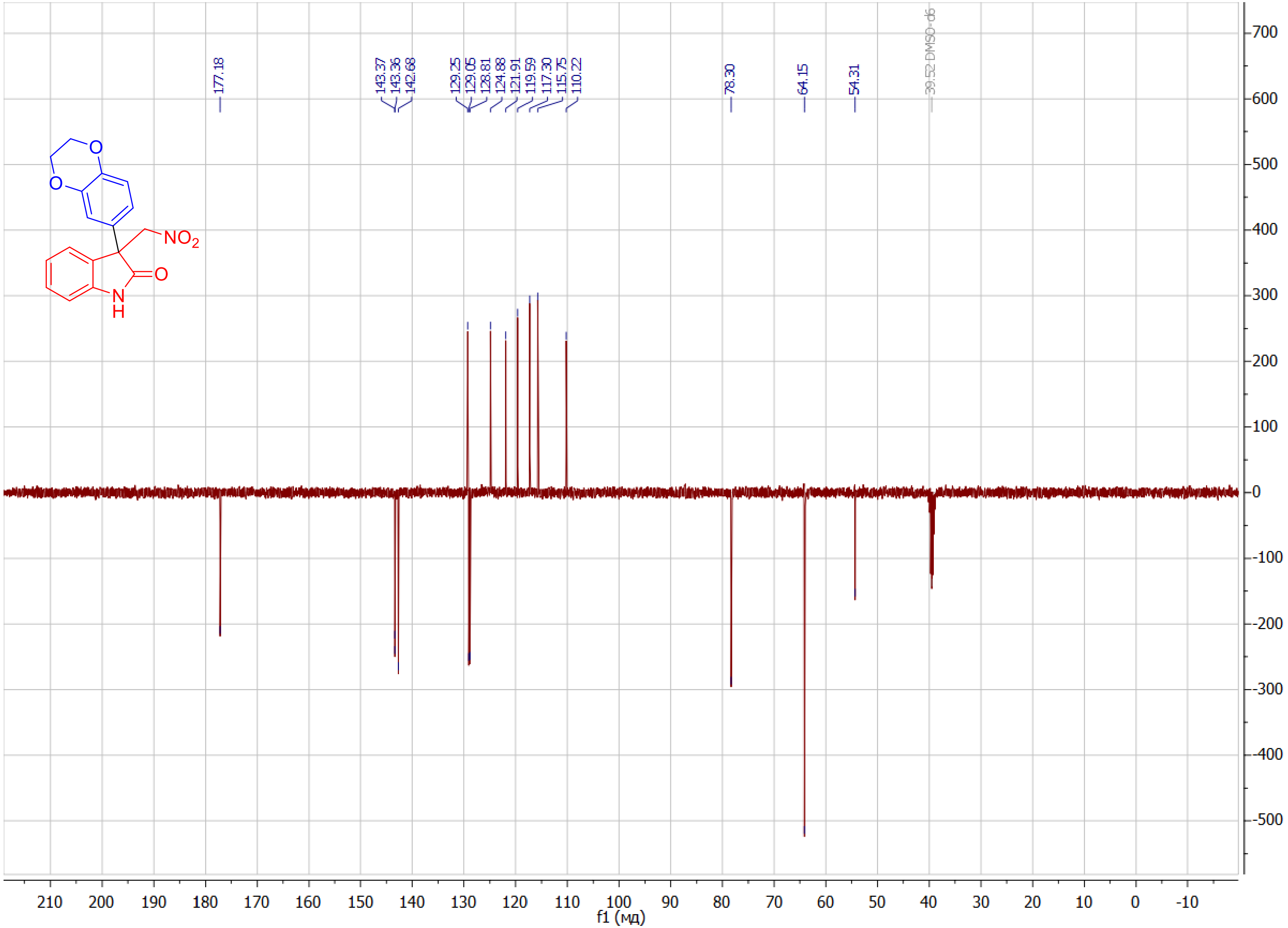

Figure S61. ^{13}C DEPTQ {^{1}H} NMR spectrum of compound **7ay** in DMSO-d_6 (101 MHz)

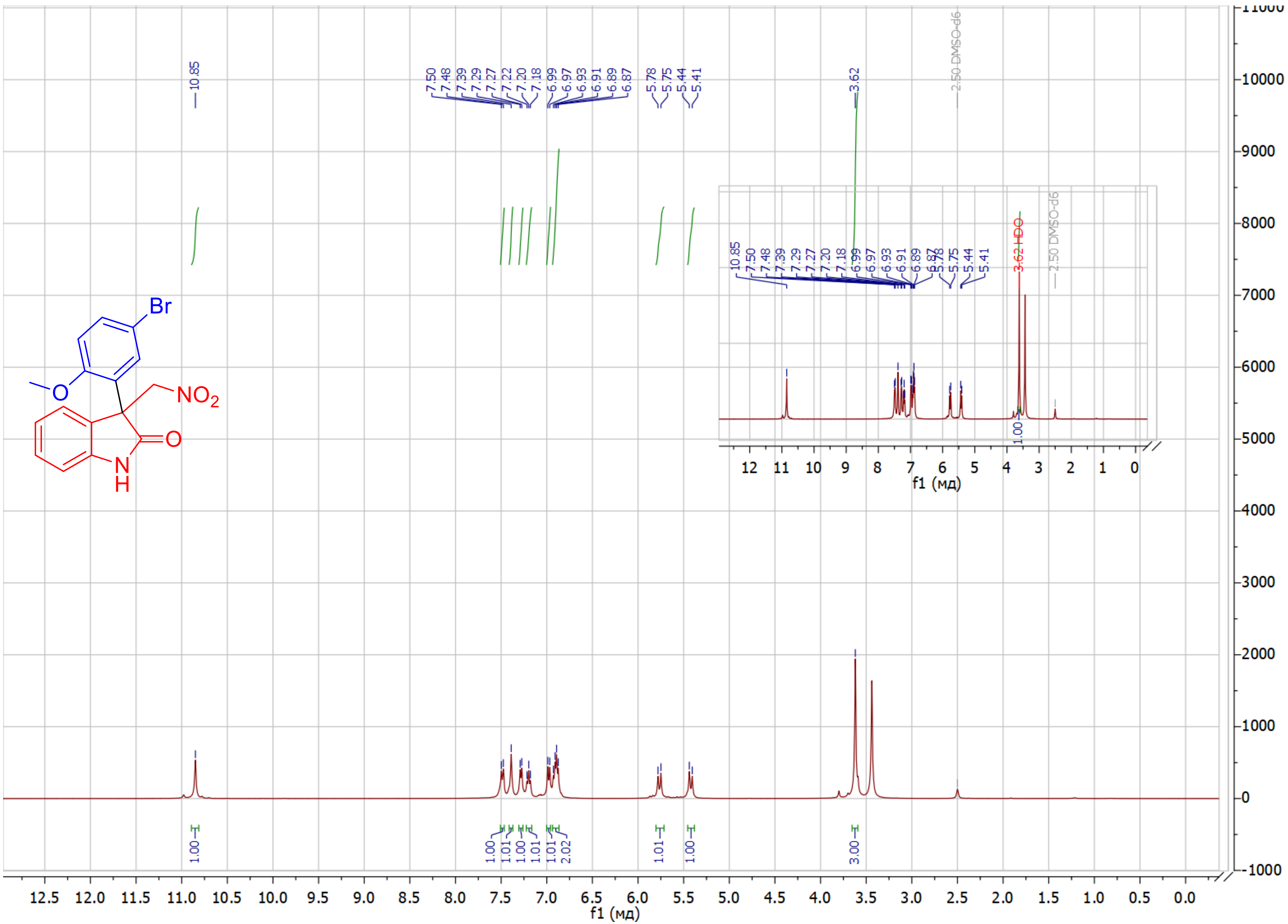

Figure S62. ^{1}H NMR spectrum of compound **7az** in DMSO-d_6 (400 MHz)

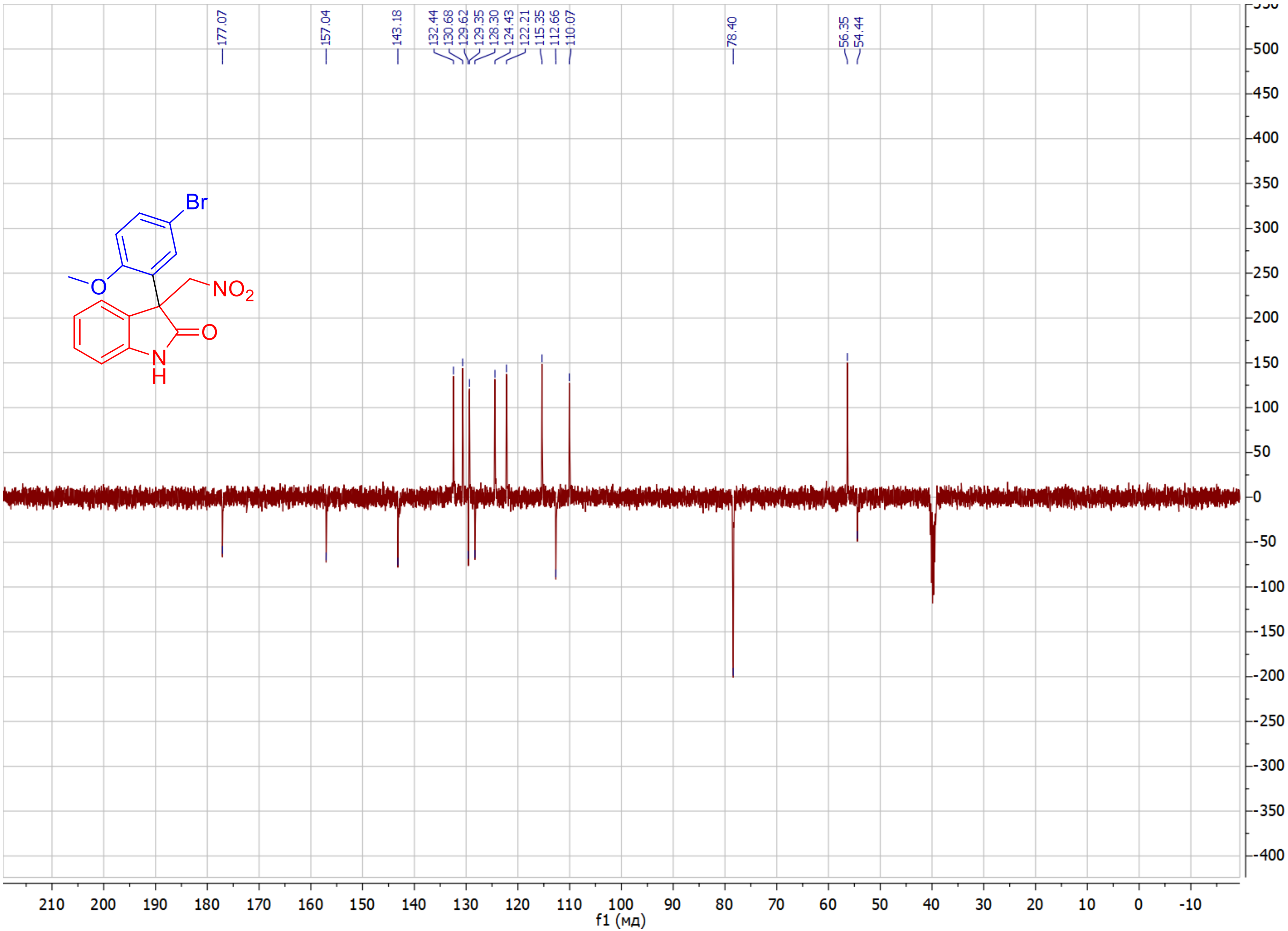

Figure S63. ^{13}C DEPTQ {^{1}H} NMR spectrum of compound **7ay** in DMSO-d_6 (101 MHz)

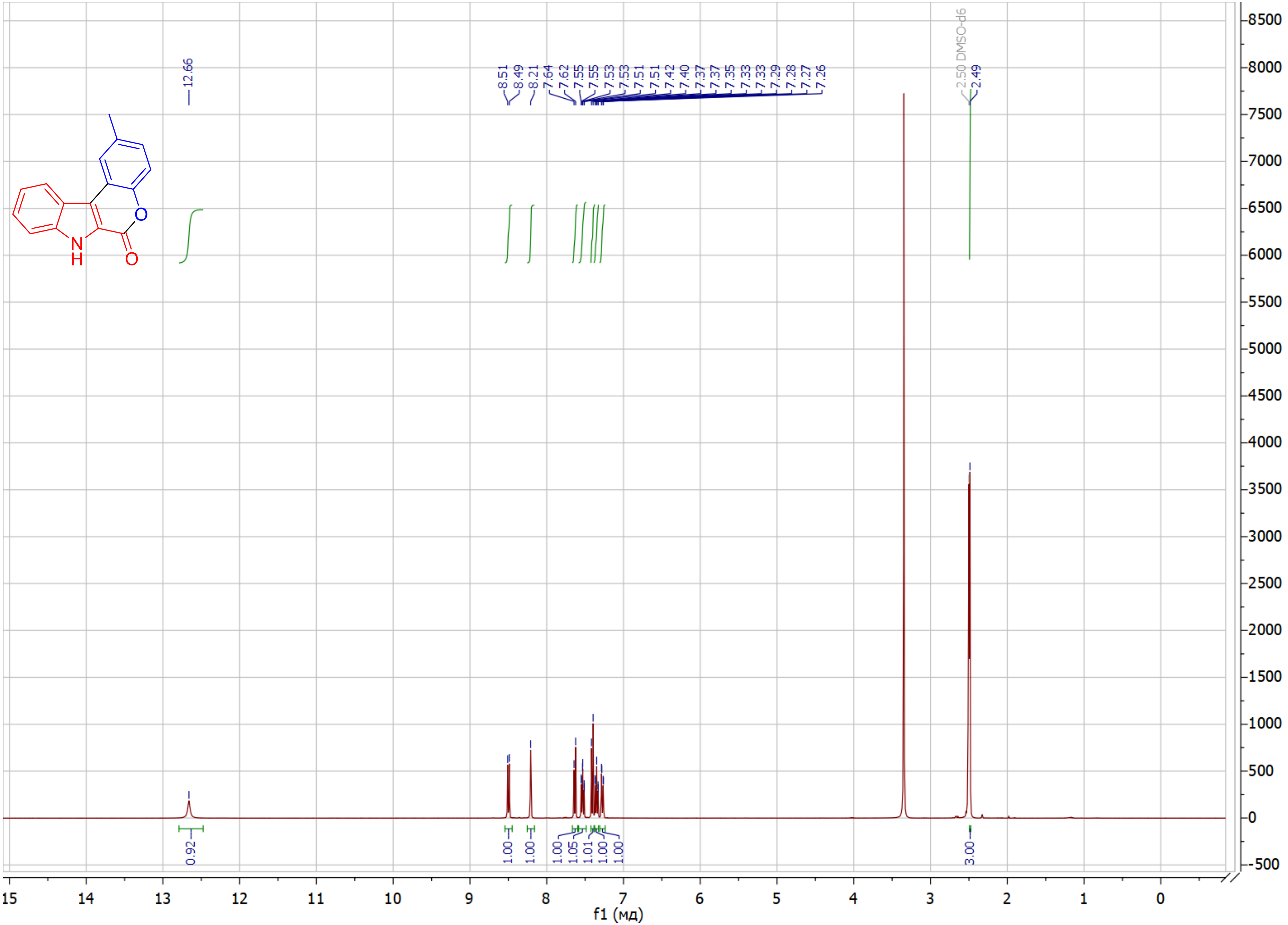

Figure S64. ^{1}H NMR spectrum of compound **8aa** in DMSO-d_6 (400 MHz)

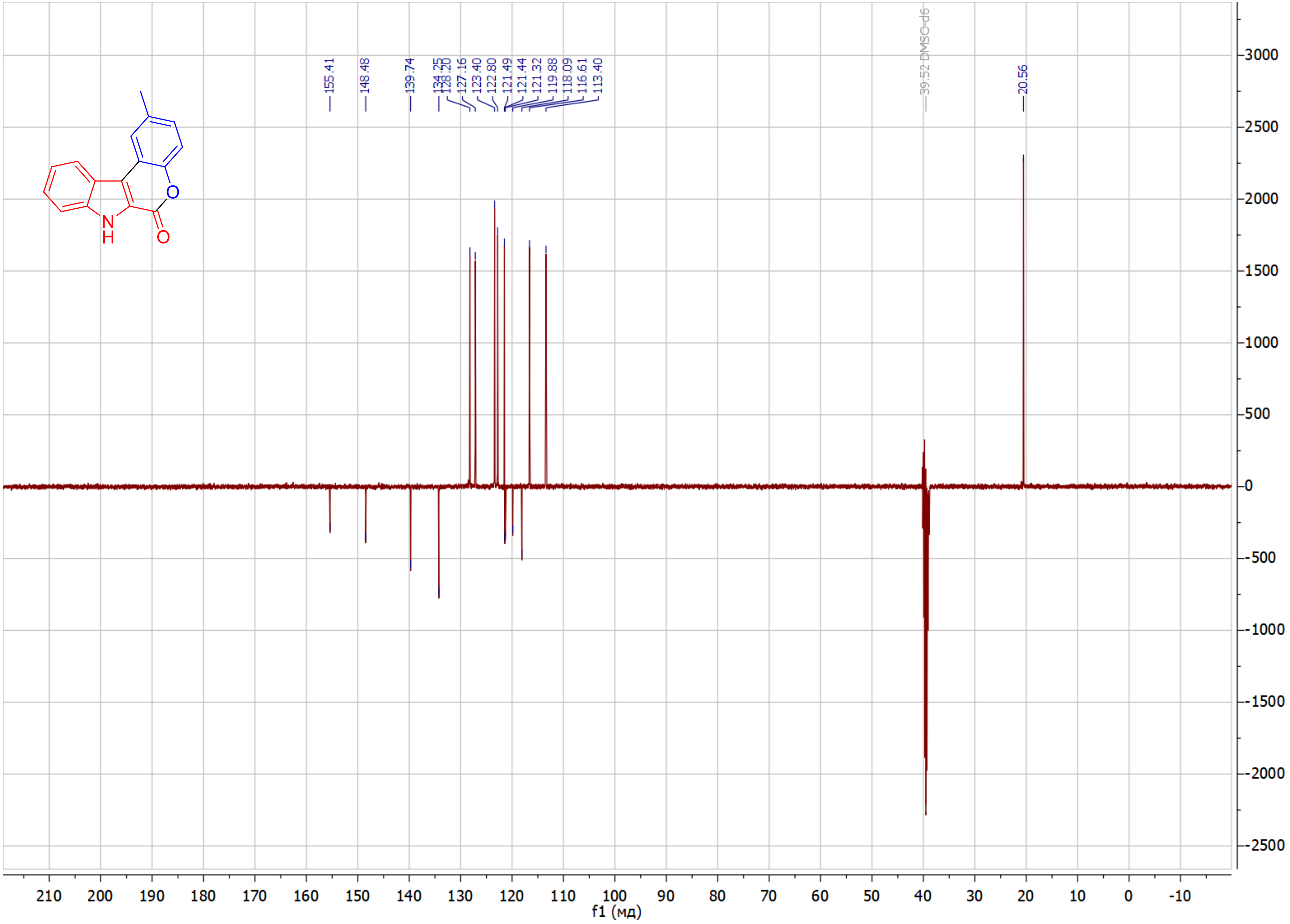

Figure S65. ^{13}C DEPTQ {^{1}H} NMR spectrum of compound **8aa** in DMSO-d_6 (101 MHz)

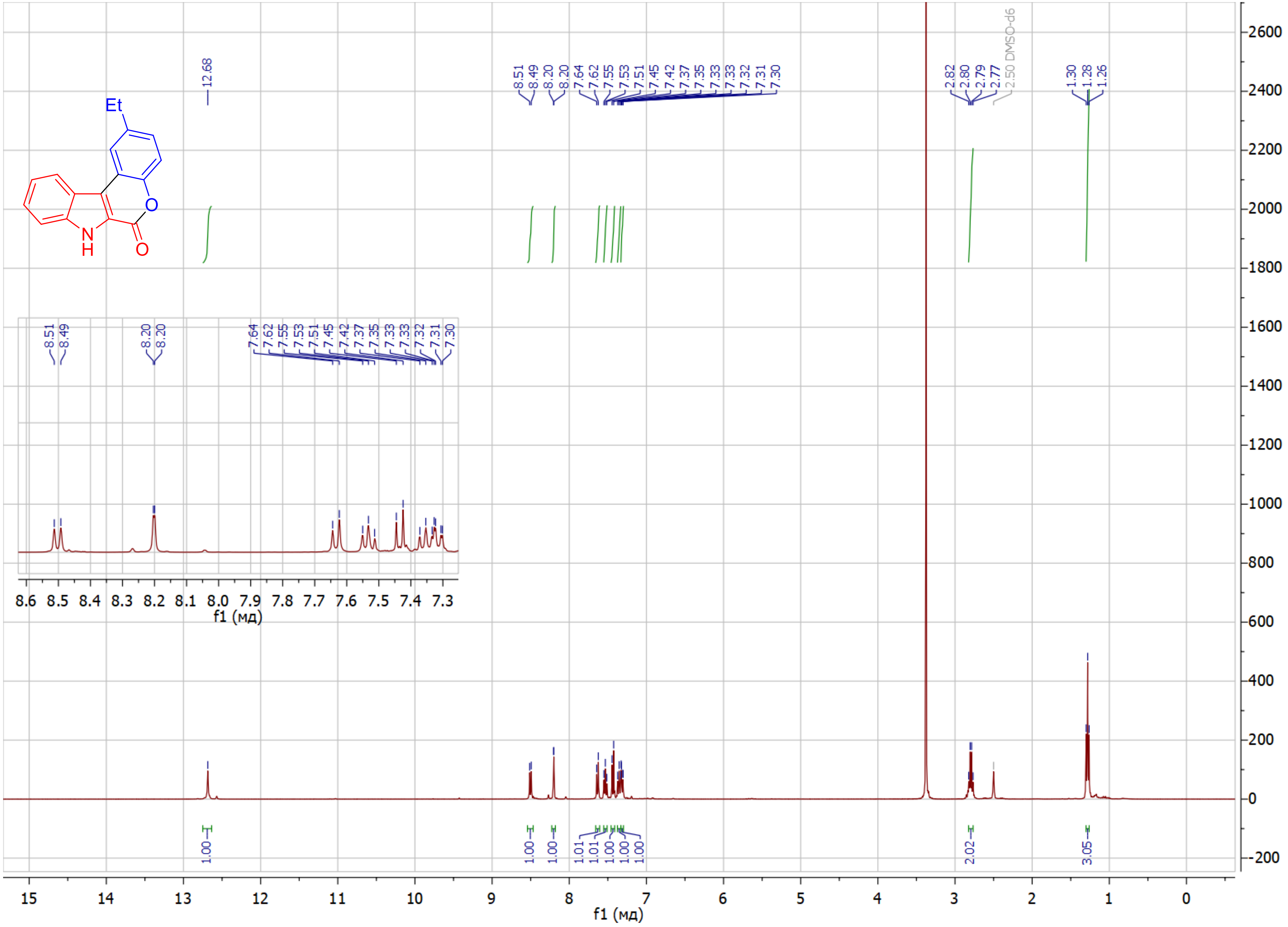

Figure S66. ^{1}H NMR spectrum of compound **8ab** in DMSO-d_6 (400 MHz)

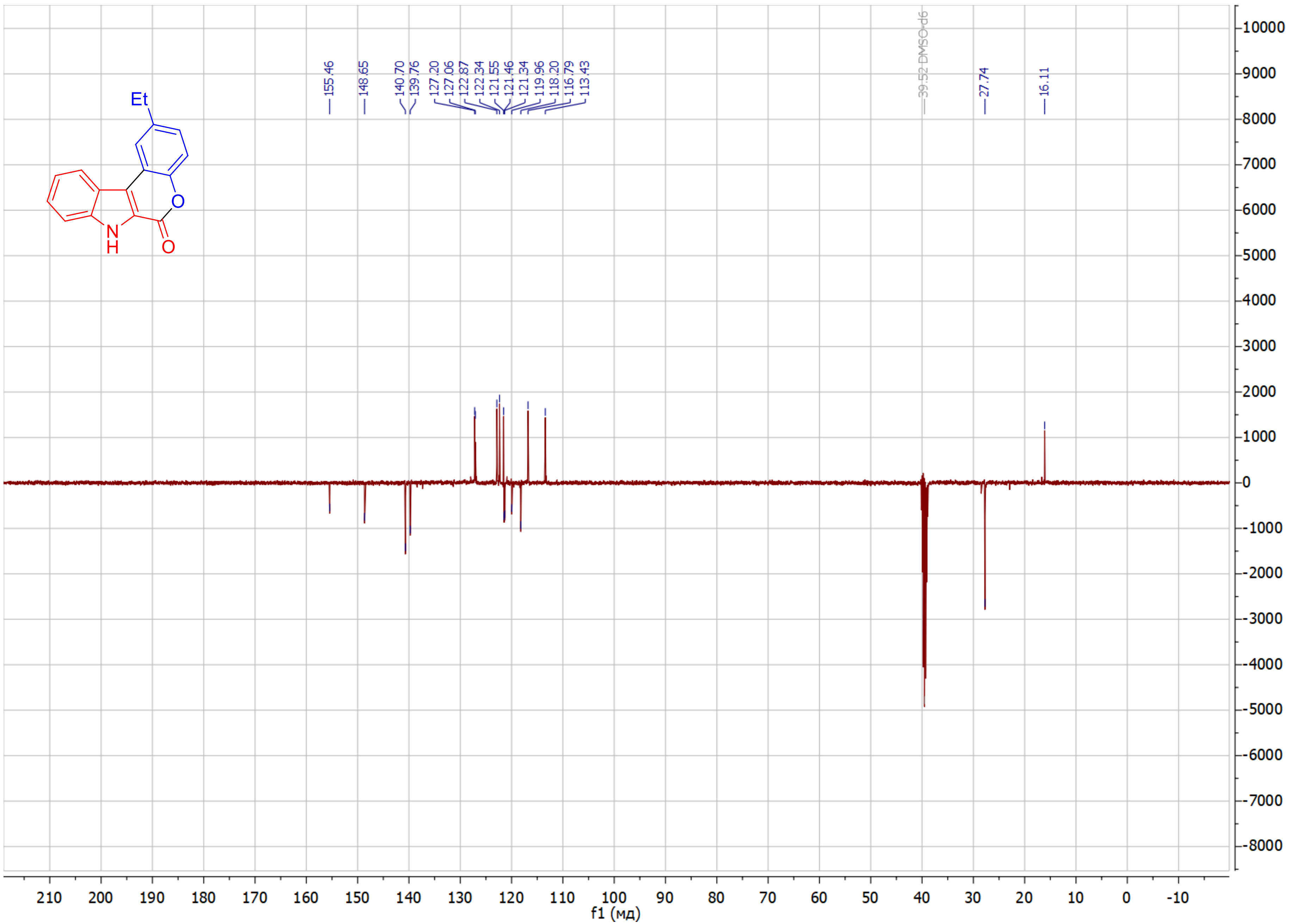

Figure S67. ^{13}C DEPTQ {^{1}H} NMR spectrum of compound **8ab** in DMSO-d_6 (101 MHz)

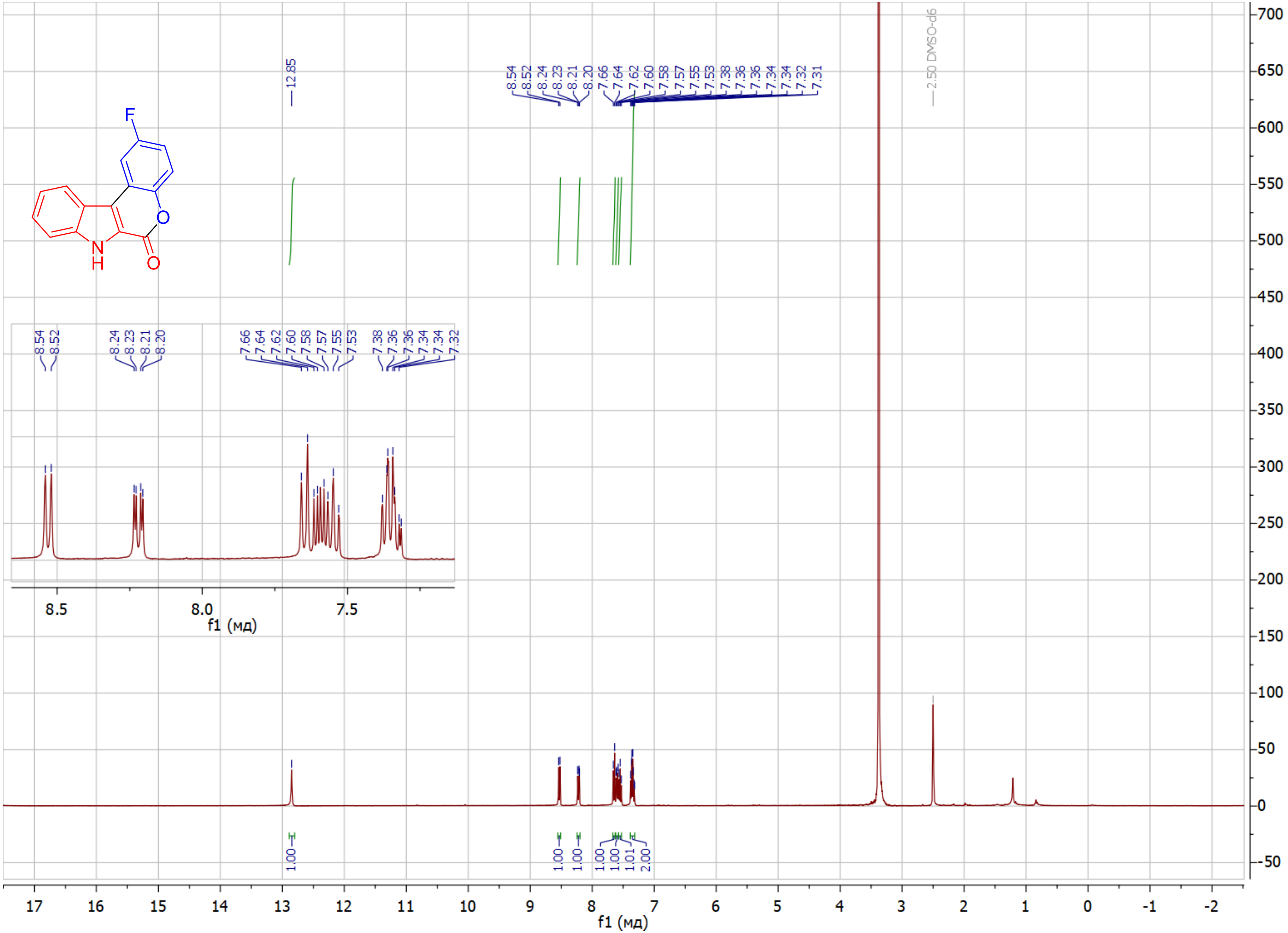

Figure S68. ^{1}H NMR spectrum of compound **8ai** in DMSO-d_6 (400 MHz)

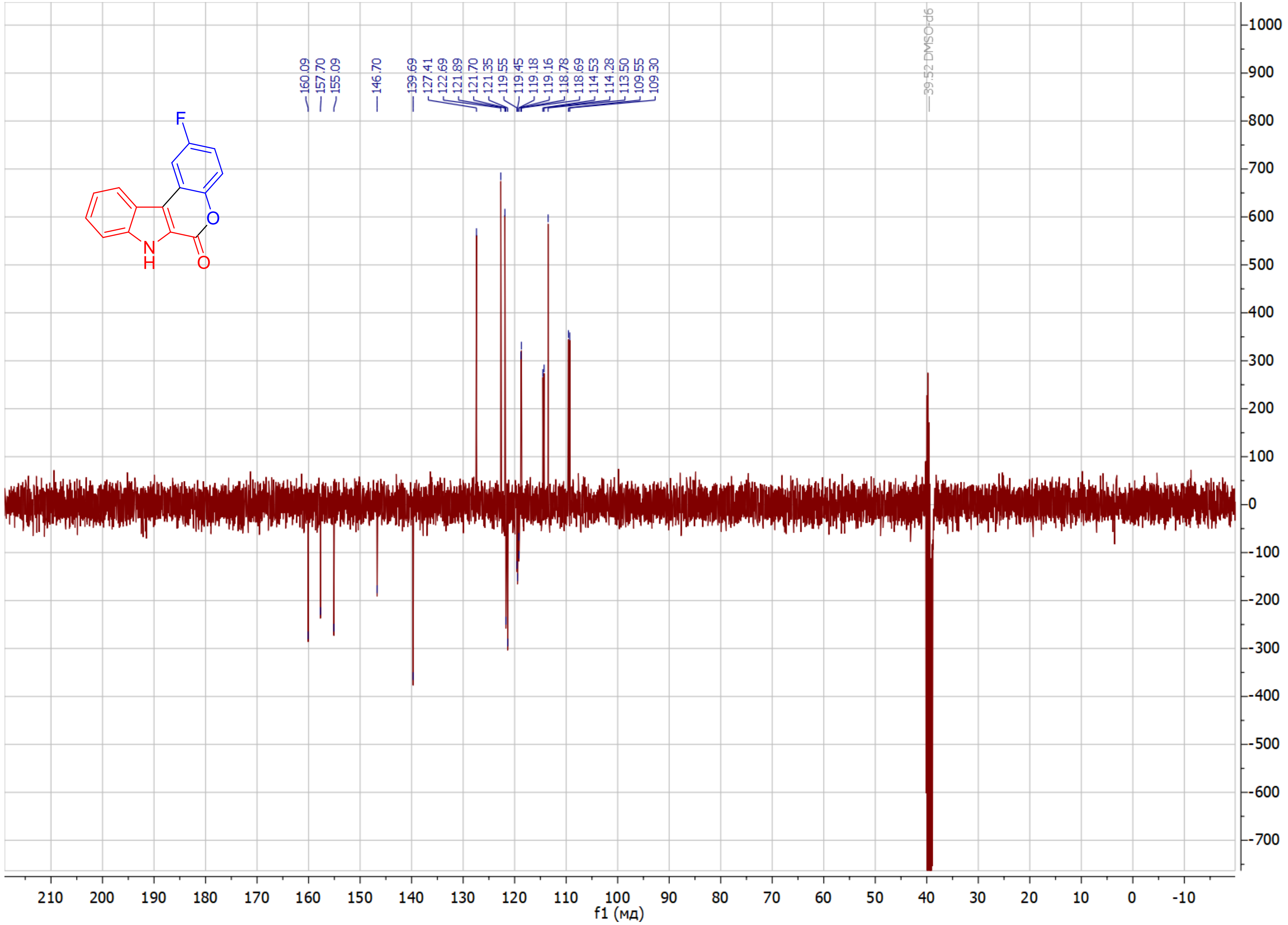

Figure S69. ^{13}C DEPTQ {^{1}H} NMR spectrum of compound **8ai** in DMSO-d_6 (101 MHz)

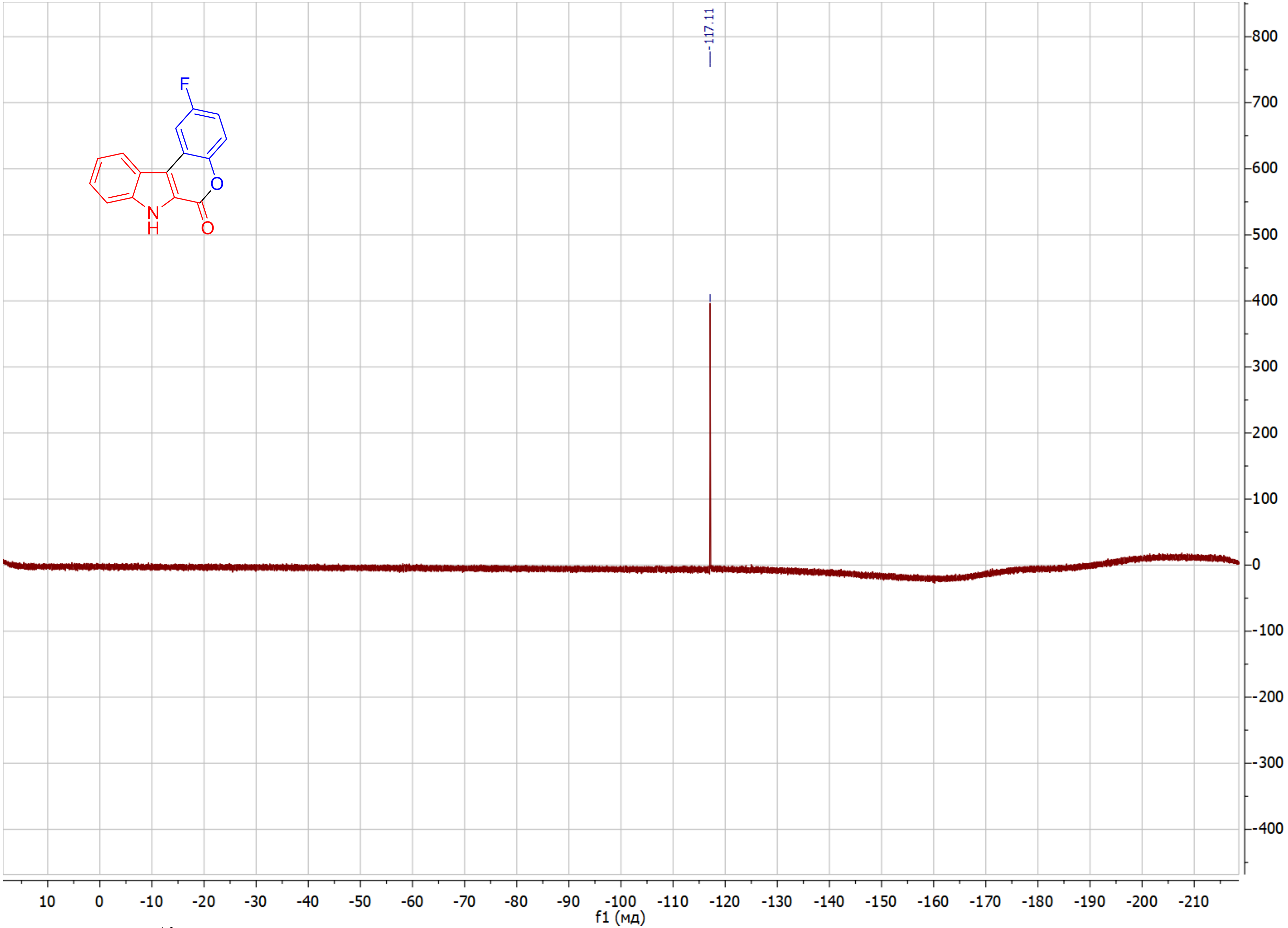

Figure S70. ^{19}F NMR spectrum of compound **8ai** in DMSO-d_6 (400 MHz)

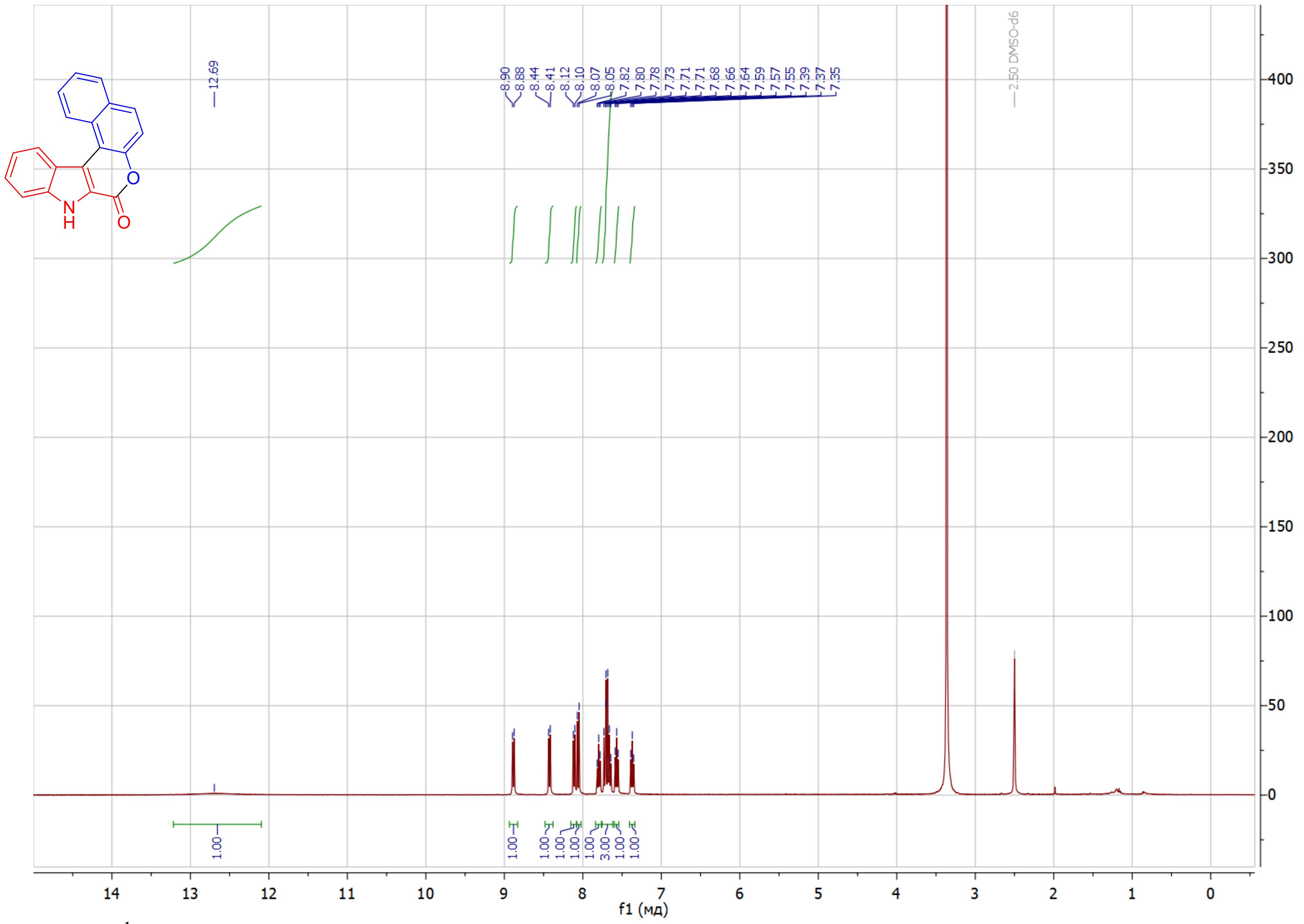

Figure S71. ^{1}H NMR spectrum of compound **8'az** in DMSO-d_6 (400 MHz)

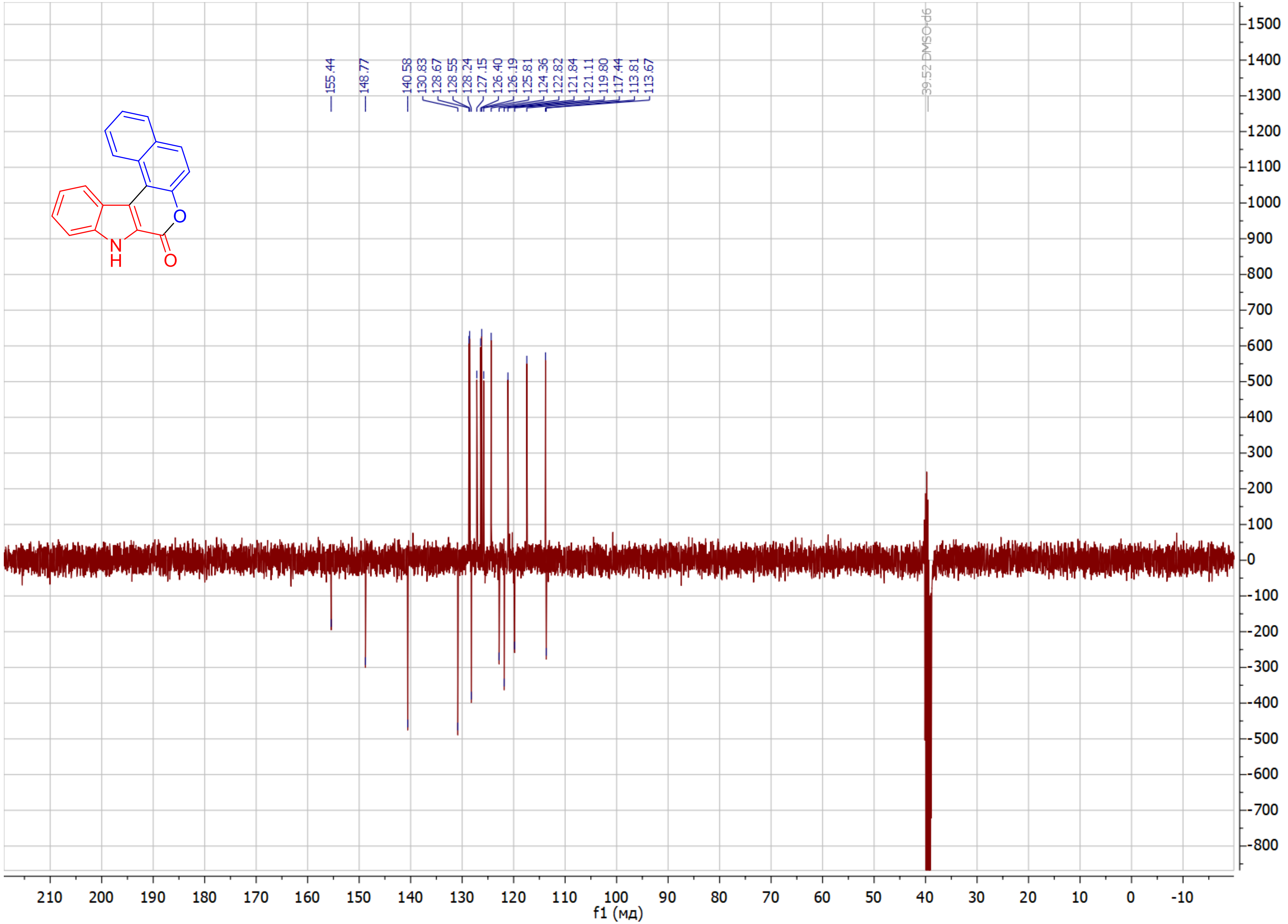

Figure S72. ^{13}C DEPTQ {^{1}H} NMR spectrum of compound **8’az** in DMSO-d_6 (101 MHz)

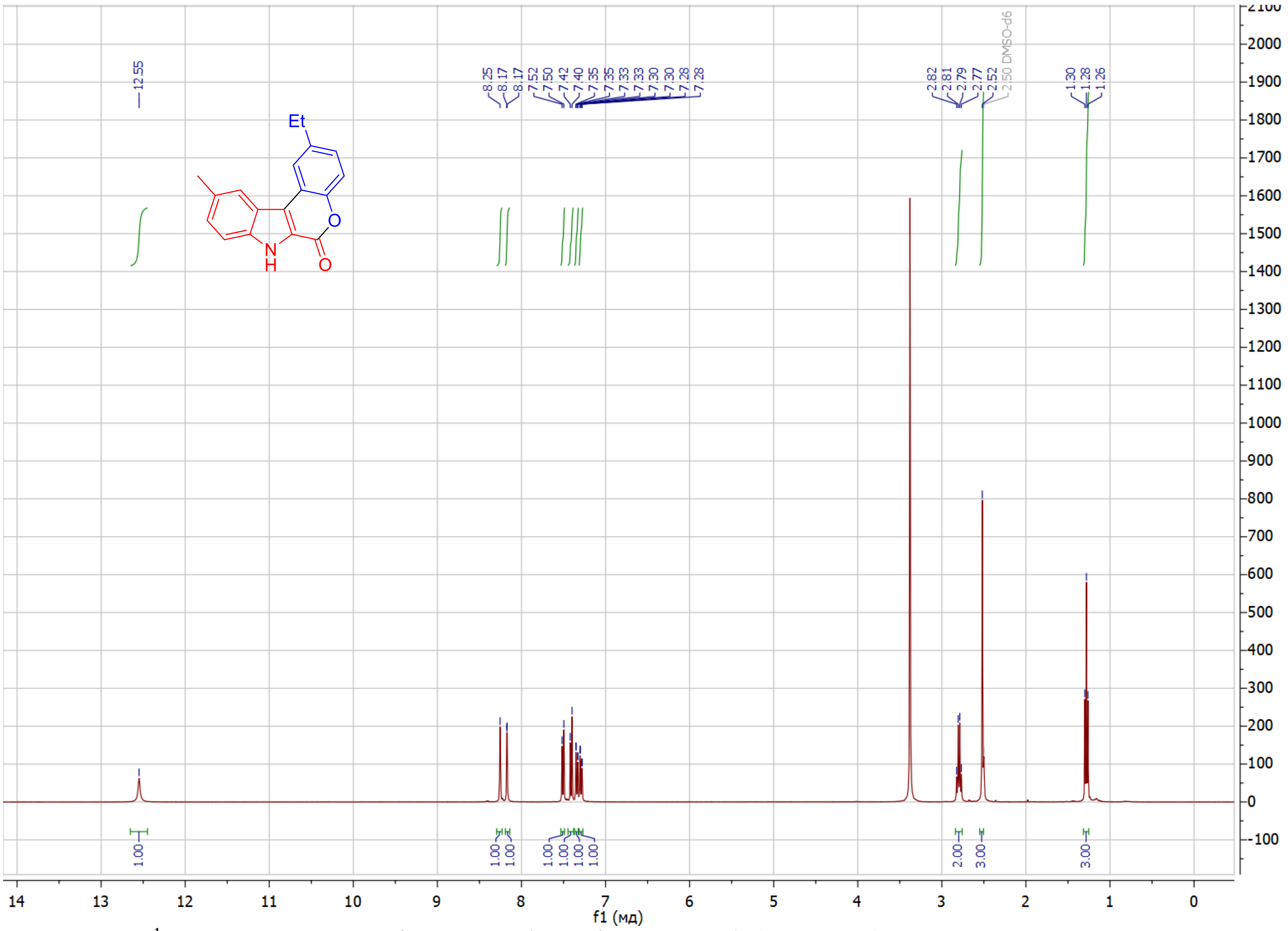

Figure S73. ^{1}H NMR spectrum of compound **8bb** in DMSO-d_6 (400 MHz)

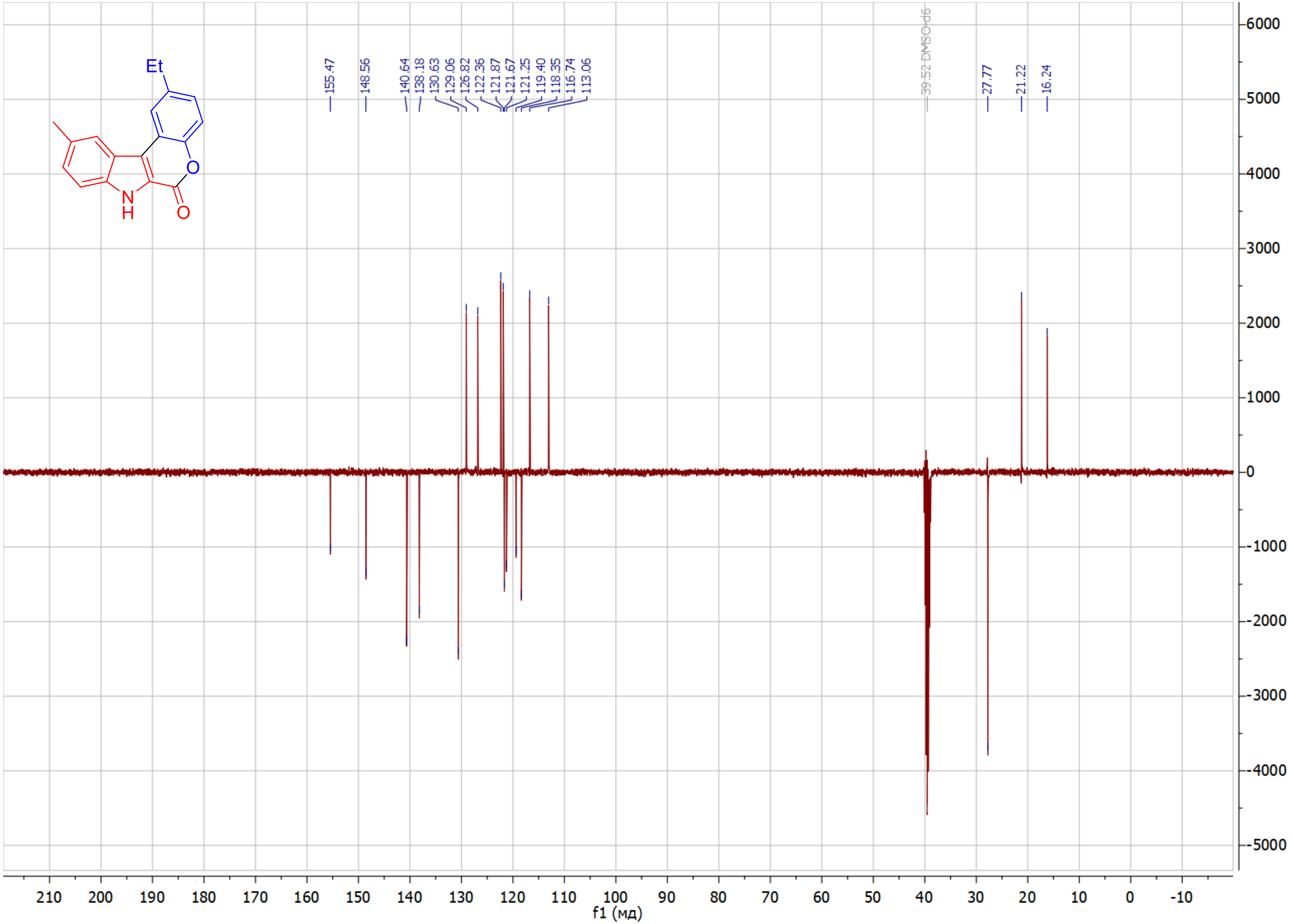

Figure S74. ^{13}C DEPTQ {^{1}H} NMR spectrum of compound **8bb** in DMSO-d_6 (101 MHz)

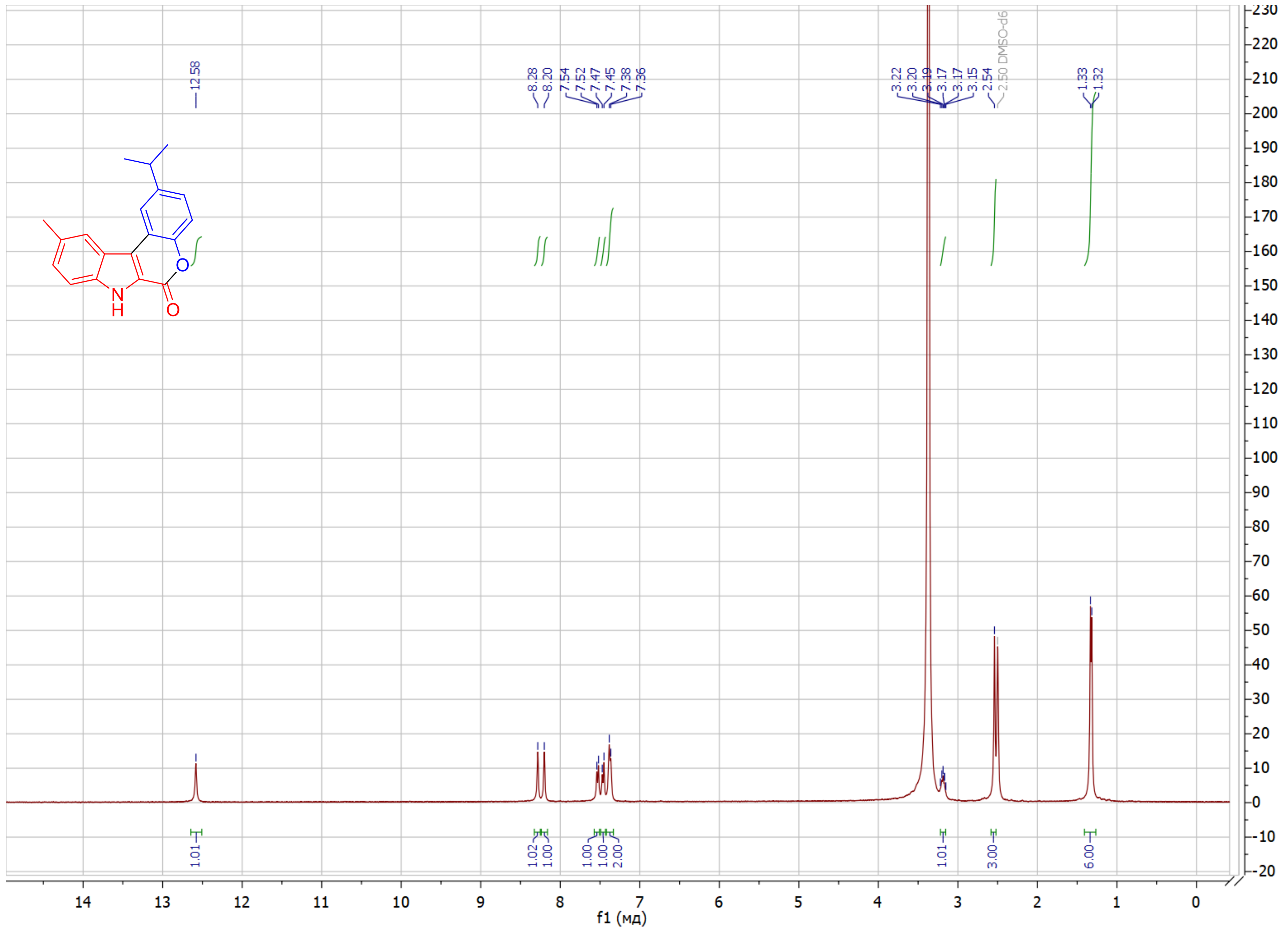

Figure S75. ^{1}H NMR spectrum of compound **8bd** in DMSO-d_6 (400 MHz)

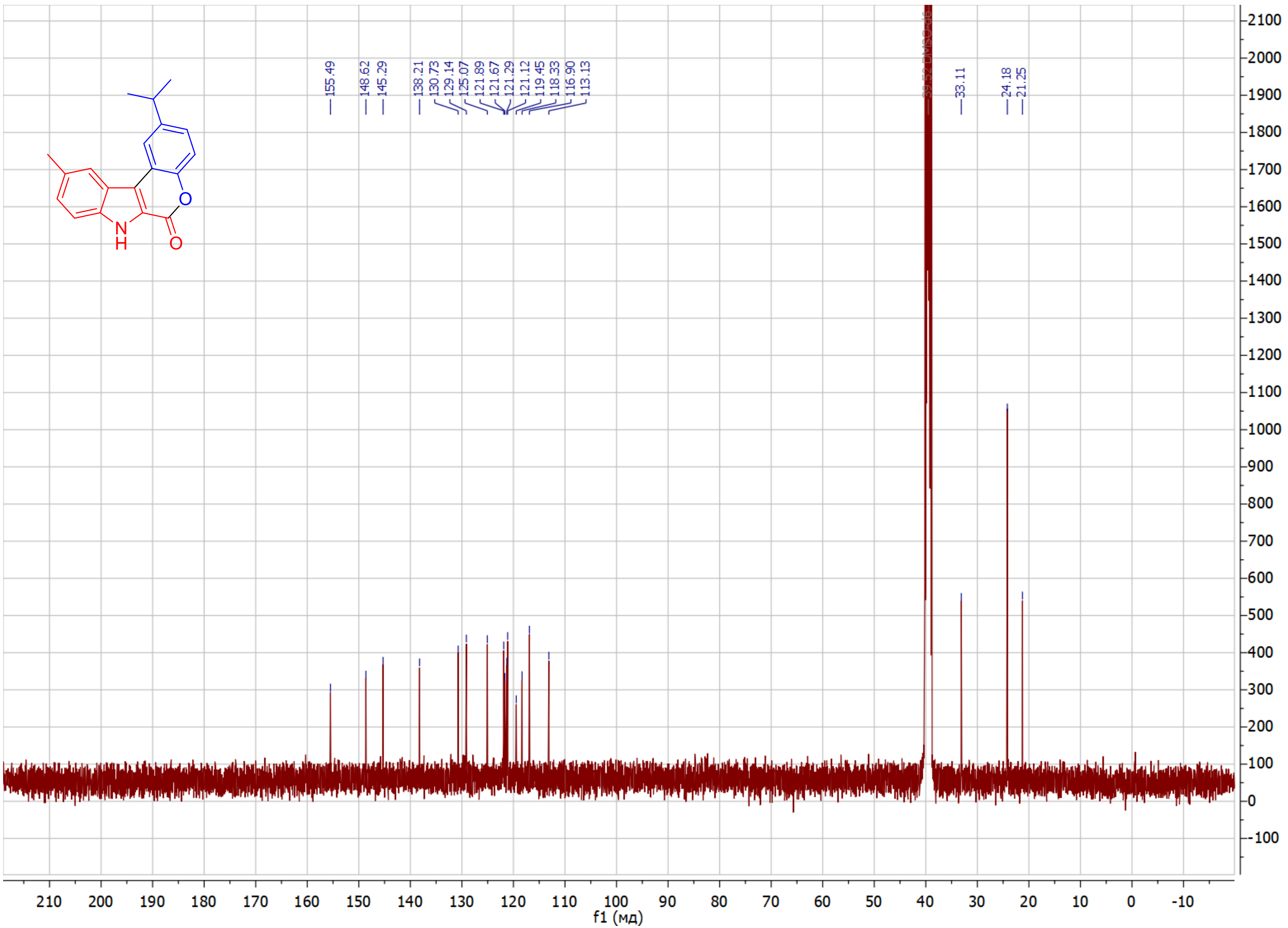

Figure S76. ^{13}C NMR spectrum of compound **8bd** in DMSO-d_6 (101 MHz)

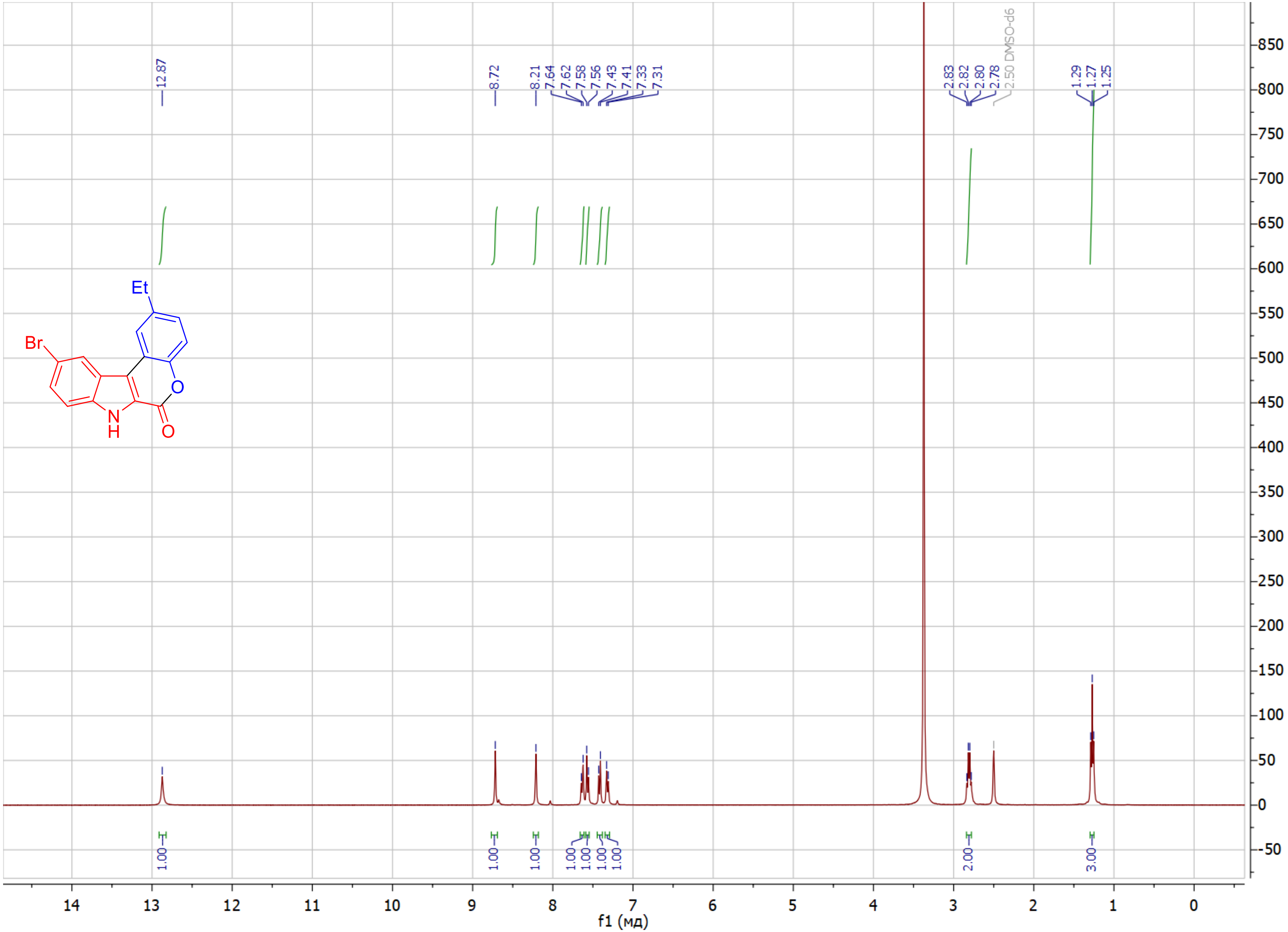

Figure S77. ¹H NMR spectrum of compound **8cb** in DMSO-d_6 (400 MHz)

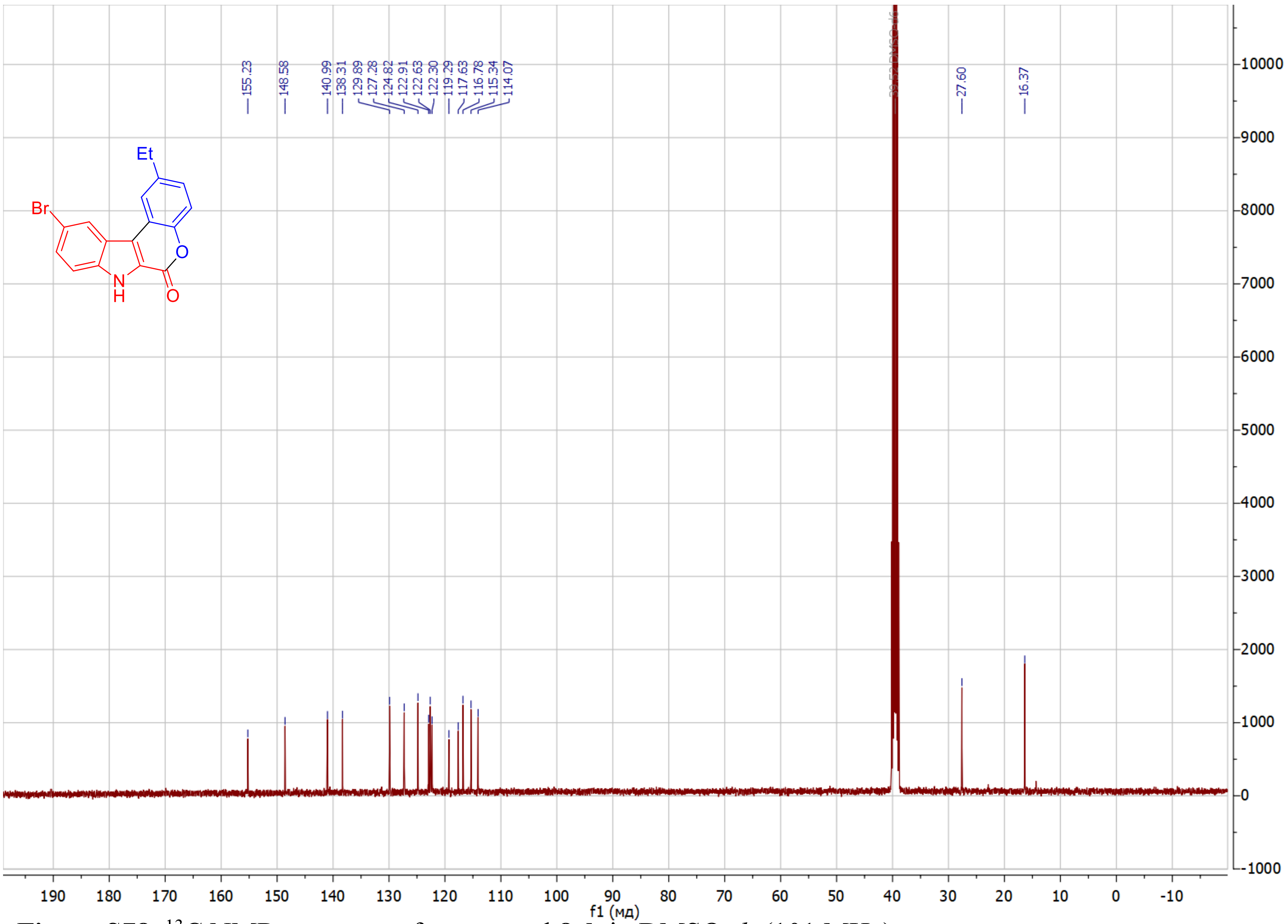

Figure S78. ^{13}C NMR spectrum of compound **8cb** in DMSO-d_6 (101 MHz)

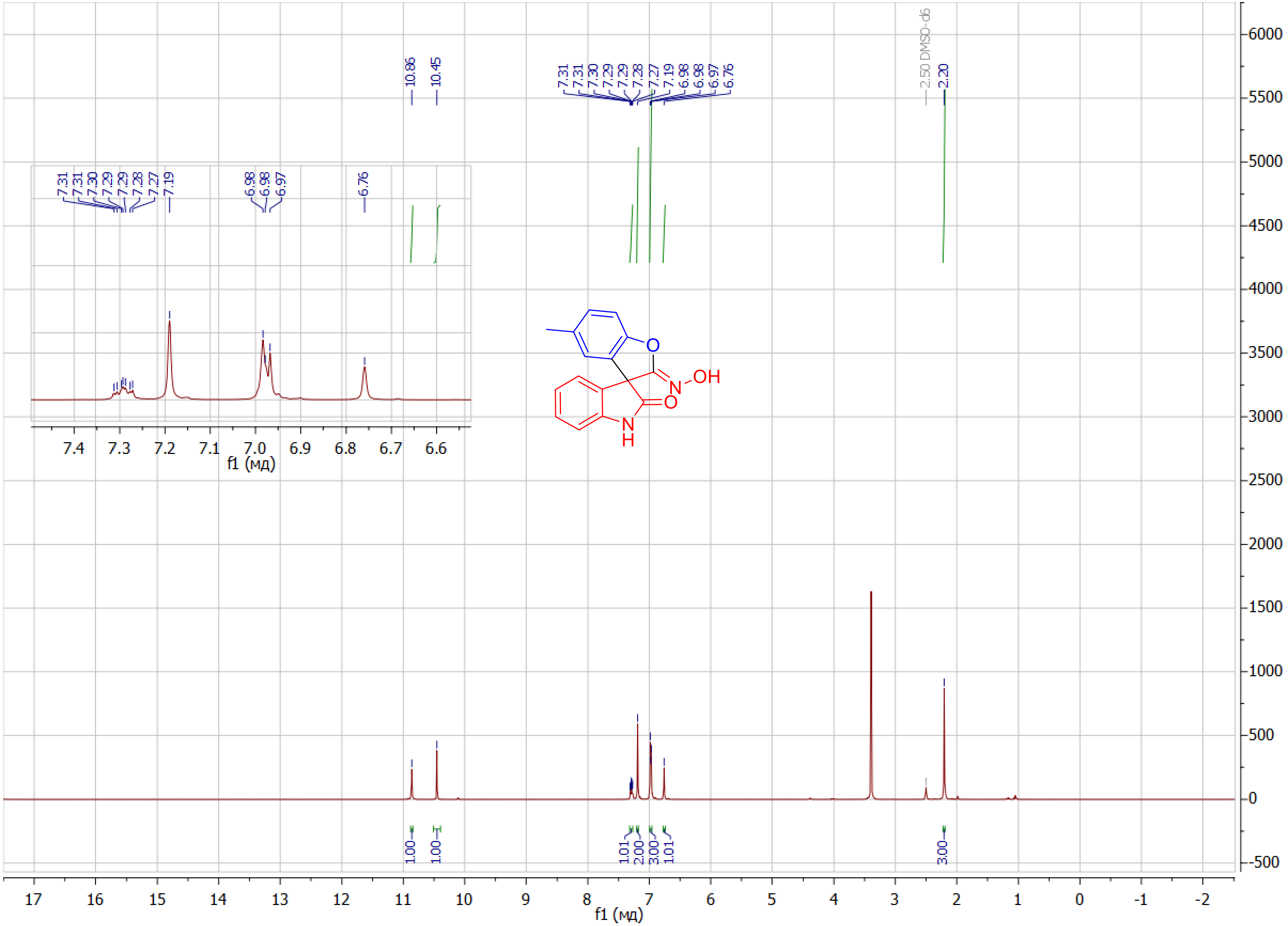

Figure S79. ^{1}H NMR spectrum of compound **13aa** in DMSO-d_6 (400 MHz)

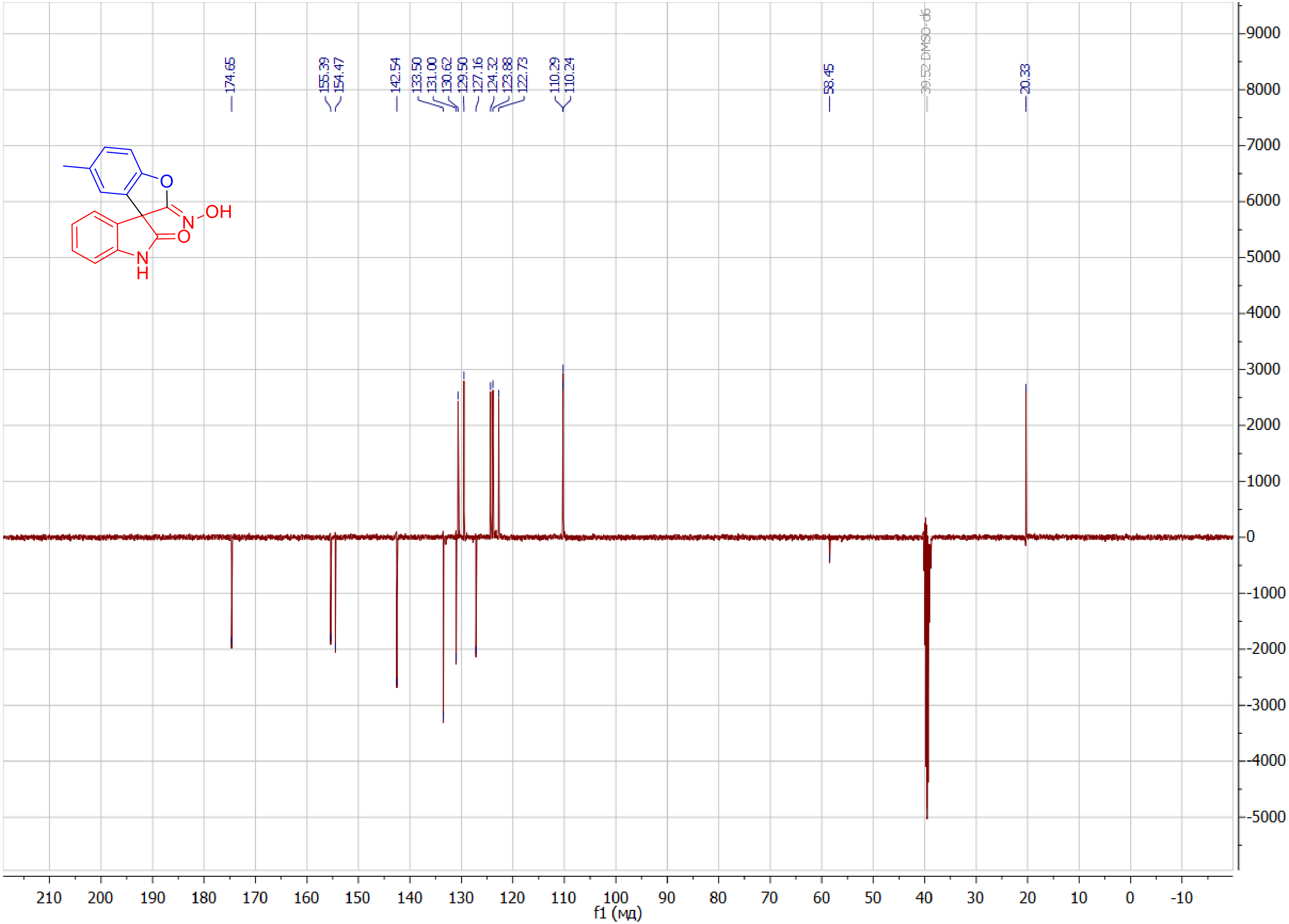

Figure S80. ^{13}C DEPTQ {^{1}H} NMR spectrum of compound **13aa** in DMSO-d_6 (101 MHz)

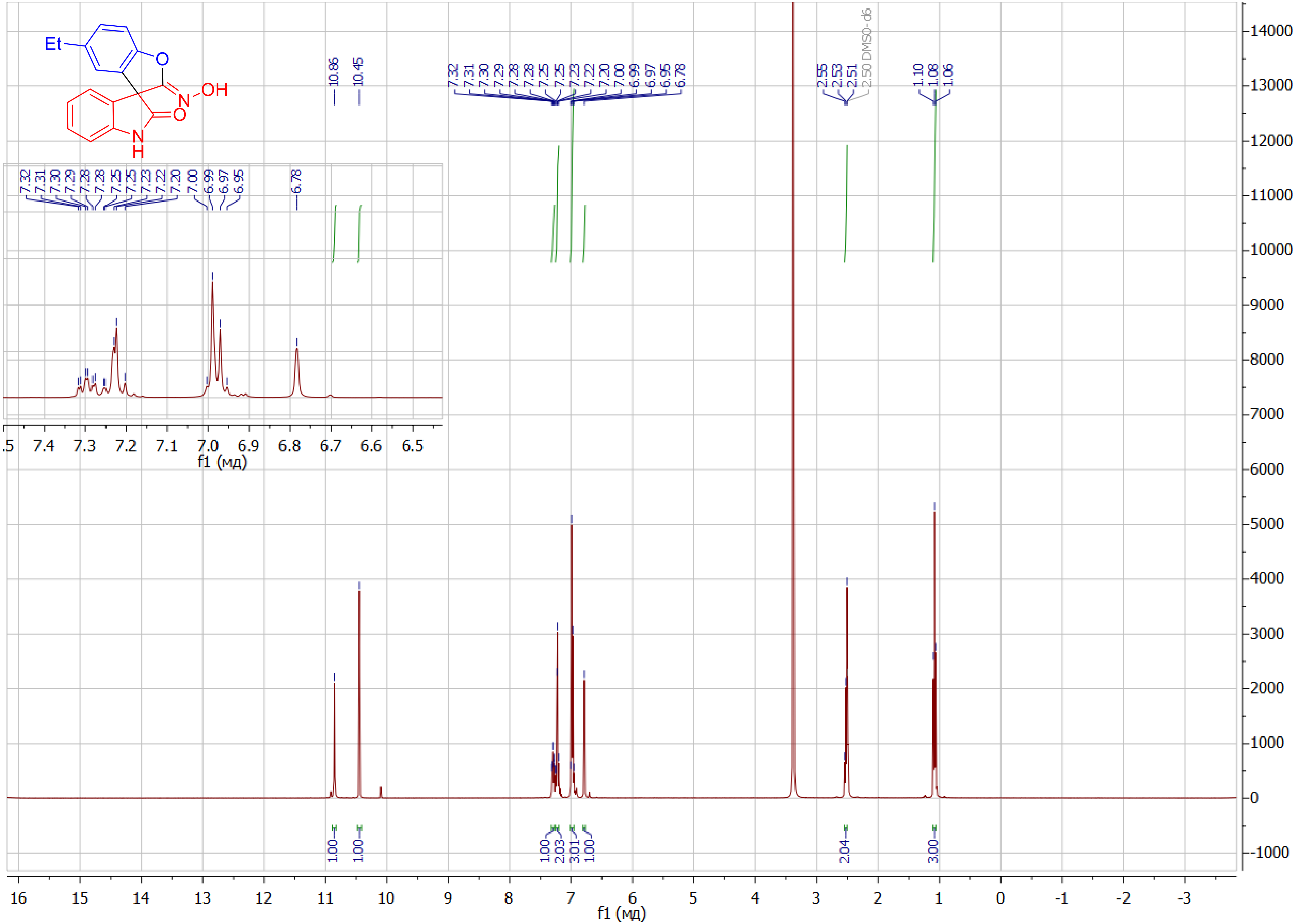

Figure S81. ^{1}H NMR spectrum of compound **13ab** in DMSO-d_6 (400 MHz)

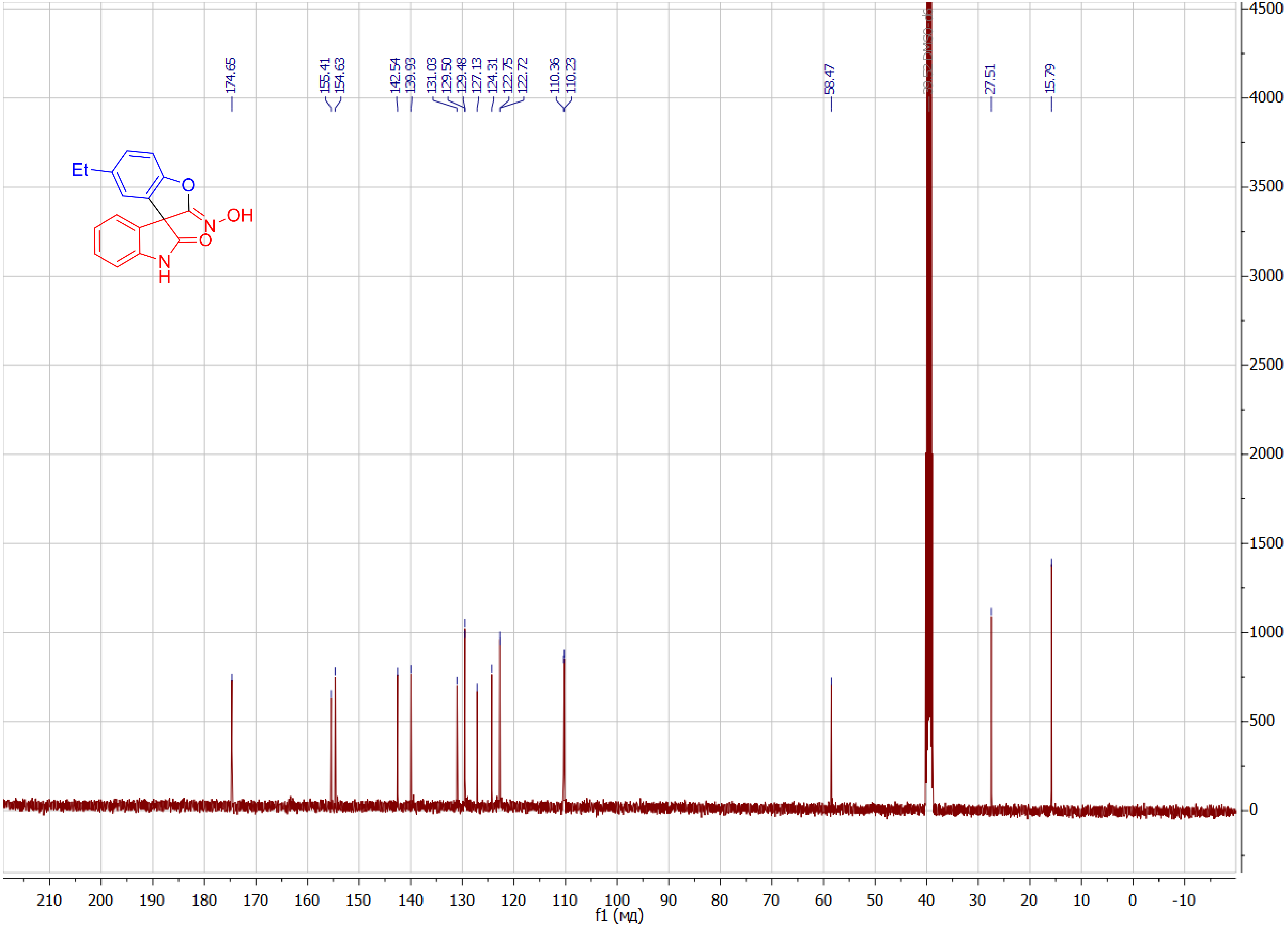

Figure S82. ^{13}C NMR spectrum of compound **13ab** in DMSO-d_6 (101 MHz)

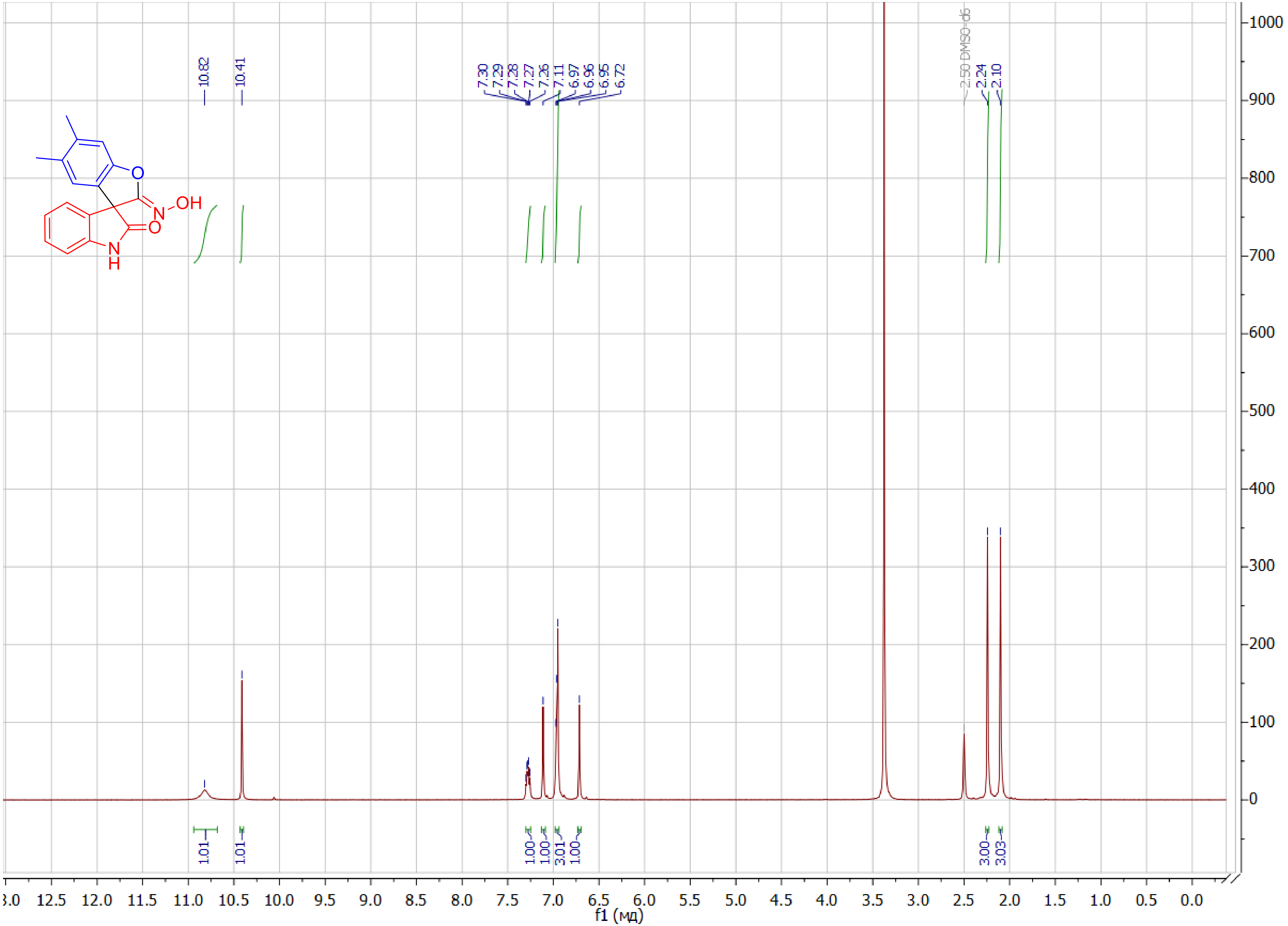

Figure S83. ^{1}H NMR spectrum of compound **13ac** in DMSO-d_6 (400 MHz)

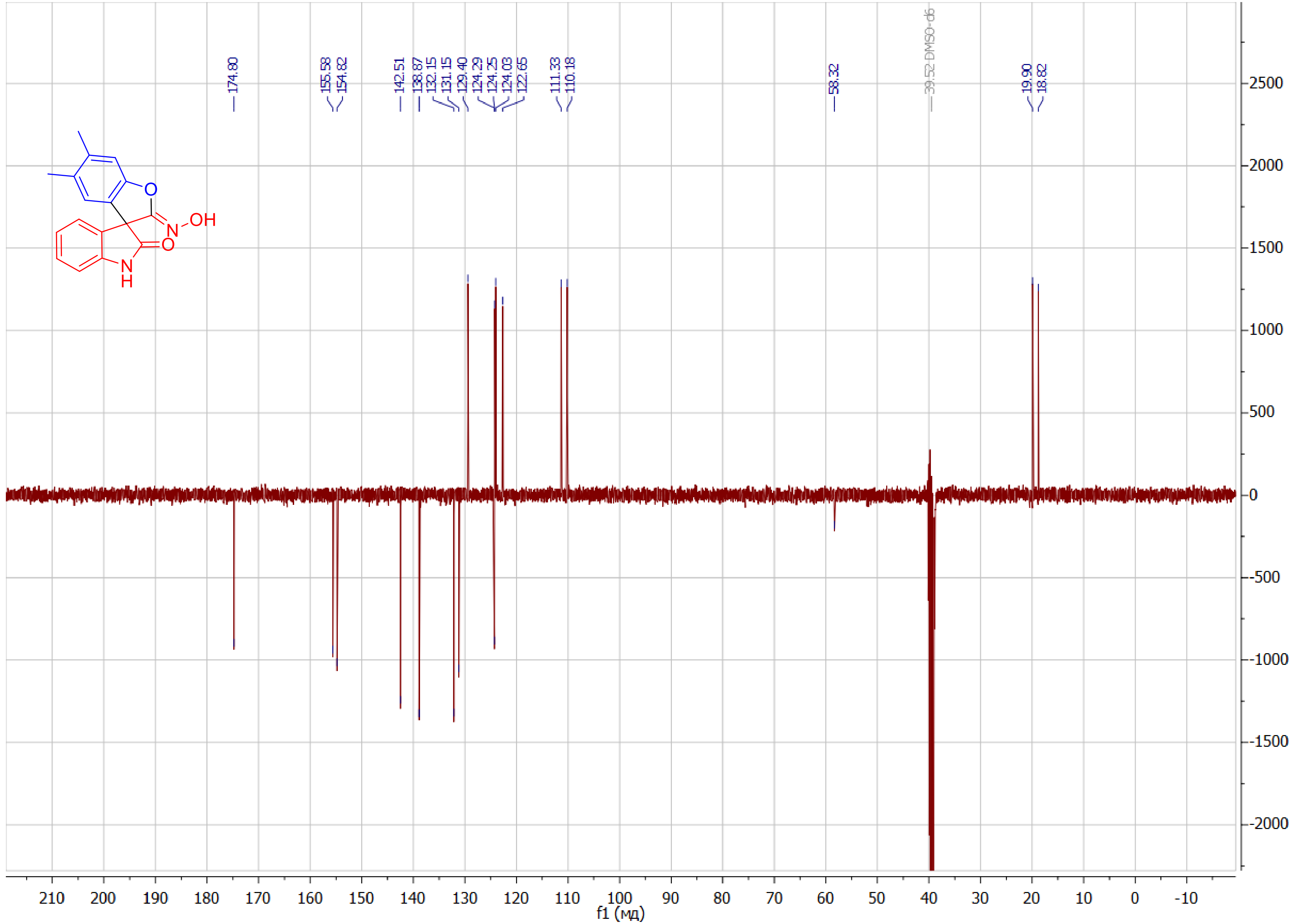

Figure S84. ^{13}C DEPTQ {^{1}H} NMR spectrum of compound **13ac** in DMSO-d_6 (101 MHz)

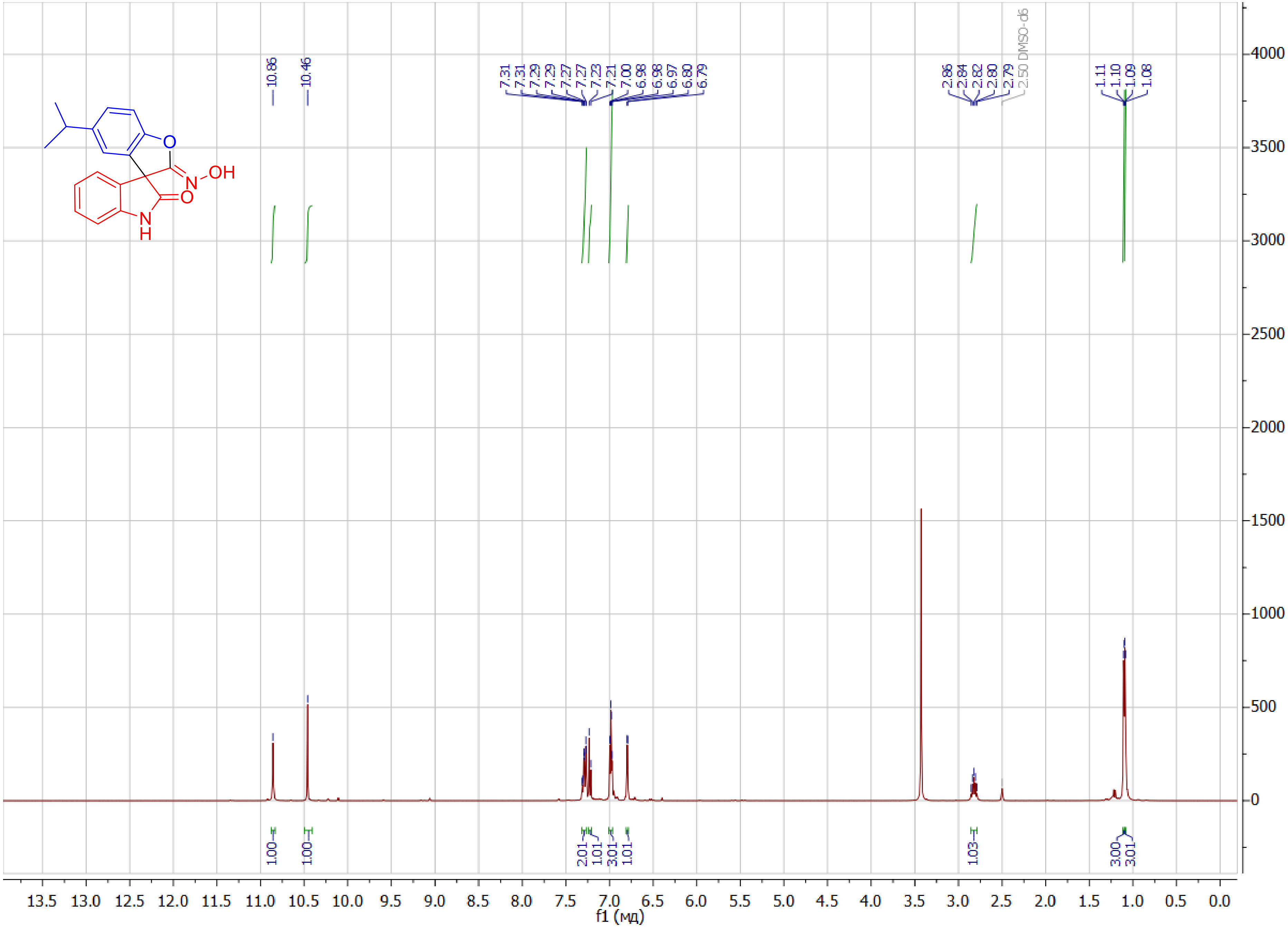

Figure S85. ^{1}H NMR spectrum of compound **13ad** in DMSO-d_6 (400 MHz)

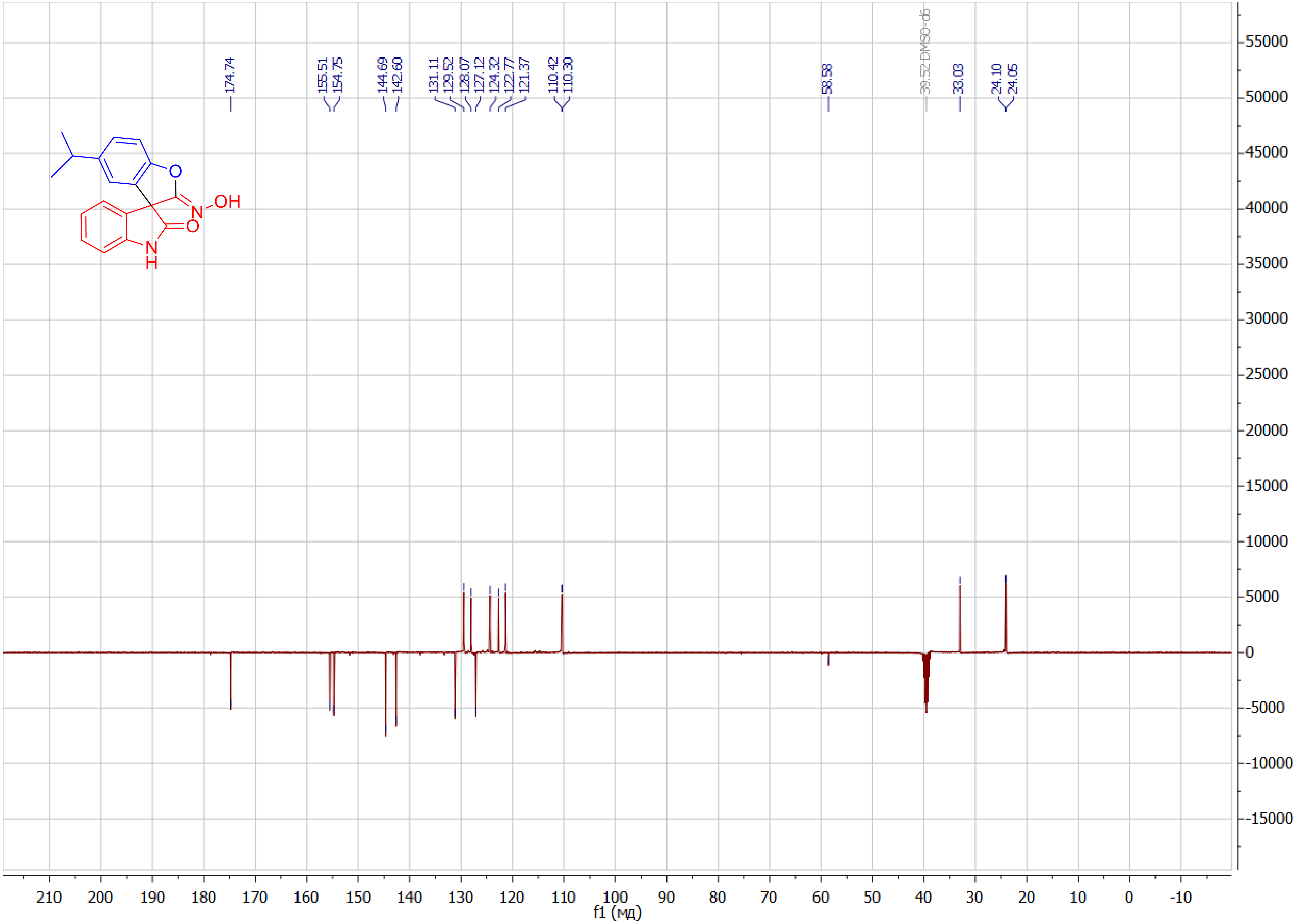

Figure S86. ^{13}C DEPTQ {^{1}H} NMR spectrum of compound **13ad** in DMSO-d_6 (101 MHz)

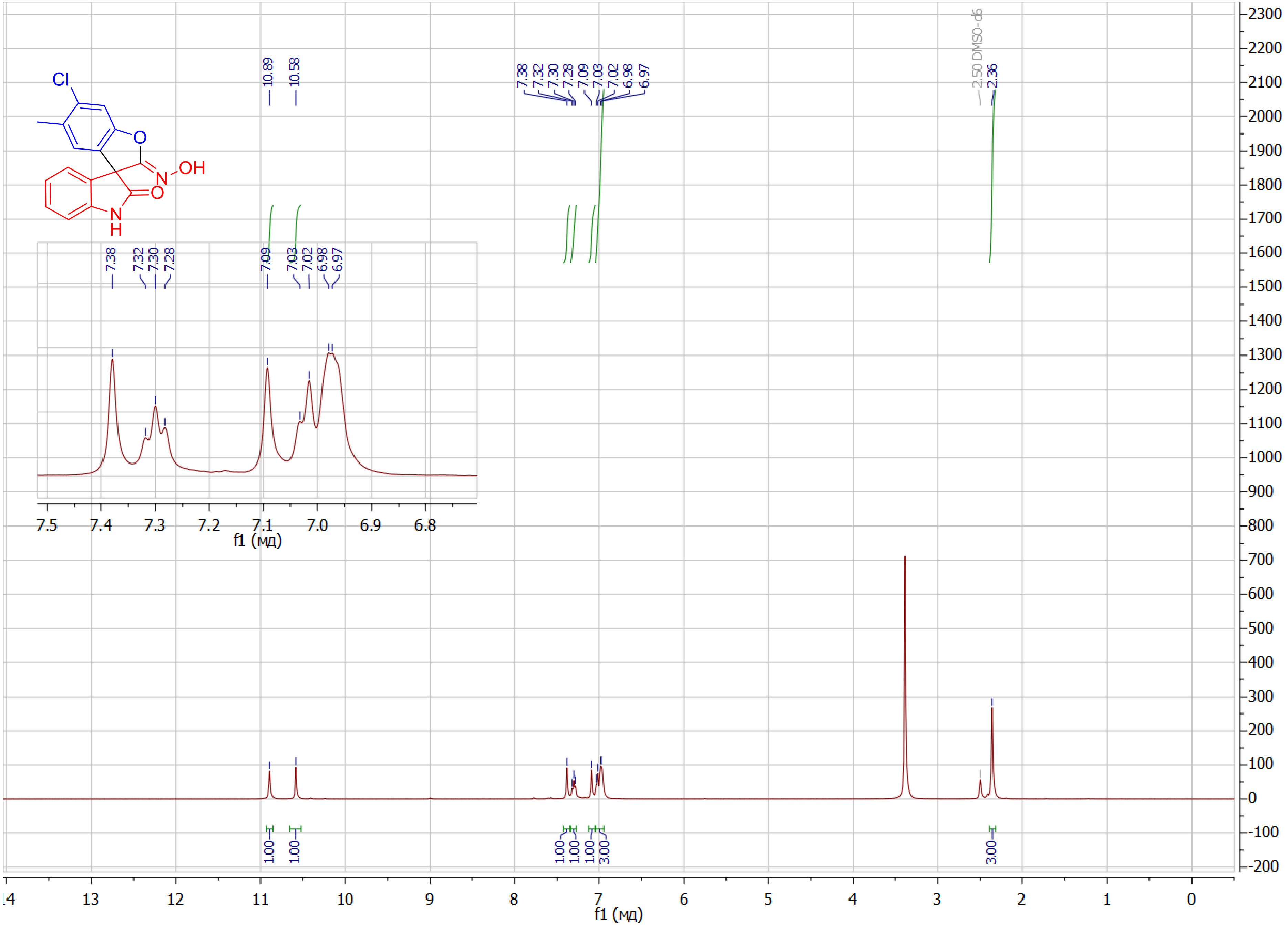

Figure S87. ^{1}H NMR spectrum of compound **13ae** in DMSO-d_6 (400 MHz)

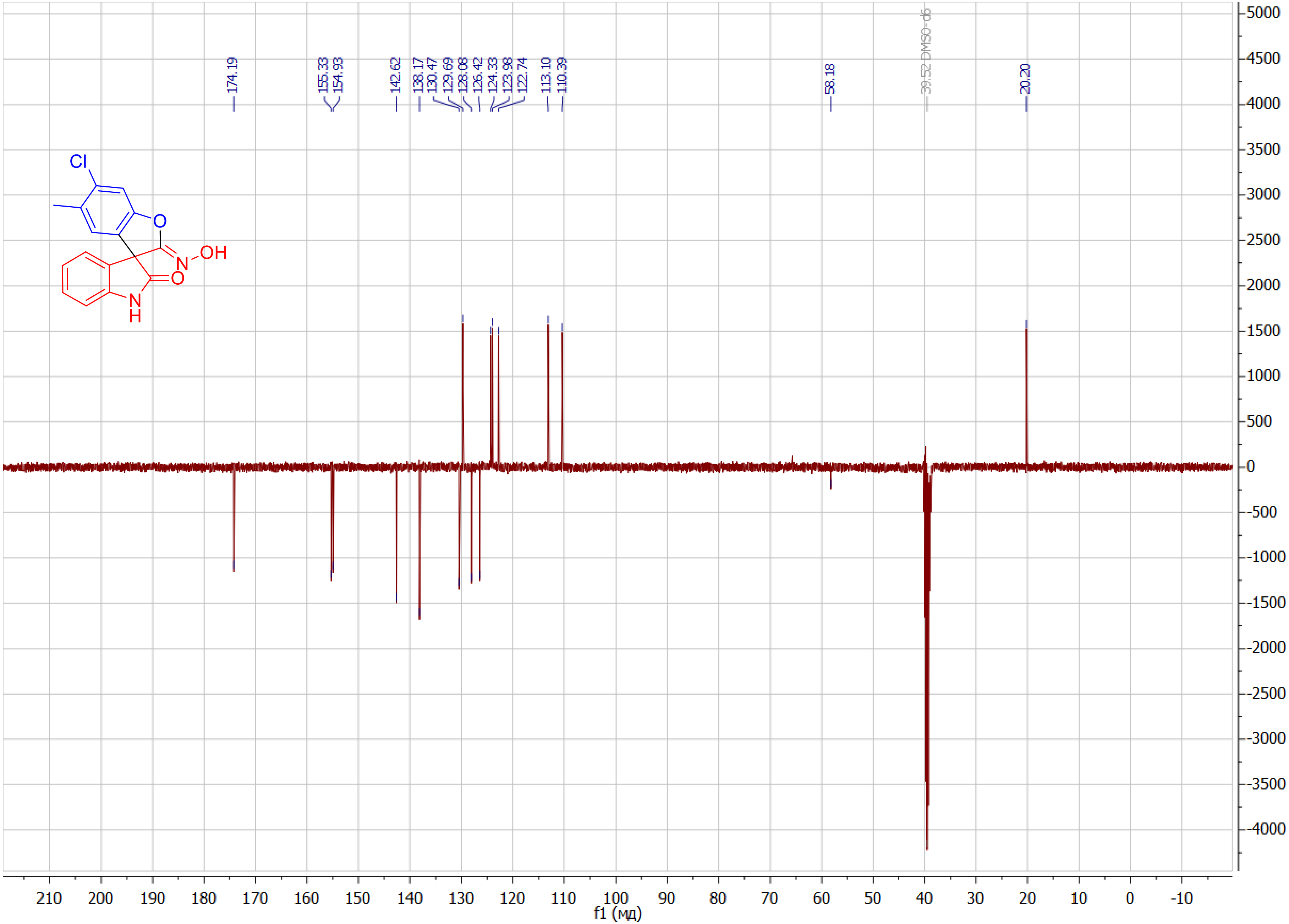

Figure S88. ^{13}C DEPTQ {^{1}H} NMR spectrum of compound **13ae** in DMSO-d_6 (101 MHz)

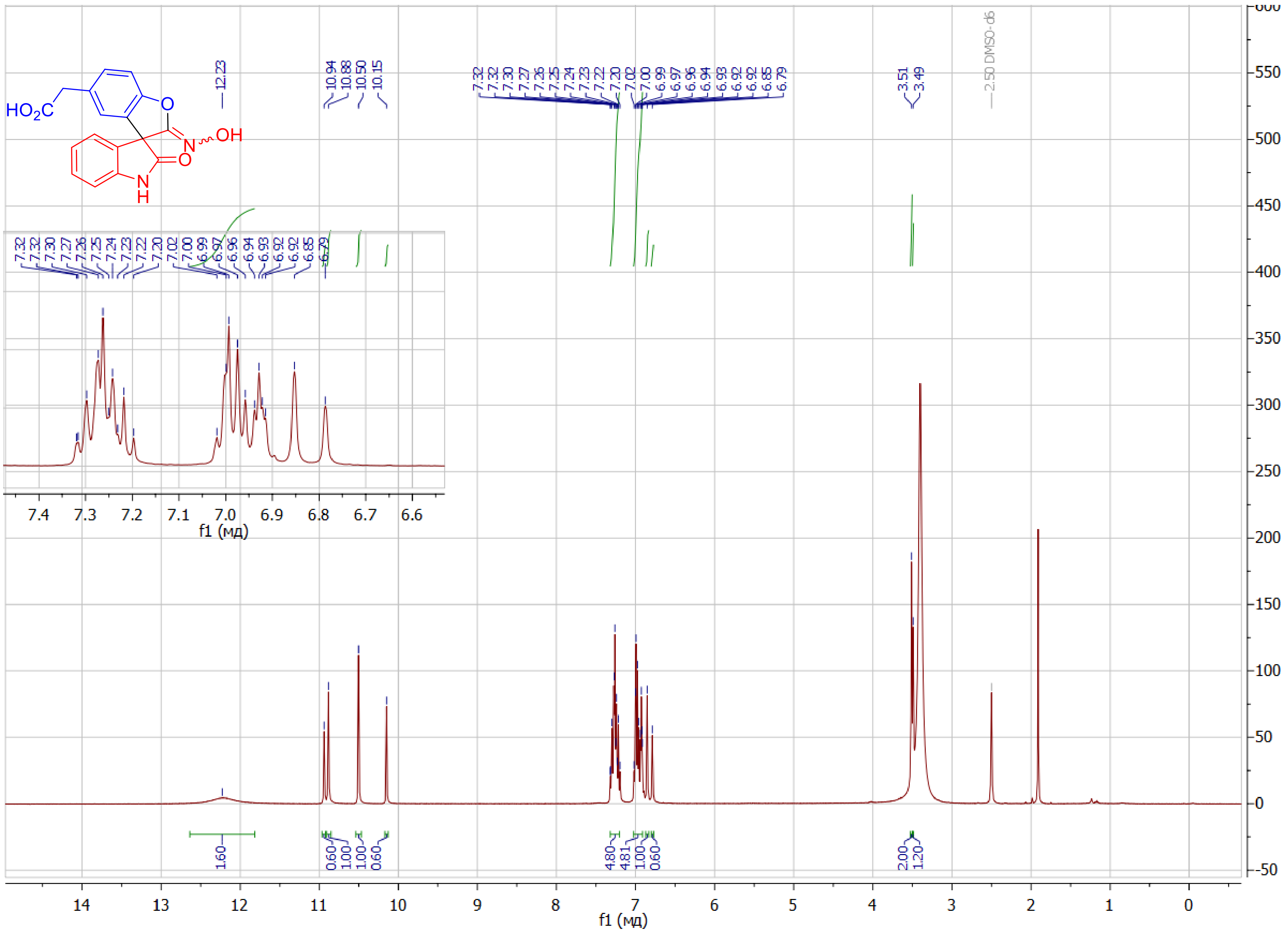

Figure S89. ^{1}H NMR spectrum of compound **13ah** in DMSO-d_6 (400 MHz)

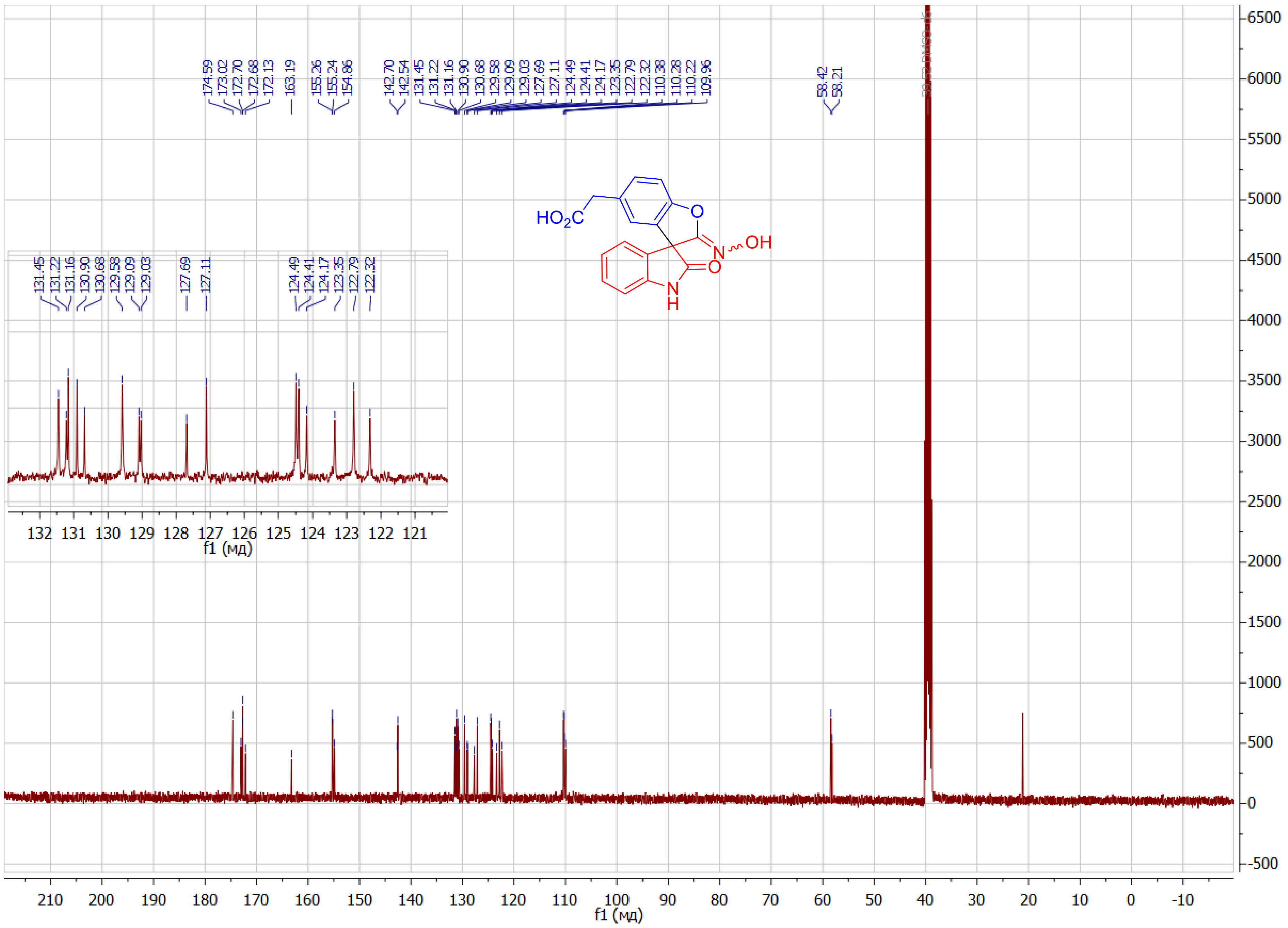

Figure S90. ^{13}C NMR spectrum of compound **13ah** in DMSO-d_6 (101 MHz)

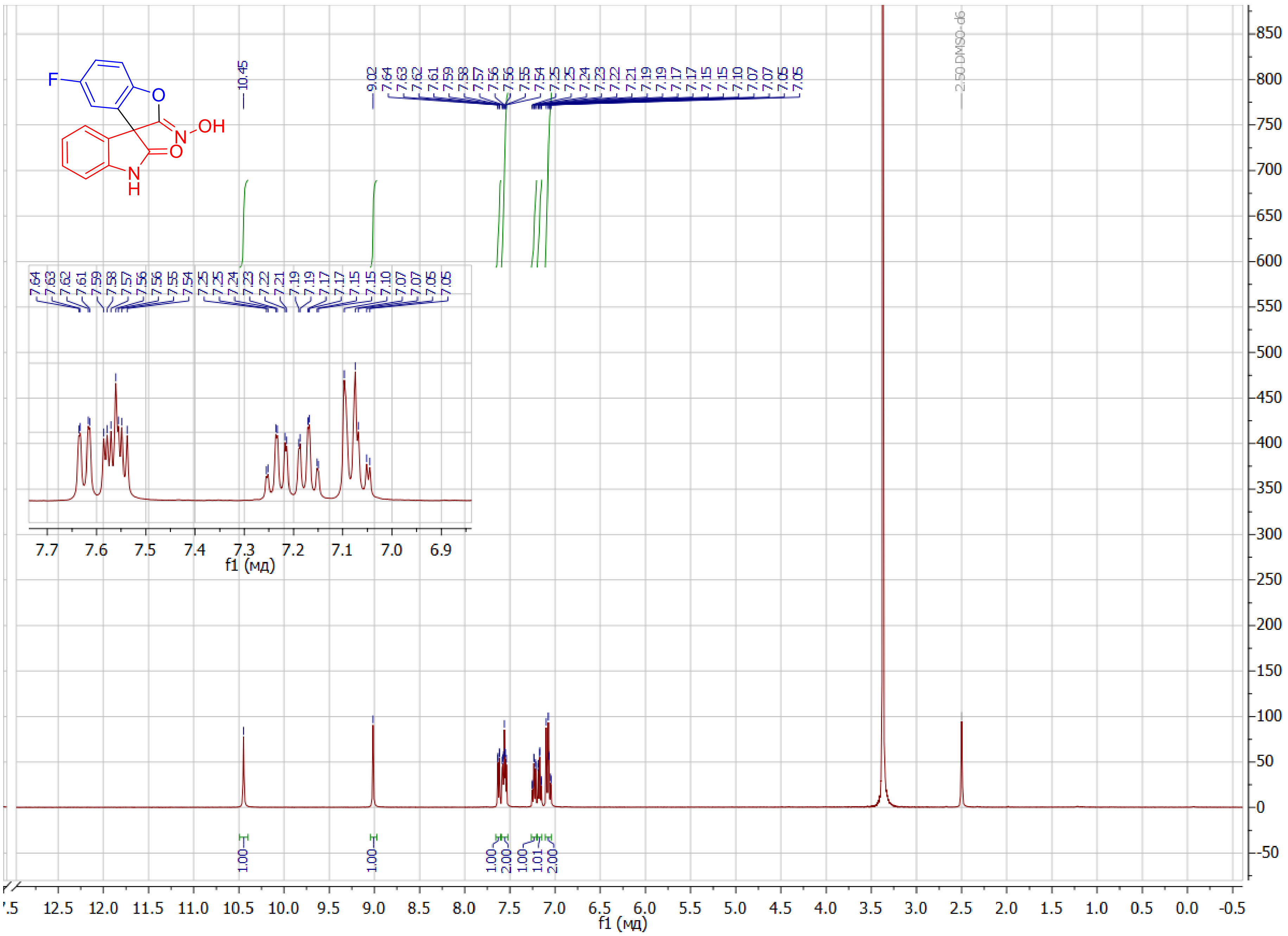

Figure S91. ^{1}H NMR spectrum of compound **13ai** in DMSO-d_6 (400 MHz)

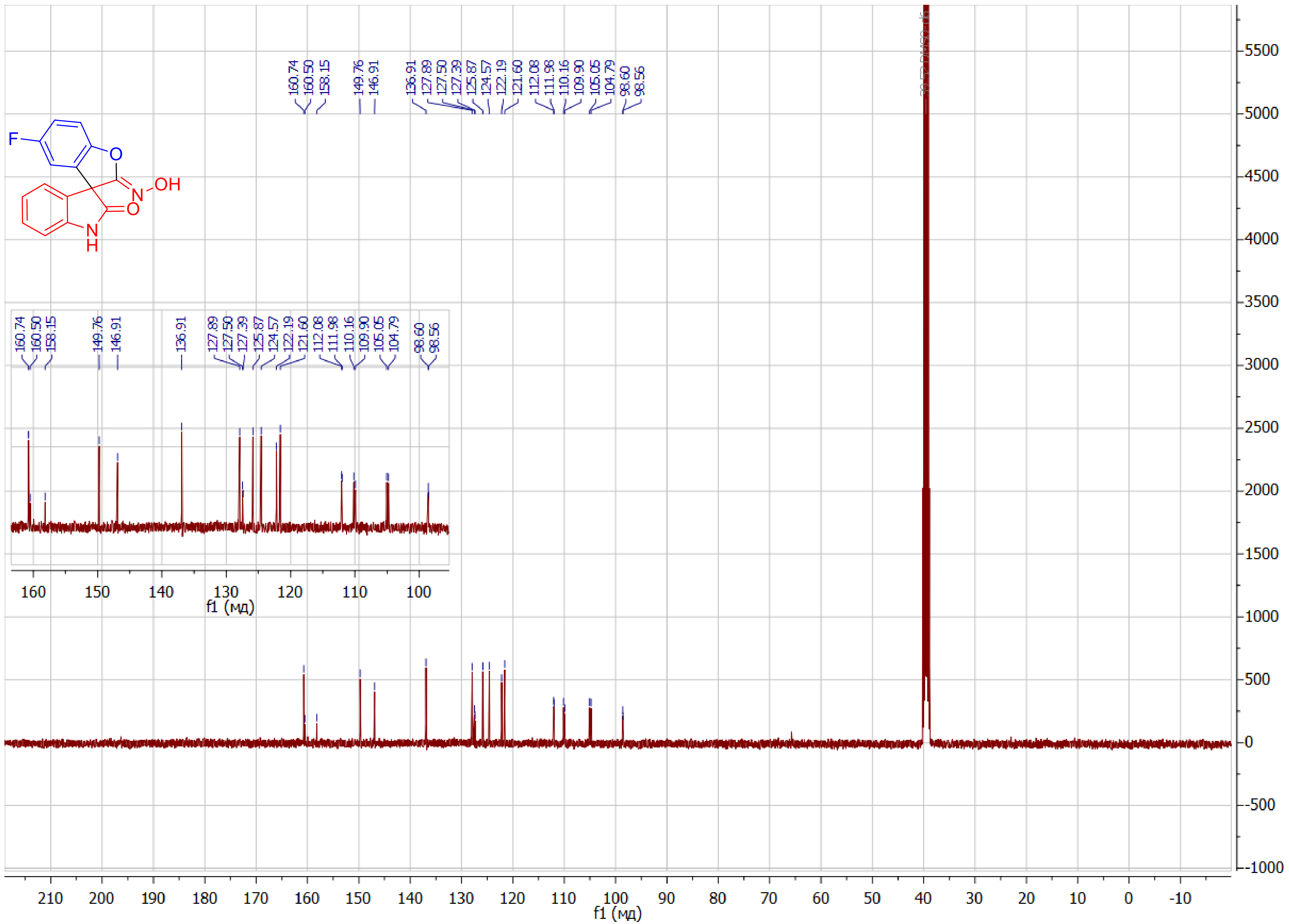

Figure S92. ^{13}C NMR spectrum of compound **13ai** in DMSO-d_6 (101 MHz)

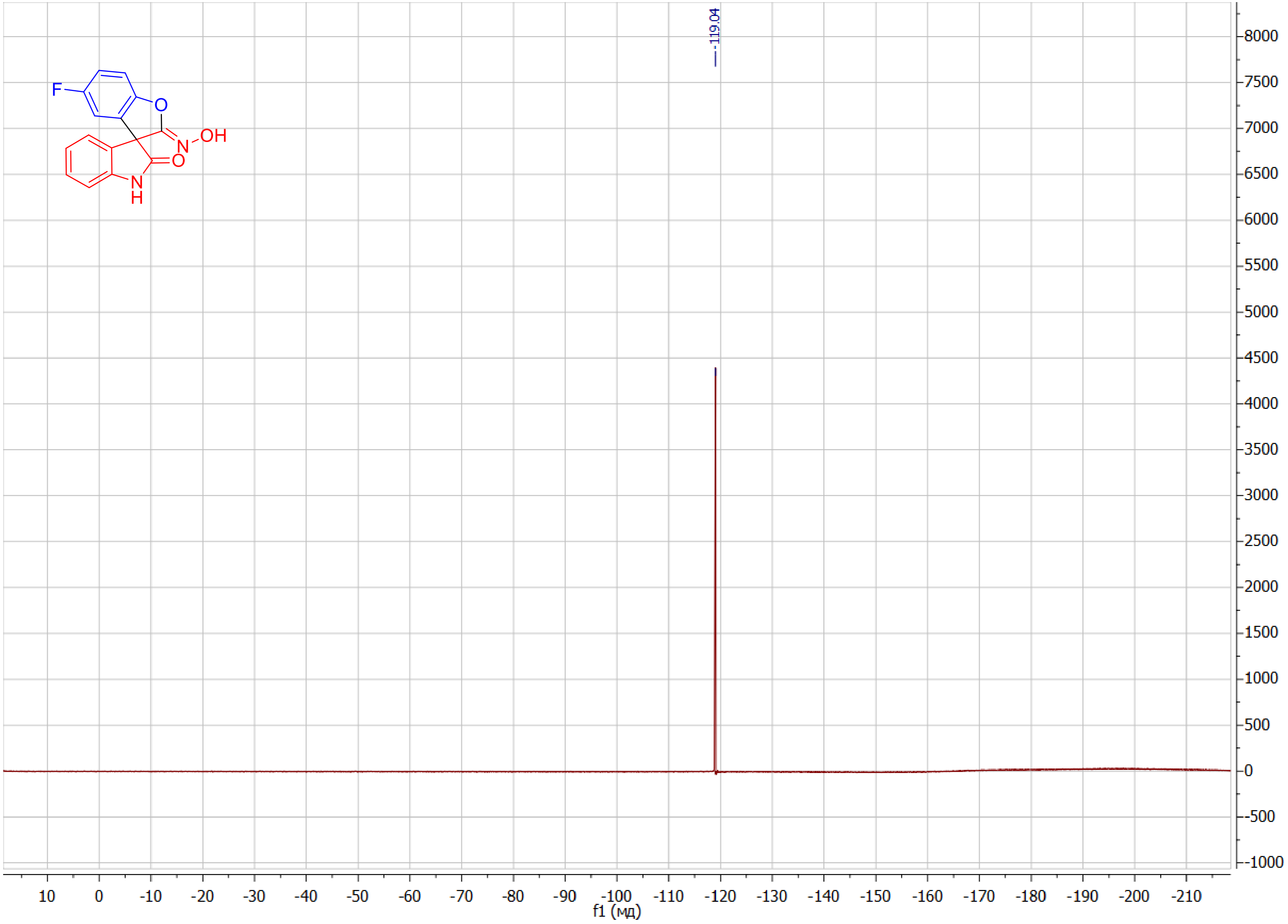

Figure S93. ^{19}F NMR spectrum of compound **13ai** in DMSO-d_6 (400 MHz)

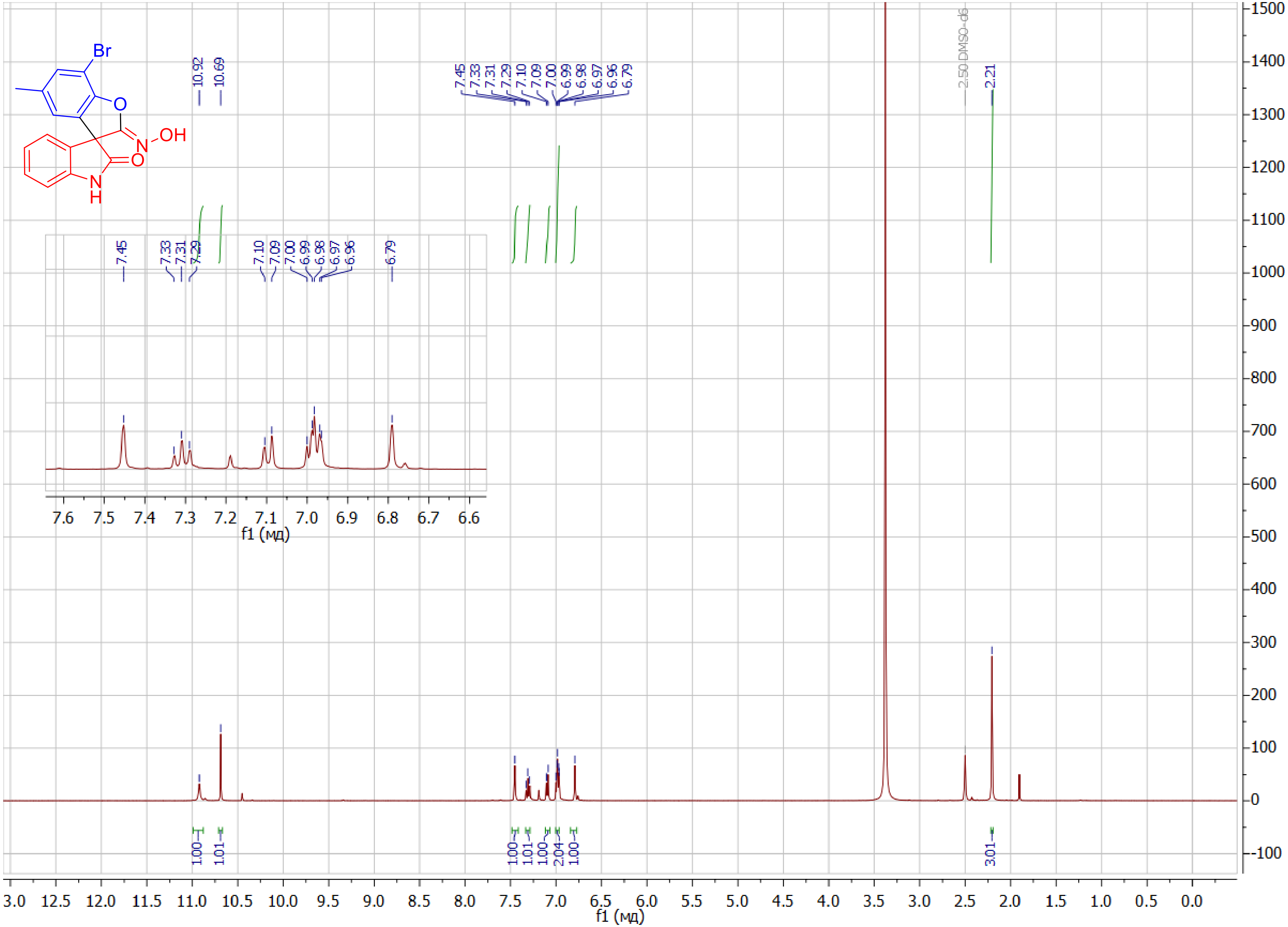

Figure S94. ^{1}H NMR spectrum of compound **13aj** in DMSO-d_6 (400 MHz)

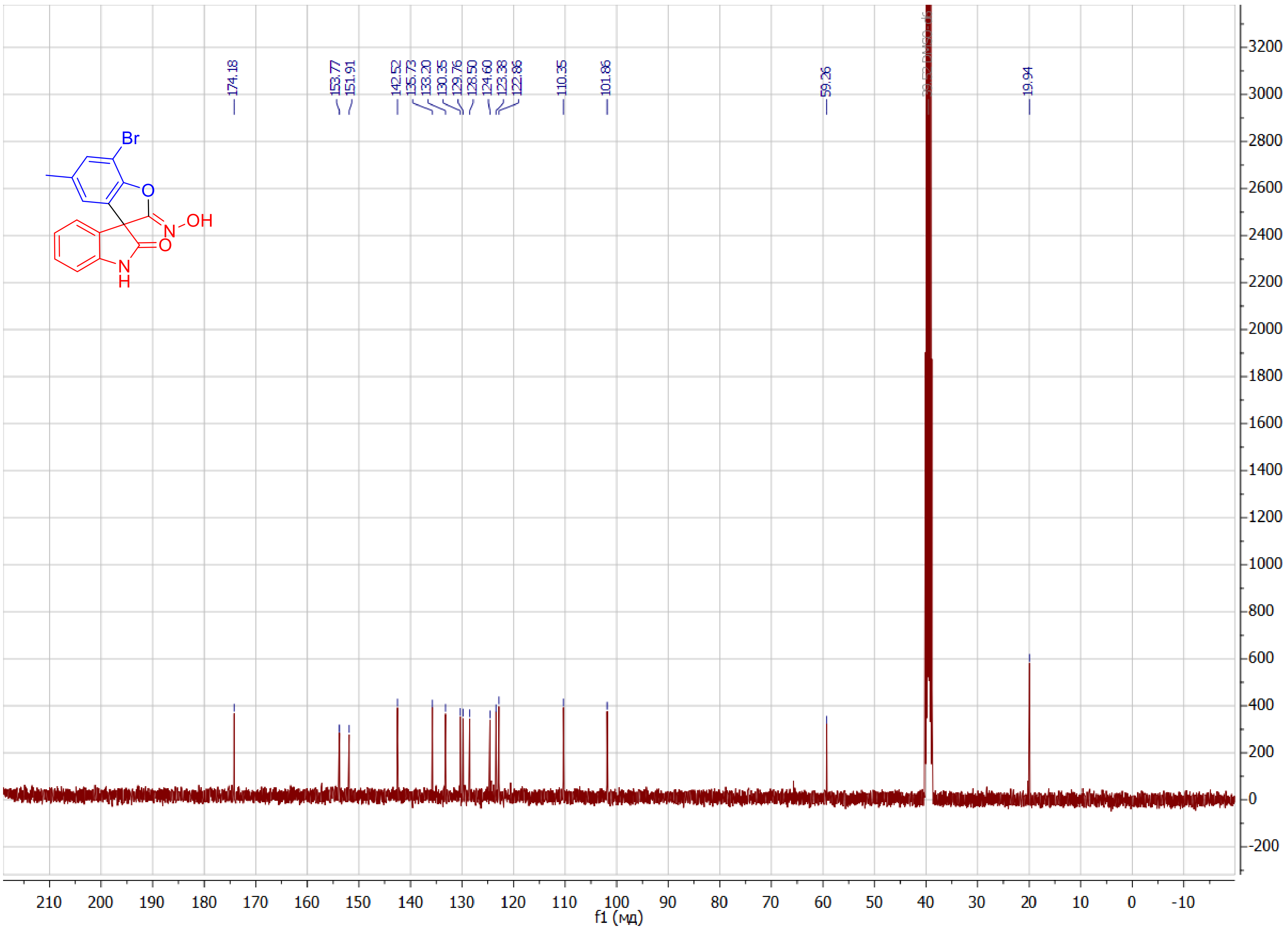

Figure S95. ^{13}C NMR spectrum of compound **13aj** in DMSO-d_6 (101 MHz)

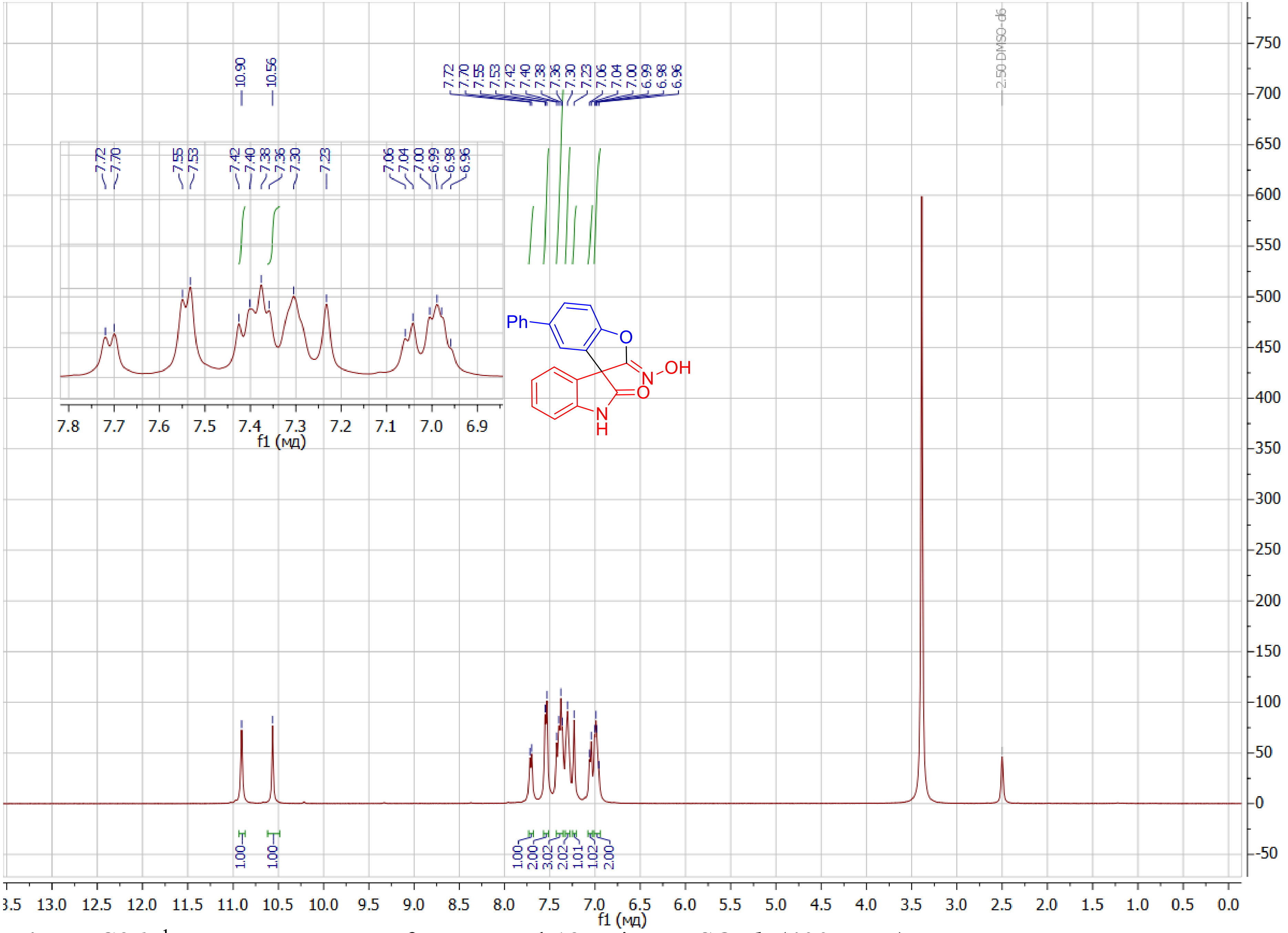

Figure S96. ^{1}H NMR spectrum of compound **13au** in DMSO-d_6 (400 MHz)

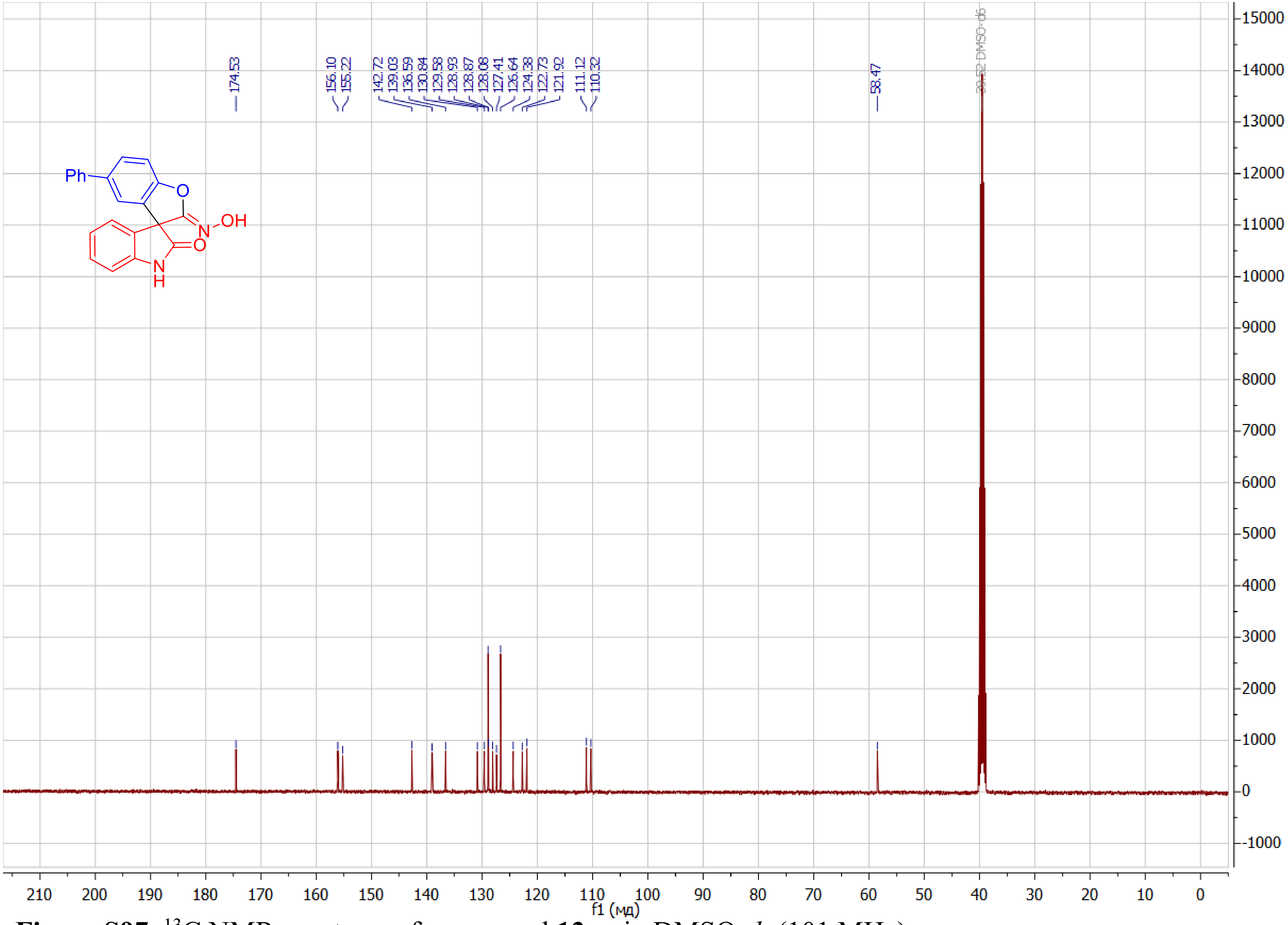

Figure S97. ^{13}C NMR spectrum of compound **13au** in DMSO-d_6 (101 MHz)

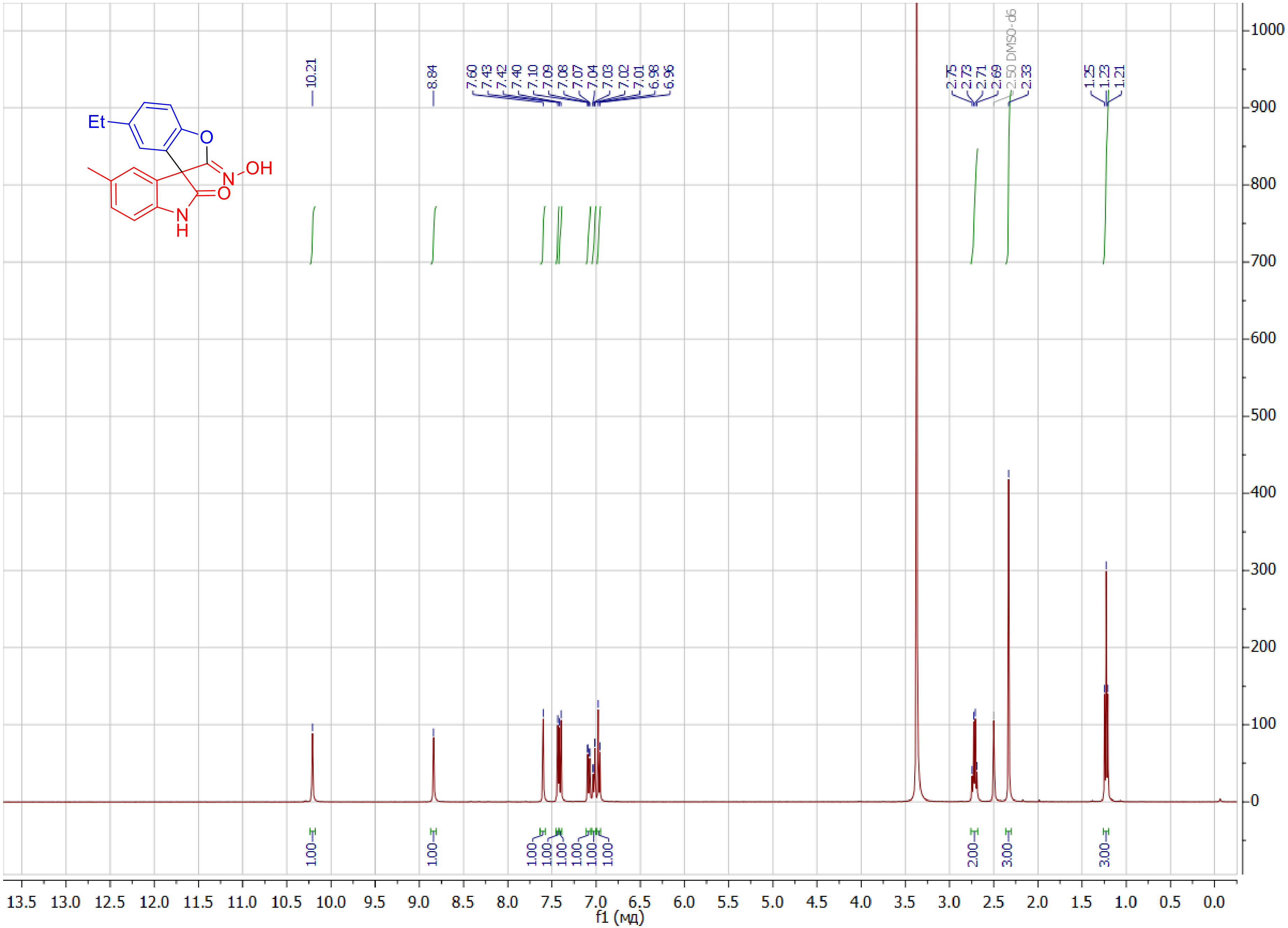

Figure S98. ^{1}H NMR spectrum of compound **13bb** in DMSO-d_6 (400 MHz)

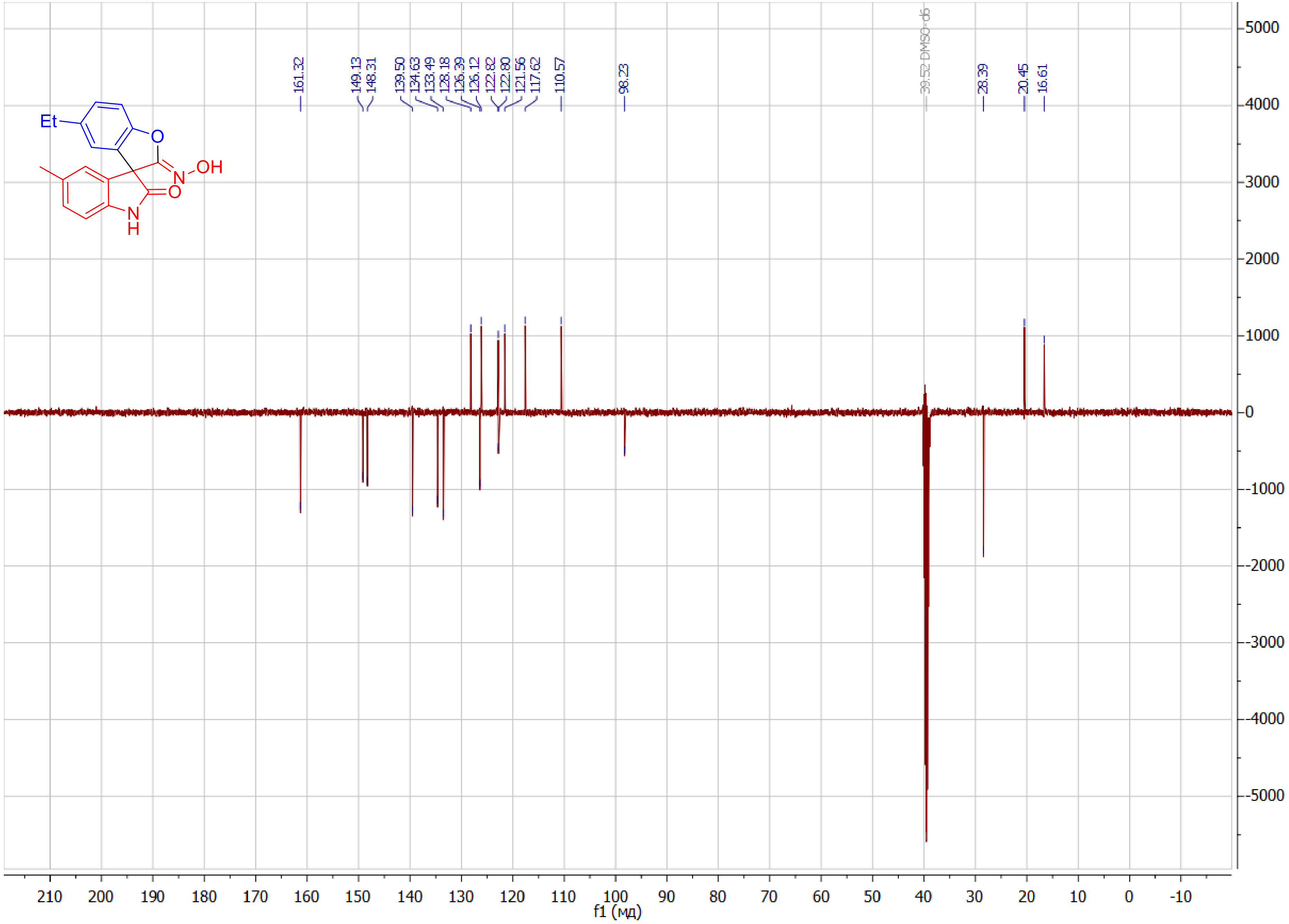

Figure S99. ^{13}C DEPTQ {^{1}H} NMR spectrum of compound **13bb** in DMSO-d_6 (101 MHz)

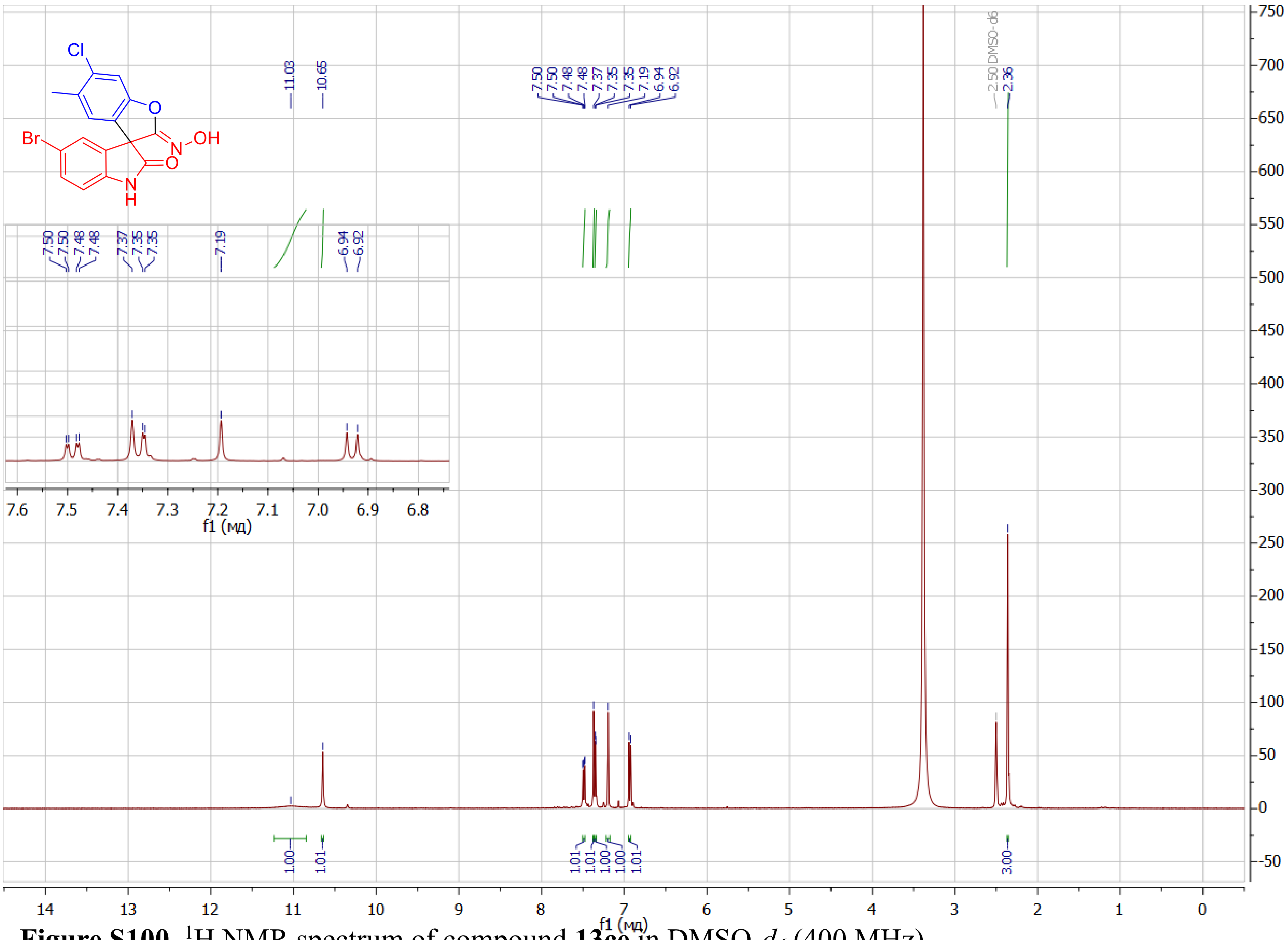

Figure S100. ^{1}H NMR spectrum of compound **13ce** in DMSO-d_6 (400 MHz)

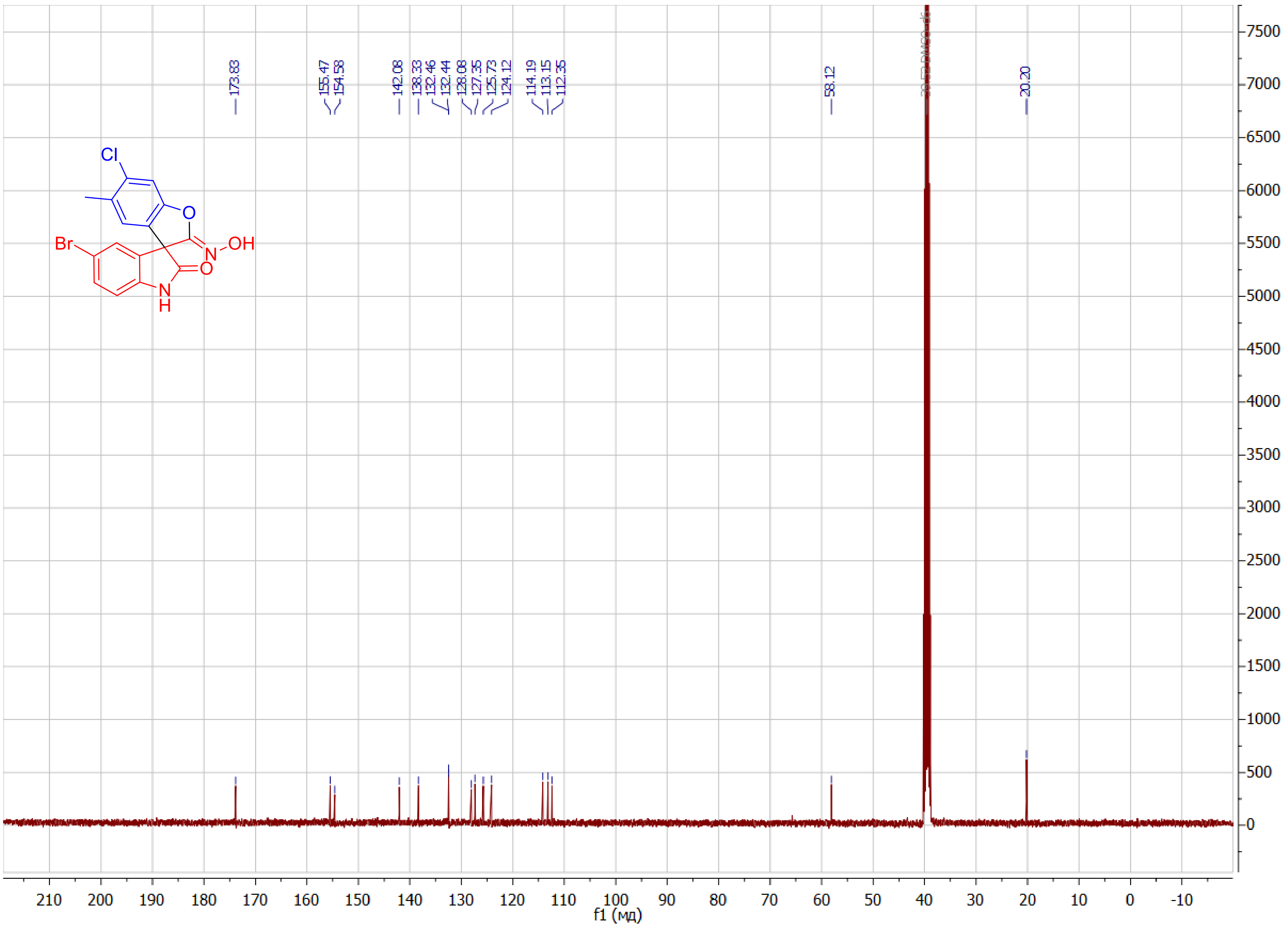

Figure S101. ^{13}C NMR spectrum of compound **13ce** in DMSO-d_6 (101 MHz)

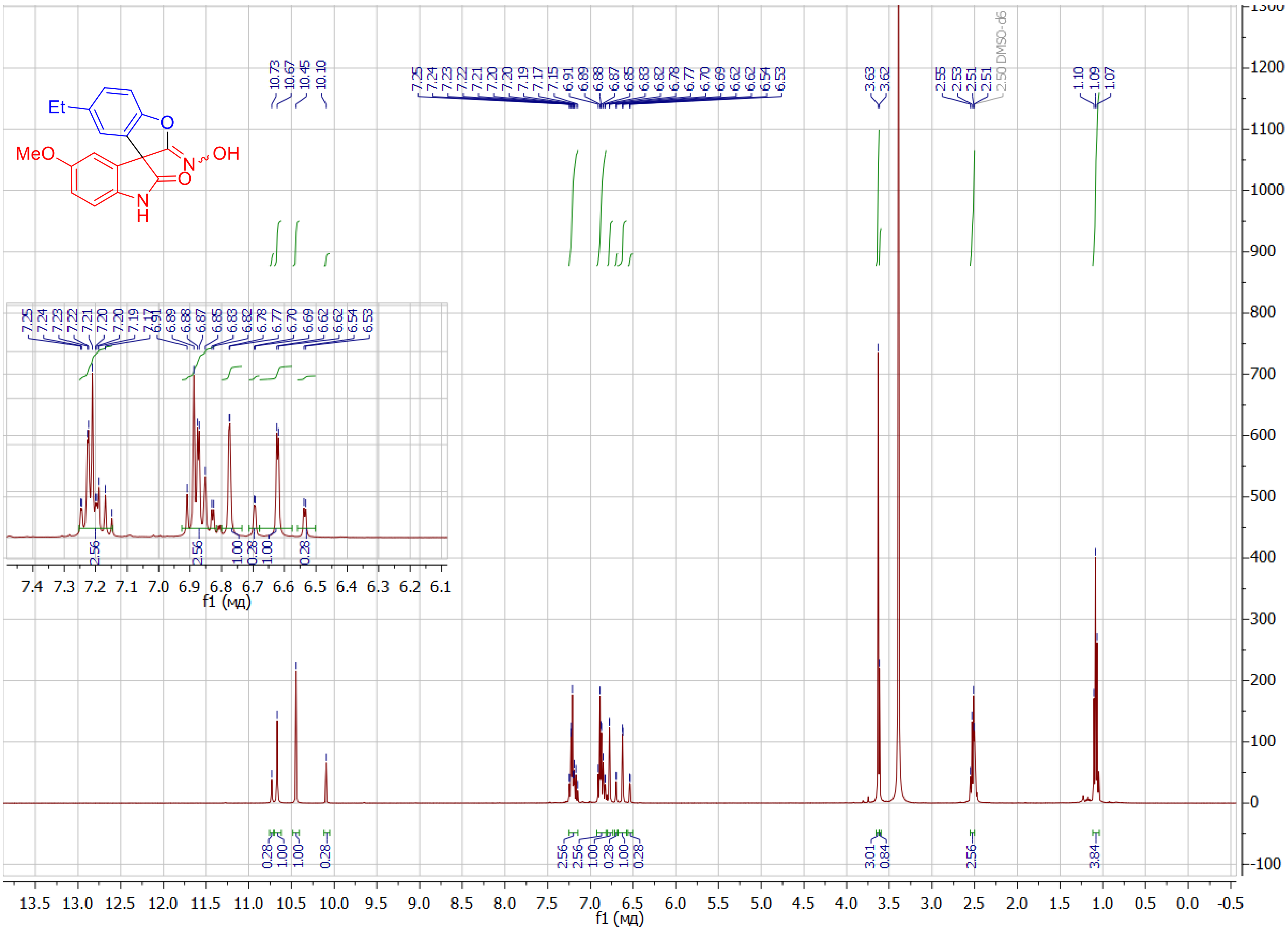

Figure S102. ^{1}H NMR spectrum of compound **13db** in DMSO-d_6 (400 MHz)

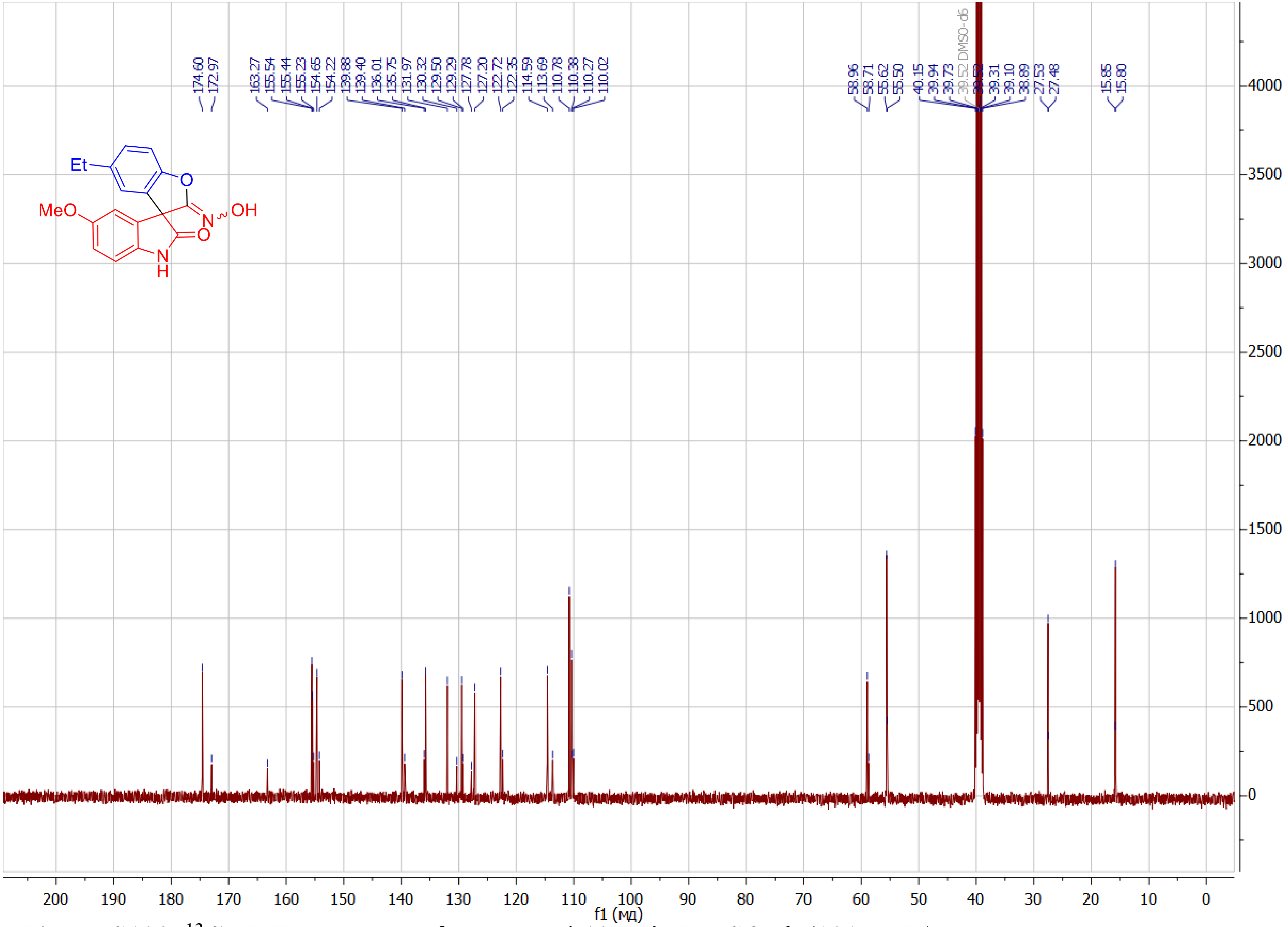

Figure S103. ^{13}C NMR spectrum of compound **13db** in DMSO-d_6 (101 MHz)

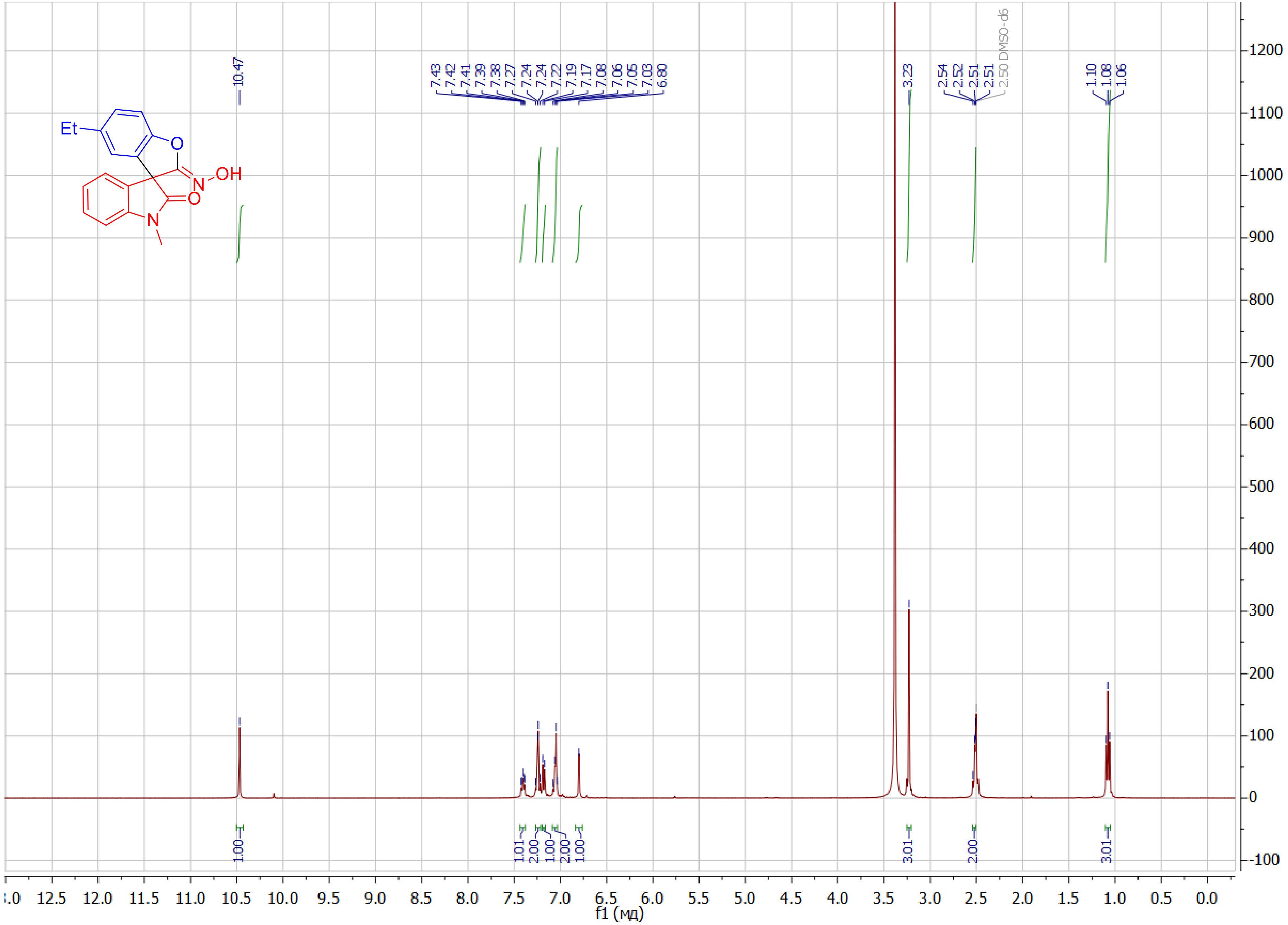

Figure S104. ^{1}H NMR spectrum of compound **13eb** in DMSO-d_6 (400 MHz)

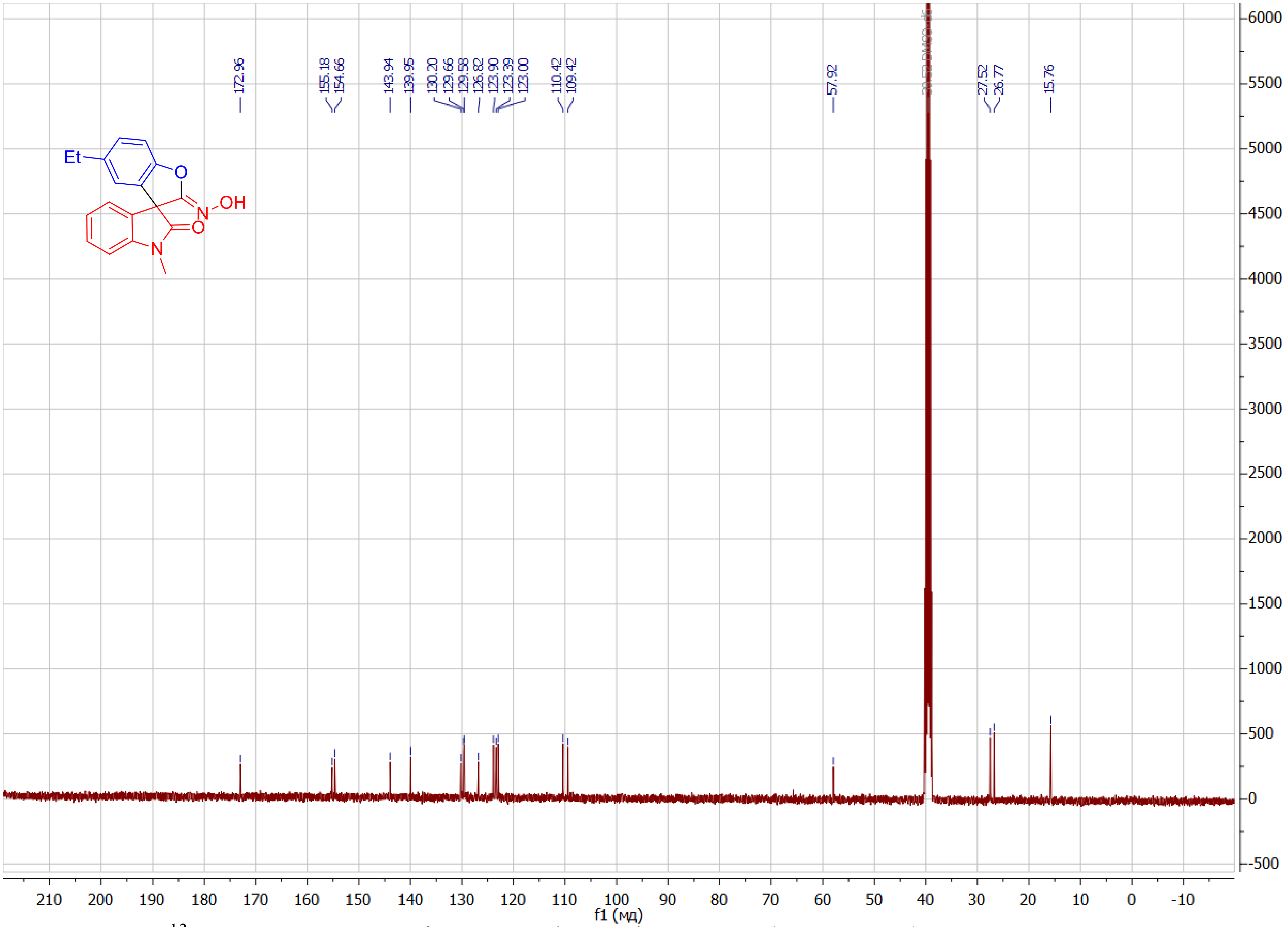

Figure S105. ^{13}C NMR spectrum of compound **13eb** in DMSO-d_6 (101 MHz)

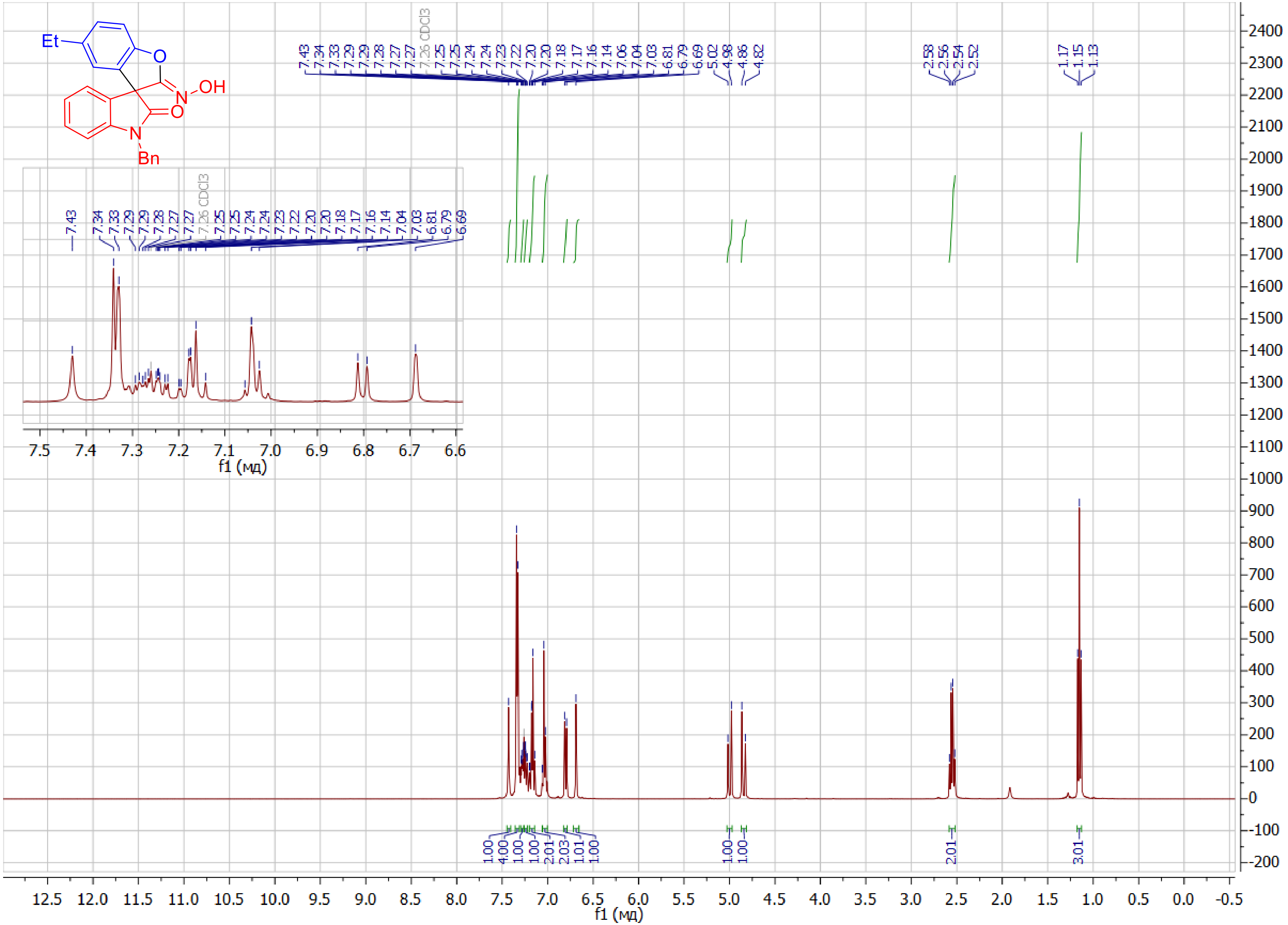

Figure S106. ^{1}H NMR spectrum of compound **13fb** in DMSO-d_6 (400 MHz)

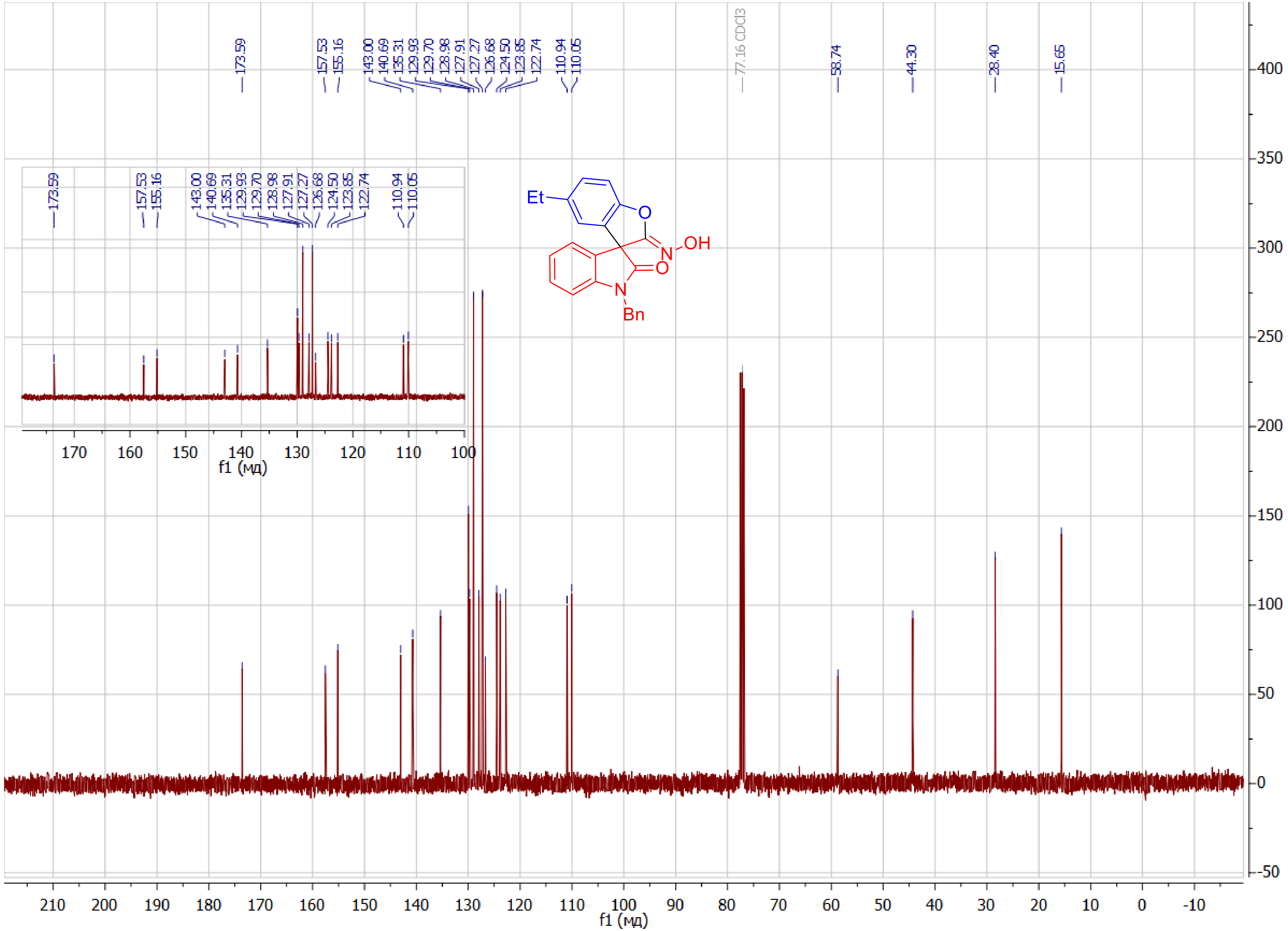

Figure S107. ^{13}C NMR spectrum of compound **13fb** in $CDCl_3$ (101 MHz)

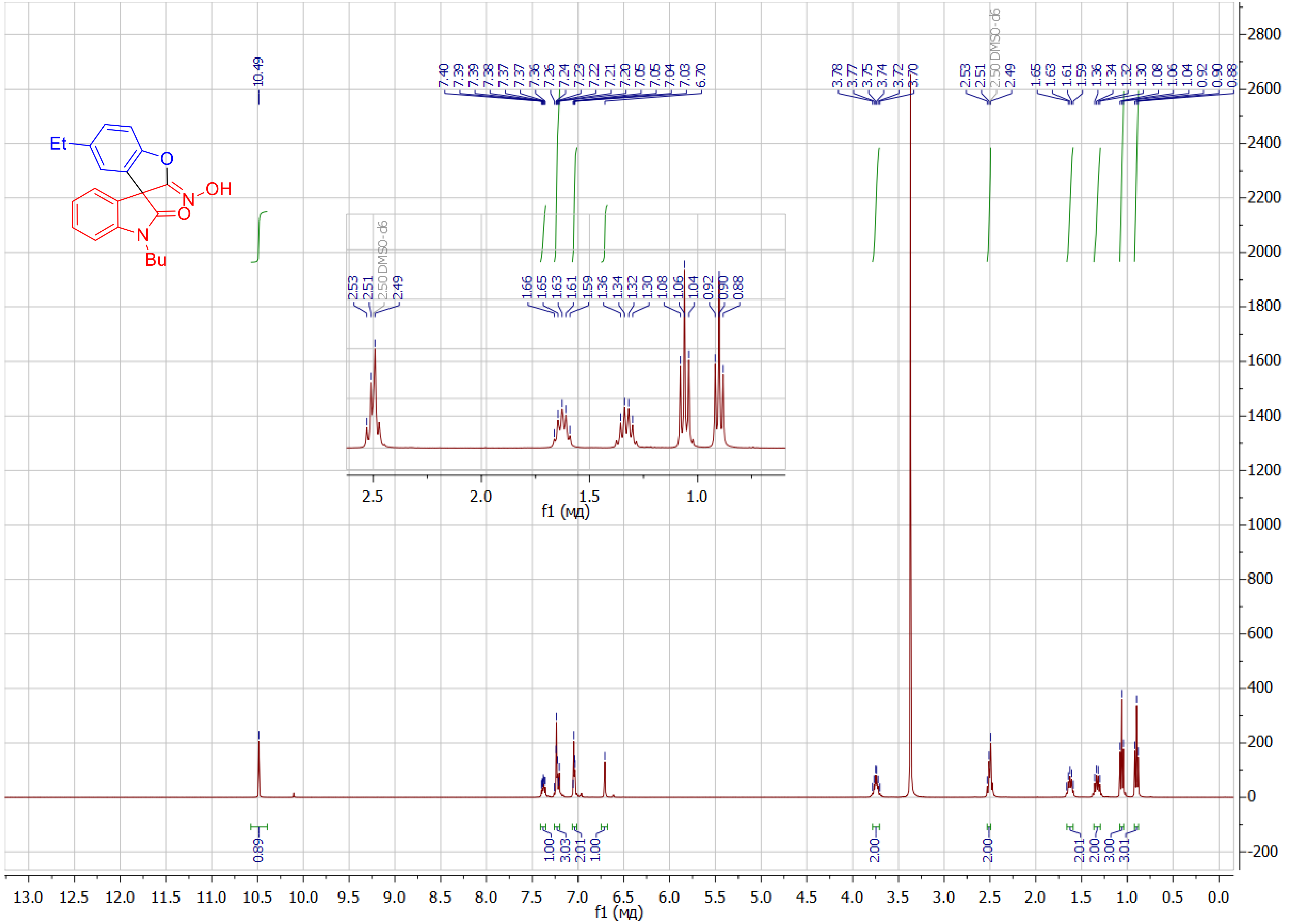

Figure S108. ^{1}H NMR spectrum of compound **13gb** in DMSO-d_6 (400 MHz)

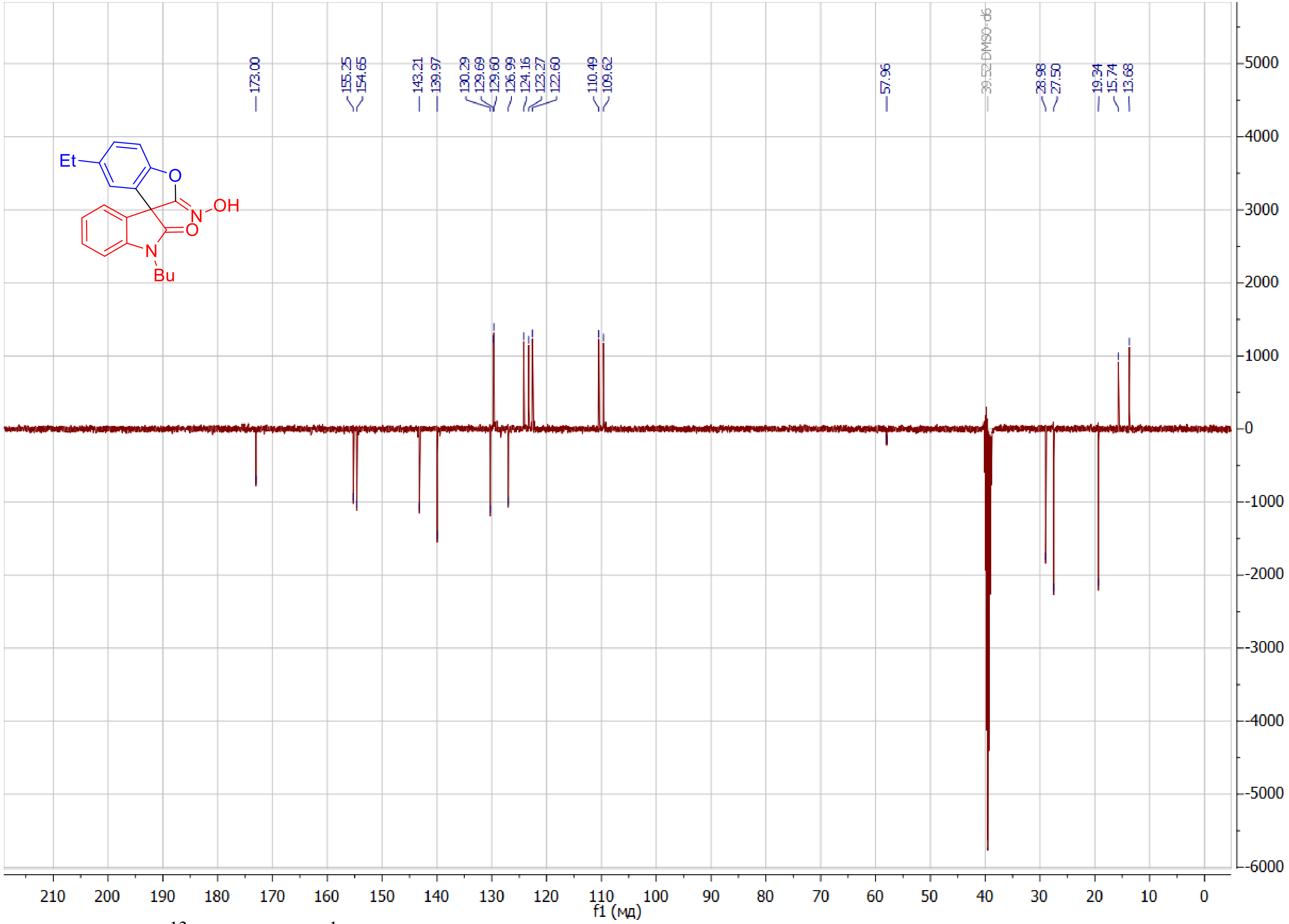

Figure S109. ^{13}C DEPTQ {^{1}H} NMR spectrum of compound **13gb** in DMSO-d_6 (101 MHz)

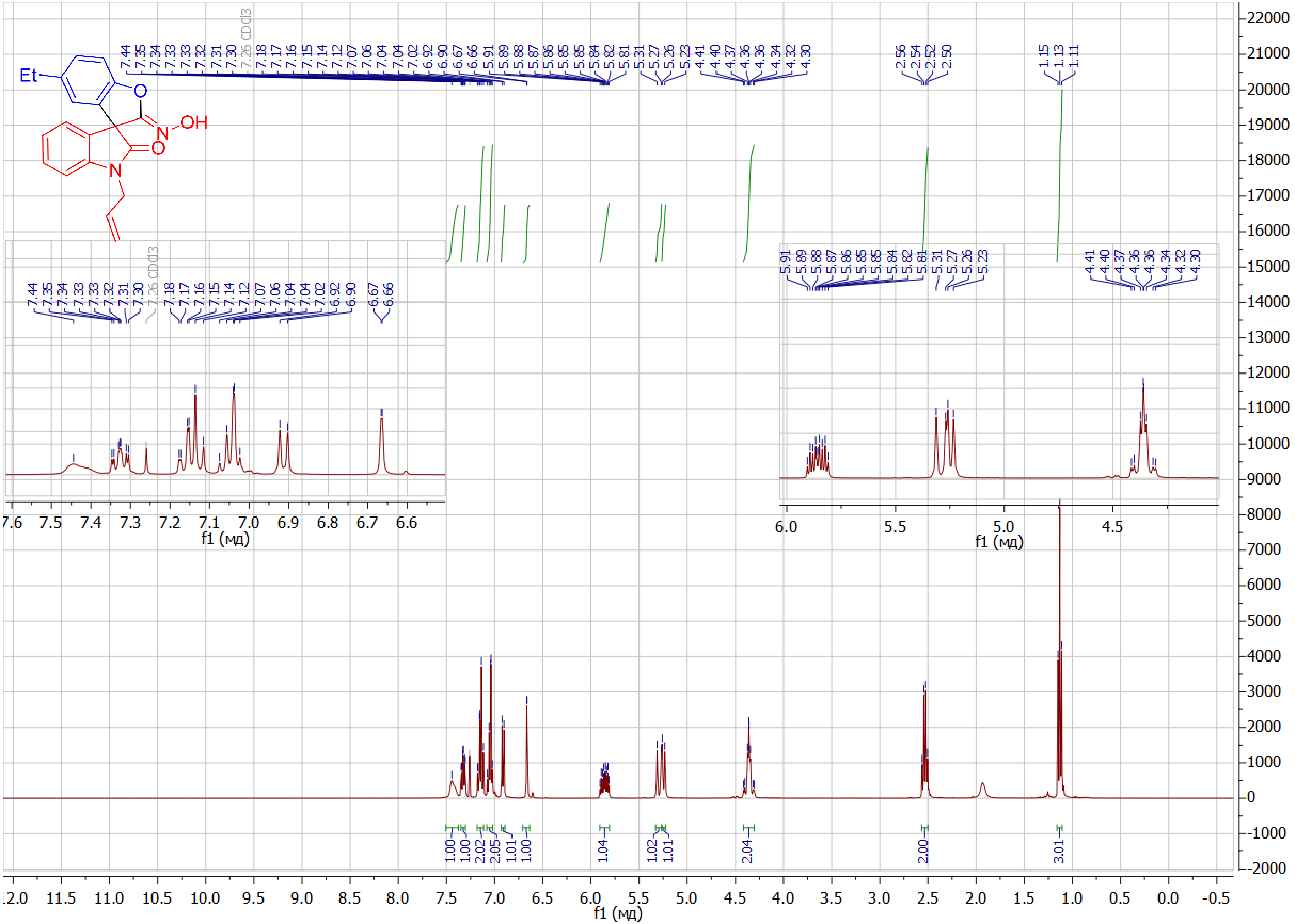

Figure S110. ^{1}H NMR spectrum of compound **13hb** in $CDCl_3$ (400 MHz)

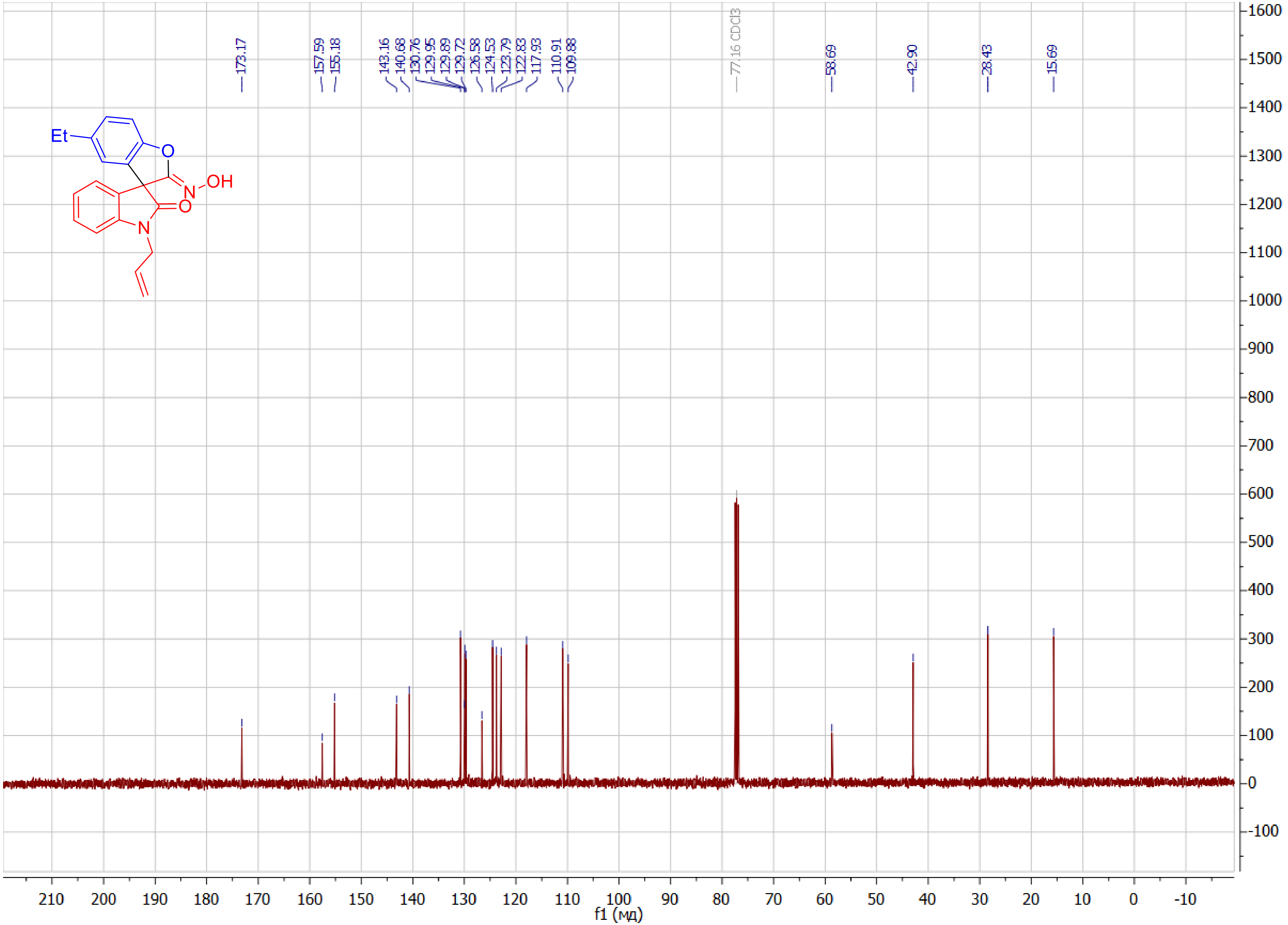

Figure S111. ^{13}C {^{1}H} NMR spectrum of compound **13hb** in $CDCl_3$ (101 MHz)

4. X-Ray Crystallography data for 2-Methylchromeno[3,4-*b*]indol-6(7*H*)-one **8aa**

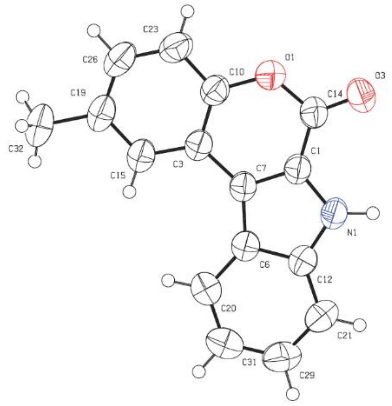

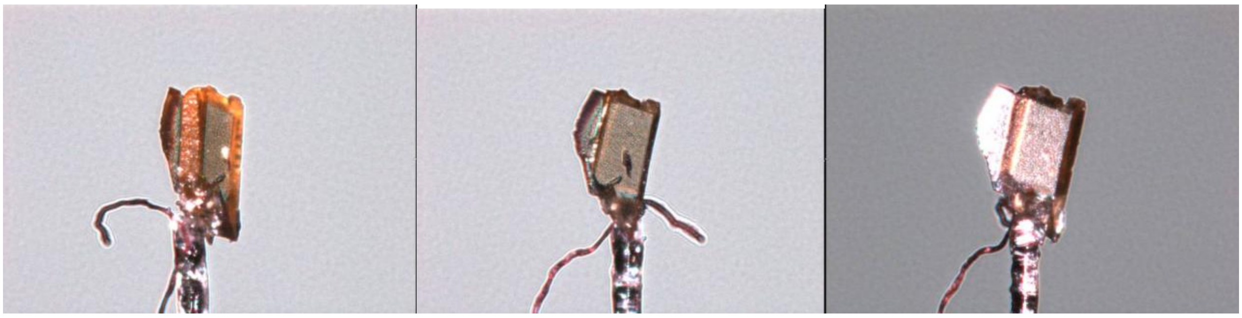

Table S1 Crystal data and structure refinement for 8aa.

Identification code	ANNA_ARK1065_2
Empirical formula	$C_{16}H_{11}NO_2$
Formula weight	249.26
Temperature/K	293(2)
Crystal system	triclinic
Space group	P-1
a/Å	8.2873(2)
b/Å	10.3532(2)
c/Å	15.0257(3)
α/°	72.683(2)
β/°	81.630(2)
γ/°	77.035(2)

Volume/Å^3	1195.09(5)
Z	4
ρ_{calc}g/cm^3	1.385
μ/mm^{-1}	0.745
F(000)	520.0
Crystal size/mm^3	0.295 × 0.188 × 0.092
Radiation	Cu Kα (λ = 1.54184)
2Θ range for data collection/°	6.184 to 152.418
Index ranges	-10 ≤ h ≤ 10, -13 ≤ k ≤ 13, -18 ≤ l ≤ 18
Reflections collected	33994
Independent reflections	4973 [R_{int} = 0.0486, R_{sigma} = 0.0226]
Data/restraints/parameters	4973/3/367
Goodness-of-fit on F^2	1.052
Final R indexes [I>=2σ (I)]	R_1 = 0.0405, wR_2 = 0.1157
Final R indexes [all data]	R_1 = 0.0463, wR_2 = 0.1224
Largest diff. peak/hole / e Å^{-3}	0.20/-0.14

Table S2 Fractional Atomic Coordinates (×10^4) and Equivalent Isotropic Displacement Parameters (Å^2×10^3) for 8aa. U_{eq} is defined as 1/3 of the trace of the orthogonalised U_{IJ} tensor.

**Atom**	***x***	***y***	***z***	**U(eq)**
O1	7762.1(12)	7331.7(9)	4632.6(6)	60.4(2)
O2	3972.3(13)	6320.8(9)	616.6(6)	60.4(2)
O3	6677.4(14)	7833.7(10)	3281.9(7)	71.0(3)
O4	5346.0(16)	5820.0(11)	1871.0(7)	78.0(3)
N1	6356.9(13)	4936.9(11)	3694.9(7)	54.0(2)
N2	4511.9(14)	8747.9(12)	1832.7(7)	58.3(3)

Table S2 Fractional Atomic Coordinates (×10^4) and Equivalent Isotropic Displacement Parameters (Å^2×10^3) for 8aa. U$_{eq}$ is defined as 1/3 of the trace of the orthogonalised U$_{IJ}$ tensor.

Atom	*x*	*y*	*z*	U(eq)
C1	6940.8(14)	5538.8(12)	4248.7(8)	50.1(3)
C2	3136.0(14)	9107.7(12)	553.6(7)	49.1(3)
C3	8102.2(14)	5036.6(13)	5733.7(8)	50.4(3)
C4	2618.6(15)	8656.1(12)	-164.3(8)	50.1(3)
C5	3011.9(15)	10406.0(12)	739.5(8)	51.3(3)
C6	7122.1(14)	3298.8(13)	5024.6(8)	50.8(3)
C7	7420.8(14)	4583.5(12)	5071.0(8)	48.6(2)
C8	4057.0(16)	8141.6(13)	1236.3(8)	53.2(3)
C9	3095.0(16)	7249.6(13)	-103.4(8)	53.1(3)
C10	8259.3(15)	6419.1(13)	5469.6(9)	53.8(3)
C11	1759.2(16)	9502.0(14)	-931.0(8)	54.7(3)
C12	6470.1(15)	3569.4(13)	4153.9(8)	52.1(3)
C13	3888.3(15)	10132.9(13)	1535.3(8)	54.0(3)
C14	7081.2(16)	6955.7(13)	3995.3(9)	55.4(3)
C15	8652.5(16)	4205.9(15)	6597.9(8)	57.4(3)
C16	2754.2(18)	6702.3(15)	-774.5(9)	61.6(3)
C17	4522.1(17)	6706.1(13)	1294.3(8)	58.4(3)
C18	1400.4(16)	8980.5(15)	-1614.9(8)	58.3(3)
C19	9335.8(16)	4727.8(16)	7176.8(9)	62.9(3)
C20	7360.9(18)	1939.2(14)	5605.7(10)	63.7(3)
C21	6058.9(18)	2536.2(16)	3855.4(10)	64.4(3)
C22	2257.5(18)	11775.7(14)	323.7(9)	61.2(3)
C23	8945.7(18)	6955.7(15)	6035.9(10)	64.7(3)
C24	1921.8(18)	7570.9(16)	-1520.8(9)	63.6(3)
C25	4042.9(18)	11175.7(16)	1916.4(10)	65.5(3)

Table S2 Fractional Atomic Coordinates (×10⁴) and Equivalent Isotropic Displacement Parameters (Å²×10³) for 8aa. U_{eq} is defined as 1/3 of the trace of the orthogonalised U_{IJ} tensor.

Atom	*x*	*y*	*z*	U(eq)
C26	9468.6(18)	6111.6(17)	6878.1(10)	67.8(4)
C27	3308(2)	12492.1(16)	1486.2(11)	72.4(4)
C28	2419(2)	12790.0(15)	703.2(11)	71.7(4)
C29	6334(2)	1222.1(16)	4436.7(12)	74.8(4)
C30	486(2)	9909.4(19)	-2437.9(10)	73.4(4)
C31	6963(2)	929.2(16)	5303.9(12)	75.6(4)
C32	9965(2)	3810(2)	8095.2(10)	82.6(5)

Table S3 Anisotropic Displacement Parameters (Å²×10³) for 8aa. The Anisotropic displacement factor exponent takes the form: $-2\pi^2[h^2a^{*2}U_{11}+2hka^*b^*U_{12}+...]$.

Atom	U_{11}	U_{22}	U_{33}	U_{23}	U_{13}	U_{12}
O1	67.2(5)	52.2(5)	64.2(5)	-17.8(4)	-17.0(4)	-6.8(4)
O2	78.0(6)	51.5(5)	52.3(4)	-15.2(4)	-14.5(4)	-6.8(4)
O3	81.7(7)	59.5(5)	66.8(6)	-0.1(4)	-27.0(5)	-13.3(5)
O4	108.6(9)	61.5(6)	58.1(5)	-14.1(4)	-32.3(5)	8.5(5)
N1	57.4(6)	60.2(6)	46.7(5)	-13.8(4)	-12.8(4)	-11.4(4)
N2	66.3(6)	62.1(6)	49.9(5)	-19.0(4)	-14.7(5)	-8.1(5)
C1	49.3(6)	54.3(6)	46.8(5)	-14.4(5)	-9.7(4)	-5.9(5)
C2	50.8(6)	52.6(6)	42.4(5)	-11.8(4)	-3.9(4)	-8.8(5)
C3	46.3(6)	58.3(6)	47.9(6)	-19.7(5)	-7.3(4)	-3.3(5)
C4	52.8(6)	54.3(6)	43.2(5)	-13.6(4)	-2.7(4)	-10.9(5)
C5	52.9(6)	54.7(6)	46.4(5)	-15.1(5)	-1.0(4)	-10.9(5)
C6	49.1(6)	55.1(6)	48.3(6)	-15.2(5)	-5.3(4)	-7.7(5)
C7	46.2(5)	53.6(6)	45.1(5)	-14.7(5)	-7.2(4)	-4.3(4)
C8	59.5(6)	55.7(6)	45.0(5)	-15.2(5)	-8.7(5)	-7.9(5)

Table S3 Anisotropic Displacement Parameters (Å^2×10^3) for 8aa. The Anisotropic displacement factor exponent takes the form: -2π^2[h^{2}a*^{2}U$_{11}$+2hka*b*U$_{12}$+...].

Atom	U_{11}	U_{22}	U_{33}	U_{23}	U_{13}	U_{12}
C9	59.5(7)	55.0(6)	45.8(5)	-14.3(5)	-5.7(5)	-11.4(5)
C10	50.3(6)	58.3(7)	55.0(6)	-22.7(5)	-9.8(5)	-1.8(5)
C11	56.7(6)	59.5(7)	47.6(6)	-12.7(5)	-7.2(5)	-11.2(5)
C12	50.6(6)	58.4(6)	48.9(6)	-16.7(5)	-3.8(4)	-11.3(5)
C13	54.6(6)	60.5(7)	49.6(6)	-19.3(5)	-2.1(5)	-12.5(5)
C14	54.6(6)	56.5(7)	53.8(6)	-12.7(5)	-13.1(5)	-5.8(5)
C15	56.8(7)	67.4(7)	48.7(6)	-18.1(5)	-9.2(5)	-7.5(6)
C16	71.5(8)	61.2(7)	58.6(7)	-21.8(6)	-7.3(6)	-17.7(6)
C17	70.2(8)	57.8(7)	45.5(6)	-13.9(5)	-11.9(5)	-4.9(6)
C18	54.7(6)	74.7(8)	47.6(6)	-14.1(5)	-6.9(5)	-18.3(6)
C19	56.3(7)	86.1(9)	49.5(6)	-27.3(6)	-10.0(5)	-4.8(6)
C20	69.0(8)	58.0(7)	59.6(7)	-8.9(6)	-10.6(6)	-9.4(6)
C21	68.3(8)	75.0(8)	60.5(7)	-27.7(6)	-3.6(6)	-24.0(6)
C22	66.0(7)	54.4(7)	59.5(7)	-13.3(5)	-3.9(5)	-8.3(6)
C23	64.1(8)	65.7(8)	73.6(8)	-35.0(7)	-11.7(6)	-6.1(6)
C24	68.0(8)	78.7(9)	54.8(6)	-25.3(6)	-8.7(5)	-23.9(7)
C25	67.2(8)	75.6(9)	64.5(7)	-33.0(7)	-0.6(6)	-20.4(7)
C26	62.5(7)	88.6(10)	65.3(8)	-41.7(7)	-12.6(6)	-8.7(7)
C27	76.7(9)	68.7(8)	82.7(9)	-38.7(7)	7.5(7)	-20.7(7)
C28	78.5(9)	54.1(7)	80.0(9)	-20.9(6)	3.6(7)	-10.5(6)
C29	85.4(10)	66.5(8)	82.7(10)	-29.1(7)	-0.1(8)	-28.0(7)
C30	74.6(9)	93.0(11)	53.2(7)	-12.1(7)	-18.4(6)	-20.0(8)
C31	86.4(10)	54.2(7)	83.8(10)	-12.9(7)	-5.9(8)	-17.4(7)
C32	85.3(10)	112.5(13)	52.6(7)	-22.2(8)	-20.5(7)	-15.6(9)

Table S4 Bond Lengths for 8aa.

Atom	Atom	Length/Å	Atom	Atom	Length/Å
O1	C10	1.3831(15)	C6	C7	1.4294(17)
O1	C14	1.3655(15)	C6	C12	1.4148(16)
O2	C9	1.3856(15)	C6	C20	1.4058(18)
O2	C17	1.3645(15)	C8	C17	1.4285(18)
O3	C14	1.2145(16)	C9	C16	1.3830(17)
O4	C17	1.2152(16)	C10	C23	1.3858(17)
N1	C1	1.3698(15)	C11	C18	1.3882(17)
N1	C12	1.3664(16)	C12	C21	1.3984(18)
N2	C8	1.3706(15)	C13	C25	1.4006(18)
N2	C13	1.3697(17)	C15	C19	1.3893(18)
C1	C7	1.3793(16)	C16	C24	1.374(2)
C1	C14	1.4288(18)	C18	C24	1.396(2)
C2	C4	1.4461(15)	C18	C30	1.5055(19)
C2	C5	1.4312(16)	C19	C26	1.393(2)
C2	C8	1.3820(16)	C19	C32	1.5118(19)
C3	C7	1.4461(15)	C20	C31	1.374(2)
C3	C10	1.3976(18)	C21	C29	1.370(2)
C3	C15	1.4020(17)	C22	C28	1.374(2)
C4	C9	1.3993(17)	C23	C26	1.373(2)
C4	C11	1.3999(17)	C25	C27	1.365(2)
C5	C13	1.4130(17)	C27	C28	1.399(2)
C5	C22	1.4073(18)	C29	C31	1.398(2)

Table S5 Bond Angles for 8aa.

Atom	Atom	Atom	Angle/°	Atom	Atom	Atom	Angle/°
C14	O1	C10	122.78(10)	C23	C10	C3	121.45(12)

Table S5 Bond Angles for 8aa.

Atom	Atom	Atom	Angle/°	Atom	Atom	Atom	Angle/°
C17	O2	C9	123.10(10)	C18	C11	C4	121.80(12)
C12	N1	C1	107.61(10)	N1	C12	C6	109.19(10)
C13	N2	C8	107.37(10)	N1	C12	C21	128.44(11)
N1	C1	C7	110.84(11)	C21	C12	C6	122.36(12)
N1	C1	C14	124.52(11)	N2	C13	C5	109.25(10)
C7	C1	C14	124.62(11)	N2	C13	C25	128.39(12)
C5	C2	C4	135.49(11)	C25	C13	C5	122.36(13)
C8	C2	C4	118.56(11)	O1	C14	C1	115.07(10)
C8	C2	C5	105.85(10)	O3	C14	O1	117.87(12)
C10	C3	C7	116.44(11)	O3	C14	C1	127.05(12)
C10	C3	C15	117.76(11)	C19	C15	C3	121.58(13)
C15	C3	C7	125.78(12)	C24	C16	C9	118.72(13)
C9	C4	C2	116.37(11)	O2	C17	C8	114.99(11)
C9	C4	C11	117.49(11)	O4	C17	O2	117.99(12)
C11	C4	C2	126.07(11)	O4	C17	C8	127.02(12)
C13	C5	C2	106.41(10)	C11	C18	C24	118.08(12)
C22	C5	C2	135.09(12)	C11	C18	C30	120.99(14)
C22	C5	C13	118.50(11)	C24	C18	C30	120.93(12)
C12	C6	C7	106.16(10)	C15	C19	C26	118.29(13)
C20	C6	C7	135.42(11)	C15	C19	C32	120.94(15)
C20	C6	C12	118.40(12)	C26	C19	C32	120.74(13)
C1	C7	C3	118.32(11)	C31	C20	C6	118.83(13)
C1	C7	C6	106.20(10)	C29	C21	C12	117.36(13)
C6	C7	C3	135.47(11)	C28	C22	C5	118.61(13)
N2	C8	C2	111.13(11)	C26	C23	C10	119.15(14)
N2	C8	C17	124.40(11)	C16	C24	C18	121.97(12)

Table S5 Bond Angles for 8aa.

Atom	Atom	Atom	Angle/˚	Atom	Atom	Atom	Angle/˚
C2	C8	C17	124.44(11)	C27	C25	C13	117.28(14)
O2	C9	C4	122.45(10)	C23	C26	C19	121.76(12)
C16	C9	O2	115.60(11)	C25	C27	C28	121.55(13)
C16	C9	C4	121.93(12)	C22	C28	C27	121.69(14)
O1	C10	C3	122.75(10)	C21	C29	C31	121.40(14)
O1	C10	C23	115.80(12)	C20	C31	C29	121.63(14)

Table S6 Torsion Angles for 8aa.

A	B	C	D	Angle/˚	A	B	C	D	Angle/˚
O1	C10	C23	C26	180.00(11)	C8	C2	C4	C9	0.36(17)
O2	C9	C16	C24	-178.49(12)	C8	C2	C4	C11	-176.50(11)
N1	C1	C7	C3	178.79(10)	C8	C2	C5	C13	0.21(13)
N1	C1	C7	C6	0.25(14)	C8	C2	C5	C22	-179.77(14)
N1	C1	C14	O1	-177.26(11)	C9	O2	C17	O4	-176.71(12)
N1	C1	C14	O3	2.1(2)	C9	O2	C17	C8	2.92(19)
N1	C12	C21	C29	178.07(13)	C9	C4	C11	C18	-0.65(19)
N2	C8	C17	O2	-178.39(12)	C9	C16	C24	C18	-0.6(2)
N2	C8	C17	O4	1.2(2)	C10	O1	C14	O3	179.70(11)
N2	C13	C25	C27	179.69(13)	C10	O1	C14	C1	-0.92(17)
C1	N1	C12	C6	0.72(13)	C10	C3	C7	C1	-1.63(16)
C1	N1	C12	C21	-178.40(13)	C10	C3	C7	C6	176.37(12)
C2	C4	C9	O2	1.85(17)	C10	C3	C15	C19	-0.13(19)
C2	C4	C9	C16	-176.48(11)	C10	C23	C26	C19	0.3(2)
C2	C4	C11	C18	176.17(11)	C11	C4	C9	O2	178.99(11)
C2	C5	C13	N2	-0.40(13)	C11	C4	C9	C16	0.66(19)
C2	C5	C13	C25	179.42(12)	C11	C18	C24	C16	0.6(2)

Table S6 Torsion Angles for 8aa.

A	B	C	D	Angle/˚	A	B	C	D	Angle/˚
C2	C5	C22	C28	-179.37(14)	C12	N1	C1	C7	-0.61(14)
C2	C8	C17	O2	-0.62(19)	C12	N1	C1	C14	177.59(11)
C2	C8	C17	O4	178.96(14)	C12	C6	C7	C1	0.19(13)
C3	C10	C23	C26	-0.6(2)	C12	C6	C7	C3	-177.98(13)
C3	C15	C19	C26	-0.1(2)	C12	C6	C20	C31	0.4(2)
C3	C15	C19	C32	-178.38(12)	C12	C21	C29	C31	1.5(2)
C4	C2	C5	C13	-176.05(13)	C13	N2	C8	C2	-0.30(15)
C4	C2	C5	C22	4.0(2)	C13	N2	C8	C17	177.73(12)
C4	C2	C8	N2	177.06(10)	C13	C5	C22	C28	0.65(19)
C4	C2	C8	C17	-0.97(19)	C13	C25	C27	C28	0.7(2)
C4	C9	C16	C24	0.0(2)	C14	O1	C10	C3	-0.17(18)
C4	C11	C18	C24	0.05(19)	C14	O1	C10	C23	179.25(11)
C4	C11	C18	C30	-179.54(12)	C14	C1	C7	C3	0.60(18)
C5	C2	C4	C9	176.26(13)	C14	C1	C7	C6	-177.94(11)
C5	C2	C4	C11	-0.6(2)	C15	C3	C7	C1	-179.86(11)
C5	C2	C8	N2	0.05(14)	C15	C3	C7	C6	-1.9(2)
C5	C2	C8	C17	-177.98(12)	C15	C3	C10	O1	179.85(11)
C5	C13	C25	C27	-0.1(2)	C15	C3	C10	C23	0.46(18)
C5	C22	C28	C27	-0.1(2)	C15	C19	C26	C23	0.0(2)
C6	C12	C21	C29	-0.9(2)	C17	O2	C9	C4	-3.70(19)
C6	C20	C31	C29	0.1(2)	C17	O2	C9	C16	174.73(12)
C7	C1	C14	O1	0.69(18)	C20	C6	C7	C1	178.45(14)
C7	C1	C14	O3	-179.99(13)	C20	C6	C7	C3	0.3(2)
C7	C3	C10	O1	1.48(17)	C20	C6	C12	N1	-179.18(11)
C7	C3	C10	C23	-177.91(11)	C20	C6	C12	C21	0.00(18)
C7	C3	C15	C19	178.08(11)	C21	C29	C31	C20	-1.1(3)

Table S6 Torsion Angles for 8aa.

A	B	C	D	Angle/˚	A	B	C	D	Angle/˚
C7	C6	C12	N1	-0.56(13)	C22	C5	C13	N2	179.58(11)
C7	C6	C12	C21	178.62(11)	C22	C5	C13	C25	-0.60(18)
C7	C6	C20	C31	-177.69(14)	C25	C27	C28	C22	-0.7(2)
C8	N2	C13	C5	0.43(14)	C30	C18	C24	C16	-179.81(14)
C8	N2	C13	C25	-179.37(13)	C32	C19	C26	C23	178.28(14)

Table S7 Hydrogen Atom Coordinates (Å×10^4) and Isotropic Displacement Parameters (Å^2×10^3) for 8aa.

Atom	*x*	*y*	*z*	U(eq)
H11	1419.29	10438.99	-983.6	66
H15	8557.79	3283.2	6788.23	69
H16	3081.96	5764.45	-721.77	74
H20	7780.6	1727.24	6184.2	76
H21	5616.25	2733.17	3284.86	77
H22	1661.86	11989.79	-196.92	73
H23	9050.7	7875.97	5847.75	78
H24	1699.15	7208.8	-1976.87	76
H25	4622.57	10979.45	2441.19	79
H26	9923.8	6473.45	7259.34	81
H27	3400.02	13207.5	1719.34	87
H28	1925.17	13697.93	432.4	86
H29	6096.53	509.86	4250.29	90
H30A	-271.64	10650.61	-2251.75	110
H30B	-123.14	9390.86	-2654.16	110
H30C	1268.73	10279.05	-2932.5	110
H31	7116.95	28.39	5686.11	91

Table S7 Hydrogen Atom Coordinates (Å×10^4) and Isotropic Displacement Parameters (Å^2×10^3) for 8aa.

Atom	*x*	*y*	*z*	U(eq)
H32A	9541.71	2970.83	8262.58	124
H32B	9594.83	4276.41	8574.16	124
H32C	11157.37	3599.16	8032	124
H2	5180(20)	8308(18)	2335(12)	77(5)
H1	5980(20)	5348(18)	3102(12)	77(5)
H30D	-720(20)	9990(50)	-2370(30)	70(12)
H30E	680(70)	9530(50)	-2980(20)	87(14)
H30F	810(70)	10810(30)	-2680(30)	96(16)

Table S8 Atomic Occupancy for 8aa.

Atom	*Occupancy*	Atom	*Occupancy*	Atom	*Occupancy*
H30A	0.56(3)	H30B	0.56(3)	H30C	0.56(3)
H30D	0.44(3)	H30E	0.44(3)	H30F	0.44(3)

Experimental

Single crystals of $C_{16}H_{11}NO_2$ **8aa** were were crystallized by slow evaporation of saturated solution in EtOAc. A suitable crystal was selected and mounted on the glass stick by acrylic glue on a **SuperNova, Dual, Cu at home/near, AtlasS2** diffractometer. The crystal was kept at 293(2) K during data collection. Using Olex2 [1], the structure was solved with the SHELXT [2] structure solution program using Intrinsic Phasing and refined with the SHELXL [3] refinement package using Least Squares minimisation.

Crystal structure determination of 8aa

Crystal Data for $C_{16}H_{11}NO_2$ (M =249.26 g/mol): triclinic, space group P-1 (no. 2), a = 8.2873(2) Å, b = 10.3532(2) Å, c = 15.0257(3) Å, α = 72.683(2)°, β = 81.630(2)°, γ = 77.035(2)°, V = 1195.09(5) Å^3, Z = 4, T = 293(2) K, μ(Cu Kα) = 0.745 mm^{-1}, $Dcalc$ = 1.385 g/cm^3, 33994 reflections measured (6.184° ≤ 2Θ ≤ 152.418°), 4973 unique (R_{int} = 0.0486, R_{sigma} = 0.0226) which were used in all calculations. The final R_1 was 0.0405 (I > 2σ(I)) and wR_2 was 0.1224 (all data).

Refinement model description

Number of restraints - 3, number of constraints - unknown.

Details:

1. Fixed Uiso
 At 1.2 times of:
  All C(H) groups
 At 1.5 times of:
  All C(H,H,H) groups
2. Restrained distances
 C30-H30A ≈ C30-H30F
 with sigma of 0.02
 C30-H30B ≈ C30-H30D
 with sigma of 0.02
 C30-H30C ≈ C30-H30E
 with sigma of 0.02
3. Others
 Sof(H30D)=Sof(H30E)=Sof(H30F)=1-FVAR(1)
 Sof(H30A)=Sof(H30B)=Sof(H30C)=FVAR(1)
4.a Aromatic/amide H refined with riding coordinates:
 C11(H11), C15(H15), C16(H16), C20(H20), C21(H21), C22(H22), C23(H23),
 C24(H24), C25(H25), C26(H26), C27(H27), C28(H28), C29(H29), C31(H31)
4.b Idealised Me refined as rotating group:
 C30(H30A,H30B,H30C), C32(H32A,H32B,H32C)

5. X-Ray Crystallography data for (*Z*)-5-Ethyl-2-(hydroxyimino)-2*H*-spiro[benzofuran-3,3'-indolin]-2'-one **13ab**

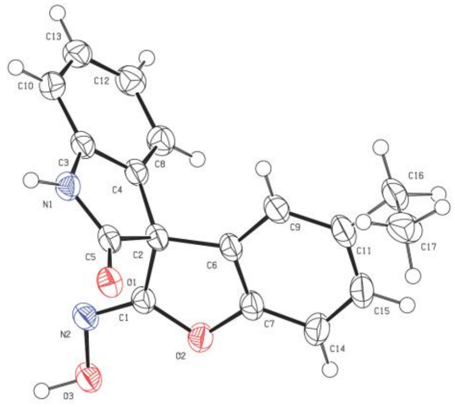

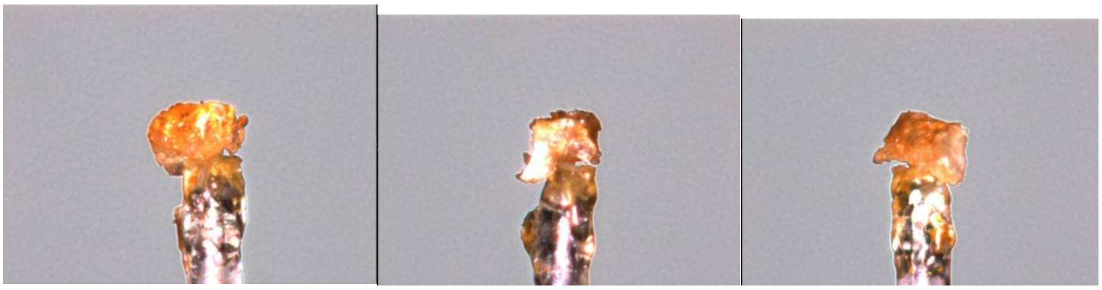

Table S1 Crystal data and structure refinement for

13ab. Identification code ANNA_ARK1266a_1

Empirical formula	$C_{17}H_{14}N_2O_3$
Formula weight	294.30
Temperature/K	100.00(11)
Crystal system	monoclinic
Space group	C2/c
a/Å	15.7588(2)
b/Å	7.03540(10)
c/Å	26.3418(3)
α/°	90
β/°	99.9520(10)
γ/°	90
Volume/Å^3	2876.56(6)
Z	8
ρ_{calc}g/cm^3	1.359
μ/mm^{-1}	0.777
F(000)	1232.0
Crystal size/mm^3	0.34 × 0.25 × 0.19
Radiation	Cu Kα (λ = 1.54184)
2Θ range for data collection/°	6.814 to 151.892
Index ranges	-19 ≤ h ≤ 19, -8 ≤ k ≤ 8, -33 ≤ l ≤ 30
Reflections collected	21171
Independent reflections	3000 [R_{int} = 0.0633, R_{sigma} = 0.0270]
Data/restraints/parameters	3000/1/216
Goodness-of-fit on F^2	1.052
Final R indexes [I>=2σ (I)]	R_1 = 0.0470, wR_2 = 0.1290
Final R indexes [all data]	R_1 = 0.0498, wR_2 = 0.1328
Largest diff. peak/hole / e Å^{-3}	0.25/-0.31

Table S2 Fractional Atomic Coordinates (×10⁴) and Equivalent Isotropic Displacement Parameters (Å²×10³) for 13ab. U_{eq} is defined as 1/3 of the trace of the orthogonalised U_{IJ} tensor.

Atom	*x*	*y*	*z*	U(eq)
O1	4885.8(6)	7116.8(15)	4153.8(4)	32.9(2)
O2	4002.3(7)	2125.6(14)	3951.4(4)	32.7(3)
O3	4260.2(7)	1694.3(15)	4926.9(4)	35.7(3)
N1	3638.9(8)	8313.9(17)	4372.7(4)	30.9(3)
N2	3951.5(8)	3504.5(17)	4752.3(4)	31.8(3)
C1	3829.8(8)	3583.9(19)	4264.3(5)	28.1(3)
C2	3508.9(8)	5356.9(19)	3957.0(5)	27.1(3)
C3	2763.4(9)	7811(2)	4302.3(5)	29.6(3)
C4	2643.6(9)	6068(2)	4050.4(5)	28.1(3)
C5	4113.4(9)	7032.3(19)	4165.5(5)	28.4(3)
C6	3569.3(8)	4682(2)	3419.4(5)	29.1(3)
C7	3853.3(9)	2822(2)	3449.6(5)	31.3(3)
C8	1833.2(9)	5273(2)	3937.2(6)	35.0(3)
C9	3375.1(9)	5558(2)	2942.5(5)	34.4(3)
C10	2088.2(10)	8807(2)	4448.8(5)	34.8(3)
C11	3485.7(9)	4532(3)	2501.5(5)	39.5(4)
C12	1140.0(10)	6257(2)	4080.5(6)	38.0(3)
C13	1270.4(10)	7995(2)	4331.7(6)	37.3(3)
C14	3971.0(10)	1755(2)	3026.7(6)	38.4(3)
C15	3780.6(10)	2662(3)	2554.4(6)	41.7(4)
C16	3337.3(13)	5422(4)	1977.2(6)	42.1(5)
C17	4183.7(13)	6053(4)	1829.9(8)	50.1(6)

Table S2 Fractional Atomic Coordinates (×10[4]) and Equivalent Isotropic Displacement Parameters (Å[2]×10[3]) for 13ab. U_{eq} is defined as 1/3 of the trace of the orthogonalised U_{IJ} tensor.

Atom	*x*	*y*	*z*	U(eq)
C18	4382(12)	7220(40)	2000(8)	50.1(6)
C19	3526(12)	6260(40)	2009(7)	42.1(5)

Table S3 Anisotropic Displacement Parameters (Å[2]×10[3]) for 13ab. The Anisotropic displacement factor exponent takes the form: $-2\pi^2[h^2a^{*2}U_{11}+2hka^*b^*U_{12}+...]$.

Atom	U_{11}	U_{22}	U_{33}	U_{23}	U_{13}	U_{12}
O1	32.4(5)	37.1(5)	27.6(5)	-2.5(4)	0.7(4)	-4.5(4)
O2	41.2(6)	28.7(5)	27.5(5)	-3.0(4)	3.9(4)	0.5(4)
O3	47.7(6)	27.5(5)	28.2(5)	0.3(4)	-3.7(4)	0.5(4)
N1	36.0(6)	27.2(6)	27.9(5)	-2.6(4)	1.4(5)	-1.8(5)
N2	37.4(6)	28.6(6)	26.7(6)	1.2(4)	-2.2(5)	1.0(5)
C1	30.2(6)	27.1(6)	25.6(6)	-2.9(5)	0.4(5)	-2.0(5)
C2	29.5(6)	28.7(7)	21.1(6)	-1.2(5)	-1.0(5)	-2.0(5)
C3	34.3(7)	30.6(7)	22.1(6)	2.1(5)	0.0(5)	0.5(5)
C4	31.4(7)	30.3(7)	21.1(6)	1.2(5)	0.8(5)	0.3(5)
C5	34.1(7)	28.6(6)	20.5(6)	1.1(5)	-0.7(5)	-2.3(5)
C6	26.5(6)	36.9(7)	22.1(6)	-4.0(5)	-0.6(5)	-2.3(5)
C7	30.6(7)	35.6(7)	26.2(6)	-5.4(5)	1.1(5)	-3.8(5)
C8	34.1(7)	35.9(7)	33.6(7)	-2.5(6)	2.3(5)	-2.5(6)
C9	30.5(6)	46.6(8)	24.6(6)	0.6(6)	0.3(5)	2.2(6)
C10	42.1(8)	34.1(7)	27.4(6)	-0.8(5)	3.8(5)	5.8(6)
C11	29.7(7)	64.1(10)	22.7(6)	-3.3(6)	-0.8(5)	-1.2(7)
C12	32.4(7)	44.5(8)	36.4(7)	1.8(6)	4.1(6)	-0.5(6)
C13	36.7(7)	44.6(8)	30.8(7)	3.9(6)	6.5(6)	8.6(6)
C14	39.3(8)	41.4(8)	34.1(7)	-11.7(6)	5.3(6)	-2.3(6)

Table S3 Anisotropic Displacement Parameters (Å^2×10^3) for 13ab. The Anisotropic displacement factor exponent takes the form: $-2\pi^2[h^2a^{*2}U_{11}+2hka^*b^*U_{12}+...]$.

Atom	U_{11}	U_{22}	U_{33}	U_{23}	U_{13}	U_{12}
C15	35.5(7)	60.3(10)	28.4(7)	-14.2(7)	3.5(6)	-3.8(7)
C16	37.1(10)	64.3(15)	22.9(7)	0.7(8)	-0.4(6)	3.7(9)
C17	45.8(10)	66.7(15)	38.9(10)	16.4(9)	10.8(8)	13.3(10)
C18	45.8(10)	66.7(15)	38.9(10)	16.4(9)	10.8(8)	13.3(10)
C19	37.1(10)	64.3(15)	22.9(7)	0.7(8)	-0.4(6)	3.7(9)

Table S4 Bond Lengths for 13ab.

Atom	Atom	Length/Å	Atom	Atom	Length/Å
O1	C5	1.2243(18)	C6	C7	1.381(2)
O2	C1	1.3723(16)	C6	C9	1.3853(19)
O2	C7	1.3910(17)	C7	C14	1.382(2)
O3	N2	1.4116(15)	C8	C12	1.399(2)
N1	C3	1.4053(18)	C9	C11	1.404(2)
N1	C5	1.3458(18)	C10	C13	1.395(2)
N2	C1	1.2678(18)	C11	C15	1.394(3)
C1	C2	1.5250(18)	C11	C16	1.498(2)
C2	C4	1.5119(18)	C11	C19	1.79(2)
C2	C5	1.5548(18)	C12	C13	1.389(2)
C2	C6	1.5121(17)	C14	C15	1.384(2)
C3	C4	1.3916(19)	C16	C17	1.519(3)
C3	C10	1.384(2)	C18	C19	1.514(17)
C4	C8	1.379(2)			

Table S5 Bond Angles for 13ab.

Atom	Atom	Atom	Angle/˚	Atom	Atom	Atom	Angle/˚
C1	O2	C7	106.80(11)	C7	C6	C2	108.41(12)
C5	N1	C3	112.29(12)	C7	C6	C9	119.52(13)
C1	N2	O3	110.70(11)	C9	C6	C2	132.04(13)
O2	C1	C2	112.00(11)	C6	C7	O2	112.85(12)
N2	C1	O2	124.41(12)	C6	C7	C14	123.75(14)
N2	C1	C2	123.54(12)	C14	C7	O2	123.40(14)
C1	C2	C5	107.81(10)	C4	C8	C12	118.56(14)
C4	C2	C1	114.19(11)	C6	C9	C11	118.80(15)
C4	C2	C5	101.92(11)	C3	C10	C13	117.16(14)
C4	C2	C6	117.43(10)	C9	C11	C16	121.75(17)
C6	C2	C1	99.88(11)	C9	C11	C19	106.0(8)
C6	C2	C5	115.79(11)	C15	C11	C9	119.32(14)
C4	C3	N1	109.52(12)	C15	C11	C16	118.86(15)
C10	C3	N1	128.33(13)	C15	C11	C19	131.4(7)
C10	C3	C4	122.15(13)	C13	C12	C8	120.42(14)
C3	C4	C2	108.49(12)	C12	C13	C10	121.40(14)
C8	C4	C2	131.18(13)	C7	C14	C15	115.83(15)
C8	C4	C3	120.31(13)	C14	C15	C11	122.78(14)
O1	C5	N1	127.02(13)	C11	C16	C17	110.73(15)
O1	C5	C2	125.38(12)	C18	C19	C11	117.4(13)
N1	C5	C2	107.57(12)				

Table S6 Torsion Angles for 13ab.

A	B	C	D	Angle/˚	A	B	C	D	Angle/˚
O2	C1	C2	C4	123.70(12)	C4	C3	C10	C13	-0.2(2)
O2	C1	C2	C5	-123.83(11)	C4	C8	C12	C13	-0.1(2)

Table S6 Torsion Angles for 13ab.

A	B	C	D	Angle/˚	A	B	C	D	Angle/˚
O2	C1	C2	C6	-2.52(13)	C5	N1	C3	C4	2.16(16)
O2	C7	C14	C15	179.09(13)	C5	N1	C3	C10	-177.89(13)
O3	N2	C1	O2	-1.96(19)	C5	C2	C4	C3	-3.34(13)
O3	N2	C1	C2	-179.18(11)	C5	C2	C4	C8	177.85(14)
N1	C3	C4	C2	1.08(14)	C5	C2	C6	C7	117.01(13)
N1	C3	C4	C8	-179.97(12)	C5	C2	C6	C9	-65.00(19)
N1	C3	C10	C13	179.83(13)	C6	C2	C4	C3	-130.97(12)
N2	C1	C2	C4	-58.78(17)	C6	C2	C4	C8	50.2(2)
N2	C1	C2	C5	53.69(17)	C6	C2	C5	O1	-48.77(18)
N2	C1	C2	C6	175.00(13)	C6	C2	C5	N1	133.26(12)
C1	O2	C7	C6	-1.35(15)	C6	C7	C14	C15	-0.3(2)
C1	O2	C7	C14	179.16(13)	C6	C9	C11	C15	0.5(2)
C1	C2	C4	C3	112.61(12)	C6	C9	C11	C16	-176.44(15)
C1	C2	C4	C8	-66.20(18)	C6	C9	C11	C19	-161.4(6)
C1	C2	C5	O1	62.04(16)	C7	O2	C1	N2	-175.02(13)
C1	C2	C5	N1	-115.92(12)	C7	O2	C1	C2	2.47(14)
C1	C2	C6	C7	1.61(13)	C7	C6	C9	C11	-0.9(2)
C1	C2	C6	C9	179.59(14)	C7	C14	C15	C11	-0.2(2)
C2	C4	C8	C12	178.77(13)	C8	C12	C13	C10	-0.1(2)
C2	C6	C7	O2	-0.30(15)	C9	C6	C7	O2	-178.58(12)
C2	C6	C7	C14	179.19(13)	C9	C6	C7	C14	0.9(2)
C2	C6	C9	C11	-178.75(13)	C9	C11	C15	C14	0.1(2)
C3	N1	C5	O1	177.76(12)	C9	C11	C16	C17	98.3(2)
C3	N1	C5	C2	-4.31(15)	C9	C11	C19	C18	82.9(16)
C3	C4	C8	C12	0.1(2)	C10	C3	C4	C2	-178.87(12)
C3	C10	C13	C12	0.2(2)	C10	C3	C4	C8	0.1(2)

Table S6 Torsion Angles for 13ab.

A	B	C	D	Angle/˚	A	B	C	D	Angle/˚
C4	C2	C5	O1	-177.45(12)	C15	C11	C16	C17	-78.6(2)
C4	C2	C5	N1	4.58(13)	C15	C11	C19	C18	-75.9(18)
C4	C2	C6	C7	-122.37(13)	C16	C11	C15	C14	177.10(16)
C4	C2	C6	C9	55.6(2)	C19	C11	C15	C14	156.6(9)

Table S7 Hydrogen Atom Coordinates (Å×10^4) and Isotropic Displacement Parameters (Å^2×10^3) for 13ab.

Atom	*x*	*y*	*z*	U(eq)
H8	1747.36	4082.29	3765.74	42
H9	3170.71	6830.92	2914.37	41
H10	2177.9	9994.15	4621.69	42
H12	576.62	5733.32	4005.35	46
H13	792.36	8644.74	4425.92	45
H14	4170.07	478.61	3058.16	46
H15	3853.85	1981	2253.46	50
H16A	2950.81	6532.13	1974.67	51
H16B	3052.23	4492.97	1720.7	51
H17A	4083.59	6466.71	1469.16	75
H17B	4591.42	4989.87	1873.75	75
H17C	4422.17	7111.17	2051.58	75
H3	4497(15)	1970(30)	5285(9)	61(6)
H1	3891(13)	9390(30)	4559(8)	53(6)
H18A	4356.33	7915.75	1674.7	75
H18B	4838.99	6264.23	2030.61	75
H18C	4506.08	8117.34	2288.85	75
H19A	3342.62	5633.99	1671.27	51

Table S7 Hydrogen Atom Coordinates (Å×10⁴) and Isotropic Displacement Parameters (Å²×10³) for 13ab.

**Atom**	***x***	***y***	***z***	**U(eq)**
H19B	3096.56	7258.44	2041.91	51

Table S8 Atomic Occupancy for 13ab.

**Atom**	***Occupancy***	**Atom**	***Occupancy***	**Atom**	***Occupancy***
C16	0.902(4)	H16A	0.902(4)	H16B	0.902(4)
C17	0.902(4)	H17A	0.902(4)	H17B	0.902(4)
H17C	0.902(4)	C18	0.098(4)	H18A	0.098(4)
H18B	0.098(4)	H18C	0.098(4)	C19	0.098(4)
H19A	0.098(4)	H19B	0.098(4)		

Experimental

Single crystals of $C_{17}H_{14}N_2O_3$ **13ab** were crystallized by slow evaporation of saturated solution in EtOAc. A suitable crystal was selected and mounted on the glass stick by acrylic glue on a **SuperNova, Dual, Cu at home/near, AtlasS2** diffractometer. The crystal was kept at 100.00(11) K during data collection. Using Olex2 [1], the structure was solved with the SHELXT [2] structure solution program using Intrinsic Phasing and refined with the SHELXL [3] refinement package using Least Squares minimisation.

Crystal structure determination of 13ab

Crystal Data for $C_{17}H_{14}N_2O_3$ (*M* =294.30 g/mol): monoclinic, space group C2/c (no. 15), *a* = 15.7588(2) Å, *b* = 7.03540(10) Å, *c* = 26.3418(3) Å, *β* = 99.9520(10)°, *V* = 2876.56(6) Å^3, *Z* = 8, *T* = 100.00(11) K, μ(Cu Kα) = 0.777 mm^{-1}, *Dcalc* = 1.359 g/cm^3, 21171 reflections measured (6.814° ≤ 2Θ ≤ 151.892°), 3000 unique (R_{int} = 0.0633, R_{sigma} = 0.0270) which were used in all calculations. The final R_1 was 0.0470 (I > 2σ(I)) and wR_2 was 0.1328 (all data).

Refinement model description

Number of restraints - 1, number of constraints - unknown.

Details:

1. Fixed Uiso
 At 1.2 times of:
  All C(H) groups, All C(H,H) groups
 At 1.5 times of:
  All C(H,H,H) groups
2. Restrained distances

C16-C17 ≈ C19-C18
with sigma of 0.02
3. Uiso/Uaniso restraints and constraints
Uanis(C18) = Uanis(C17)
Uanis(C19) = Uanis(C16)
4. Others
Sof(C18)=Sof(H18A)=Sof(H18B)=Sof(H18C)=Sof(C19)=Sof(H19A)=Sof(H19B)=1-FVAR(1)
Sof(C16)=Sof(H16A)=Sof(H16B)=Sof(C17)=Sof(H17A)=Sof(H17B)=Sof(H17C)=FVAR(1)
5.a Secondary CH2 refined with riding coordinates:
C16(H16A,H16B), C19(H19A,H19B)
5.b Aromatic/amide H refined with riding coordinates:
C8(H8), C9(H9), C10(H10), C12(H12), C13(H13), C14(H14), C15(H15)
5.c Idealised Me refined as rotating group:
C17(H17A,H17B,H17C), C18(H18A,H18B,H18C)

5. References

1. Dolomanov, O. V.; Bourhis, L. J.; Gildea, R. J.; Howard, J. A. K.; Puschmann, H., OLEX2: a complete structure solution, refinement and analysis program. *J. Appl. Crystallogr.* **2009**, *42* (2), 339-341.
2. Sheldrick, G. M., SHELXT - Integrated space-group and crystal-structure determination. *Acta Crystallogr., Sect. A: Found. Adv.* **2015**, *71* (1), 3-8.
3. Sheldrick, G. M., Crystal structure refinement with SHELXL. *Acta Crystallogr., Sect. C: Struct. Chem.* **2015**, *71* (1), 3-8.
